# Talkin’ About a Revolution. Changes and Continuities in Fruit Use in Southern France From Neolithic to Roman Times Using Archaeobotanical Data (ca. 5,800 BCE – 500 CE)

**DOI:** 10.3389/fpls.2022.719406

**Published:** 2022-02-07

**Authors:** Laurent Bouby, Vincent Bonhomme, Manon Cabanis, Frédérique Durand, Isabel Figueiral, Laurie Flottes, Philippe Marinval, Lucie Martin, Laure Paradis, Rachël Pinaud, Jérôme Ros, Núria Rovira, Margaux Tillier

**Affiliations:** ^1^ISEM, Université Montpellier, CNRS, IRD, EPHE, Montpellier, France; ^2^Institut national de recherches en archéologie préventive (INRAP), Paris, France; ^3^TRACES, Université Jean Jaurès, CNRS, Ministère de la Culture, Toulouse, France; ^4^Archeodunum SAS, Chaponnay, France; ^5^ASM, Université Paul Valery-Montpellier 3, CNRS, MCC, INRAP, Montpellier, France; ^6^Laboratoire d’archéologie préhistorique et anthropologie, Université de Genève, Geneva, Switzerland; ^7^EDYTEM, Le Bourget-du-Lac, France; ^8^Ipso Facto Scop, Arles, France

**Keywords:** fruit uses, domestication, arboriculture, archaeobotany, biogeography, diffusion, management practices, Mediterranean

## Abstract

The use and socio-environmental importance of fruits dramatically changed after the emergence of arboriculture and fruit domestication in the eastern Mediterranean, between the 5th and the 3rd millennia BCE. Domesticated fruits together with cultivation techniques apparently reached the western Mediterranean *via* colonial activities during the 1st millennium BCE – early 1st millennium CE. However, the pace and chronology of this diffusion as well as the recompositions in diversity, to adapt to new socio-environmental conditions, remain poorly known. In this study we investigate archaeobotanical records in Southern France from the Neolithic to the end of the Roman empire (ca. 5,800 BCE – 500 CE) to assess changes in fruit use as well as the emergence, spread and evolution of fruit cultivation. We explore changes in native traditions faced with innovations brought by Mediterranean colonization and how domesticated fruit cultivation spread from the Mediterranean to more temperate areas. Archaeobotanical data from 577 assemblages were systematically analyzed distinguishing two datasets according to preservation of plant remains (charred *vs*. uncharred), as this impacts on the quantity and diversity of taxa. The 47 fruit taxa identified were organized in broad categories according to their status and origin: exotic, allochtonous cultivated, indigenous cultivated, wild native. We also analyzed diversity, quantity of fruits compared to the total of economic plants and spatio-temporal variations in the composition of fruit assemblages using correspondence factor analyses. Archaeobotanical data reflect variations and continuities in the diversity of species used through time and space. In the Mediterranean area, significant changes related to the arrival of new plants and development of fruit cultivation occurred mainly, first during the Iron Age (6th-5th c. BCE), then in the beginning of the Roman period. Large cities played a major role in this process. In agreement with archeological information, archaeobotanical data reveal the predominance of viticulture in both periods. However, arboriculture also included other fruit species that have been subject to less intensive and specialized cultivation practices. Most significantly, this study pinpoints the continuous contribution of native, supposedly wild fruits throughout the chronology. Despite the homogenizing Roman influence, results reveal clear differences between the Mediterranean and temperate regions.

## Introduction

Eaten fresh, dried or transformed according to different procedures (pressing, cooking, fermentation…), fruits are valuable components of human diet (Maurizio, 1932; Brothwell and Brothwell, 1998). They provide not only sugars, vitamins, mineral salts, and fiber, but also lipids and proteins (Tiwari and Cummins, 2013; FAO, 2017). Furthermore, they can be charged with cultural and symbolic values, which can be reflected in social (Flandrin and Montanari, 1999; Toussaint-Samat, 2008) or ritual contexts (Bouby and Marinval, 2004; Rottoli and Castiglioni, 2011). Many fruits and their subproducts possess characteristics that can make them considered luxury foods as they are unessential to diet, difficult to obtain in large quantities, labor-intensive to transform, able to produce sweetened products or alcoholic drinks (van der Veen, 2003; Ouerfelli, 2016). Fruits and fruit tree cultivation are keystones of Mediterranean landscapes, food traditions and economies. However, in the Western Mediterranean, domesticated fruit species and cultivation techniques are considered as being foreign and of late introduction after the onset of agriculture.

We recall that the emergence of arboriculture is considered as a major change in the history of agriculture. Wild fruits were regularly eaten by Early Holocene hunter-gatherers (Zvelebil, 1994; Martinoli and Jacomet, 2004; Bishop et al., 2015) and by Neolithic populations (Colledge and Conolly, 2007; Antolín, 2016) all-over Europe and the Mediterranean but arboriculture allowed fruits to gain economic and cultural importance. Without excluding older attempts, the onset of fruit-tree domestication and large-scale cultivation crystallized in the Eastern Mediterranean and South-West Asia during the Later Neolithic and Bronze Age, about the 5th-3rd millennia BCE (Zohary and Spiegel-Roy, 1975; Weiss, 2015; Fuller and Stevens, 2019). It was interconnected with the development of urbanization, social complexification and commercial exchanges (Renfrew, 1972; Gilman, 1981; Halstead, 1992; Hamilakis, 1996; Fall et al., 1998; Sherratt, 1999). Specialized arboriculture involved new specific agricultural techniques and practices compared to those applied to annual species grown since the onset of the Neolithic. Among the most distinctive and recurring are pruning (i.e., removal of unnecessary branches to control tree growth and ensure the quantity and quality of fruit production) and vegetative propagation (i.e., cuttings, grafting). Most fruit trees require vegetative propagation to maintain and disseminate cultivars, which are basically selected clones (Zohary and Spiegel-Roy, 1975; Ladizinsky, 1998; Zohary, 2004). Fruit growing implies long-term investment and therefore long lasting settlement. Generally, fruit trees only become profitable several years after plantation but require constant care from that time onward. On the other hand, they will bear fruit for many seasons. The hierarchized societies that emerged in the Eastern Mediterranean during the Later Neolithic and Bronze Age (ca 4,250-2,000 BCE) are regarded as offering the required conditions to develop intensive fruit production: a strong degree of attachment to the land, extensive commercial networks and capital investment capacity. In return, fruit trees, especially the emblematic Mediterranean species that are grapevine (*Vitis vinifera*), olive (*Olea europaea*), fig (*Ficus carica*), and date palm (*Phoenix dactylifera*), provide products that preserve easily, can be transported over long distances and have high commercial value (oil, wine, dried fruits). Thus, they were likely to offer a large income for capital investors.

Fruit tree cultivation is considered to have spread only secondarily from its Eastern cradle to the whole Mediterranean basin and Europe. This process was fueled by the colonial and trading activities developed by the technologically advanced and powerful Mediterranean societies, during the 1st millennium BCE (Phoenicians, Greeks, Etruscans) and the following centuries with the building of the Roman Empire (Sherratt and Sherratt, 1993; van Dommelen, 2012; Hodos et al., 2016). Connectivity in the Mediterranean increased progressively and fruits started to travel early. The transport of olives and pomegranates is attested as early as the Late Bronze Age (1,350-1,300 BCE) by the Uluburun shipwreck off the coast of Turkey (Haldane, 1993). The transport of fruits and fruit-products became commonplace all-over the Mediterranean and Europe in Roman times (André, 2009). The spread of fruit species and arboriculture techniques must have occurred simultaneously. However, the pace and exact chronology of their diffusion in the Western Mediterranean, the role played by the different Mediterranean societies, as well as the adaptation of arboriculture and fruit cultivation to new territories remain largely unknown. This ‘globalization’ necessarily implied changes in the diversity and distribution of fruit species according to time, environmental conditions, economic context and cultural preferences. Archeology allows us to track the diffusion of new arboricultural practices only when specialized activities leaving diagnostic traces and artifacts are involved. For example, the early diffusion of viticulture was recognized in Huelva (Southern Spain), based on the discovery of vine plantation pits dating back to the 9th c. BCE (Echevarria Sanchez and Vera Rodriguez, 2015) and in Marseille (Southern France), where the production of typical wine amphorae started during the 6th c. BCE (Bats, 1990; Bertucchi, 1992). However, these activities require a high degree of specialization and production levels while the domestic use of fruits and small-scale cultivation do not require such specialized equipment and may go unnoticed. Archaeobotanical fruit remains, on the other hand, although unable to provide conclusive evidence of local cultivation, allow us to track the use of fruits in a large number of sites, regardless of their exact chronology, location and socio-economic status. As a result, it becomes possible to investigate the diversity of species consumed in the past and its recombination through space and time. In this study we use archaeobotanical data as a proxy to assess how fruit exploitation changed in Southern France from the Neolithic to the end of the Roman empire (ca. 5,800 BCE – 500 CE), and how fruit growing appeared, spread and evolved. It is particularly important to explore the influence of Mediterranean trade and colonial activities on the evolution of native traditions and to follow the spread of fruit cultivation from the Mediterranean to temperate areas.

## Archeological and Environmental Setting

### Chronology, Archeological Cultures and Human Occupation

**FIGURE 1:**
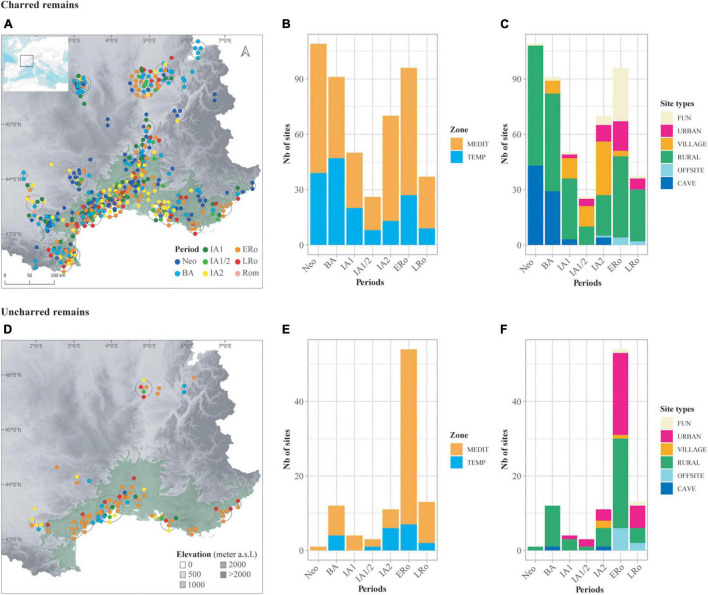
Distribution of the archeological sites included in the database according to type of preservation of plant remains, charred and uncharred remains. **(A,D)** Maps showing site location; color of symbols corresponds to period of site occupation. The distribution area of the olive tree (Leguédois et al., 2011) is represented (in light green) as the limit of the Mediterranean bioclimatic zone. Topography is derived from the BDAlti 250 m of IGN. The map was created using QGIS version 3.16. To visualize all overlapping points, symbols are distributed in a circle around the central point (QGIS Point displacement Renderer). **(B,E)** Bar plots showing the distribution of sites in relation to chronology and bioclimatic zone. **(C,F)** Bar plots showing the distribution of sites in relation to chronology and broad site categories.

The Neolithic way of life, including agriculture, was introduced in southern France (Figure 1) around 5,850 BCE, by populations of the Impressa group coming from Italy with their techno-cultural values and equipment (Manen et al., 2018; Bouby et al., 2020a). From what is known today, these colonists only settled sporadically in the littoral area, the diffusion of the Neolithic toward the interior being achieved by the subsequent Cardial complex, between 5,400 and 4,500 BCE. In Southern France, the Neolithic lasted until about 2,100 BCE, with agriculture sometimes considered to have contributed unevenly to the economy of different groups (Beeching et al., 2000) and to the population growth of the Late Neolithic (ca 3,500-2,100 BCE) (Jallot, 2007; Berger et al., 2019). In line with the Neolithic habits, the Bronze Age populations lived in isolated farms, hamlets and villages, occasionally surrounded by fortifications. Social centralization and hierarchization increased during the Late Bronze Age (LBA, ca 1,350-750 BCE) (Carozza et al., 2007), along with population density and human impact on the vegetation (Berger et al., 2019). While agriculture comprised globally the same cereals, pulses and oil-plants during the Neolithic and most of the Bronze Age, the LBA period witnessed major changes with the arrival of new domesticates, especially millets (Bouby et al., 2017).

The emergence of colonial activities occurred during the First Iron Age, mostly after the foundation of Marseille by Greek settlers (Phoeceans) ca. 600 BCE, but commercial and cultural exchanges also involved Etruscans and Phoenicians. Food products, especially wine, played a prominent part in colonial relations and in the transformation of indigenous societies (Dietler, 2007a,b). The advent of urbanization, from the middle of the 6th century BCE onward, is one of the most obvious symptoms of these transformations (Garcia, 2004).

Changes occurred toward the end of the Iron Age with the rapidly increasing activity of Roman merchants followed by Roman colonization, which started in 121 BCE and consolidated after the conquest of inner Gaul, by 50 BCE. During the Roman period, the effects of acculturation were deeply felt, with the establishment of colonies of Roman veterans, centuriation in most of south-eastern France and the emergence of large urban centers (e.g., Narbonne, Nîmes, Lyon) sharing the same characteristics as the cities of Italy. From the end of the Iron Age onward, the number of excavated archeological sites testifies to the peak of population density during the Roman period, especially during the first two centuries CE (Berger et al., 2019). During this period, viticulture flourished and local wine was extensively exported to different parts of the Roman Empire, including Rome (Brun, 2010).

### Biogeographical Background

Our geographical area (Figure 1) covers the mediterranean plains and plateaux, also extending to the north and north-west, to areas with a more temperate climate, mainly along the main alluvial valleys (Mottet, 1993). This area is constrained by the mountain ranges of the Alps to the east, the Massif Central to the north-west and the Pyrenees to the south-west. The axis of the Aude-Garonne valleys to the west and the Rhône to the north were major communication axes between the mediterranean world and central and northern Europe (Braudel, 1979). In Roman times, this area was an integral part of, and made up most of, the province of *Gallia Narbonensis*.

Most of the sites considered in this study are located at an altitude of under 400 m, with a few sites between 500 and 1000 m. They are to be found in the plains, hills and plateaux of the Rhône and Aude valleys and the lower parts of their tributaries, as well as the basins of some coastal rivers, the littoral plain and some inland basins such as the Limagnes d’Auvergne. Limestone bedrock predominates. Deep soils, the most favorable for agriculture, are largely limited to the valleys and basins. Traditionally, specialized arboriculture was taking place on these soils, although viticulture and oleiculture was largely spread to ancient alluvial terrasses, hillsides and slopes. Domestic fruit cultivation could be performed on virtually every farm.

In the southern part, climate is typically mediterranean, hot and dry during the summer, with irregular rainfall (with sporadic downpours) and frequent fierce winds. The vegetation belongs to either the thermomediterranean level (by the coast) or the mesomediterranean level (elsewhere). Both are characterized by evergreen formations (forests and garrigues) dominated by evergreen oak (*Quercus ilex*) (Quézel and Médail, 2003). The climate gradually becomes oceanic (mild and humid) toward the west and more continental (cold winters and more regular rainfalls) toward the north, rapidly becoming a mountainous type with altitude. As a result, the natural vegetation changes and is dominated by diversified deciduous oak forests in the lowlands and mid-mountains. The mountain and subalpine vegetation predominate above 750 m high. In this article we divide our sites into two major biogeographical groups: Mediterranean and temperate. In the traditional way, we consider as Mediterranean the sites that are located in the area where the olive tree currently grows (Leguédois et al., 2011).

## Materials and Methods

### Choice of Sites

Our database is composed of both published and unpublished archaeobotanical data (Supplementary Table 1). To better evaluate the overall recurrence of fruits, all sites with archaeobotanical information are taken into account, whether they contain fruit remains or not. Annual crops present at each site are also recorded to assess the quantitative importance of the fruits, by comparison.

The chronological boundaries range from 5,850 BCE, corresponding to the earliest Neolithic settlement, to 500 CE, which roughly marks the end of the Roman Empire in Western Europe. Dates are based on stratigraphy, calibrated carbon dating and archeological artifacts. The data were recorded according to the chrono-cultural phases identified on the sites (site-phases) but when several samples were available for a given phase, they are combined to compose a single entry in the database. Assemblages were subsequently assigned to a broad chrono-cultural period: Neolithic (Neo: 5,850-2,200 BCE), Bronze Age (BA: 2,200-750 BCE), First Iron Age (IR1: 750-525 BCE), Transition First/Second Iron Age (IA1/2: 525-425 BCE), Second Iron Age (IA2: 425-50 BCE), Early (ERo: 50 BCE-250 CE) and Late Roman Empire (LRo: 250-500 CE). As they correspond to the emergence of colonial contacts in the Mediterranean the Iron Age and Roman period have been divided into subphases.

For each site and phase the archaeobotanical information was recorded as different entries according to preservation of macroremains. Provided they have been introduced and processed at the sites and the sediments have been properly sieved, all fruit species can be documented in the archaeobotanical record. The main limitation here is whether or not they are preserved over time in the sediments. Type of preservation has a major impact on the composition and diversity of the fruits and wild plants recorded in a site (Colledge and Conolly, 2014; Antolín and Jacomet, 2015). Therefore, data from charred and uncharred remains were recorded and analyzed separately (Figure 1). Uncharred items are composed of waterlogged plant remains, with the very rare exception of desiccated material (Beaume Layrou site). Mineralized plant remains are very seldom found in our area for the periods under consideration and were therefore not included in the database.

Most of the sites are located in the Mediterranean area with 394 site-phases vs. 183 site-phases in the temperate bioclimatic zone. More data are available for charred plant remains (479 site-phases) than for uncharred remains (98 site-phases). The chronological distribution of the sites also shows some variability between periods. IA1, IA1/2, and LRo are the periods with less data but they are also among the shorter time-spans. A large proportion of the uncharred assemblages are dated to the ERo. Their frequency at this period is favored by the number of wells in rural sites and the number of ports that were excavated.

Sites were classified in broad categories according to their main characteristics: cave/rock-shelter (CAVE), rural (RURAL), small agglomeration (VILLAGE), urban (URBAN) offsite (OFFSITE) and funerary/ceremonial (FUN). The first four categories mostly relate to domestic occupations (habitats) but they can also include more specialized functions (crafting activities, animal husbandry, etc.). Site functions are often difficult to characterize precisely and to disentangle. The offsite records correspond to paleochannels, ditches or basin type structures located near human dwellings. Even using these very broad categories, some site types are strongly associated with particular periods, which hinders diachronic comparisons. Most of the cave/rock-shelter occupations are Neolithic or Bronze Age, whereas funerary/ceremonial sites and ‘offsites’ are mainly Roman. Urban sites do not exist before the Iron Age. Only rural sites are regularly recorded all along the chronology. In total, mainly domestic assemblages have been studied.

### Taxa Selection and Standardization

As our study associates diverse datasets from different archaeobotanists it was necessary to combine and standardize taxa names in order to make samples and sites more readily comparable (Colledge, 2001; Bogaard, 2004). Therefore, taxonomic synonyms and initial identifications were amalgamated when they are likely to represent the same taxa. This standardization was based on the level of identification most commonly considered appropriate for the taxon in question, when fruit and seeds are well preserved. In particular, tentative and confident identifications were grouped together in the last category (e.g., *Cucumis* cf. *sativus* under *C. sativus*). Very rare, uncertain or dubious identifications (*Prunus armeniaca*, cf. *Castanea sativa*) were excluded. A few taxa (*Celtis australis*, *Cupressus sempervirens*), whose fruits are only occasionally eaten, have nevertheless been kept in the database because of their symbolic and cultural value during ancient times.

Fruit taxa were classified in broad categories according to status and origin (Bakels and Jacomet, 2003; Livarda, 2011): EXOTIC (non-native species that cannot grow in the study area and whose fruits were necessarily imported), ALLOCULT (non-native species that can be successfully acclimatized and cultivated in the area), INDCULT (native species that were domesticated and regularly cultivated), WILD (native species never domesticated).

### Quantitative Methods and Data Analysis

For the analyses, it was also necessary to standardize the method of quantification between sites (Wallace et al., 2019). The most common method used in the primary data was the counting of plant remains. When entire seeds (NER) and fragments (NF) were counted separately, the Minimum number of individuals (MNI) was calculated using the formula: MNI = NER + 1/2 NF. When other quantification methods were used (percentages or presence/absence) values were converted to counts. When data were recorded in presence/absence a count of 1 was assigned to each taxon recorded. In order to make representations easier and to reduce skewness of the data rough counts were log-transformed before any graphical representation based on MNI. Samples with less than 25 fruit remains were excluded from all quantitative analyses.

The economic importance of fruit in the sites was evaluated by calculating the proportion of the fruit categories in the total of economic plants, including annual crops such as cereals, pulses, technical plants, and condiments. The diversity of fruits used at the sites was appraised by calculating the taxonomic richness of fruit categories. This is not a straightforward measurement of the diversity of taxa exploited as other factors can interact, such as sample size, taphonomy and accuracy of taxonomic identifications (Martínez et al., 2016). To counteract the effects of variability in identification, when this was carried out to a specific level we did not consider the higher taxonomic levels, such as genus, when calculating richness. We only took the high taxonomic categories into account when no specific identification was made at the site.

Variation in fruit taxa composition among sites was investigated using correspondence factor analysis (CFA). Raw counts of fruit remains were transformed logarithmically (logMNI + 1) to limit the influence of sample size. Only taxa recorded in at least 5% of the samples were taken into consideration in order to minimize noise brought by rare taxa (van der Veen, 1992). The CFA includes sites from all periods, separated into two datasets according to type of preservation (charred *vs*. uncharred). In addition, a second CFA was carried out exclusively on the Roman sites, as they showed limited structuring after the first global analysis. Boxplot representations are combined with a comparison of all pairs of samples using the Wilcoxon rank sum test to determine which groups can be considered as different. All analyses were performed in the R 4.0.2 environment (R Core Team, 2020). CFA were done using the package FactoMineR (Lê et al., 2008). All graphs were created using ggplot2 (Wickham, 2016).

## Results

### A Large Range of Fruits With Economic Value

**FIGURE 2:**
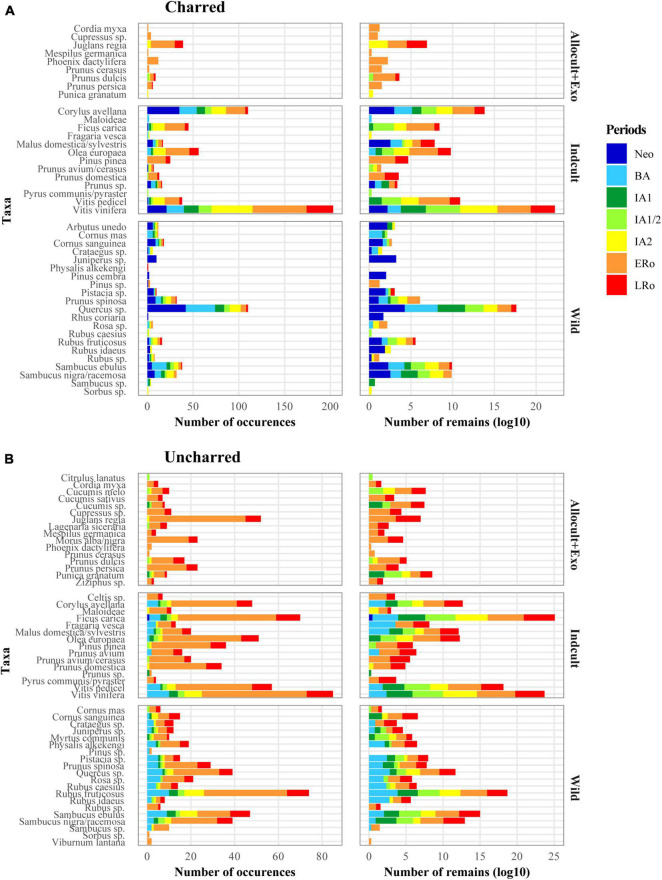
Bar plots showing the overall representation of fruit taxa within **(A)** charred and **(B)** uncharred plant remains in relation to broad fruit categories (Exotic + Allocult, Indcult, Wild) and to cultural periods. Two methods of representation are used: number of occurrences of taxa in all sites, log-transformed (log10) raw counts of fruits in all the sites.

A wide spectrum of economically valuable fruits was recognized with a minimum of 47 different taxa (Supplementary Table 2). Taxa diversity is high regardless of preservation type (Figure 2), most taxa being recorded in both the charred and uncharred state. However, as might be expected, due to well-known taphonomic issues, the diversity recorded was slightly higher for uncharred material, although the number of phases was more than four times lower for this type of preservation. Moreover, taxa tended to be more ubiquitous and quantitatively better represented when preserved uncharred. This bias is mainly due to the fact that charring favors (1) fruits that were regularly roasted prior to storage or consumption and (2) woody fruits that are more resistant to charring. Some of them, especially olive pressings, may even have been used as fuel (Rowan, 2015). Fruits usually eaten raw are more likely to be recorded uncharred. One must then bear in mind that even occasional finds of charred fruit with well-known economic properties might reflect important economic resources for past populations (Colledge and Conolly, 2014; Antolín and Jacomet, 2015).

Many of the recorded fruits (Nb = 21) are native plants that were never domesticated (WILD). Wild taxa are strongly represented in the Neolithic and Bronze Age periods but, in general, their presence remains constant until the end of the Roman times, pointing to a long-lasting use. The most common wild fruits are acorns (*Quercus* sp.), sloes (*Prunus spinosa*) and small berries, including brambles (*Rubus fructicosus* more especially) and elderberries (*Sambucus ebulus*, *S. nigra/racemosa*). Wild taxa are generally considered as natural resources gathered locally. The situation is less clear for other 12 indigenous taxa classified in category INDCULT, as they were domesticated in the Mediterranean and may have achieved at some point the status of cultivated plants in the study area. This category includes some of the best-represented taxa from a quantitative point of view: first of all grapevine (*Vitis vinifera*) but also hazel (*Corylus avellana*), fig (*Ficus carica*), and olive (*Olea europaea*). Interestingly, grapevine is most often documented as pips, but pedicels also appear frequently. All the most common INDCULT taxa are recorded from the Neolithic or Bronze Age until the Roman period. A wide range of cultivated fruit taxa of non-native origin (ALLOCULT) is also listed. The 13 taxa in this group are less frequent than those in the previous categories. In addition, the earliest records in the ALLOCULT group only date back to the First Iron Age and most records concern the Roman period. The most common fruit tree in this category is the walnut (*Juglans regia*). Strictly exotic species, which can not be grown in the study area, are restricted to *Cordia myxa* and *Phoenix dactylifera*. They are only recorded in the Roman period and quite sporadically.

### Economic Weight and Diversity of Fruits

Dispite biases due to taphonomy and sampling methods, the quantity of fruit remains found may be considered as a proxy of their importance in site economy.

**FIGURE 3:**
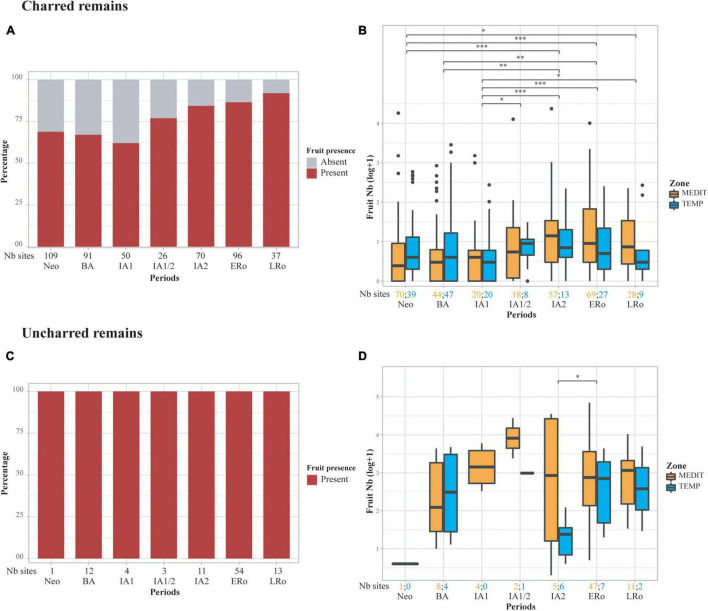
Abundance of fruit remains in the archeological sites in relation to type of preservation and cultural periods, **(A,B)** charred remains, **(C,D)** uncharred remains, **(A,C)** stacked bar plots showing the proportion of sites with fruits, **(B,D)** box plots showing the distribution of log-transformed (log10) raw counts of fruits. Statistical significances of the Wilcoxon tests are indicated on the box plots, only when the result is significant (*p ≤ 0.05; **p ≤ 0.01; ***p ≤ 0.001). The number of sites for each period and bioclimatic zone is provided at the bottom of the graph.

Fruit remains are common since the Neolithic but there is a high variability between sites regardless of the period (Figure 3). Concerning charred fruit remains, we notice an increase in their importance from the IA1/2 onward, both in terms of number of sites and amount of remains. This is particularly noticeable in the Mediterranean area; sites from the temperate zone generally provided slightly lower amounts of fruit remains. The uncharred material does not show such a clear pattern, primarily because there are fewer sites for the Neolithic and Bronze Age periods and because uncharred fruit remains are present in all sites, regardless of chronology. However, uncharred fruits appear to be generally more abundant during the Iron Age and Roman period than in the Bronze Age. Sites in the temperate zone also tend to lag behind those of the Mediterranean for this Iron Age-Roman period.

**FIGURE 4:**
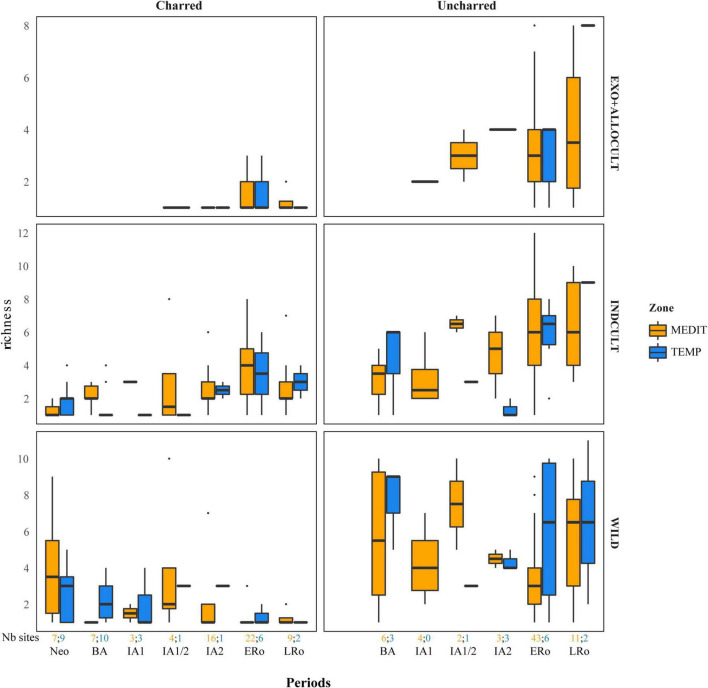
Richness of fruit taxa in the archeological sites in relation to type of preservation, categories of fruit taxa and cultural periods. Richness is calculated as the raw count of fruit taxa in assemblages of at least 25 fruit remains. The number of sites for each period and bioclimatic zone is provided at the bottom of the graph.

The trend observed concerning the abundance of fruit remains must be compared with the variations in fruit taxa richness (Figure 4). A certain decrease in the richness of charred wild fruits is observed from the Neolithic to the Roman period. In contrast, no trend is discernible in the uncharred material. The INDCULT group richness increase slightly from the IA1/2 or IA2 for charred and uncharred material alike. The ALLOCULT – EXOTIC group is identified in the Iron Age, mainly in the Mediterranean area, but it is not possible to identify a clear increase in the following periods. There is no obvious richness pattern between the two bioclimatic regions for any fruit category.

### Variations in Fruit Assemblages

**FIGURE 5:**
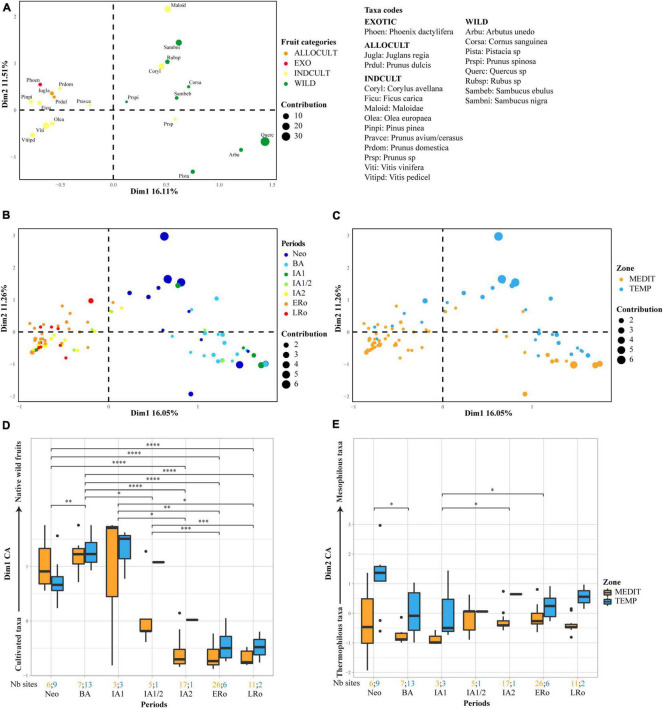
Correspondence factor analysis on charred fruit remains. First biplot of the correspondence factor analysis on log-transformed raw counts of charred fruit remains, **(A)** plot of taxa, **(B)** plot of sites according to main archeological periods, **(C)** plot of sites according to bioclimatic zones. Box plots showing the distribution of the sites on **(D)** axis 1 and **(E)** axis 2 of the CFA in relation to cultural periods and bioclimatic zones. Axis 1 displays a discrimination between cultivated taxa to the left and native wild fruits to the right. Axis 2 displays a discrimination between thermophilous taxa in the lower part and mesophilous taxa in the upper part. The number of sites for each period and bioclimatic zone is provided at the bottom of the graph. Statistical significances of the Wilcoxon tests are indicated on the box plots, only when the result is significant (*p ≤ 0.05; **p ≤ 0.01; ***p ≤ 0.001).

Variations in the composition of fruit taxa recorded at the sites were studied using correspondence factor analyses performed separately on charred (Figure 5) and uncharred (Supplementary Figure 1) fruit remains. It seems remarkable that, although the sets of taxa and the representation of sites by period are not identical for both preservation types, the analyses show very consistent results.

There is always a main discrimination between cultivated (INDCULT) and allochtonous taxa on one-hand and native wild fruits on the other. Furthermore, a distinction between thermophilous and more mesophilous taxa is observed (see Rameau et al., 1989), and this regardless of their status. For both preservation types, few taxa appear outside their *a priori* group (INDCULT gathered with WILD taxa and vice-versa). In all these cases the initial classification could be questioned. Taxa such as *Corylus avellana*, *Fragaria vesca*, and Maloideae, classified as INDCULT, could mostly represent gathered wild fruits. On the other hand, *Cornus mas* and *Myrtus communis*, although initially considered as indigenous, are known to be regularly planted. According to Pliny the elder, myrtle was commonly cultivated in ancient Rome as a sacred plant and/or for the usefulness of its fruits (Hist Nat, L XV).

The structuration of taxa is linked to an organization of sites determined mostly by chronology and geographical location. This organization is particularly clear in the CFA carried out on charred material.

Axis 1 separates two chronological groups, one assembling most of the sites from the Neolithic, BA and IA1 periods, in association with wild fruits, and the other composed of the Roman, IA2 and most of the IA1/2 sites, linked with INDCULT and ALLOCULT taxa. When we spatialized the results of the CFA on charred remains using the coordinates on axis 1, it became clear that the shift started during the IA1 and IA1/2 periods, in the Mediterranean zone (about 600-425 BCE) (Supplementary Figure 2). This shift was largely accomplished by the IA2, after 425 BCE. Unfortunately, in the temperate zone few sites provided significant charred fruit remains for that period, thus blurring our perception of the early spread of cultivated fruit taxa toward the north. For both preservation types, a slight downturn shift in the trend toward cultivated taxa is observed between ERo and LRo sites.

Our analysis systematically separated mediterranean sites, which shift toward the range of ALLOCULT, INDCULT and typical wild Mediterranean taxa, from temperate sites, mainly determined by more mesophilous wild fruit trees (*Corylus avellana*, Maloideae, *Sambucus* sp., *Rubus* sp.; Supplementary Figure 1). This shift, most noticeable on axis 2 of the CFA based on charred material, is clearly visible whatever the period, persisting until the end of the Roman period (Figure 5E).

**FIGURE 6:**
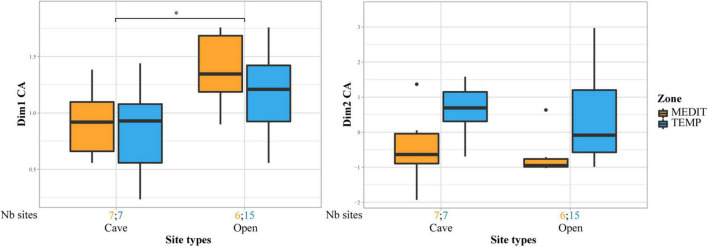
Comparison of site types for the Neolithic and Bronze Age periods. Cave sites are opposed to open air settlements (rural and village sites). Box plots showing the distribution of sites on axis 1 and 2 of the CFA performed on charred fruit remains in relation to site types and bioclimatic zones. Statistical significances of the Wilcoxon tests are indicated on the box plots, only when the result is significant (*p ≤ 0.05). The number of sites for each site category and bioclimatic zone is provided at the bottom of the graph.

Contrasts in fruit records are also associated with site categories (Figure 6). For the Neolithic and BA periods, in the charred fruits record, a shift between cave/rock-shelter occupations and rural open-air sites is noticeable on axis 1, especially in the Mediterranean region.

These global CFAs, especially the one carried out on charred material, shows a strong clustering of Roman sites within the Dim 1 × Dim 2 biplot, which points to a greater homogeneity of fruit assemblages than in previous periods. To perceive more clearly a possible structuration among Roman sites we performed specific CFAs on them.

The sets of taxa are once again different but their structuration is still largely conditioned by the discrimination between (1) cultivated and wild fruit taxa and (2) thermophilous and mesophilous taxa (Supplementary Figures 3, 4). In the CFA on charred material, axis 1 separates *Vitis* remains from all the other taxa. In the CFA on uncharred material, ALLOCULT taxa (*Lagenaria siceraria*, *Mespilus germanica*) are mixed with indigenous wild taxa in relation to the presence of offsite records. It may indicate that these plants were growing in the immediate vicinity. In the Roman CFAs, sites were generally organized according to site categories and bioclimatic regions rather than chronology.

**FIGURE 7:**
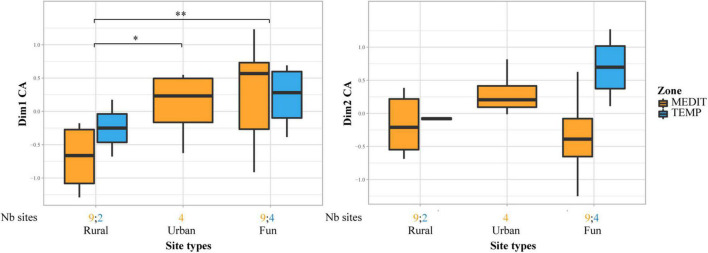
Comparison of site types for the Early Roman period. Only Rural, Urban and Funerary/ceremonial sites are taken into consideration. Box plots showing the distribution of sites on axis 1 and 2 of the CFA performed on charred fruit remains in Roman sites, in relation to site types and bioclimatic zones. Statistical significances of the Wilcoxon tests are indicated on the box plots, only when the result is significant (*p ≤ 0.05; ^**^p ≤ 0.01). The number of sites for each site category and bioclimatic zone is provided at the bottom of the graph.

The rural sites are strongly attached to INDCULT taxa, especially *Vitis vinifera*, in comparison with urban and funeral/ceremonial sites. We must underline that axis 2 of the CFA on uncharred material shows a fairly strong differentiation between fruits with pips on the one hand and stone and nut fruits on the other. This may be triggered by the presence of different types of deposits. Fecal waste generally favor the presence of pips. Those could have been better represented in our rural samples and more culinary waste, favoring stone and nut fruits, could have been more often sampled in urban contexts. The difference between site types registered by uncharred remains may then be heightened by underlying deposit differences. They should nevertheless be considered as significant as this result is in line with a similar distinction between rural and urban sites on charred material, but in this case there was no specific distinction between pips and nuts/stone fruits (Figure 7).

Funerary/ceremonial sites mostly provided charred fruit remains, particularly thermophilous fruits (*Ficus carica*, *Phoenix dactylifera*, *Pinus pinea*, *Prunus dulcis*).

The CFA on uncharred material confirms the chronological separation of ERo and LRo sites, due to a relative shift from cultivated and thermophilous fruits to wild and more mesophilous ones (Supplementary Figure 5).

## Discussion

As mentioned before, preservation conditions have a major effect on the quantity and diversity of fruit taxa recorded in archeological sites (Colledge and Conolly, 2014; Antolín and Jacomet, 2015). Our study is consistent with this tendency, with a higher quantity and diversity of fruit recorded when uncharred material is preserved. However, on the broad scale covered by this study, the trend observed in the charred fruit remains is similar to that of the uncharred material. More remarkably, charred and uncharred material show similar patterns in the spatio-temporal variation of fruit assemblages composition explored by means of CFA. This includes a chronological trend toward an increasing integration of cultivated fruits in the economy with a major shift around the transition between Iron Age 1 and 2.

### The Exploitation of Fruits During the Neolithic and Bronze Age

Our work shows that a wide range of fruits was already used during the Neolithic and Bronze Age. According to quantitative archaeobotanical data, fruit exploitation would have been almost as important as in the Iron Age 2 and Roman times. We should however consider this cautiously as methodological biases could tilt the existing pattern. On the one hand, we mainly studied domestic assemblages, which may underestimate specialized uses of fruits outside the domestic context. On the other hand, some of the most important fruits from the Neolithic and the Bronze Age are among those which preserve better when charred (e.g., acorns, hazelnuts), possibly reinforcing their quantitative weight in ancient sites.

Differences between bioclimatic zones are particularly noticeable during the Neolithic and the Bronze Age. This is easily explained as the use of fruit depended exclusively on resources available in the local vegetation, as noticed previously in eastern Iberia (Alonso et al., 2016).

Should the use of wild fruits during Neolithic be regarded as the persistence of a longstanding hunter-gatherer tradition? This could be questionable, as the thorough consideration of radiocarbon dates of the late Mesolithic and Early Neolithic sites shows that the two communities were only rarely contemporaneous in the region, offering little possibility of interactions between groups (Perrin and Manen, 2021).

Current paleogenomic data further reveal that the first Neolithic farmers in Western Europe were settlers whose genetic lineage only included a small European hunter-gatherer genetic component (Shennan, 2018). In fact, investigations into the habitats of the first Neolithic settlers on the littoral of Southern France have shown that their economy was focused on agricultural production, including very little wild plant gathering (Bouby et al., 2020a) and hunting (Vigne, 2007; Rowley-Conwy et al., 2013). These activities only increased later, with the spread of the Neolithic inland. The integration of a higher proportion of wild foods could thus be interpreted as an adaptation of the Neolithic economy to local forest resources, whether or not facilitated by interactions with Mesolithic hunter-gatherers. During the Neolithic, archaeobotanical evidence indicates that wild fruits were more widely used in the Mediterranean hinterland than on the littoral plain (Bouby et al., 2020a,b). In Spain also, charred fruits, especially hazelnuts and acorns, were more numerous in the Neolithic sites of the Pyrenean foothills and mountains (Antolín et al., 2018). Spatial variability goes together with variations according to type of site. Our study shows that the fruit record is different in cave/rock-shelter sites, to be found in the hinterland, and in open-air settlements, more common in the littoral and fluvial plains. The more prominent and/or diversified role of fruits in the economic activities of hinterland sites does not necessarily imply a larger contribution to human diet. Caves and rock-shelters often correspond to specialized activities or short-term, seasonal occupations rather than permanent habitats. Hinterland sites may have been more involved in fruit gathering or processing activities, such as roasting of fruits to improve preservation and enhance taste (Antolín and Jacomet, 2015). Furthermore, the good representation of fruits in some cave and rock-shelters can be explained by the collection of branches and leaves as fodder for livestock. In fact, sedimentological evidence showed that many caves and rock-shelters of Southern France were used as penning sites (Brochier et al., 1992). This is also supported by archaeobotanical data suggesting the use of leaf fodder, as is the case at la Grande Rivoire (Isère) (Delhon et al., 2008; Martin, 2014). Actual habitat sites, such as the Taï cave, also suggest the strong contribution of leaf fodder to the fruit record (Bouby et al., 2019).

Acorns were the most frequent and abundant fruits in Neolithic and Bronze Age sites. The CFA shows that they tend to be better represented in the South and in Bronze Age sites, and are more strongly associated with open air settlements than with caves and rock-shelters. The concentrations of charred acorns found at several Late Neolithic sites in the Mediterranean hinterland represent fruits accidentally burned during heat treatment (drying, roasting) or storage. This is particularly visible at Boussargues (Hérault), where large numbers of acorns were found in several storage rooms (Marinval, 2008). Acorns were consequently voluntarily gathered and processed in the area, representing a steady and reliable resource, rather than casual foraging; they probably supplied food to humans and livestock alike. Due to their high carbohydrate content, acorns can be a valuable substitute for cereals. According to our results, their exploitation increased during the Bronze Age, especially in the northern part of our study area. However, acorns were already eaten in northern France in the Neolithic (Martin et al., 2016).

### The Changes of the Iron Age

Significant changes in the use of fruits occurred in the Mediterranean zone during the Iron Age. As mentioned before, colonial activities of Mediterranean peoples (Greeks, Etruscans, Phoenicians) increased dramatically, mostly after the foundation of Marseille in ca. 600 BCE. The first traces of cultivated non-native fruits were actually recorded during the 6th-5th c. BCE: *Cucumis* sp., *Prunus dulcis*, *Punica granatum* followed by *Citrullus lanatus* and *Lagenaria siceraria*. All these early attestations came from the Greek city of Marseille (Bouby, 2014) and the major port of Lattara, which was involved in trade activities and included Etruscans among its population (Alonso and Rovira, 2010, 2016; Rovira and Alonso, 2018). Besides fruits, newcomers registered in these coastal sites also include a pulse (*Cicer arietinum)* (Marinval, 1988), condiments (*Allium sativum, Coriandrum sativum*, and *Foeniculum vulgare*) (Bouby, 2014; Alonso and Rovira, 2016) and a dye plant, *Isatis tinctoria* (Alonso and Rovira, 2010). According to available data, evidence of new plants is limited to large urban port sites, strongly involved in commercial activities and harboring foreigners with their own food preferences. The new fruit species may have been introduced and locally cultivated or may have been imported. In Spain too, the adoption of new fruits occurred first on the coast, in relation to colonial activities, starting in the beginning of the 1st millennium BCE in Andalucia, where important Phoenician colonies were founded (Pérez-Jordà et al., 2021a) and spread progressively to the North-East (Buxó, 2008; Pérez-Jordà et al., 2021b).

It is difficult to know whether, and to what extent, the new plants penetrated the hinterland and the native society. Marseille and Lattara benefited from very favorable conditions for the recording of fruit taxa, with the preservation of waterlogged material in both sites and exceptionally extensive sampling at Lattara. It is likely that, in comparison, the record of allochtonous taxa is underestimated in native sites. In fact, if we consider pulses, which preserve well by charring, the only new species (*Cicer arietinum*) is present in several indigenous sites, especially in the vicinity of Marseille, where the species may have been introduced under Greek influence.

**FIGURE 8:**
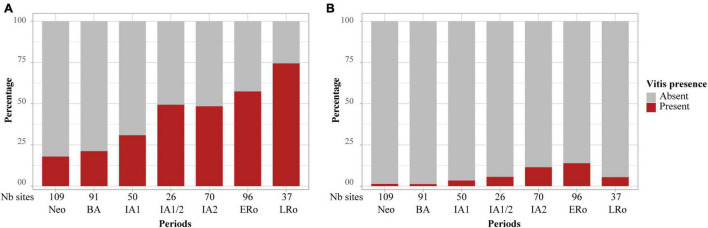
Occurrence of *Vitis* remains in the charred archeological record in relation to cultural periods, **(A)** pips, **(B)** pedicels. Bar plots showing the proportion of sites which delivered *Vitis* remains. The number of sites for each period is provided at the bottom of the graph.

At the same time as non-native fruit taxa are first recorded, the frequency and abundance of some INDCULT taxa increase in Mediterranean sites. This is particularly the case for *Vitis vinifera*, found in most sites of periods IA1 and IA/2. By the IA2, grape pips were as frequent and abundant in the Mediterranean sites as they will be during Roman times. Most significantly, grape pedicels, which were up to then quite rare and largely restricted to waterlogged assemblages, became common during the Iron Age (Figure 8). The record of pedicels beside grape pips is a good indicator of wine making residues, even if these can only be securely characterized by the presence of numerous grape pips associated with pedicels, pressed skins and possibly other bunch elements (Margaritis and Jones, 2006). Such assemblages were found at the Mediterranean IA2 sites of Île de Martigues, Coudouneu, and Le Castellan (Bouby et al., 2014). Evidence of wine making was recorded at Lattara, in the late 5th c. BCE, by matching results from archaeobotany (Py and Buxó i Capdevila, 2001; Alonso and Rovira, 2010; Rovira and Alonso, 2018) and chemical analysis of a wine press (McGovern et al., 2013).

Information on the spread of viticulture is also provided by Morphometric analyses of grape pips, which allow to securely discriminate wild and domesticated grapevines (Mangafa and Kotsakis, 1996; Bouby et al., 2013). The domesticated pip type was present and dominant in the Mediterranean area at least from the 5th c. BCE onward (Bouby et al., 2014). In short, the increase in the frequency of grape pips, the spread of the domesticated pip morphotype and of evidence of wine making suggest that the grapevine was regularly cultivated in the Mediterranean area during the Iron Age.

This activity clearly went beyond the vicinity of the colonial sites and penetrated the indigenous areas, particularly in the coastal plain. But how far did it actually go? In general, it is difficult to track changes in fruit uses in the temperate zone as less Iron Age sites were investigated. However, it is clear that pip remains were found much less frequently outside the mediterranean region. In addition, all the Iron Age pips from the temperate zone belong to the wild type (Bouby et al., 2014). This suggests that the domesticated grapevine and viticulture remained largely confined to areas strongly permeated by Mediterranean values. Nevertheless, evidence of grapevine cultivation was actually found at Alba-la-Romaine, about 150 km to the North from the Mediterranean (Cabanis et al., 2021). Pip fragments and charcoal of *Vitis vinifera* were identified in 5th-4th c. BCE archeological layers. Morpho-anatomical analyses of charcoal fragments suggest they belong to the cultivated grapevine (Limier et al., 2018).

**FIGURE 9:**
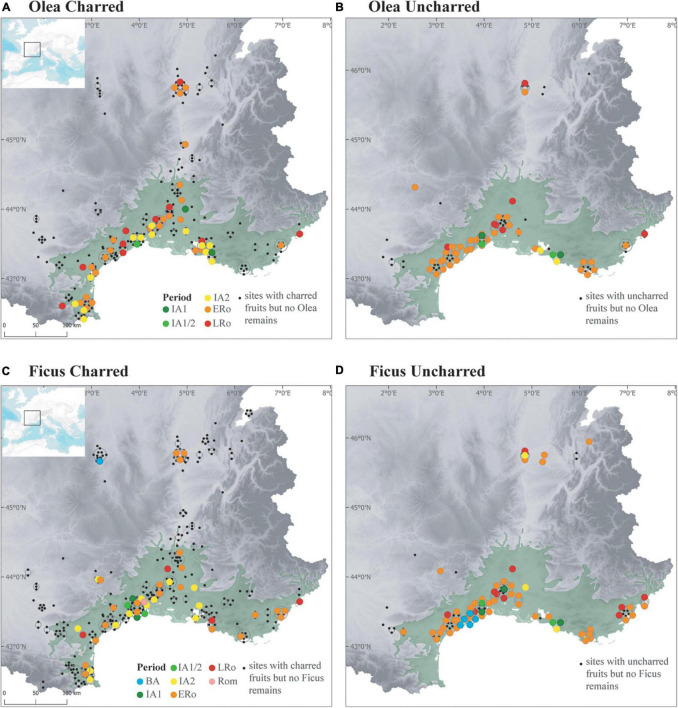
Maps showing the distribution of **(A,B)** *Olea* finds in Iron Age and Roman sites, **(C,D)** *Ficus* finds in Bronze Age-Iron Age and Roman sites, according to preservation type (**A,C**, Charred; **B,D,** Uncharred).

Other species, such as fig and olive trees were also most probably cultivated in the Mediterranean zone, at least during IA2, if we consider the increase in their frequency and abundance (Figures 5, 9). However, they were even more limited to the Mediterranean area than grapevine. All finds of olive stones occurred within the bioclimatic zone where the tree grows naturally. All sites are located close to the sea or in the littoral plain with the exception of Le Mourre de Sève, from the beginning of the 5th c. BCE (Pinaud-Querrac’h, 2015). Most sites, however, only date back to the last two centuries BCE, when the regional increase in the frequency of press facilities is interpreted as an indication of the development of olive oil production (Brun et al., 1998; Brun, 2004).

Occasional IA2 records of Mediterranean and allochtonous fruits in the temperate zone are difficult to interpret. Numerous nutshell fragments of *Juglans regia* were recovered in the Late IA2 layers of La Grande Rivoire (Isère; 100-0 BCE) (Martin, unpublished). Few fig achenes were identified at Puech de Mus (Aveyron; 400-300 BCE) (Durand, unpublished) and Parc Saint Georges (Rhône; 200-75 BCE) (Bouby, 2013). In addition, a much earlier fig achene was found in the Late Bronze Age site of Corent (Puy-de-Dôme; 1130-900 BCE) (Milcent et al., in press), in the north of the Massif Central. The chronology of the macrorest is confirmed by direct radiocarbon dating. This is also needed to confirm the IA2 finds. Nevertheless, if we accept their age, two main hypotheses can be considered to explain these scattered early finds of non-native fruits: (1) The trees were introduced and planted around the sites or (2) dried fruit was transported from the Mediterranean region, where the fig tree is part of the spontaneous vegetation and *Juglans regia*, although allochtonous, was probably already introduced. Lastly, we cannot rule out the spontaneous presence of thermophilous taxa such as the fig tree outside the Mediterranean zone, at specific spots under favorable conditions; nowadays, mediterranean trees (*Quercus ilex*, *Pistacia terebinthus*) grow sporadically along the Rhône valley, south of Lyon.

### A New Step in Roman Times

The economic and cultural contribution of cultivated fruit taxa increased in Roman times. We have reported that for the olive tree, this intensification is already visible during the last two centuries BCE, with the beginning of Romanization in the Mediterranean area. The expansion of viticulture may even have started earlier in the 3rd c. BCE (Py and Buxó i Capdevila, 2001) as suggested by the vineyards uncovered in the periphery of cities such as Marseille (Boissinot, 2001; Bouiron, 2005), Lattara (Jung, 2007), and Nîmes (Pomarèdes et al., 2009).

But a new step in the spread of non-native fruits and cultivated trees was undoubtedly achieved by the late 1st c. BCE, with the expansion of the Roman Empire. The first dates (*Phoenix dactylifera*) were recorded close to the Mediterranean Sea, in Capelles, near the large city of Narbonne (Tillier, 2019), and in the rural site of La Lesse, in the proximity of the urban center of Béziers (Figueiral et al., 2015). Dates were exotic luxury fruits, imported from the southern shores of the Mediterranean or the Near East. Also, Cypress (*Cupressus sempervirens*) remains dating from the early Empire were identified in several sites near the coast. At La Nautique and Capelles, the association of *Cupressus* seeds, cones and leaves show that this tree was planted locally (Tillier, 2019). The cypress tree had culinary, ornamental and symbolic uses in the Roman world (Feemster Jashemski and Meyer, 2002). Thus, *Cupressus* was planted at an early stage in the northern Mediterranean to decorate the Roman cities and neighboring rural settlements.

Other fruits were first recorded around the beginning of the Roman Empire, including another exotic species (*Cordia myxa*) and various allochtonous trees, which could have been acclimatized in Southern France: *Mespilus germanica*, *Morus alba/nigra*, *Prunus cerasus*, *P. persica*, *Ziziphus* cf. *ziziphus*. Concerning *Celtis australis* its significance remains unclear. This species is considered as native to Southern France (Quézel and Médail, 2003). However, while *Celtis* is recorded by paleobotany up to the Middle Pleistocene (Saporta, 1867; Boone and Renault-Miskowsky, 1976), it appears absent from Holocene archeological sites until the Roman period. By then, *Celtis* fruits, leaves and charcoal appear in several urban and rural sites from the 1st c. CE (Rovira, 2012; Bouchette et al., 2017; Figueiral et al., 2017). The species may have persisted after the Pleistocene, remaining undetected by archaeobotany, or may have been reintroduced by the Romans. In any case, it was cultivated and spread in Roman times, particularly as an ornamental tree, as its recurrent record in urban contexts seems to indicate. Ornamental trees such as *Celtis* and *Cupressus* provide a good example of the key role that cities continued to play in the adoption of new plants in Roman times.

The CFAs (Figure 5 and Supplementary Figure 1) shows that Roman rural and urban sites differed in their fruit record, in the Mediterranean and temperate zones, even if taphonomic factors could reinforce this differentiation. This difference probably resulted from the greater connectivity of urban centers with the Mediterranean networks, favoring a quicker adoption of new taxa in the cities. On the other hand, the southern rural sites are characterized by the importance of grapevine, which is consistent with the exceptional development of viticulture in the region, as shown by the large number of wine-producer sites and wine amphora ateliers (Buffat et al., 2001; Brun, 2010). Furthermore, the traces of vineyards found in Southern France were mostly assigned to the ERo period (Boissinot, 2001; Pomarèdes et al., 2012; Jung et al., 2013). In addition to grapes, the expanding role of fruits in the economy and rural landscape of the Mediterranean region during the ERo can be recognized in the more frequent appearance of *Ficus* and *Olea* remains (Figure 9). Orchards are identified for the first time, even if their traces cannot be associated with a specific fruit tree (Jung et al., 2013).

The diffusion and adoption of new fruits were largely fueled by the cultural values conveyed by Romanization. This is evident in the differentiation between rural and funerary/ceremonial sites, sustained by the greater importance of allochthonous and cultivated fruits in the last sites. They include *Juglans regia*, *Phoenix dactylifera*, and *Pinus pinea*, which had strong symbolic and religious values in the Roman world and that were also recorded in funerary and ceremonial contexts in Italy (Rottoli and Castiglioni, 2011) and in many other regions of the Empire (Zach, 2002; Preiss et al., 2005; Livarda, 2013; Reed et al., 2018). In the Western Mediterranean region, some of the early records of dates are linked to ritual foundation deposits (Rovira and Chabal, 2008; Figueiral et al., 2015) but are in general mostly linked to grave offerings (Marinval, 1993, 2004).

**FIGURE 10:**
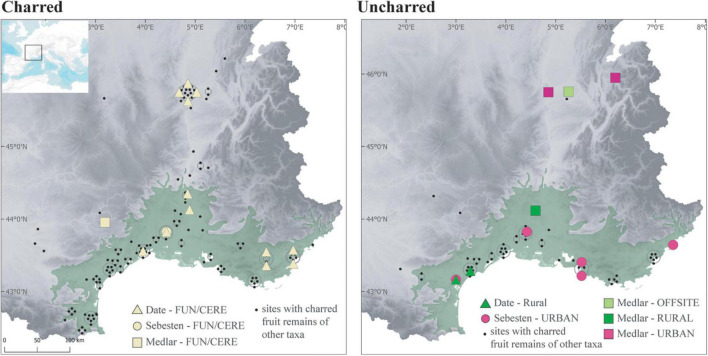
Maps showing the distribution of the records of date (*Phoenix dactylifera*), sebesten (*Cordia myxa*) and medlar (*Mespilus germanica*) in Roman sites according to preservation and site types.

The spread of Roman culture was promoted directly *via* human migration, in particular with the establishment of colonies of Roman veterans in most of South-Eastern France, as well as through the unprecedented strengthening of a Mediterranean-wide economic and cultural connectivity. The hypothesis of a connection with the presence of immigrants has been raised for *Cordia myxa* (Bouby et al., 2011a), an exotic species traditionally used in the South-Eastern Mediterranean, and recorded in several large cities in Southern France, including harbors, and in a funeral context (Figure 10). The population of these cities is known to have included people of eastern origins (Jewish, Greeks, Egyptians), especially merchants.

The establishment of the Roman Empire clearly helped to spread Mediterranean and non-native fruits outside of the Mediterranean area. The most characteristic fruits are also identified in funerary contexts from the temperate zone, at least in those of Lyon (Marinval, 2004) where the largest number of burials was excavated (Figure 10). Findings of *Olea* and *Ficus* are more frequent in the temperate zone during the Roman period than during the IA2 (Figure 9). Some of these fruits were imported, especially olives, which could not be grown as far north. But some species appear to have been introduced as suggested by offsite agrarian records, which primarily reflect local vegetation (Jackson et al., 1997). The palaeochanel of Les Cariaux and the rural ditches of Saint-Romain-de-Jalionas (Isère) included several allochtonous cultivated fruit species, such as *Ficus carica*, *Lagenaria siceraria*, and *Prunus persica*, together with other taxa that represent the local vegetation (Bouby, 2014). We believe that these non-native fruits were cultivated locally in the ERo. Today, fig trees still occasionnally grow in the area. However, the northward spread of allochthonous taxa was not sufficient to homogenize the Roman fruit record. Obviously, thermophilous fruits remained more common in the South. In addition, the temperate zone was differentiated by indigenous wild fruits but also by new cultivated plants, especially *Mespilus germanica*, an acidophilus mesophilous non-native species well adapted to the soil and conditions of the middle and upper Rhône basin (Figure 10).

The CFAs suggest a small decline in cultivated and allochthonous fruits in the LRo. This trend is in line with a similar change observed in annual crops. The dependence on naked wheat and barley, typical of the ERo, slightly decreased during the LRo in favor of greater crop diversity (Zech-Matterne and Bouby, 2020). These changes are consistent with the hypothesis of a crisis of the speculative agricultural system during the LRo with a return to a more diversified agriculture and traditional productions (Ouzoulias et al., 2001; Brun, 2010).

### Changes in Fruit Taxa, Changes in Management Practices: Traditions and Innovations

The important changes in fruit exploitation that occurred from the 6th-5th c. BCE onward were concomitant with changes in cultivation practices and a mutation of agrarian systems. These are particularly obvious for specialized, speculative productions, which involve specific tools and structures as well as massive facilities that can be detected and recognized by archeology. In our area, such specialized production was above all represented by viticulture. At least by the middle of the 6th c. BCE, Marseille developed a specialized viticulture, as shown by the production of particular wine amphorae that allowed to massively supply the indigenous market in southern France (Bats, 1990; Bertucchi, 1992). Traces of vineyards are reported in Marseille from the 4th-3rd c. BCE (Boissinot, 2001; Bouiron, 2005) but probably existed before. Standardized plantations such as vineyards undoubtedly reflected the use and spread of a whole range of arboricultural techniques, including plantation, fertilization, pruning and vegetative propagation of selected phenotypes. The vineyards identified in France regularly show secondary pits, related to the original pits, evidence of a reproduction technique (‘provignage’) consisting of laying down a branch from an existing plant to produce a new one (Boissinot, 2001).

The exceptional development of specialized viticulture during the ERo period emphasizes the spread of these arboricultural techniques. By then, vineyard traces are also sporadically identified in temperate France (Poux et al., 2011). Viticulture aside, evidence of specialized arboriculture is limited to speculative oil production, which possibly started at the end of the Iron Age but remained clearly secondary (Brun, 2010), and to rare traces of orchards (Jung et al., 2013; Boissinot et al., 2016). However, the increasing number of finds of fruit remains suggests that cultivation of fruit trees was more widespread than the sole archeological traces can indicate. Naturally, some of these finds can be the result of importation. The records of exotic fruits or olives are undeniable examples (Tillier, 2019). Nevertheless, the identification of fruit trees by pollen and wood charcoal analyses provides congruent evidence of local arboriculture for plants that can be identified using these techniques. Pollen indicators of cultivated fruit trees increased in Southern France from about 500 BCE onward (Berger et al., 2019). Charcoal remains of *Ficus carica*, *Juglans regia*, Maloideae, *Olea europaea*, *Pinus pinea*, *Prunus* sp. are recorded more frequently from the Iron Age onward (Chabal, 1997; Figueiral et al., 2010a).

Fruit trees can be cultivated according to a variety of low-intensity and unspecialized techniques, which leave hardly any trace identifiable by archeology. No doubt, such forms of arboriculture were employed alongside specialized plantations in ancient times. For that matter, with the exception of vineyards, specialized orchards only developed recently in southern France (last two centuries). Before that, fruit trees were most often scattered in gardens, vineyards, fields and permanent pastures, in hedgerows and along paths (Leterme, 2018). Similar situations are still common in many agrosystems when fruit production is not primarily oriented toward the market (Wiersum, 1997; Bouby and Ruas, 2014).

In the Iron Age, vineyards were found close to cities but domesticated pips, charcoals and wine making residues were identified in smaller settlements where no vineyards have been spotted up to now. In these cases, a less intensive form of viticulture, with scattered vines, can be hypothesized (Bouby et al., 2014). For the Roman period, archaeobotanical evidence from Le Gasquinoy (Hérault; Figueiral et al., 2010b) and Fréjus (Var; Bouby et al., 2011b) suggests that fruit trees were cultivated in farms dedicated to viticulture as well as in periurban horticultural areas. In some of the Roman vineyards uncovered, the presence of larger than usual pits suggests the occasional plantation of large trees associated with vines (Boissinot et al., 2016).

We may wonder whether the beginning of specialized viticulture in the 6th century BCE marked the start of any form of arboriculture or rather a phase of intensification. In other words, does the management of fruit trees precede the introduction of allochthonous fruits?

The archaeobotanical record also shows important continuities. Some of the most important cultivated plants in the Iron Age and Roman period were regularly exploited beforehand (*Ficus carica*, *Vitis vinifera*). Many wild taxa have been used continuously (e.g., *Prunus spinosa*, *Quercus* sp., *Rubus* sp., *Sambucus* sp.). During Roman times they are found in both rural and urban settings and occasionally in burials (Marinval, 2004). Perhaps, these wild fruits should not be considered as occasional food, but as having a cultural and nutritional value of their own, which would explain why they were looked for and consumed in cities and were considered suitable to accompany the dead in the afterlife. When we consider the recurrence of wild fruits in large cities such as Marseille, Nîmes or Toulon, it seems unlikely that they were simply gathered in the surroundings. Some could have been harvested from favored, managed plants or obtained by trade. A similar observation was made concerning the Middle Ages (Ruas, 1996). In the medieval texts from southern France (12th-14th c. CE) the market price of acorns is mentioned, which proves that these were regularly sold (Ruas et al., 2006; Ros et al., 2020). In Roman times, Pliny the Elder explained how to sow and transplant brambles and elderberries to form a hedge (Hist Nat, L XVII, 11). According to André (2009) elderberries (*Sambucus* sp.) could be boiled in water or wine to make a marmalade, which would make them easier to preserve and trade. Brambles (*Rubus* sp.) and elderberries are among the most common wild fruit taxa in Roman archaeobotanical assemblages.

Nowadays, in unmechanized agricultural systems, many wild fruits are still sold in local markets, wild fruit trees being protected, managed or even transplanted, especially in home-gardens (e.g., Vavilov, 1930; Otero-Arnaiz et al., 2005; Dawson et al., 2020). In fact, domestication of fruits should be considered a continuous process from wild, to managed populations and domesticated clonal varieties in the end. Management practices involve, in ascending order, elimination of competitors, protection of useful-plants, thinning/pruning, improvement of soil properties, transport of useful-plants, favoring of desired phenotypes, selection and cloning (e.g., Wiersum, 2008; Bouby and Ruas, 2014; Furlan et al., 2017; Levis et al., 2018). Domestication practices increase when resources are threatened, especially by deforestation, or when demand grows.

We believe that low intensity management practices of fruit plants may have been implemented in our area before the arrival of allochthonous species and specialized arboriculture but this is difficult to demonstrate. When acorns had a very significant economic role in the Mediterranean hinterland in the Late Neolithic, or on a larger scale in the Bronze Age, it is reasonable to assume that the most valuable oak trees were favored or at least protected (Marinval, 2008). This seems all the more likely as the Late Neolithic (about 2,500 BCE) and even more so the Late Bronze Age are periods of demographic growth associated with increasing deforestation (Berger et al., 2019). Acorns contain tannins that have an astringent taste and can be toxic to humans and domestic animals when they eat a lot of them. After harvesting, different processes can be used to eliminate theses tannins (e.g., Mason, 1995). However, the proportion of tannins varies according to various parameters and especially from tree to tree. It would therefore be particularly advantageous to favor trees with sweeter acorns.

However, in our study area, clear evidence that fruit plants were transported and planted before the beginning of specialized arboriculture is scarce. We have mentioned a few records of fig remains outside their native Mediterranean area in the Late Bronze Age and toward the end of the Second Iron Age, which could mean a diffusion of the tree or a simple transport of the fruit. In south-eastern Spain, the large number of figs in archaeobotanical samples, combined with the presence of charcoal, suggests that the fig tree was already cultivated in the Bronze Age of El Argar (2,200-1,500 BC) (Stika, 1988). Figs, together with grapes and olives, may even have been transported from producer to consumer areas within the El Argar territory (Celma Martínez and Stika, 2020).

**FIGURE 11:**
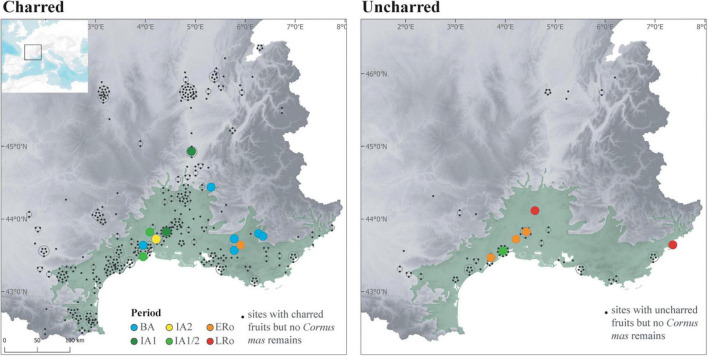
Maps showing the distribution of the records of cornelian cherry (*Cornus mas*) in the sites according to archeological periods and preservation type.

Also, cornelian cherry (*Cornus mas*) could have been favored and spread by man (Bouby, 2014). Nowadays, this small tree thrives in open forests on calcareous soils in Eastern and South-Western France, where it is regarded as indigenous (Rameau et al., 1989; Da Ronch et al., 2016). And yet, *Cornus mas* appears unreported by archaeobotany in France before the Roman period, except in our study area, where repeated mentions exist since the Bronze Age, more particularly in the south-eastern part, south of the Alps (Figure 11). Most remarkably, concentrations of stones of *Cornus mas* were often found in Northern Italy since the Early Neolithic. The species is consequently considered as massively exploited, and probably managed, as early as the Late Neolithic and throughout the Bronze Age, possibly for the preparation of a fermented beverage (Castelletti et al., 2001; Fiorentino et al., 2004; Rottoli and Pessina, 2007). Bronze Age records in southeastern France may then constitute the most westerly point of a vast area of exploitation of *Cornus mas*. For the moment, the earliest records are clustered to the east and seem to extend westwards in the Iron Age. However, it is impossible to assess whether the Bronze and Iron Age use of *Cornus mas* was based on spontaneous stands or whether man actually spread this species. Unlike many fruit species, *Cornus mas* is easily propagated by seeds, making it unnecessary to master vegetative propagation techniques.

## Conclusion

Archaeobotanical fruit and seed records are relevant to investigate changes in fruit exploitation in Southern France from the Neolithic to the end of the Roman Empire in Western Europe, despite taphonomic, methodological and contextual drawbacks. They clearly reflect changes and continuities in the diversity of species used through time and the correspondence factor analyses of charred and uncharred seed and fruit records conveyed consistent spatio-temporal patterns.

Significant changes are documented in relation with the arrival of allochthonous fruits and the development of cultivated native species. Two major steps are recognized, (1) during the Iron Age (6th-5th c. BCE) in the Mediterranean zone only, and (2) at the beginning of the Roman Empire (late 1st c BCE-early 1st c CE). This pattern is consistent with the rise and intensification of colonial activities and their impact on the introduction of allochthonous fruits and the development of arboriculture. The archaeobotanical data particularly reflect the prevailing importance of viticulture, in line with archeological evidence.

This work also reveals differences throughout the chronology in the fruit taxa exploited in the Mediterranean and in temperate regions. This is due to the variability of the fruit resources available nearby but also to the extension of better-adapted cultivated species, or even to cultural preferences. A larger scale survey would certainly allow a better assessment of spatial variability in fruit preferences and its determinants. During the Roman Imperial period, certain new arrivals to Southern France (*Cordia myxa*, *Morus alba/nigra*, *Ziziphus* sp.) seem to have spread little or not at all to central and western Europe, even in strongly romanized areas (Bakels and Jacomet, 2003; Livarda, 2011). Does this reflect regional specificities or a global Mediterranean pattern?

Our study highlights the major role of cities in the introduction and adoption of new fruit species, already in the Iron Age and still during the Roman period. Data from funerary and ritual contexts also illustrate the importance of cultural and symbolic values in the process.

Traditional archeology only identifies specialized arboriculture (grapevines and olive trees) but archaeobotany points to a much broader spectrum suggesting different low intensity management and cultivation practices occurring in addition to specialized cultivation. Most significantly, our work acknowledges the constant dietary importance of native, supposedly wild, fruits throughout the chronology, even after the full development of commercial arboriculture and the arrival of domestic taxa, and this even in the largest Roman cities. This reminds us that fruit exploitation practices are complex and should not be reduced to a simple opposition between gathering wild plants in the forest and implementing monocultural systems based on selected varieties. Alternative management practices existed and most probably predated the development of viticulture in the Iron Age.

The question remains as to whether the region contributed to the domestication process of the native species or if the domesticated forms were entirely introduced together with specialized cultivation practices. In the case of grapevine and olive, current molecular approaches reveal recurrent introgression events between cultivated and wild populations that blur the perception of possible earlier domestication in the western Mediterranean (Besnard et al., 2018; Grassi and De Lorenzis, 2021). The advent of studies in geometric morphometry and palaeogenomics allows us to foresee future progress on these matters (Ramos-Madrigal et al., 2019).

## Data Availability Statement

The raw data supporting the conclusions of this article is available in the Supplementary Material section.

## Author Contributions

LB: conceptualization, project administration, and writing – original draft. LB, MC, FD, IF, LF, PM, LM, LP, RP, JR, NR, and MT: data curation. LB and VB: formal analysis and methodology. LB, MC, FD, IF, LF, PM, LM, RP, JR, NR, and MT: resources. LB, LP, and VB: visualization. All authors: writing – review and editing.

## Conflict of Interest

The authors declare that the research was conducted in the absence of any commercial or financial relationships that could be construed as a potential conflict of interest.

## Publisher’s Note

All claims expressed in this article are solely those of the authors and do not necessarily represent those of their affiliated organizations, or those of the publisher, the editors and the reviewers. Any product that may be evaluated in this article, or claim that may be made by its manufacturer, is not guaranteed or endorsed by the publisher.
