# Supplementary material for: Talkin’ About a Revolution. Changes and Continuities in Fruit Use in Southern France From Neolithic to Roman Times Using Archaeobotanical Data (ca. 5,800 BCE – 500 CE)
Source: Front Plant Sci. 2022 Feb 7;13:719406. doi: 10.3389/fpls.2022.719406 (PMC8859487; doi:10.3389/fpls.2022.719406)

Supplementary Figure 1. CFA on uncharred fruit remains. First biplot of the correspondance factor analysis on log-transformed raw counts of uncharred fruit remains, (A) Plot of taxa, (B) Plot of sites according to main archaeological periods, (C) Plot of sites according to bioclimatic zones. Box plots showing the distribution of the sites on (D) axis 1 and (E) axis 2 of the CFA in relation to cultural periods and bioclimatic zones. Axis 1 displays a discrimination between cultivated and thermophilous taxa to the left and native and mesophilous wild fruits to the right. Axis 2 displays a discrimination mostly between fruits with pips in the lower part and stone and nut fruits in the upper part. The number of sites for each period and bioclimatic zone is provided at the bottom of the graph.

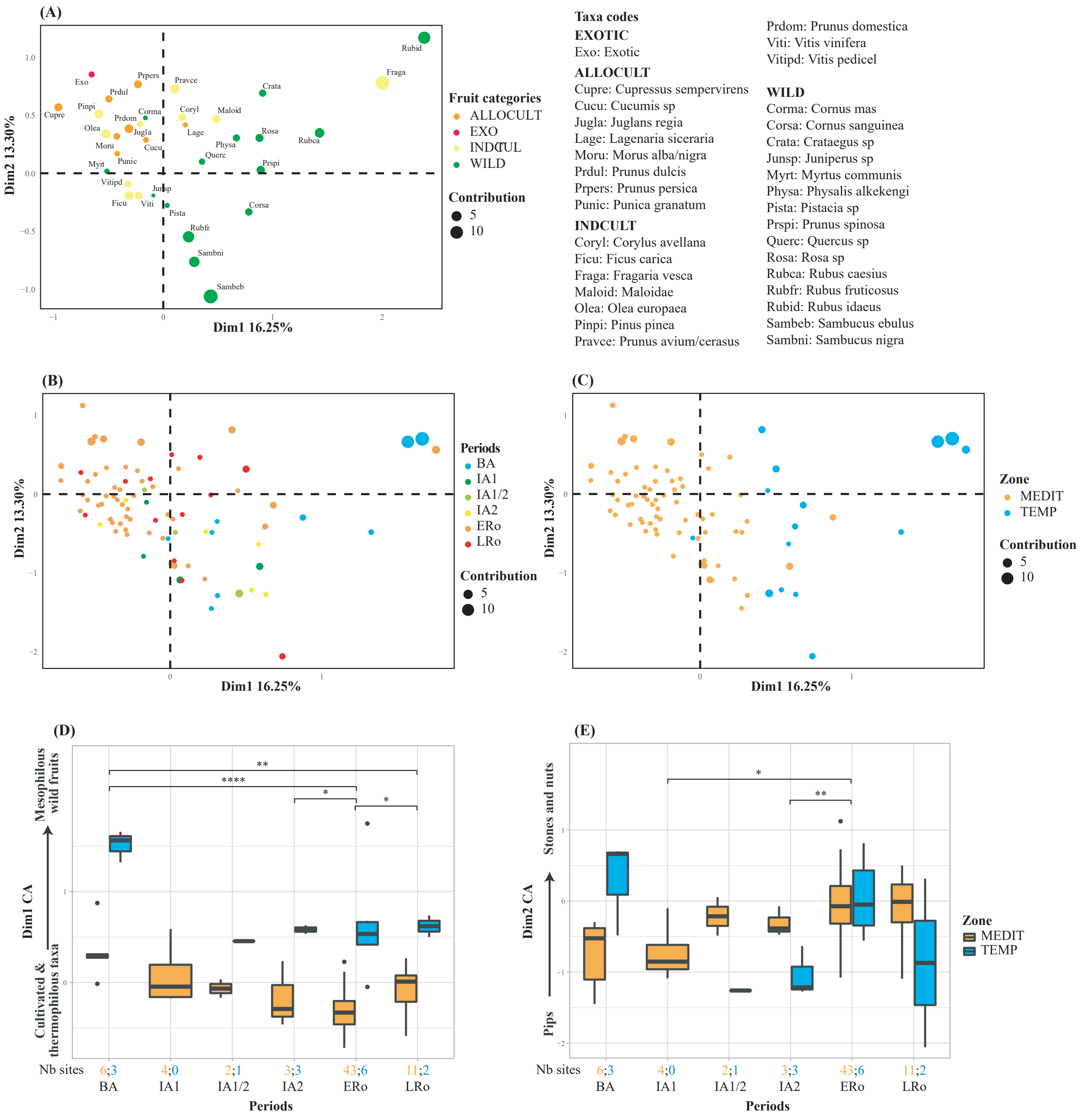

Supplement: Supplementary file 1 [file Image_1.pdf]
