# Supplementary material for: Talkin’ About a Revolution. Changes and Continuities in Fruit Use in Southern France From Neolithic to Roman Times Using Archaeobotanical Data (ca. 5,800 BCE – 500 CE)
Source: Front Plant Sci. 2022 Feb 7;13:719406. doi: 10.3389/fpls.2022.719406 (PMC8859487; doi:10.3389/fpls.2022.719406)

Supplementary Figure 2. Maps showing the distribution of the sites according to their period and their coordinate on the first axis of the CFA carried out on the data from charred fruit remains.

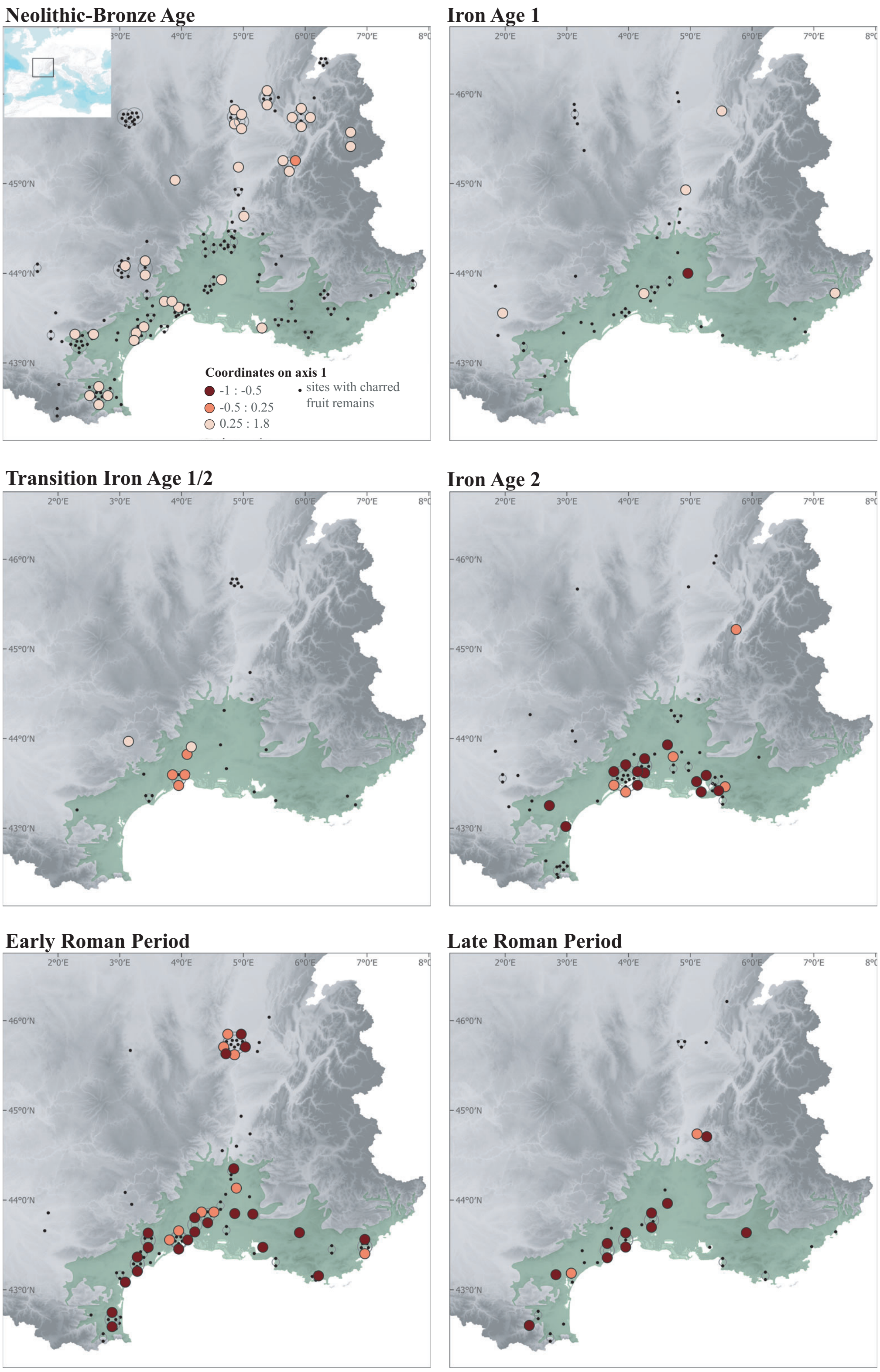

Supplement: Supplementary file 2 [file Image_2.pdf]
