# Supplementary material for: Talkin’ About a Revolution. Changes and Continuities in Fruit Use in Southern France From Neolithic to Roman Times Using Archaeobotanical Data (ca. 5,800 BCE – 500 CE)
Source: Front Plant Sci. 2022 Feb 7;13:719406. doi: 10.3389/fpls.2022.719406 (PMC8859487; doi:10.3389/fpls.2022.719406)

Supplementary Figure 3. CFA on Roman charred fruit remains. First biplot of the correspondance factor analysis performed on log-transformed raw counts of charred fruit remains in the Roman sites only, (A) Plot of the taxa, (B) Plot of the sites according to main archaeological periods, (C) Plot of the sites according to bioclimatic zones.

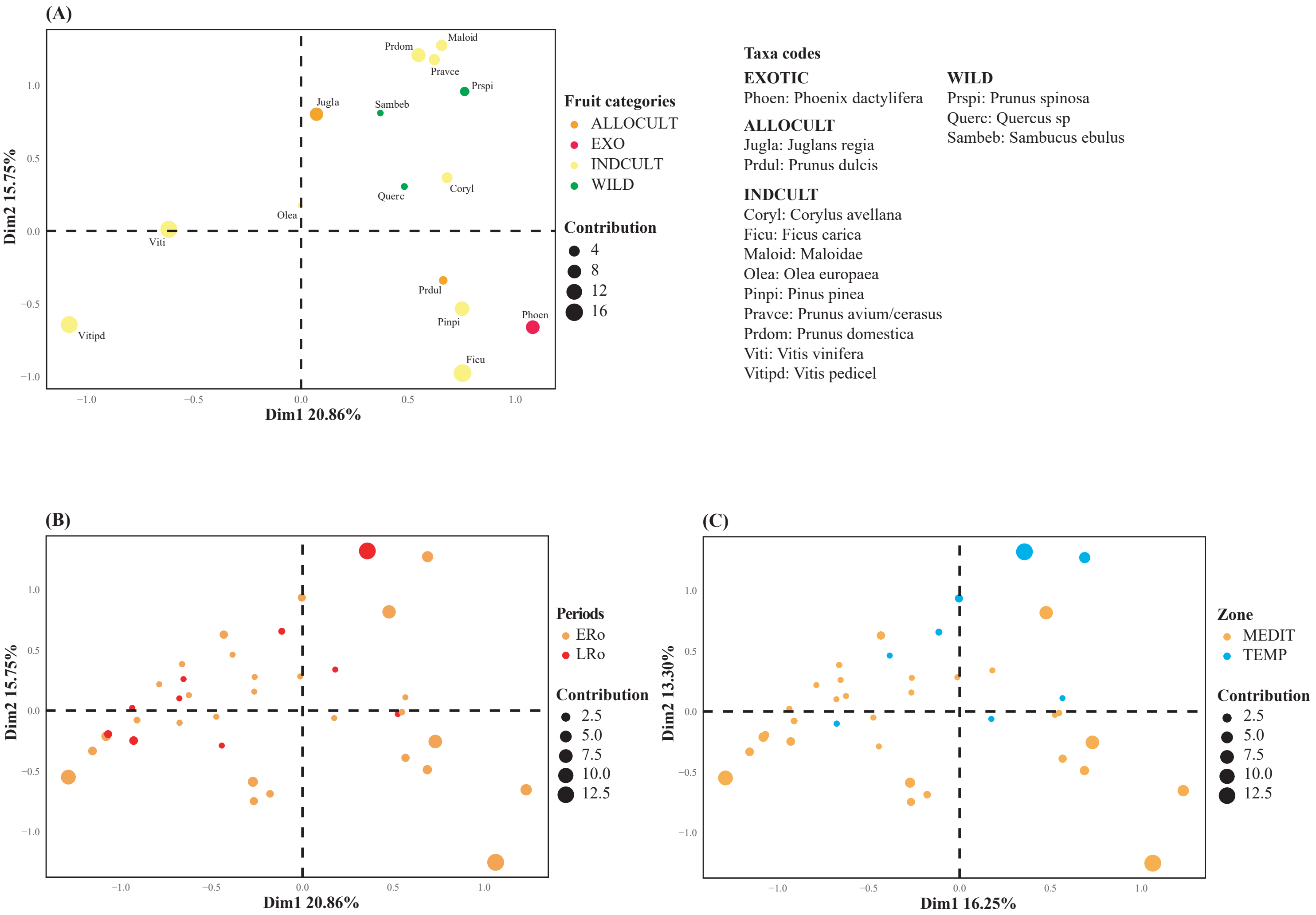

Supplement: Supplementary file 3 [file Image_3.pdf]
