# Supplementary material for: Talkin’ About a Revolution. Changes and Continuities in Fruit Use in Southern France From Neolithic to Roman Times Using Archaeobotanical Data (ca. 5,800 BCE – 500 CE)
Source: Front Plant Sci. 2022 Feb 7;13:719406. doi: 10.3389/fpls.2022.719406 (PMC8859487; doi:10.3389/fpls.2022.719406)

Supplementary Figure 4. CFA on Roman uncharred fruit remains. First biplot of the correspondance factor analysis performed on log-transformed raw counts of uncharred fruit remains in the Roman sites only, (A) Plot of the taxa, (B) Plot of the sites according to main archaeological periods, (C) Plot of the sites according to bioclimatic zones, (D) Plot of the sites according to site types.

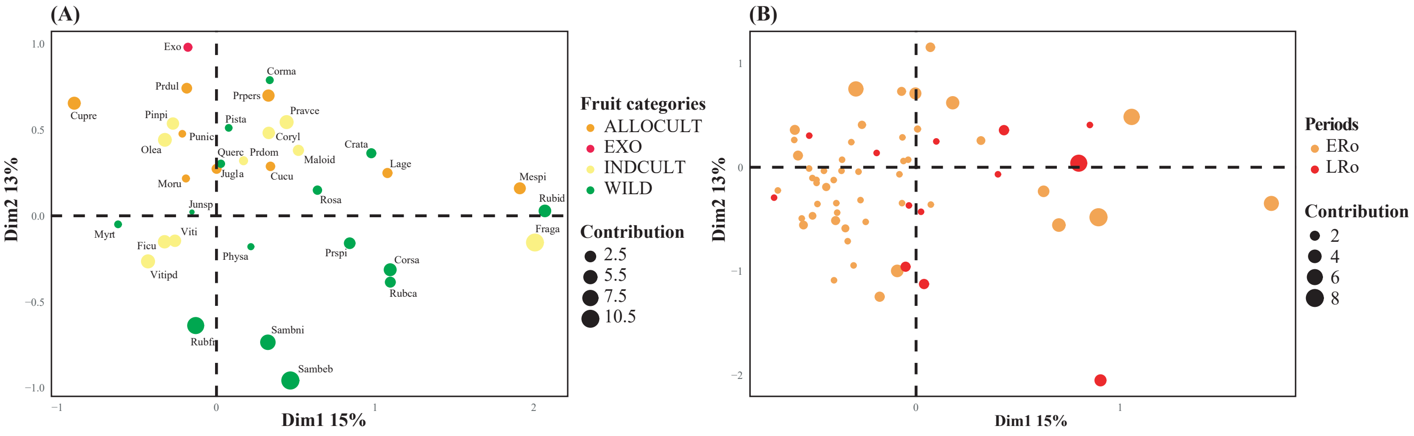

Taxa codes

EXOTIC
Exo: Exotic

ALLOCULT
Cupre: Cupressus sempervirens
Cucu: Cucumis sp
Jugla: Juglans regia
Lage: Lagenaria siceraria
Mespi: Mespilus germanica
Moru: Morus alba/nigra
Prdul: Prunus dulcis
Prpers: Prunus persica
Punic: Punica granatum

INDCULT
Coryl: Corylus avellana
Ficu: Ficus carica
Fraga: Fragaria vesca
Maloid: Maloidae
Olea: Olea europaea
Pinpi: Pinus pinea
Pravce: Prunus avium/cerasus
Prdom: Prunus domestica
Viti: Vitis vinifera
Vitipd: Vitis pedicel

WILD
Corma: Cornus mas
Corsa: Cornus sanguinea
Crata: Crataegus sp
Junsp: Juniperus sp
Myrt: Myrtus communis
Physa: Physalis alkekengi
Pista: Pistacia sp
Prspi: Prunus spinosa
Querc: Quercus sp
Rosa: Rosa sp
Rubca: Rubus caesius
Rubfr: Rubus fruticosus
Rubid: Rubus idaeus
Sambeb: Sambucus ebulus
Sambni: Sambucus nigra

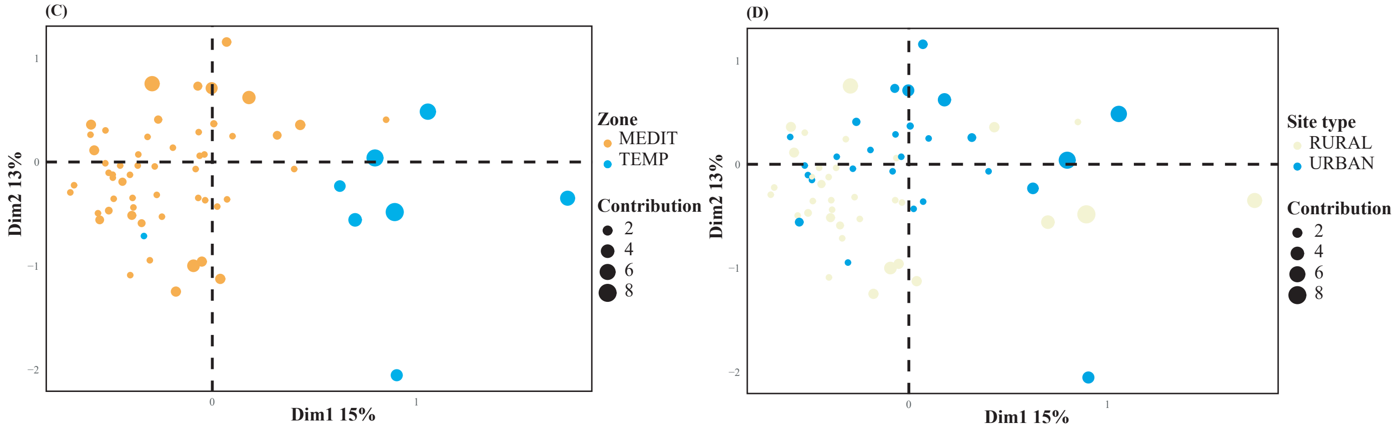

Supplement: Supplementary file 4 [file Image_4.pdf]
