# Supplementary material for: Talkin’ About a Revolution. Changes and Continuities in Fruit Use in Southern France From Neolithic to Roman Times Using Archaeobotanical Data (ca. 5,800 BCE – 500 CE)
Source: Front Plant Sci. 2022 Feb 7;13:719406. doi: 10.3389/fpls.2022.719406 (PMC8859487; doi:10.3389/fpls.2022.719406)

Supplementary Figure 5. Comparison of Roman sites according to (A) Roman periods and (B) site types. Only Rural and Urban sites are taken into consideration. Box plots showing the distribution of sites on axis 1 and 2 of the CFA performed on uncharred fruit remains in Roman sites only. Statistical significances of the Wilcoxon tests are indicated on the box plots, only when the result is significant (*:p<=0.05; **:p<=0.01;***:p<=0.001). The number of sites for each period, site category and bioclimatic zone is provided at the bottom of the graph.

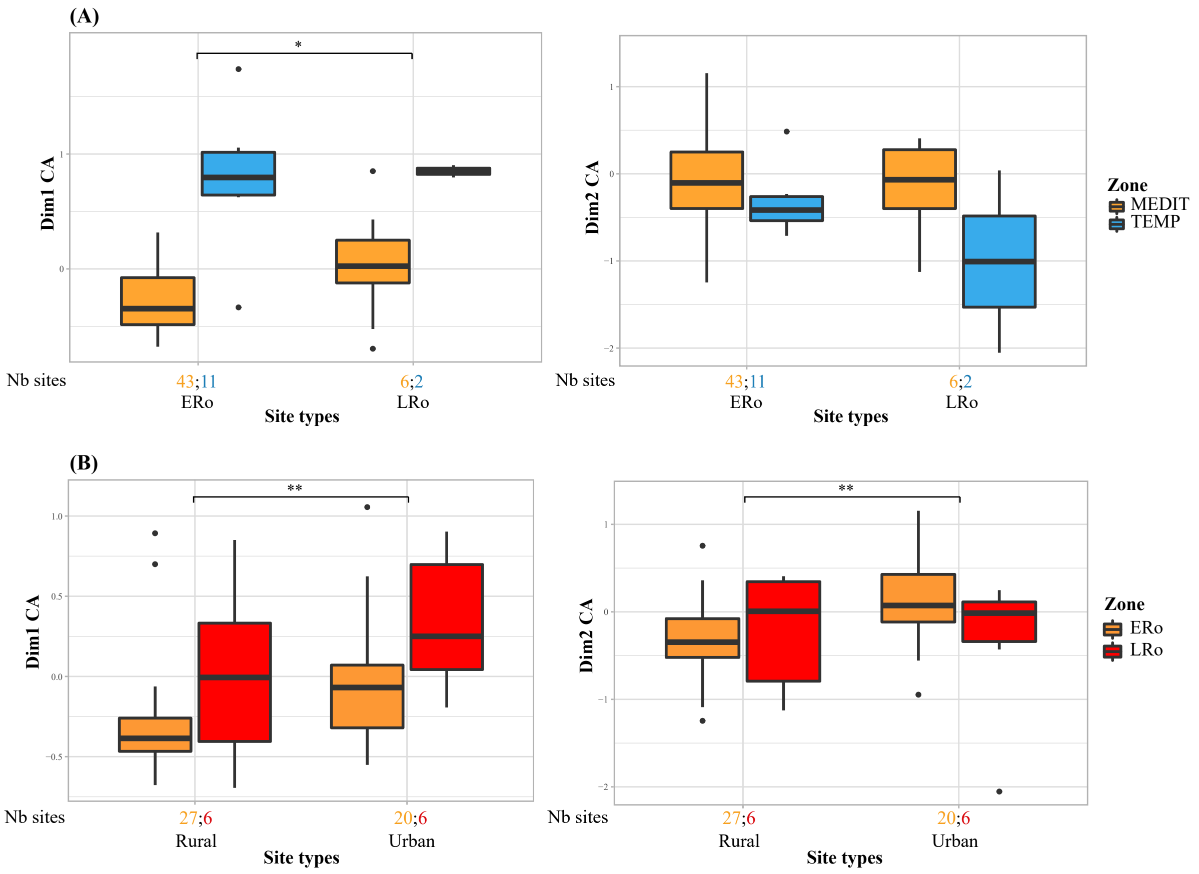

Supplement: Supplementary file 5 [file Image_5.pdf]
