# Supplementary material for: Talkin’ About a Revolution. Changes and Continuities in Fruit Use in Southern France From Neolithic to Roman Times Using Archaeobotanical Data (ca. 5,800 BCE – 500 CE)
Source: Front Plant Sci. 2022 Feb 7;13:719406. doi: 10.3389/fpls.2022.719406 (PMC8859487; doi:10.3389/fpls.2022.719406)
Supplement: Supplementary file 6 [file Data_Sheet_1.PDF]

Site	Municipality	Dep.	CodeSite	Bioclim	latitude	longitude	type	Period	DATE BP	Reference
Saint Etienne (Abri)	Saint-Etienne-de-Gourgas	34	AbStE	TEMP	43.77	3.38	CAVE	BA	3200 - 2900	Erroux 1981
Motte (La)	Agde	34	AgMot	MEDIT	43.32	3.47	RURAL	BA	2800 - 2700	Bouby et al 2016
Alba la Romaine	Alba-la-Romaine	07	AlRom	TEMP	44.55	4.59	FUN	ERo	1900 - 1800	Marinval 1993a
Ilet	Annecy-le-Vieux	74	AnIlet	TEMP	45.92	6.15	URBAN	ERo	1900 - 1700	Lundström-Baudais (1991)
Mas (Le)	Ansignan	66	AnsMa	MEDIT	42.77	2.52	RURAL	Rom	2025 - 1500	Ros unpubl
Pendimoum (Abri)	Castellar	06	APend	MEDIT	43.80	7.50	CAVE	Neo	7750 - 7000	Binder et al 1993, 2020
Conesa (Place)	Agde	34	APlCo	MEDIT	43.32	3.47	URBAN	IA1/2	2525 - 2400	Bouby 2014
Conesa (Place)	Agde	34	APlCo	MEDIT	43.32	3.47	URBAN	IA2	2400 - 2300	Bouby 2014
Boussargues	Argelliers	34	ArgBou	MEDIT	43.70	3.68	RURAL	Neo	4700 - 4200	Marinval 2008
Arles-Rhône 3	Arles	13	ArlR3	MEDIT	43.67	4.63	URBAN	ERo	2000 - 1750	Tillier 2019
Arles-Rhône 5	Arles	13	ArlR5	MEDIT	43.67	4.63	URBAN	ERo	1900 - 1850	Tillier 2019
Artière Ronzière	Beaumont	63	ArRz	TEMP	45.75	3.10	RURAL	Neo	6000 - 5500	Cabanis, Bouby 2016
Aspre del Paradis	Corneilla-del-Vercol	66	AspPar	MEDIT	42.63	2.95	RURAL	Neo	7000 - 6500	Manen et al 2001
Aspra del Paradis	Corneilla-del-Vercol	66	AspPar	MEDIT	42.63	2.95	RURAL	LRo	1625 - 1550	Ros and Ruas, 2017
Soumaltre	Aspiran	34	AsSou	MEDIT	43.57	3.43	RURAL	ERo	2000 - 1800	Zwierzinsky, Ruas 2004
Soumaltre - funerary	Aspiran	34	AsSouF	MEDIT	43.57	3.43	FUN	ERo	2000 - 1800	Zwierzinsky, Ruas 2004
Aumignanes	Saint-Victor-la-Coste	30	Aumig	MEDIT	44.07	4.63	RURAL	ERo	1800 - 1750	Bouby 2014
Auriac	Carcassonne	11	Auria	MEDIT	43.22	2.35	RURAL	Neo	6000 - 5500	Marinval unpubl
Aven des Corneilles	Sainte-Enimie	48	AvCor	TEMP	44.37	3.42	CAVE	Neo	4700 - 4200	Erroux 1979b
Fourches (Aven des)	Sault	84	AvFch	TEMP	44.09	5.41	CAVE	BA	3400 - 3250	Buisson-Catil et al 1997
Baume Abeurador	Félines-Minervois	34	BAbeu	MEDIT	43.33	2.60	CAVE	Neo	7400 - 6000	Vaquer, Ruas 2009
Baume Bourbon	Cabrières	30	BaBou	MEDIT	43.90	4.47	CAVE	Neo	7400 - 7000	Erroux 1976
Baou des Noirs	Vence	06	BadNo	MEDIT	43.72	7.12	VILLAGE	BA	3400 - 2750	Bouby unpubl
Baou des Noirs	Vence	06	BadNo	MEDIT	43.72	7.12	VILLAGE	IA1	2600 - 2400	Bouby unpubl
Baume d'Oullins	Le Garn	30	BaOul	MEDIT	44.30	4.48	CAVE	Neo	7400 - 7000	Marinval unpubl
Baou Roux	Bouc-Bel-Air	13	BaRx	MEDIT	43.45	5.42	RURAL	BA	4200 - 2750	Marinval 1988
Baou Roux	Bouc-Bel-Air	13	BaRx	MEDIT	43.45	5.42	VILLAGE	IA2	2425 - 2375	Marinval 1988
Bayard (Valence)	Guilherand-Granges	07	Baya	TEMP	44.93	4.86	RURAL	IA1	2800 - 2700	Durand unpubl
Bouc-Bel-Air	Bouc-Bel-Air	13	BcBA	MEDIT	43.45	5.42	RURAL	Neo	5500 - 4200	Figueiral unpubl
Colombier (Le)	Beaumont	63	BClmb	TEMP	45.75	3.10	RURAL	Neo	6000 - 5500	Cabanis, Bouby 2016
Béragne	Trèbes	11	Bergn	MEDIT	43.22	2.43	RURAL	IA2	2125 - 2050	Figueiral unpubl
Foisses (Les)	Beaumont	63	BFoiss	TEMP	45.75	3.10	RURAL	Neo	6000 - 5500	Cabanis, Bouby 2016
Baume Fontbrégoua	Salernes	83	BFtbg	MEDIT	43.55	6.23	CAVE	Neo	7500 - 5500	Savard 2000
Balme Gontran	Chaley	01	Bgon	TEMP	45.95	5.53	CAVE	BA	3100 - 2900	Bouby et al. 2005
BMVR	Marseille	13	BMVR	MEDIT	43.30	5.37	OFFSITE	ERo	1900 - 1800	Bouby 2014
BMVR	Marseille	13	BMVR	MEDIT	43.30	5.37	OFFSITE	LRo	1600 - 1500	Bouby 2014
BMVR	Marseille	13	BMVR	MEDIT	43.30	5.37	OFFSITE	IA2	2300 - 2200	Bouby 2014
Nogeiret (Le)	Bollène	64	BoNgrt	MEDIT	44.28	4.75	RURAL	BA	4200 - 2750	Pinaud unpubl
Le Chenet des Pierres	Bozel	73	BozCP	TEMP	45.45	6.65	RURAL	Neo	6500 - 5500	Martin et al 2016
BPNL	Lyon	69	BPNL	TEMP	45.75	4.82	RURAL	ERo	2000 - 1800	Bouby 2014
BPNL	Lyon	69	BPNL	TEMP	45.75	4.82	RURAL	LRo	1700 - 1600	Bouby 2014
BPNL	Lyon	69	BPNL	TEMP	45.75	4.82	RURAL	BA	4200 - 3300	Vital et al 2007
BPNL	Lyon	69	BPNL	TEMP	45.75	4.82	RURAL	IA1/2	2525 - 2450	Bouby 2014
BPNL - funerary	Lyon	69	BPNLfu	TEMP	45.75	4.82	FUN	ERo	2027 - 1750	Bouby 2014
Barreau de la Devèze Sud	Béziers	34	BrDev	MEDIT	43.35	3.25	RURAL	Neo	6000 - 5500	Martin et al 2016
Brégoule	Soyons	07	Brego	TEMP	44.88	4.85	RURAL	BA	2800 - 2700	Bouby 2014
Buffe Arnaud	Saint-Martin-de-Brômes	04	BufAr	MEDIT	43.77	5.95	VILLAGE	IA2	2150 - 2100	Marinval unpubl
BDS	Béziers	34	BzBDS	MEDIT	43.35	3.25	RURAL	Neo	6500 - 5500	Figueiral unpubl
Crès (Le)	Béziers	34	BzCrs	MEDIT	43.35	3.25	RURAL	Neo	6500 - 6000	Martin et al 2016
Mazeran	Béziers	34	BzMaz	MEDIT	43.35	3.25	RURAL	ERo	1900 - 1700	Figueiral unpubl
Careiron & Pesquier	Milhaud	30	C&Pesq	MEDIT	43.78	4.30	RURAL	LRo	1625 - 1600	Bouby 2014
Coudoumines 1365B (Les)	Caramany	66	CaCoud	MEDIT	42.73	2.57	RURAL	Neo	6500 - 6200	Marinval unpubl
Coudoumines 541 (Les)	Caramany	66	CaCoud	MEDIT	42.73	2.57	RURAL	LRo	1630 - 1500	Ros unpubl
Calade	Cabasse	83	Calad	MEDIT	43.42	6.23	FUN	ERo	1960 - 1750	Marinval 1993a
Pla de l'Aïgo	Caramany	66	CaPla	MEDIT	42.73	2.57	RURAL	ERo	1950 - 1650	Ros unpubl
Carrerrassa (La)	Perpignan	66	Carre	MEDIT	42.68	2.88	RURAL	BA	4200 - 3650	Bouby unpubl
Carsac	Carcassonne	11	Carsa	MEDIT	43.22	2.35	VILLAGE	BA	3050 - 2750	Erroux 1986
Carsac	Carcassonne	11	Carsa	MEDIT	43.22	2.35	VILLAGE	IA1	2750 - 2450	Erroux 1986
L'Alba	Castres	81	CasAlb	TEMP	43.60	2.25	RURAL	IA2	2175 - 2125	Durand unpubl
Castellas	Rognac	13	CasRo	MEDIT	43.48	5.23	VILLAGE	IA2	2150 - 2050	Chabot 1972
Castellan	Istres	13	CastIs	MEDIT	43.52	4.98	VILLAGE	IA2	2200 - 2100	Bouby 2014
Colline Saint Jacques	Cavaillon	84	CaStJ	MEDIT	43.84	5.04	VILLAGE	IA2	2125 - 2000	Marinval unpubl
Terrofort 268	Caramany	66	CaTer	MEDIT	42.73	2.57	RURAL	LRo	1630 - 1550	Ros unpubl
Vergers (Les)	Cavaillon	84	CaVgrs	MEDIT	43.84	5.04	OFFSITE	ERo	1900 - 1900	Pinaud unpubl
Camp del Viver	Baho	66	CaViv	MEDIT	42.70	2.83	RURAL	BA	3100 - 2920	Toledo i Mur et al 2018
Bourbousson 1	Crest	26	CB1	TEMP	44.73	5.03	RURAL	IA1/2	2500 - 2450	Bouby 2014
Bourbousson 3	Crest	26	CB3	TEMP	44.73	5.03	RURAL	ERo	1825 - 1750	Bouby 2014
Bourbousson 3	Crest	26	CB3	TEMP	44.73	5.03	RURAL	LRo	1725 - 1700	Bouby 2014
Combe Grèze	La Cresse	12	CbGrz	TEMP	44.18	3.13	CAVE	Neo	8000 - 6500	Durand unpubl
Centre des Impôts	Millau	12	CDImp	TEMP	44.10	3.08	RURAL	Neo	4700 - 4400	Bouby et al 2020b
Bente Farine	Céret	66	CerBF	TEMP	42.48	2.75	RURAL	LRo	1650 - 1600	Ros and Ruas, 2017
Château Royal	Céret	66	CerCR	TEMP	42.48	2.75	RURAL	LRo	1550 - 1450	Ros unpubl
Sant Jaume del Crest	Céret	66	CerSJC	TEMP	42.48	2.75	RURAL	ERo	1925 - 1850	Figueiral unpubl
Tumbas (Les)	Céret	66	CerTu	TEMP	42.48	2.75	RURAL	ERo	2100 - 1900	Ros unpubl
Pâtural 2 (Le)	Clermont-Ferrand	63	CFPt2	TEMP	45.78	3.08	RURAL	IA1	2650 - 2520	Hajnalovà 2006
Champs (Les)	Baix	07	Champ	TEMP	44.72	4.77	RURAL	IA1	2750 - 2450	Cabanis et al. 2021
Château-l'Arc	Fuveau	13	ChArc	MEDIT	43.45	5.57	RURAL	Neo	6500 - 5500	Figueiral unpubl
Châtillon	Chindrieux	73	ChCha	TEMP	45.82	5.85	RURAL	BA	2906 - 2814	Jacquot 1994
Champ du Perrier (ZAC du)	Fareins	01	ChdPe	TEMP	46.02	4.77	RURAL	IA1	2850 - 2600	Cabanis unpubl
Champ du Poste (Le)	Carcassonne	11	ChdPo	MEDIT	43.22	2.35	RURAL	Neo	6500 - 6000	Convertini, Georjon 2018
Christol 3	Carcassonne	11	Chri3	MEDIT	43.22	2.35	RURAL	IA1/2	2525 - 2450	Figueiral et al 2015
Claparouse	Lagnes	84	Clapa	MEDIT	43.90	5.12	RURAL	Neo	6000 - 5500	Bouby Léa 2006
Charnage (Rue de)	Chens-sur-Léman	74	CLChar	TEMP	46.33	6.27	RURAL	BA	3400 - 2800	Cousseran-Néré et al. 2020
Clos de la Lombarde	Narbonne	11	ClLomb	MEDIT	43.18	3.00	URBAN	ERo	2000 - 1700	Rovira 2015
Cloche (La)	Les Pennes-Mirabeau	13	Cloch	MEDIT	43.41	5.32	VILLAGE	IA2	2100 - 2050	Marinval 1988
Pré d'Ancy/ Charnage (Rue de)	Chens-sur-Léman	74	CLPAC	TEMP	46.33	6.27	RURAL	BA	4200 - 2900	Cousseran-Néré et al. 2020
Véreître	Chens-sur-Léman	74	CLVer	TEMP	46.33	6.27	RURAL	BA	3300 - 3050	Cousseran-Néré et al. 2020
Coumo dal Cat	Padern	11	CoCat	MEDIT	42.87	2.67	RURAL	IA1	2600 - 2500	Guilaine et al 2016
Chomette (La)	Cournon-d'Auvergne	63	CoCho	TEMP	45.75	3.22	RURAL	BA	3700 - 3500	Carozza et al 2006
Cova de l'Esperit	Salses-le-Château	66	CoEsp	MEDIT	42.83	2.92	CAVE	Neo	7400 - 7000	Marinval 1988
Collombières (Les)	Saint-Jean-le-Vieux	01	Collo	TEMP	46.03	5.39	RURAL	ERo	2025 - 1750	Flottes unpubl
Collombières (Les)	Saint-Jean-le-Vieux	01	Collo	TEMP	46.03	5.39	RURAL	IA2	2370 - 2172	Flottes unpubl
Cormail, Locus 3	Espaly-Saint-Marcel	43	CoLoc3	TEMP	45.05	3.87	RURAL	Neo	6000 - 5500	Bouby 1993
Les Combariès	Baraqueville	12	Comba	TEMP	44.28	2.43	VILLAGE	IA2	2125 - 2075	Durand unpubl
Condamine 7	Vauvert	30	Cond7	MEDIT	43.70	4.28	RURAL	IA1	2525 - 2475	Séjalon et al 2014
Condamine (La)	Villeneuve-Minervois	11	Conda	MEDIT	43.32	2.47	RURAL	IA2	2500 - 2400	Figueiral et al 2015
Plaines (Les)	Cournon-d'Auvergne	63	CoPla	TEMP	45.75	3.22	RURAL	BA	4100 - 2800	Cabanis et al. 2010
Coudouneu	Lançon-Provence	13	Coudo	MEDIT	43.58	5.13	VILLAGE	IA2	2425 - 2400	Verdin 1997
Courtinals	Mourèze	34	Courti	MEDIT	43.62	3.37	RURAL	IA1/2	2450 - 2450	Garcia et al 1990
Les Croisières	Guilherand-Granges	07	Croisi	TEMP	44.93	4.86	RURAL	Neo	7000 - 5500	Durand unpubl
Les Croisières	Guilherand-Granges	07	Croisi	TEMP	44.93	4.86	RURAL	IA1	2850 - 2600	Durand unpubl
Cariaux	Frontonas	38	CrxFro	TEMP	45.65	5.18	OFFSITE	ERo	2000 - 1770	Bouby 2014
Castelle et Fromigue - funerary	Lattes	34	CstFro	MEDIT	43.57	3.90	FUN	ERo	2000 - 1800	Figueiral unpubl
Castelle GR	Lattes	34	CstGR	MEDIT	43.57	3.90	RURAL	ERo	1900 - 1700	Figueiral unpubl

Site	Municipality	Dep.	CodeSite	Bioclim	latitude	longitude	type	Period	DATE BP	Reference
Caisses Saint Jean	Mouriès	13	CStJea	MEDIT	43.68	4.87	VILLAGE	IA2	2455 - 2100	Marinval 1988
Castelle-Pahon-Pinède - funerary	Lattes	34	CstPP	MEDIT	43.57	3.90	FUN	ERo	2000 - 1800	Figueiral unpubl
Côtes de Roquefort	Saint-Rome-de-Cernon	12	CtRoq	TEMP	44.02	2.97	CAVE	Neo	4800 - 4200	Vernet 1973
Croix Saint Etienne (La)	Chanonat	63	CxStE	TEMP	45.68	3.10	RURAL	Neo	5500 - 4200	Cabanis unpubl
Machal (Le)	Dallet	63	DaMch	TEMP	45.77	3.23	RURAL	BA	4050 - 3600	Bouby 1993
Baume des Anges	Donzère	26	DBAg	MEDIT	44.45	4.72	CAVE	BA	2900 - 2750	Marinval 1986
Derrière le Château	Géovreissiat	01	DeCha	TEMP	46.20	5.57	RURAL	LRo	1700 - 1500	Bouby 2014
Doulouzargues	Codognan	30	Doulz	MEDIT	43.73	4.23	RURAL	IA2	2300 - 2200	Figueiral unpubl
Dourgne (Roc de)	Fontanès-de-Sault	11	Dourg	TEMP	42.77	2.08	CAVE	Neo	7000 - 6500	Marinval 1993b
Maison Philippon	Elne	66	ElMP	MEDIT	42.60	2.97	RURAL	ERo	2000 - 1900	Ros, 2020
Encombres	Quarante	34	Enc40	MEDIT	43.35	2.97	RURAL	Neo	6500 - 6000	Martin et al 2016
Escarpes 2	Agde	34	Escp2	MEDIT	43.32	3.47	RURAL	ERo	2000 - 1900	Bouby 2014
Escarpes 2	Agde	34	Escp2	MEDIT	43.32	3.47	RURAL	LRo	1650 - 1500	Bouby 2014
Estagnon	Fos-sur-Mer	13	Estag	MEDIT	43.43	4.95	VILLAGE	ERo	1950 - 1920	Marty et al 2016
Menhir (Le)	Eyne	66	EyMen	TEMP	42.47	2.07	RURAL	BA	3050 - 2800	Bouby, Ruas unpubl
Camelin (Le)	Fréjus	83	FrCam	MEDIT	43.43	6.74	URBAN	ERo	2040 - 1800	Tillier 2019
10 rue des frères de Turenne	Rodez	12	FrdTur	TEMP	44.33	2.57	URBAN	ERo	2000 - 1900	Durand unpubl
Pauvadou	Fréjus	83	FrPauv	MEDIT	43.43	6.74	FUN	ERo	1950 - 1800	Marinval 1993a
Saint Lambert	Fréjus	83	FrSLam	MEDIT	43.43	6.74	FUN	ERo	1950 - 1800	Marinval 1993a
Théâtre d'Agglomération	Fréjus	83	FrThAg	MEDIT	43.43	6.74	OFFSITE	ERo	2030 - 1970	Excoffon et al 2006
Théâtre de Villeneuve	Fréjus	83	FrThVi	MEDIT	43.43	6.74	OFFSITE	ERo	2025 - 1800	Bouby et al 2011b
Théâtre de Villeneuve	Fréjus	83	FrThVi	MEDIT	43.43	6.74	OFFSITE	LRo	1800 - 1500	Bouby et al 2011b
Font Juvénal	Conques-sur-Orbiel	11	FtJu	MEDIT	43.27	2.40	CAVE	Neo	7000 - 6000	Marinval 1988
Font aux Pigeons	Châteauneuf-les-Martigues	13	FtPig	MEDIT	43.38	5.17	CAVE	Neo	7500 - 7000	Marinval 1988
Font aux Pigeons	Châteauneuf-les-Martigues	13	FtPig	MEDIT	43.38	5.17	CAVE	Neo	7000 - 6500	Courtin, Erroux, Thommeret 1974
Gabache XVI	Bram	11	Gab16	TEMP	43.25	2.12	RURAL	BA	3600 - 3350	de Labriffe et al 2016
Gach	Cruzy	34	Gach	MEDIT	43.35	2.95	RURAL	IA1	2750 - 2450	Marinval unpubl
Plan Tour	Gailhan	30	GaPdT	MEDIT	43.83	4.03	VILLAGE	IA1/2	2500 - 2400	Erroux 1980
Plan Tour	Gailhan	30	GaPdT	MEDIT	43.83	4.03	VILLAGE	IA2	2400 - 2300	Erroux 1980
Gasquinoy	Béziers	34	Gasq	MEDIT	43.35	3.25	RURAL	ERo	2000 - 1800	Figueiral et al 2010
Grande Borne (La)	Aulnat	63	GdBor	TEMP	45.80	3.17	RURAL	IA1	2800 - 2650	Collis et al 1979
Grande Chaberte (La)	La Garde	83	GdChab	MEDIT	43.13	6.02	RURAL	ERo	1975 - 1700	Tillier 2019
Grande Chaberte (La) - funerary	La Garde	83	GdChab	MEDIT	43.13	6.02	FUN	LRo	1800 - 1650	Tillier 2019
Grande Chaberte (La)	La Garde	83	GdChab	MEDIT	43.13	6.02	RURAL	LRo	1700 - 1500	Tillier 2019
Grand Contras	Graveson	13	GdCont	MEDIT	43.85	4.77	RURAL	ERo	2000 - 1800	Figueiral unpubl
Grande Rivoire (La)	Sassenage	38	GdRiv	TEMP	45.20	5.67	CAVE	Neo	7300 - 6500	Martin 2010
Grande Rivoire (La)	Sassenage	38	GdRiv	TEMP	45.20	5.67	CAVE	Neo	6500 - 5500	Martin et al 2016
Grande Rivoire (La)	Sassenage	38	GdRiv	TEMP	45.20	5.67	CAVE	IA2	2100 - 2000	Martin 2003
Grande Terre	Alba-la-Romaine	07	GdTer	TEMP	44.55	4.59	RURAL	IA1	2500 - 2400	Cabanis et al. 2021
Gardon (Grotte du)	Ambérieu-en-Bugey	01	GGard	TEMP	45.95	5.35	CAVE	Neo	7300 - 6250	Bouby 2009
Gardon (Grotte du)	Ambérieu-en-Bugey	01	GGard	TEMP	45.95	5.35	CAVE	BA	3600 - 3000	Bouby unpubl
Gardon (Grotte du)	Ambérieu-en-Bugey	01	GGard	TEMP	45.95	5.35	CAVE	IA2	2300 - 2200	Bouby 2014
Gardi	Goult	84	GoGard	MEDIT	43.87	5.25	VILLAGE	IA1/2	2550 - 2475	Marinval unpubl
Goiffieux	Saint-Laurent-d'Agny	69	Goiff	TEMP	45.63	4.68	RURAL	ERo	2000 - 1900	Poux et al 2013
Goulles (Les)	Garde-Freinet (La)	83	Goull	MEDIT	43.32	6.47	FUN	IA1/2	2440 - 2440	Pinaud unpubl
Grésine Est	Brison-Saint-Innocent	73	GRSE	TEMP	45.72	5.88	RURAL	BA	2905 - 2850	Bouby, Billaud 2001
Grésine Ouest	Brison-Saint-Innocent	73	GRSW	TEMP	45.72	5.88	RURAL	BA	2905 - 2850	Bouby, Billaud 2001
Aigle (Grotte de l')	Méjannes-le-Clap	30	GtAig	MEDIT	44.23	4.33	CAVE	Neo	7200 - 7000	Erroux 1979a
Aven du Poteau (Grotte)	Saint-Pons-de-Thomières	34	GtAPo	TEMP	43.49	2.76	CAVE	Neo	5500 - 5100	Erroux unpubl
Bauduen (Grotte de)	Bauduen	83	GtBaud	TEMP	43.73	6.18	CAVE	BA	3350 - 2800	Erroux, Courtin 1974
Bélesta (Grotte de)	Bélesta	66	GtBel	MEDIT	42.72	2.60	CAVE	BA	3650 - 2800	Buxo I Capdevila 1993
Bélesta (Grotte de)	Bélesta	66	GtBel	MEDIT	42.72	2.60	CAVE	IA1	2750 - 2450	Buxo I Capdevila 1993
Buffens (grotte de)	Caunes-Minervois	11	GtBuf	MEDIT	43.32	2.53	CAVE	BA	3650 - 3350	Guilaine, Hopf 1984
Cazals (Grotte de)	Sallèles-Cabardès	11	GtCaz	MEDIT	43.32	2.43	CAVE	BA	3740 - 3535	Van Zeist et al 1983
C et G (Grotte)	Baudinard-sur-Verdon	83	GtCG	TEMP	43.72	6.13	CAVE	Neo	6000 - 5500	Erroux, Courtin 1974
G (Grotte)	Baudinard-sur-Verdon	83	GtCG	TEMP	43.72	6.13	CAVE	BA	3650 - 2800	Marinval unpubl
Gazel (Grotte)	Sallèles-d'Aude	11	GtGaz	MEDIT	43.27	2.95	CAVE	Neo	7400 - 7000	Bouby unpubl
Hasard (Grotte du)	Tharaux	30	GtHas	MEDIT	44.25	4.32	CAVE	BA	3200 - 3050	Erroux 1993
Lombard (Grotte)	Saint-Vallier-de-Thiey	06	GtLom	TEMP	43.70	6.85	CAVE	Neo	7500 - 7000	Marinval unpubl
Montou (Grotte de)	Corbère-les-Cabanes	66	GtMto	MEDIT	42.65	2.68	CAVE	BA	4200 - 2800	Buxo 2006
Montou (Grotte de)	Corbère-les-Cabanes	66	GtMto	MEDIT	42.65	2.68	CAVE	IA2	2425 - 2300	Buxo 2006
Murée (Grotte)	Montagnac-Montpezat	04	GtMur	MEDIT	43.77	6.10	CAVE	BA	3600 - 3500	Erroux, Courtin 1974
Pontiar (Grotte du)	Vallon-Pont-d'Arc	07	GtPon	MEDIT	44.40	4.40	CAVE	IA1	2550 - 2550	Erroux unpubl
Sargel (Grotte)	Saint-Rome-de-Cernon	12	GtSag	TEMP	44.02	2.97	CAVE	Neo	6500 - 4200	Erroux, Poulain 1984
Salpétrière (grotte de)	Remoulins	30	GtSal	MEDIT	43.93	4.57	CAVE	Neo	4700 - 4200	Vernet 1973
Saint Marcel (Grotte)	Saint-Marcel-d'Ardèche	07	GtSMa	MEDIT	44.32	4.62	CAVE	Neo	7500 - 4800	Erroux 1988
Saint Marcel (Grotte)	Saint-Marcel-d'Ardèche	07	GtSMa	MEDIT	44.32	4.62	CAVE	BA	3300 - 3050	Erroux 1988
Guérine	Cabasse	83	Gueri	MEDIT	43.42	6.23	FUN	ERo	1925 - 1875	Marinval 1993a
Martigues (île)	Martigues	13	IlMart	MEDIT	43.40	5.05	VILLAGE	IA2	2400 - 2200	Marinval 1988
île Saint Martin	Gruissan	11	IlStMa	MEDIT	43.10	3.08	RURAL	ERo	2000 - 1800	Tillier 2019
île Saint Martin	Gruissan	11	IlStMa	MEDIT	43.10	3.08	RURAL	LRo	1700 - 1500	Tillier 2019
Jas del Biau 2	Millau	12	JdBia2	TEMP	44.10	3.08	RURAL	Neo	5400 - 4400	Bouby et al 2020b
Jas del Biau	Millau	12	JDBia	TEMP	44.10	3.08	CAVE	Neo	5400 - 4400	Bouby et al 2020b
Jardin d'hiver	Arles	13	JdHiv	MEDIT	43.67	4.63	VILLAGE	IA1/2	2500 - 2400	Marinval 1988
Jardin d'hiver	Arles	13	JdHiv	MEDIT	43.67	4.63	VILLAGE	IA2	2425 - 2400	Marinval 1988
Jouffe	Montmirat	30	Jouff	MEDIT	43.92	4.10	VILLAGE	IA1/2	2525 - 2450	Marinval unpubl
Jurières Basses	Puissalicon	34	JuBs	MEDIT	43.45	3.23	RURAL	LRo	1600 - 1400	Bouchette 1998
Jules Verne 10	Marseille	13	JV10	MEDIT	43.30	5.37	URBAN	ERo	1925 - 1800	Bouby 2014
Jules Verne 10	Marseille	13	JV10	MEDIT	43.30	5.37	URBAN	LRo	1800 - 1600	Bouby 2014
Jules Verne 11	Marseille	13	JV11	MEDIT	43.30	5.37	URBAN	IA2	2300 - 2100	Bouby 2014
Jules Verne 14	Marseille	13	JV14	MEDIT	43.30	5.37	URBAN	IA1	2600 - 2500	Bouby 2014
Jules Verne 14	Marseille	13	JV14	MEDIT	43.30	5.37	URBAN	IA1/2	2525 - 2400	Bouby 2014
Jardins de Vert Parc	Castelnau-le-Lez	34	JVV	MEDIT	43.63	3.90	RURAL	Neo	6000 - 5500	Martin et al 2016
Capelles (Lac de)	Narbonne	11	LaCap	MEDIT	43.18	3.00	RURAL	ERo	2030 - 1990	Tillier 2019
La Garanne	Berre-l'Etang	13	LaGar	MEDIT	43.47	5.18	RURAL	ERo	1900 - 1750	Rovira unpubl
La Garanne	Berre-l'Etang	13	LaGar	MEDIT	43.47	5.18	RURAL	LRo	1800 - 1650	Rovira unpubl
Girardes (Les)	Lapalud	84	LaGird	MEDIT	44.30	4.68	RURAL	ERo	1900 - 1800	Bouby 2014
Gravette (La)	Cavanac	11	LaGrav	MEDIT	43.17	2.33	RURAL	BA	2950 - 2950	Marinval unpubl
Gravette (La)	Cavanac	11	LaGrav	MEDIT	43.17	2.33	RURAL	IA1	2525 - 2500	Marinval unpubl
Roberte (La)	Châteauneuf-du-Rhône	26	LaRob	MEDIT	44.48	4.72	RURAL	Neo	6000 - 5500	Martin et al 2016
Roque (La)	Graveson	13	LaRoq	MEDIT	43.85	4.77	RURAL	IA2	2125 - 2075	Bouby 2014
La Royale	Villardonnel	11	LaRoy	TEMP	43.33	2.32	RURAL	BA	4200 - 3700	Figueiral unpubl
Salle (La)	Carcassonne	11	LaSal	MEDIT	43.22	2.35	RURAL	Neo	6000 - 5500	Marinval 2003
Lautagne	Valence	26	Lautag	TEMP	44.93	4.90	VILLAGE	ERo	2125 - 2025	Ros unpubl
Cailar (Le)	Le Cailar	30	Lcail	MEDIT	43.68	4.24	RURAL	IA2	2500 - 2200	Rovira, Alonso 2017
Conque (La)	Mèze	34	LConq	MEDIT	43.42	3.60	RURAL	BA	3200 - 2900	Bouby 2014
Laouret (Le)	Floure	11	LeLao	MEDIT	43.18	2.48	RURAL	BA	3050 - 2900	Marinval unpubl
Fangade (La)	Sète	34	LFang	MEDIT	43.40	3.68	RURAL	BA	3450 - 2900	Bouby et al 1999
Gramière (La)	Castillon-du-Gard	30	LGram	MEDIT	43.97	4.55	RURAL	ERo	2000 - 1700	Buffat et al 2009
Gramière (La)	Castillon-du-Gard	30	LGram	MEDIT	43.97	4.55	RURAL	LRo	1750 - 1600	Buffat et al 2009
Joncasses (Les)	Cournonterral	34	Ljoncs	MEDIT	43.55	3.72	FUN	IA1	2700 - 2600	Figueiral, Ivorra unpubl
Liquière (La)	Calvisson	30	Lliq	MEDIT	43.78	4.18	VILLAGE	IA1	2600 - 2575	Erroux 1984
Lladre (Lo)	Llo	66	LLLa	TEMP	42.45	2.05	RURAL	BA	3050 - 2800	Ruas et al 2009
Moulin (Le)	Barret-de-Lioure	26	Lmoul	TEMP	44.18	5.50	RURAL	Neo	6500 - 6000	Martin et al 2016

Site	Municipality	Dep.	CodeSite	Bioclim	latitude	longitude	type	Period	DATE BP	Reference
Logis de Berre (Le)	Les Granges-Gontardes	26	LoBer	MEDIT	44.42	4.77	RURAL	Neo	6000 - 5500	Martin et al 2016
Bourbou	Loupian	34	LpBo	MEDIT	43.45	3.62	RURAL	LRo	1750 - 1600	Rovira unpubl
Camp de la Torre	Le Perthus	66	LPCTo	MEDIT	42.47	2.87	RURAL	IA2	2200 - 2075	Ros unpubl
Près Bas	Loupian	34	LpPBa	MEDIT	43.45	3.62	RURAL	LRo	1700 - 1670	Rovira unpubl
Cougourlude (La)	Lattes	34	LtCog	MEDIT	43.57	3.90	RURAL	IA1	2550 - 2475	Figueiral, Bouby unpubl
Chemin Saint Pierre	Lattes	34	LtCSP	MEDIT	43.57	3.90	FUN	IA2	2500 - 2300	Figueiral unpubl
Lattara	Lattes	34	LtLat	MEDIT	43.57	3.90	URBAN	ERo	2000 - 1875	Buxó 1992, 1993, 1996, 2005, Tillier 2019
Lattara	Lattes	34	LtLat	MEDIT	43.57	3.90	URBAN	IA1/2	2500 - 2400	Alonso, Rovira 2016, Rovira, Alonso 2010, 2017
Lattara	Lattes	34	LtLat	MEDIT	43.57	3.90	URBAN	IA2	2400 - 2100	Buxó 2003b, Alonso, Rovira 2016, Rovira, Alonso 2017
Lattara	Lattes	34	LtLat	MEDIT	43.57	3.90	URBAN	IA2	2100 - 2000	Buxó 1992, 1993, 1996, 2005, Tillier 2019
Lattara - Cultuel	Lattes	34	LtLatC	MEDIT	43.57	3.90	FUN	ERo	1975 - 1940	Rovira Chabal 2008
Lattara - Port	Lattes	34	LtLatP	MEDIT	43.57	3.90	URBAN	ERo	2050 - 1800	Tillier 2019, Rovira et al unpubl
Mas de Causse	Lattes	34	LtMdC	MEDIT	43.57	3.90	RURAL	ERo	2000 - 1900	Rovira unpubl
Mas de Causse	Lattes	34	LtMdC	MEDIT	43.57	3.90	RURAL	LRo	1800 - 1600	Rovira unpubl
Mas de Causse	Lattes	34	LtMdC	MEDIT	43.57	3.90	RURAL	IA1	2600 - 2500	Rovira unpubl
Mas de Causse	Lattes	34	LtMdC	MEDIT	43.57	3.90	RURAL	IA1/2	2525 - 2400	Rovira, Alonso 2017
Mas de Causse	Lattes	34	LtMdC	MEDIT	43.57	3.90	RURAL	IA2	2500 - 2300	Rovira, Alonso 2017
Mas de Causse	Lattes	34	LtMdC	MEDIT	43.57	3.90	RURAL	IA2	2100 - 2000	Rovira unpubl
Mas de Causse - Cultual	Lattes	34	LtMdCC	MEDIT	43.57	3.90	FUN	IA2	2500 - 2300	Rovira unpubl
Port Ariane	Lattes	34	LtPA	MEDIT	43.57	3.90	RURAL	BA	3400 - 2800	Alonso et al 2007
Port Ariane	Lattes	34	LtPA	MEDIT	43.57	3.90	RURAL	IA1	2700 - 2650	Alonso et al 2007
Port Ariane	Lattes	34	LtPA	MEDIT	43.57	3.90	RURAL	IA2	2350 - 2300	Alonso et al 2007
Berthet 1	Lyon	69	LyBer1	TEMP	45.75	4.82	RURAL	IA1/2	2525 - 2450	Bouby 2014
Berthet 1 - funerary	Lyon	69	LyBer1F	TEMP	45.75	4.82	FUN	ERo	1825 - 1775	Bouby 2014
Berthet 2	Lyon	69	LyBer2	TEMP	45.75	4.82	RURAL	BA	2900 - 2700	Bouby 2014
Berthet 2	Lyon	69	LyBer2	TEMP	45.75	4.82	RURAL	IA1/2	2525 - 2450	Bouby 2014
Contrescarpe (Rue de)	Lyon	69	LyCont	TEMP	45.75	4.82	FUN	ERo	1950 - 1900	Pradat 1994
Favorite	Lyon	69	LyFav	TEMP	45.75	4.82	FUN	ERo	2000 - 1800	Marinval 1993a
Gorge de Loup	Lyon	69	LyGdL	TEMP	45.75	4.82	RURAL	IA1/2	2525 - 2450	Marinval unpubl
Horand 2	Lyon	69	LyHor2	TEMP	45.75	4.82	FUN	ERo	2020 - 1750	Pradat 1994
Parc Saint Georges	Lyon	69	LyPSG	TEMP	45.75	4.82	URBAN	ERo	2000 - 1875	Bouby 2013
Parc Saint Georges	Lyon	69	LyPSG	TEMP	45.75	4.82	URBAN	LRo	1775 - 1575	Bouby 2013
Parc Saint Georges	Lyon	69	LyPSG	TEMP	45.75	4.82	URBAN	IA2	2200 - 2075	Bouby 2013
Quai Arloing	Lyon	69	LyQAr	TEMP	45.75	4.82	FUN	ERo	2000 - 1875	Marinval 1993a
Pierre Audry (Rue)	Lyon	69	LyRPA	TEMP	45.75	4.82	FUN	ERo	1950 - 1800	Pradat 1994
14 rue des Tuileries	Lyon	69	LyTuil	TEMP	45.75	4.82	RURAL	IA1/2	2525 - 2425	Schaal unpubl
Vaise	Lyon	69	LyVais	TEMP	45.75	4.82	RURAL	Neo	7200 - 5500	Martin et al 2016
Vaise - ZAC Industrie Nord	Lyon	69	LyZIN	TEMP	45.75	4.82	FUN	ERo	2025 - 1750	Flottes unpubl
Magasins (Les), Buzerens	Bram	11	MaBuz	TEMP	43.25	2.12	RURAL	IA2	2500 - 2400	Flottes unpubl
Magasins (Les), Buzerens	Bram	11	MaBuz	TEMP	43.25	2.12	RURAL	IA2	2120 - 2050	Flottes unpubl
Cadoules	Mauguio	34	MaCad	MEDIT	43.62	4.02	RURAL	Neo	5500 - 4200	Figueiral unpubl
Montfau	Magalas	34	MagMt	MEDIT	43.47	3.22	VILLAGE	IA1	2550 - 2475	Bacou, Bacou 1983
Terrasses Montfo	Magalas	34	MagTM	MEDIT	43.47	3.22	FUN	LRo	1700 - 1600	Figueiral unpubl
Maladrerie/Lot. du Grand Chêne	Saillans	26	MalGdC	TEMP	44.70	5.18	RURAL	LRo	1800 - 1525	Le Roy et al 2011
Peras 1	Mauguio	34	MaPe1	MEDIT	43.62	4.02	RURAL	Neo	6500 - 4200	Figueiral unpubl
Peras 2	Mauguio	34	MaPe2	MEDIT	43.62	4.02	RURAL	Neo	6500 - 5500	Figueiral unpubl
Brassières Nord	Mondragon	84	MDBN	MEDIT	44.23	4.72	RURAL	ERo	2020 - 1975	Bouby 2014
Brassières Nord	Mondragon	84	MDBN	MEDIT	44.23	4.72	RURAL	IA2	2150 - 2020	Bouby 2014
Brassières Sud	Mondragon	84	MDBS	MEDIT	44.23	4.72	RURAL	IA1	2750 - 2625	Bouby 2014
Mas de Vignoles 10	Nîmes	30	MdV10	MEDIT	43.83	4.35	RURAL	Neo	7000 - 6500	Bouby, Figueiral 2014
Mas de Vignoles 10	Nîmes	30	MdV10	MEDIT	43.83	4.35	RURAL	IA1	2750 - 2625	Bouby 2014
Mas de Vignoles 9	Nîmes	30	MdV9	MEDIT	43.83	4.35	RURAL	ERo	2000 - 1900	Figueiral, Séjalon 2014
Mas de Vignoles 9	Nîmes	30	MdV9	MEDIT	43.83	4.35	RURAL	Neo	6500 - 5500	Figueiral, Séjalon 2014
Mas de Vignoles 9	Nîmes	30	MdV9	MEDIT	43.83	4.35	RURAL	BA	2600 - 1350	Figueiral, Séjalon 2014
Mas de Vignoles 9	Nîmes	30	MdV9	MEDIT	43.83	4.35	RURAL	IA1	2750 - 2450	Figueiral, Séjalon 2014
Médor	Ornaisons	11	Medo	MEDIT	43.18	2.83	RURAL	LRo	1600 - 1400	Ruas 1989a
Miremand	Nîmes	30	Mirmd	MEDIT	43.83	4.35	RURAL	ERo	2000 - 1900	Figueiral unpubl
Mourre de la Barque	Jouques	13	ModBq	MEDIT	43.63	5.63	CAVE	BA	3650 - 2750	Marinval unpubl
Moulin d'eau	Riom	63	Moeau	TEMP	45.90	3.12	RURAL	IA1	2850 - 2650	Cabanis et al. 2010
Mourrel-Ferrat	Olonzac	34	MoFer	MEDIT	43.27	2.73	URBAN	IA2	2325 - 2275	Pinaud unpubl
Guillemot-Labouygue	Montans	81	MoGLa	TEMP	43.87	1.90	VILLAGE	ERo	2000 - 1900	Durand unpubl
Guillemot-Labouygue	Montans	81	MoGLa	TEMP	43.87	1.90	VILLAGE	IA2	2125 - 2075	Durand unpubl
Monédière (La)	Bessan	34	Moned	MEDIT	43.37	3.42	URBAN	IA1	2575 - 2525	Pinaud et al. 2020
Monédière (La)	Bessan	34	Moned	MEDIT	43.37	3.42	URBAN	IA1/2	2525 - 2450	Pinaud et al. 2020
Rouget (Le)	Montans	81	MoRou	TEMP	43.87	1.90	RURAL	IA1	2700 - 2600	Marinval unpubl
Moulin à Vent (Le)	Mèze	34	MoVt	MEDIT	43.42	3.60	RURAL	ERo	2025 - 1900	Tillier 2019
Marduel	Saint-Bonnet-du-Gard	30	Mrdu	MEDIT	43.93	4.55	VILLAGE	BA	2900 - 2750	Marinval 1988
Marduel	Saint-Bonnet-du-Gard	30	Mrdu	MEDIT	43.93	4.55	VILLAGE	IA1	2550 - 2500	Marinval 1988
Marduel	Saint-Bonnet-du-Gard	30	Mrdu	MEDIT	43.93	4.55	VILLAGE	IA1/2	2500 - 2450	Marinval 1988
Marduel	Saint-Bonnet-du-Gard	30	Mrdu	MEDIT	43.93	4.55	VILLAGE	IA2	2450 - 2150	Marinval 1988
Mas Roig	Théza	66	MRoig	MEDIT	42.63	2.95	RURAL	ERo	1990 - 1970	Ros and Ruas, 2017
Renaudi	Massac-Seran	81	MSRen	TEMP	43.67	1.85	RURAL	ERo	2000 - 1900	Lelouvier et al. 2020
Mort des Anes	Villeneuve-lès-Maguelone	34	MtAne	MEDIT	43.53	3.86	CAVE	Neo	5500 - 5300	Erroux unpubl
Bouquet (Le)	Montélimar	26	MtBouq	TEMP	44.57	4.75	RURAL	BA	3400 - 3200	Cabanis unpubl
Bouquet (Le)	Montélimar	26	MtBouq	TEMP	44.57	4.75	RURAL	IA1	2750 - 2600	Cabanis unpubl
Mont Ferrier	Tourbes	34	MtFer	MEDIT	43.45	3.38	RURAL	ERo	2000 - 1900	Fabre et al 2009
Montjean	Gassin	83	Mtjea	MEDIT	43.22	6.58	VILLAGE	IA1/2	2525 - 2475	Marinval 1988
Mont Joui	Florensac	34	MtJo	MEDIT	43.38	3.45	VILLAGE	IA1/2	2525 - 2475	Bouby 2014
Juvignac	Montpellier	34	MtpJuv	MEDIT	43.60	3.88	RURAL	Neo	5500 - 4200	Figueiral unpubl
Lycée Technique	Montpellier	34	MtpLT	MEDIT	43.60	3.88	RURAL	IA1	2700 - 2600	Erroux 1966
Mont Redon	Carcassonne	11	MtRed	MEDIT	43.22	2.35	RURAL	ERo	1900 - 1800	Figueiral unpubl
Beaulieu	Monteux	84	MtxBea	MEDIT	44.03	5.00	RURAL	ERo	2000 - 1900	Rovira unpubl
Aubettes (Les)	Mudaison	34	MuAub	MEDIT	43.65	4.04	RURAL	Rom	2000 - 1600	Figueiral unpubl
Nages	Nages-et-Solorgues	30	Nage	MEDIT	43.78	4.23	RURAL	ERo	2000 - 1999	Pottrain, Py 1975
Nautique (La)	Narbonne	11	Nautiq	MEDIT	43.18	3.00	RURAL	ERo	2025 - 1750	Tillier 2019
Jean Jaurès (Avenue)	Nîmes	30	NAvJJ	MEDIT	43.83	4.35	FUN	ERo	2000 - 1800	Figueiral unpubl
Notre Dame de Pitié	Marignane	13	NDPit	MEDIT	43.42	5.22	VILLAGE	IA2	2220 - 2190	Marinval unpubl
Mas des Abeilles	Nîmes	30	NMdAb	MEDIT	43.83	4.35	RURAL	Neo	5500 - 4200	Figueiral in Bel 2017
Mas des Abeilles	Nîmes	30	NMdAb	MEDIT	43.83	4.35	RURAL	IA1	2700 - 2600	Figueiral in Bel 2017
Mas Neuf	Nîmes	30	NMNeu	MEDIT	43.83	4.35	RURAL	Neo	7000 - 6500	Bouby, Figueiral 2014
Assas (Place)	Nîmes	30	NPAs	MEDIT	43.83	4.35	URBAN	ERo	2000 - 1900	Bouchette et al 2017
Parking Jean Jaurès	Nîmes	30	NPJJ	MEDIT	43.83	4.35	URBAN	ERo	2025 - 1700	Bouchette unpubl
Parking Jean Jaurès	Nîmes	30	NPJJ	MEDIT	43.83	4.35	URBAN	LRo	1625 - 1475	Bouchette unpubl
Parking Jean Jaurès	Nîmes	30	NPJJ	MEDIT	43.83	4.35	URBAN	IA2	2150 - 2050	Bouchette unpubl
Opme le Cimetière	Romagnat	63	OpCim	TEMP	45.73	3.10	RURAL	BA	4135 - 3830	Dartevelle et al 2004
Saint Marcel (Oppidum)	Le Pègue	26	OpStM	MEDIT	44.43	5.05	VILLAGE	IA1/2	2500 - 2480	Marinval1988
Saint Marcel (Oppidum)	Le Pègue	26	OpStM	MEDIT	44.43	5.05	VILLAGE	IA2	2400 - 2375	Marinval1988
Colomina d'en Maurell	Ortaffa	66	OrCEM	MEDIT	42.58	2.93	RURAL	IA2	2360 - 2200	Bouby unpubl
Closeraie (La)	Orange	84	OrClo	MEDIT	44.13	4.80	FUN	ERo	2010 - 1800	Delbois 2016
Pujals 4	Ortaffa	66	OrPuj	MEDIT	42.58	2.93	RURAL	IA2	2200 - 2100	Ros, 2020
Tourteix (Le)	Orcet	63	OrTxt	TEMP	45.70	3.18	RURAL	BA	3600 - 3200	Bouby 2000
Bousquetas	Paulhan	34	PaBsq	MEDIT	43.53	3.45	RURAL	Neo	5000 - 4700	Carozza et al 1999
Pré-aux-Pêcheurs	Antibes	06	PaPe	MEDIT	43.58	7.12	URBAN	ERo	2083 - 1930	Figueiral unpubl, Tillier 2019
Pré-aux-Pêcheurs	Antibes	06	PaPe	MEDIT	43.58	7.12	URBAN	LRo	1850 - 1530	Figueiral unpubl, Tillier 2019

Site	Municipality	Dep.	CodeSite	Bioclim	latitude	longitude	type	Period	DATE BP	Reference
Puech Haut	Paulhan	34	PaPH	MEDIT	43.53	3.45	RURAL	Neo	5300 - 4300	Bouby et al 2020b
Champ Lamet III	Pont-du-Château	63	PdCCL3	TEMP	45.80	3.25	RURAL	Neo	6000 - 5500	Pelletier, Cabanis 2006
Champ Lamet III	Pont-du-Château	63	PdCCL3	TEMP	45.80	3.25	RURAL	BA	3700 - 3500	Pelletier, Cabanis 2006
Puech de Mus	Sainte-Eulalie-de-Cernon	12	PdMus	TEMP	43.98	3.13	VILLAGE	IA1	2700 - 2600	Durand unpubl
Puech de Mus	Sainte-Eulalie-de-Cernon	12	PdMus	TEMP	43.98	3.13	VILLAGE	IA1/2	2600 - 2500	Durand unpubl
Puech de Mus	Sainte-Eulalie-de-Cernon	12	PdMus	TEMP	43.98	3.13	VILLAGE	IA2	2400 - 2300	Durand unpubl
Pla de Peyre	Creissels	12	PdPey	TEMP	44.08	3.05	RURAL	Neo	5500 - 5000	Bouby et al 2020b
Pech de Tardieu	Vinassan	11	PedTa	MEDIT	43.20	3.07	RURAL	LRo	1750 - 1300	Rovira, Tillier unpubl
Garenne (La)	Penne	81	PeGar	TEMP	44.07	1.73	CAVE	BA	3050 - 2750	Marinval unpubl
Peigros	Sainte-Maxime	83	Peigr	MEDIT	43.30	6.63	VILLAGE	IA1	2600 - 2500	Marinval unpubl
Madeleine I	Perpignan	66	PeMa1	MEDIT	42.68	2.88	RURAL	ERo	2150 - 2075	Ros, 2020
Pech Maho	Sigean	11	PeMah	MEDIT	43.03	2.98	VILLAGE	IA1	2550 - 2425	Rovira, Alonso 2017
Pech Maho	Sigean	11	PeMah	MEDIT	43.03	2.98	VILLAGE	IA2	2425 - 2175	Rovira, Alonso 2017
Mas Coste	Perpignan	66	PeMC	MEDIT	42.68	2.88	RURAL	ERo	2120 - 1940	Ros, 2020
Mas Delfau	Perpignan	66	PeMDe	MEDIT	42.68	2.88	RURAL	ERo	2100 - 1900	Ros, 2020
Petit Clos I	Perpignan	66	PePC1	MEDIT	42.68	2.88	RURAL	ERo	2020 - 1850	Ros, 2020
Pierredon	Eguilles	13	Pierr	MEDIT	43.57	5.37	VILLAGE	IA2	2225 - 2100	Marinval 1988
Plafète	Epagny	74	Plafet	TEMP	45.93	6.10	RURAL	BA	4200 - 2800	Flottes unpubl
Pla de Molas	Le Boulou	66	PlMol	MEDIT	42.52	2.83	RURAL	IA2	2150 - 2100	Ros, 2020
Plots (Les)	Berriac	11	Plots	MEDIT	43.22	2.42	RURAL	Neo	6500 - 6000	Marinval unpubl
Pont de Roque Haute	Portiragnes	34	PoPRH	MEDIT	43.30	3.33	RURAL	Neo	7750 - 7600	Marinval unpubl
Peiro Signado	Portiragnes	34	PoPS	MEDIT	43.30	3.33	RURAL	Neo	7750 - 7600	Marinval unpubl
Pré de la Cour	Montagnieu	01	PrdCo	TEMP	45.80	5.47	RURAL	BA	2900 - 2750	Marinval 1993c
Pré de la Cour	Montagnieu	01	PrdCo	TEMP	45.80	5.47	RURAL	IA1	2800 - 2700	Marinval 1993c
Rue de la Basse	Prades	66	PrRBa	MEDIT	42.62	2.43	RURAL	LRo	1750 - 1500	Ros unpubl
Prével Supérieur	Montclus	30	PrSup	MEDIT	44.27	4.43	CAVE	BA	3300 - 3200	Erroux, Courtin 1974
Rue du Partegal	La Farlède	83	Prtgl	MEDIT	43.17	6.03	RURAL	ERo	1950 - 1900	Rovira unpubl
Petites Bâties	Lamotte-du-Rhône	84	PtBat	MEDIT	44.27	4.68	RURAL	Neo	7500 - 7000	Figueiral unpubl
Petit Beaulieu/Puy Long	Clermont-Ferrand	63	PtBPL	TEMP	45.78	3.08	RURAL	BA	3900 - 3750	Durand unpubl
Petit Beaulieu/Puy Long - funerary	Clermont-Ferrand	63	PtBPLfu	TEMP	45.78	3.08	FUN	BA	3900 - 3750	Durand unpubl
Petit Garlambaud	Béziers	34	PtGrl	MEDIT	43.35	3.25	RURAL	BA	3350 - 3100	Figueiral unpubl
Plateau Raverre	Saint-Uze	26	PtRav	TEMP	45.18	4.87	RURAL	Neo	6500 - 6000	Martin et al 2016
Puig del Baja	Canet-en-Roussillon	66	PuBaj	MEDIT	42.71	3.01	RURAL	ERo	2100 - 1850	Ros, 2020
Cordouls (Les)	Puylaurens	81	PuyCor	TEMP	43.57	2.02	VILLAGE	BA	2900 - 2750	Durand, Marinval unpubl
Cordouls (Les)	Puylaurens	81	PuyCor	TEMP	43.57	2.02	VILLAGE	IA1	2700 - 2600	Durand unpubl
Cordouls (Les)	Puylaurens	81	PuyCor	TEMP	43.57	2.02	VILLAGE	IA2	2500 - 2400	Durand unpubl
Cordouls (Les)	Puylaurens	81	PuyCor	TEMP	43.57	2.02	VILLAGE	Rom	2025 - 1500	Durand unpubl
La Plaine	Puylaurens	81	PuyPla	TEMP	43.57	2.02	RURAL	IA1	2600 - 2500	Durand unpubl
La Plaine	Puylaurens	81	PuyPla	TEMP	43.57	2.02	RURAL	IA2	2150 - 2100	Durand unpubl
Portal Vielh	Vendres	34	PVV	MEDIT	43.27	3.22	RURAL	BA	3200 - 2750	Bouby et al 1999
Auribelle-Basse (L')	Pézenas	34	PzAuri	MEDIT	43.45	3.42	URBAN	ERo	1855 - 1740	Delbois 2017
Grange Rouge	Quincieux	69	QuGRo	TEMP	45.92	4.78	RURAL	BA	3250 - 2900	Cabanis unpubl
Grange Rouge	Quincieux	69	QuGRo	TEMP	45.92	4.78	RURAL	IA1	2700 - 2525	Cabanis unpubl
Quinquiris	Castelnaudary	11	Quinq	TEMP	43.32	1.95	RURAL	Neo	4830 - 4480	Durand unpubl
Quinquiris	Castelnaudary	11	Quinq	TEMP	43.32	1.95	FUN	BA	3650 - 3350	Durand unpubl
Quinquiris	Castelnaudary	11	Quinq	TEMP	43.32	1.95	RURAL	IA1	2525 - 2475	Durand unpubl
Acropole (Rue)	Villeneuve-lès-Béziers	34	RAcro	MEDIT	43.32	3.27	RURAL	ERo	2000 - 1750	Bouby 2014
Acropole (Rue)	Villeneuve-lès-Béziers	34	RAcro	MEDIT	43.32	3.27	RURAL	LRo	1750 - 1700	Bouby 2014
Acropole (Rue)	Villeneuve-lès-Béziers	34	RAcro	MEDIT	43.32	3.27	RURAL	BA	3250 - 2900	Bouby 2014
Rajal del Gorp	Millau	12	RajdG	TEMP	44.10	3.08	FUN	ERo	2000 - 1900	Demierre 2015
Rajal del Gorp	Millau	12	RajdG	TEMP	44.10	3.08	CAVE	IA2	2200 - 2000	Demierre 2015
Ravaner 1 (Le)	Argelès-sur-Mer	66	Rav1	MEDIT	42.55	3.02	RURAL	BA	2900 - 2750	Marinval unpubl
Pampelune (Roc de)	Argelliers	34	RcPamp	MEDIT	43.70	3.68	RURAL	LRo	1700 - 1500	Rovira unpubl
Roc Troué (Abri)	Sainte-Eulalie-de-Cernon	12	RcTro	TEMP	43.98	3.13	CAVE	Neo	7000 - 6500	Erroux 1992
RD20e	Marignane	13	RD20	MEDIT	43.42	5.22	RURAL	ERo	2000 - 1750	Flottes unpubl
Ro di Bolo	Sanilhac-Sagriès	30	RdBo	TEMP	43.95	4.42	CAVE	Neo	5500 - 4200	Marinval 2008
Reille (La)	Montbazin	34	Reill	MEDIT	43.52	3.68	RURAL	ERo	1950 - 1575	Rovira unpubl
Resclauza (La)	Gabian	34	Resclz	MEDIT	43.50	3.27	RURAL	Neo	7000 - 6500	Marinval unpubl
Revaute (La)	Lempdes-sur-Allagnon	43	Revaut	TEMP	45.39	3.27	RURAL	IA1	2650 - 2580	Cabanis unpubl
Toulons (Les)	Rians	83	RiToul	MEDIT	43.62	5.75	RURAL	ERo	1850 - 1800	Bouby 2014
Toulons (Les)	Rians	83	RiToul	MEDIT	43.62	5.75	RURAL	LRo	1750 - 1600	Bouby 2014
Rivaux (Les)	Espaly-Saint-Marcel	43	Rivox	TEMP	45.05	3.87	RURAL	Neo	6500 - 5500	Bouby 1993
Rochas	Grospierres	07	Rocha	MEDIT	44.40	4.30	RURAL	Neo	4800 - 4200	Marinval 1988
Roche Noire	Montagnieu	01	RoNo	TEMP	45.80	5.47	CAVE	IA1	2600 - 2525	Wiethold, Treffort 2002
Roquette (La)	Cavillargues	30	Roqt	MEDIT	44.12	4.52	RURAL	LRo	1625 - 1550	Bouby 2014
Roumèges	Poussan	34	Roumg	MEDIT	43.48	3.67	RURAL	ERo	1970 - 1875	Rovira unpubl
Roquemengarde	Saint-Pons-de-Mauchiens	34	RQMG	MEDIT	43.52	3.52	RURAL	Neo	5450 - 4550	Bouby et al 2020b
Roquepertuse	Velaux	13	RQP	MEDIT	43.52	5.27	VILLAGE	IA2	2450 - 2175	Bouby et al 2011a
Serre 1 (Le)	Roynac	26	RS1	TEMP	44.63	4.93	RURAL	Neo	7000 - 6500	Bouby unpubl
Serre 1 (Le)	Roynac	26	RS1	TEMP	44.63	4.93	RURAL	BA	4200 - 3900	Bouby unpubl
Taï (Le)	Remoulins	30	RTai	MEDIT	43.93	4.57	CAVE	Neo	7400 - 5200	Bouby et al 2019
Corbières (Rue des)	Elne	66	RuCor	MEDIT	42.60	2.97	VILLAGE	IA2	2400 - 2100	Bénézet et al 2014
Lagarel	Saint-André-de-Sangonis	34	SALag	MEDIT	43.65	3.50	RURAL	Neo	5000 - 4600	Bouby et al 2020b
Lesse (La)	Sauvian	34	SaLSS	MEDIT	43.30	3.27	RURAL	ERo	2025 - 1725	Figueiral et al 2015
Sauzet/A7-Section 2	Sauzet	26	SauzA7	TEMP	44.60	4.82	RURAL	ERo	1925 - 1900	Rovira unpubl
Cadenet	Sernhac	30	SeCad	MEDIT	43.92	4.55	RURAL	ERo	2000 - 1800	Bouby 2014
Serre de Boidon	Grospierres	07	SedBo	MEDIT	44.40	4.30	RURAL	Neo	4800 - 4200	Marinval 1988
Gandus (Les)	Saint-Ferréol-Trente-Pas	26	SFTPGa	MEDIT	44.43	5.22	VILLAGE	BA	2900 - 2750	Marinval1988
Sizen-Vigne	Beaucaire	30	SizVig	MEDIT	43.80	4.63	FUN	IA2	2400 - 2200	Tillier, Rovira 2016
Saint Martin d'Azirou	La Vacquerie-St-Martin-de-Castries	34	SMAz	TEMP	43.78	3.45	FUN	Neo	5500 - 4600	Ruas unpubl
Saint Martin III	Chabrillan	26	SMT3	TEMP	44.72	4.93	RURAL	BA	4200 - 3650	Bouby 2000
Mourre de Sève	Sorgues	84	SoMDS	MEDIT	44.00	4.87	VILLAGE	IA1	2550 - 2500	Pinaud 2015
Moulins (Les)	Saint-Paul-Trois-Châteaux	26	SP3CMo	MEDIT	44.35	4.77	RURAL	Neo	6000 - 5500	Martin et al 2016
Valladas (Le)	Saint-Paul-Trois-Châteaux	26	SP3CVal	MEDIT	44.35	4.77	RURAL	Neo	7500 - 7000	Beeching et al 2000
Valladas (Le)	Saint-Paul-Trois-Châteaux	26	SP3CVal	MEDIT	44.35	4.77	FUN	ERo	1975 - 1800	Marinval 1993a
Saint Romain de Jalionas	Saint-Romain-de-Jalionas	38	SRJ	TEMP	45.75	5.22	RURAL	ERo	2000 - 1770	Bouby 2014
Saint Romain de Jalionas -T57	Saint-Romain-de-Jalionas	38	SRJ	TEMP	45.75	5.22	OFFSITE	ERo	2000 - 1770	Bouby 2014
Saint Romain de Jalionas	Saint-Romain-de-Jalionas	38	SRJ	TEMP	45.75	5.22	RURAL	LRo	1800 - 1600	Bouby 2014
Rue de la Corderie	Saint-Romain-en-Gal	69	SRRco	TEMP	45.53	4.87	URBAN	Rom	2025 - 1500	Flottes unpubl
Saint Bézard	Aspiran	34	StBez	MEDIT	43.57	3.43	RURAL	ERo	1925 - 1875	Mauné et al 2013
Saint Etienne de Dions	Saint-Marcel-d'Ardèche	07	StEDi	MEDIT	44.32	4.62	VILLAGE	IA1/2	2525 - 2475	Bouby 2014
Saint Etienne de Dions	Saint-Marcel-d'Ardèche	07	StEDi	MEDIT	44.32	4.62	VILLAGE	IA2	2325 - 2275	Bouby 2014
Saint Pierre	Martigues	13	StPie	MEDIT	43.40	5.05	VILLAGE	ERo	2020 - 1800	Chausserie Laprée 2005
Saint Pierre	Martigues	13	StPie	MEDIT	43.40	5.05	VILLAGE	IA1/2	2550 - 2475	Chausserie Laprée 2005
Sables (Les)	Théziers	30	ThSab	MEDIT	43.90	4.62	RURAL	IA1	2750 - 2625	Bouby 2014
Garennes (Les)	Tourbes	34	ToGar	MEDIT	43.45	3.38	RURAL	IA1	2550 - 2550	Figueiral unpubl
Riaux (Les)	Toulon	83	ToRiax	MEDIT	43.12	5.93	URBAN	ERo	1950 - 1700	Bouby 2014
Touar (Le)	Les Arcs	83	Touar	MEDIT	43.45	6.48	RURAL	BA	3250 - 2900	Berato et al 1989
Touar (Le)	Les Arcs	83	Touar	MEDIT	43.45	6.48	RURAL	IA1	2650 - 2500	Berato et al 1989
Baume Layrou	Trèves	30	TrBlay	TEMP	44.08	3.39	CAVE	BA	3200 - 3050	Bouby et al. 2005
Tremaïe	Arles	13	Trema	MEDIT	43.67	4.63	URBAN	IA2	2125 - 2100	Marinval 1988
Teste Nègre	Les Pennes-Mirabeau	13	TsNeg	MEDIT	43.41	5.32	VILLAGE	IA2	2300 - 2100	Marinval 1988
Val du Fou	Martigues	13	VaFou	MEDIT	43.40	5.05	RURAL	IA1	2650 - 2500	Bouby unpubl
Vautes (Les)	Saint-Gély-du-Fesc	34	Vaute	MEDIT	43.70	3.80	RURAL	Neo	4700 - 4200	Buxo 2003a

Site	Municipality	Dep.	CodeSite	Bioclim	latitude	longitude	type	Period	DATE BP	Reference
Vayssière	L'Hospitalet-du-Larzac	12	Vayss	TEMP	43.97	3.18	FUN	ERo	2000 - 1800	Marinval 1993a
Clapiers	Villeneuve-lès-Béziers	34	VBzCl	MEDIT	43.32	3.27	FUN	ERo	2025 - 1750	Marinval 2004
Ceron	Venasque	84	VeCero	MEDIT	43.98	5.15	RURAL	Neo	7000 - 6500	Bouby unpubl
Arriasse	Vic-le-Fesq	30	VFArs	MEDIT	43.87	4.08	RURAL	IA1	2700 - 2625	Marinval 1985
Vignes de l'Espérance	Banyuls-dels-Aspres	66	ViEsp	MEDIT	42.57	2.87	RURAL	IA2	2500 - 2300	Figueiral et al 2015
Villa Giribaldi	Nice	06	ViGir	MEDIT	43.70	7.25	RURAL	Neo	6500 - 6000	Martin et al 2016
Villelongue	Castelnaudary	11	Villg	TEMP	43.32	1.95	RURAL	ERo	2025 - 1975	Figueiral unpubl
Villelongue	Castelnaudary	11	Villg	TEMP	43.32	1.95	RURAL	IA2	2125 - 2025	Figueiral unpubl
Ambrussum	Villetelle	34	VlAmb	MEDIT	43.73	4.15	URBAN	ERo	2025 - 1750	Ruas 1989b, Rovira 2012b
Ambrussum	Villetelle	34	VlAmb	MEDIT	43.73	4.15	URBAN	LRo	1750 - 1600	Rovira 2012b
Ambrussum - funerary	Villetelle	34	VlAmbF	MEDIT	43.73	4.15	FUN	ERo	2015 - 1975	Fiches et al 2007
Ambrussum - funerary	Villetelle	34	VlAmbF	MEDIT	43.73	4.15	FUN	IA2	2300 - 2030	Rovira 2012a, Fiches et al 2007
Corent	Veyre-Monton	63	VMCor	TEMP	45.68	3.17	RURAL	ERo	2050 - 1700	Flottes 2015
Corent	Veyre-Monton	63	VMCor	TEMP	45.68	3.17	VILLAGE	BA	2950 - 2800	Flottes 2015
Corent	Veyre-Monton	63	VMCor	TEMP	45.68	3.17	RURAL	IA1	2550 - 2510	Flottes 2015
Corent	Veyre-Monton	63	VMCor	TEMP	45.68	3.17	VILLAGE	IA2	2140 - 2050	Flottes 2015
Pirou (Le)	Valros	34	VPiro	MEDIT	43.42	3.37	RURAL	BA	4200 - 3600	Rousselet unpubl
Mas Sauvy	Villeneuve-de-la-Raho	66	VRaMS	MEDIT	42.63	2.92	RURAL	ERo	1940 - 1880	Ros and Ruas 2017
Rec de Ligno	Valros	34	VRcLi	MEDIT	43.42	3.37	RURAL	ERo	2025 - 1750	Jung, Bel 2017
Rec de Ligno	Valros	34	VRcLi	MEDIT	43.42	3.37	FUN	ERo	1950 - 1950	Jung, Bel 2017
Renaussas	Valros	34	VRena	MEDIT	43.42	3.37	FUN	ERo	2000 - 1700	Figueiral unpubl
Roquessols	Valros	34	VRoq	MEDIT	43.42	3.37	RURAL	Neo	5500 - 4200	Figueiral unpubl
Vieux-Mounoï (Grotte)	Signes	83	VxMou	MEDIT	43.30	5.87	CAVE	Neo	7500 - 5500	Marinval unpubl
Vieux-Mounoï (Grotte)	Signes	83	VxMou	MEDIT	43.30	5.87	CAVE	BA	3350 - 2800	Marinval unpubl
Zac de la Burlière	Trets	13	ZACBrl	MEDIT	43.45	5.68	RURAL	Neo	6500 - 5500	Figueiral unpubl
Saint Aunès (ZAC)	Saint-Aunès	34	ZACSA	MEDIT	43.63	3.97	RURAL	Neo	6500 - 5500	Martin et al 2016
ZAC de Sagnon	Graveson	13	ZACSag	MEDIT	43.85	4.77	FUN	ERo	1970 - 1830	Rovira unpubl
Feuilly (ZAC)	Saint-Priest	69	ZAFeuil	TEMP	45.69	4.93	RURAL	Neo	7300 - 6500	Zwierzinsky unpubl
Feuilly (ZAC)	Saint-Priest	69	ZAFeuil	TEMP	45.69	4.93	RURAL	BA	3200 - 2900	Bouby 2014
Feuilly (ZAC)	Saint-Priest	69	ZAFeuil	TEMP	45.69	4.93	RURAL	IA1/2	2525 - 2400	Bouby 2014
Feuilly (ZAC)	Saint-Priest	69	ZAFeuil	TEMP	45.69	4.93	RURAL	IA2	2425 - 2375	Bouby 2014

Codes

**Bioclim**	**Bio-climatic conditions**
MEDIT	Mediterranean
TEMP	Temperate

**Preserv**	**Preservation**
CHAR	Charred
UNCHAR	Uncharred

**Site tpe**	
CAVE	Cave and Rock-shelter occupations
FUN/CERE	Funerary and ceremonial sites
OFFSITE	Offsite records near human settlements
RURAL	Farms, hamlets and rural occupations
SM-AGGLO	Villages, small agglomerations
URBAN	CITIES

**Periods**	
1-Neo	Neolithic
2-BA	Bronze Age
3-IA1	First Iron Age
4-TrIA1/2	Transition First/Second Iron Age
5-IA2	Second Iron Age
6-Ero	Early Roman Empire
7-Lro	Late Roman Empire

DATE BP Date Before Present interval

References

Alonso N., Buxó R., Rovira N. (2007). Recherches sur l'alimentation végétale et l'agriculture du site de Lattes-Port Ariane : étude des semences et fruits. Lattara, 20 : 219-249.

Alonso, N., Rovira N., 2010. Consommation et traitement des produits végétaux à Lattara entre -475 et -350. Lattara, 21: 329-388.

Alonso, N., Rovira, N., 2016. Plant uses and storage in the 5th century bc Etruscan quarter of the city of Lattara, France. Vegetation History and Archaeobotany, 25: 323-337.

Bacou J.-P., Bacou A. (1983). L'Oppidum de Montfau à Magalas, Hérault. 1963-1979. Archéologie en Languedoc, 5 : 61-114.

Beeching A, Berger J-F, Brochier J-L et al. (2000). Chasséens: agriculteurs ou éleveurs, sédentaires ou nomades? Quels types de milieux, d'économies et de sociétés? In: Leduc M, Valdeyron N, Vaquer J (eds.), Rencontres méridionales de Préhistoire récente. Troisième session (Toulouse, 1998). Toulouse, Archives d'Ecologie Préhistorique : 59–79.

Bel, V. (2017). Deux ensembles funéraires d'époque romaine, Avenue Jean-Jaurès à Nîmes (Gard). Supplément 46. Revue archéologique de Narbonnaise, Montpellier.

Bénézet J., avec la collaboration de Hallavant Ch., Bouby L., Machado Yanes C. (2014). Le fossé de la rue des Corbières et le système de défense d'Elne (Pyrénées-Orientales), durant le deuxième âge du Fer (IVe-IIe s. av. n. è.). Documents d'Archéologie Méridionale, 35 : 253-284.

Bérato J., Magnin F., Bérato N., Brien F., Columeau P., Dugas F., Galliano G., Imbert L., Marinval P., Thinon M. (1989). Le Touar, les Arcs-sur-Argens. Un habitat de plaine du Bronze Final II/IIIa et du premier Age du Fer dans son environnement. Documents d'Archéologie Méridionale, 12 : 7-40.

Binder D., Battentier J., Bouby L, Brochier J.E., Carré A., Cucchi T. et al. (2020). First farming in the North-Western Mediterranean: evidence from Castellar – Pendimoun during the 6th mill. BCE. In: Gron K., Sorensen L., Rowley Conwy P. (dir.). Farmers at the Frontier: A Pan European Perspective on Neolithisation. Oxbow books, Oxford & Philadelphia : 145-159.

Binder D., Brochier J.-E., Duday H., Helmer D., Marinval P. Thiebault S., Wattez J. (1993). L'abri Pendimoun à Castellar (Alpes-Maritimes) : nouvelles données sur le complexe culturel de la céramique imprimée méditerranéenne dans son contexte stratigraphique. Gallia Préhistoire, 35 : 177-251.

Bouby L. (1993). Apports de la carpologie à la connaissance de l'économie végétale de l'Auvergne : du Mésolithique à l'époque gallo-romaine. Master's thesis, Université Montpellier

Bouby L. (2000). Agriculture et cueillette à l'âge du Bronze ancien dans la vallée du Rhône et en basse Auvergne. In : Leduc M., Valdeyron N., Vaquer J. (eds.). Sociétés et espaces. Archives d'Ecologie Préhistorique, Toulouse : 201-210.

Bouby L. (2009). Les restes carpologiques des couches 60 à 47. In : Voruz J.-L. (ed.). La grotte du Gardon (Ain) – Volume 1. Le site et la séquence néolithique des couches 60 à 47. Archives d’Ecologie Préhistorique, Toulouse : 227-230.

Bouby L. (2013). Les carporestes. In : Ayala G. (ed.). Lyon, Saint-Georges. Archéologie, environnement et histoire d’un espace fluvial en bord de Saône. Documents d’Archéologie Française, 106. Editions de la Maison des Sciences de l’Homme, Paris : 369-387.

Bouby L. (2014). L’agriculture dans le Bassin du Rhône du Bronze final à l’Antiquité. Agrobiodiversité, économie, cultures. Archives d’Ecologie Préhistorique, Toulouse, 335 p.

Bouby L., Billaud Y. (2001). Economie agraire à la fin de l'âge du Bronze sur les bords du lac du Bourget (Savoie, France). Comptes Rendus de l'Académie des Sciences, Paris, Sciences de la Terre et des planètes, 333 : 749-756.

Bouby L., Boissinot P., Marinval P. (2011a). Never mind the bottle. Archaeobotanical evidence of beer-brewing in Mediterranean France and the consumption of alcoholic beverages during the 5th century BC. Human Ecology, 39, 3: 351-360.

Bouby L., Durand F., Rousselet O., Manen C. (2019). Early farming economy in Mediterranean France: fruit and seed remains from the Early to Late Neolithic levels of the site of Taï (ca 5300–3500 cal bc). Vegetation History and Archaeobotany, 28, 1: 17-34.

Bouby L., Fages G., Treffort J.-M. (2005). Food storage in two Late Bronze Age caves of Southern France: palaeoethnobotanical and social implications. Vegetation History and Archaeobotany, 14, 4: 313-328.

Bouby L., Figueiral I. (2014). Les ressources végétales du Néolithique ancien nîmois : Mas de Vignoles X et Mas Neuf. In : Perrin T., Manen C., Séjalon P. (dir.). Le Néolithique ancien de la plaine de Nîmes (Gard, France). Archives d’Ecologie Préhistorique, Toulouse : 339-343.

Bouby L., Figueiral I., Schaal C. (2011b). Graines, fruits bois et charbons d’un espace horticole. In : Excoffon P. (ed.). Ville et campagne de Fréjus romaine. La fouille préventive de « Villa Romana ». Bibliothèque d'Archéologie Méditerranéenne et Africaine, 8. Errance/Centre Camille Julian, Paris/Aix-en-Provence : 221-236.

Bouby L., Léa V. (2006). Exploitation de la vesce commune (Vicia sativa L.) au Néolithique moyen dans le Sud de la France. Données carpologiques du site de Claparouse (Lagnes, Vaucluse). Comptes Rendus Palevol, 5 : 973-980.

Bouby L., Leroy F., Carozza L. (1999). Food plants from late Bronze Age lagoon sites in Languedoc, southern France: reconstruction of farming economy and environment. Vegetation History and Archaeobotany, 8 : 53-69.

Bouby L., Marinval P., Durand F. et al. (2020a). Early Neolithic (ca. 5850-4500 cal BC) agricultural diffusion in the Western Mediterranean: An update of archaeobotanical data in SW France. PLoS ONE, 15 (4): e0230731. https://doi.org/10.1371/journal. pone.0230731

Bouby L., Marinval P., Rovira N. (2020b). Late Neolithic plant subsistence and farming activities on the southern margins of the Massif Central (France). The Holocene, https://doi.org/10.1177/0959683619895576

Bouby L., Ponel P., Girard V., Chen C.T., Garnier L., Tillier M., Devillers B., Lachenal T., Tourrette C., Gascó J. (2016). Premiers résultats carpologiques et entomologiques sur le site subaquatique Bronze final de la Motte (Agde, Hérault). Aquitania, Suppl. 36 : 65-87.

Bouchette, A. (1998). Les données carpologiques. In: Clavel-Lévêque, M., Vignot, A. (eds) Actes du Colloque européen, Béziers octobre 1997. Cité et territoire II, Presses Universitaires Franc-Comtoises. Les Belles Lettres, Paris, pp 106–107.

Bouchette, A., Rovira, N., Figueiral, I. (2017). Des plantes et des fruits importés et introduits à Nîmes (Gard, France) à l'époque gallo-romaine. In: Rovira, N., Bouby, L., Bouchette, A., Ruas, M.-P. (eds.), Plantes, produits et pratiques : diffusion et adoption de la nouveauté dans les sociétés préindustrielles. Monographies d'Archéologie Méditerranéenne, Hors Série 8: 85-118.

Buffat L., Guerre J., Masbernat-Buffat A., Renaud A., Rovira N., Piques G., Gardeisen A., Cantuel J., Garcia L., Longepierre S., Porcier S., Payan G. (2009). La villa de La Gramière (Castillon-du-Gard). Premier bilan de la recherche. Revue Archéologique de Narbonnaise, 42 : 115-216.

Buisson-Catil J., Sauzade G., Courtaud A., Hasler A., Puig J.-M., Thiébault S. (1997). Le gisement Bronze final de l'aven des Fourches I à Sault. Contribution à l'étude fonctionnelle des cavités karstiques des plateaux du Vaucluse. Documents d'Archéologie Méridionale, 19-20 : 7-31.

Buxó, R. (1992). Cueillette et agriculture à Lattes : les ressources végétales d'après les semences et les fruits. Lattara, 5: 45-90.

Buxo I Capdevila R. (1993). Des semences et des fruits. Cueillette et agriculture en France et en Espagne Méditerranéennes du Néolithique à l'âge du Fer. Unpublished PhD. Université de Montpellier 2, Montpellier, 2 vol., 633 p.

Buxó, R. (1996). Evidence for vines and ancient cultivation from an urban area, Lattes (Hérault), Southern France. Antiquity, 70: 393-407.

Buxó, R. (1999). Première approche des plantes exploitées au IVe siècle avant notre ère à Lattes. Lattara, 12: 525-535.

Buxo I Capdevila R. (2003a). Environnement, économie et population du site des Vautes. Approche carpologique des échantillons issus de la fouille. In : Guilaine J., Escalon G. (eds), Les Vautes (Saint-Gély du Fesc, Hérault) et la fin du Néolithique en Languedoc oriental. Archives d'Ecologie Préhistorique, Toulouse: 145-146.

Buxó, R. (2003b). Etude carpologique de la place 123 : problématique de la présence de restes de semences et de fruits dans un espace urbain non construit. Lattara, 16: 193-217.

Buxó, R. (2005). Étude carpologique des puits de Lattes. Évaluation et comparaison avec l'habitat. Lattara, 18 : 199-219.

Buxo R. (2006). Les restes des semences et fruits archéologiques de la grotte de Montou. Etudes Rousillonnaises, 22 : 33–42.

Cabanis M., Bouby L. (2016). Environnement exploitation des ressources végétales Un habitat en Auvergne. Champ Madame à Beaumont (Puy-de-Dôme) au cours du Néolithique Moyen II. In: Saintot S. (ed.), Un habitat en Auvergne. Champ Madame à Beaumont (Puy-de-Dôme) au cours du Néolithique Moyen II. Collection "Recherches Archéologiques", 11. CNRS/INRAP, Paris : 424-432.

Cabanis M., Durand E., Bouby L., Durand F. (2021). Agriculture et cueillette en Ardèche du Bronze moyen à la fin des âges du Fer (1550-50 BC) : premiers indices carpologiques. Ardèche Archéologie n°38 : 57-67.

Cabanis M., Mennessier-Jouannet C., Bouby L., Hajnalovà M., Wiethold J. (2010). Economie végétale en basse Auvergne à l'âge du Bronze et au premier âge du Fer. In : Delhon C., Théry-Parisot I., Thiébault S. (eds.), Des hommes et des plantes – Exploitation du milieu et gestion des ressources végétales de la préhistoire à nos jours. Actes des XXXe rencontres internationales d'Archéologie et d'histoire d'Antibes. ADPDCA, Antibes: 67-84.

Carozza L., Bouby L. avec la collaboration de Ballut C. (2006). Un habitat du Bronze moyen à Cournon-d'Auvergne (Puy-de-Dôme) : nouvelles données sur la dynamique de l'Âge du Bronze moyen sur la bordure méridionale du Massif central. Bulletin de la Société préhistorique française, 103, 3 : 535-584.

Carozza L, Vialet P, Bouchette A et al. (1999). L'habitat de plaine Néolithique final du Bousquetas à Paulhan (Hérault). Bulletin de la Société Préhistorique Française, 96 (1): 39–51.

Chabot L. (1972). Le Castellas de Rognac et l'Etang de Berre à l'époque préromaine.

Chausserie-Laprée J. (2005). Martigues,terre gauloise. Entre Celtique et Méditerranée. Errance, Paris, 251 p.

Collis J.R., Périchon R., Chopelin C., Murphy P. (1979). Etudes de céréales sur le site protohistorique d'Aulnat. Revue Archéologique du Centre de la France, 18, 1-2 : 35-38.
Convertini, F. Georjon C., 2018. Le Champ du Poste (Carcassonne, Aude). Une succession d'occupations du début du Néolithique moyen à l'âge du Bronze ancien. Archives d'Écologie Préhistorique, Toulouse.

Convertini F. Georjon C. (2018). Le Champ du Poste (Carcassonne, Aude). Une succession d'occupations du début du Néolithique moyen à l'âge du Bronze ancien. Archives d'Écologie Préhistorique, Toulouse, 500 p.

Courtin J., Erroux J., Thommeret J. (1976). Les céréales du Néolithique ancien de Châteauneuf-lès-Martigues (Bouches-du-Rhône). *Bull. du Musée d'Hist. Nat. De Marseille* 36 : 11-15.

Cousseran-Néré S., Néré E., Cabanis M., Lalai D. Mougin E., Notier F. (2020). Déjeuner au bord du Lac : Que mangeait-on à Chens-sur-Léman 1000 ans avant notre ère. Bulletin de l'APRAB, 2: 194-201

Dartevelle H., Alix P., Bouby L., Fontana L., Surmely F. (2004). Opme Le Cimetière à Romagnat (Puy-de-Dôme). In : Dartevelle H. (ed.). 5èmes rencontres Méridionales de Préhistoire Récente. Auvergne et Midi, actualité de la recherche. Actes de la cinquième session, Clermont-Ferrand (Puy-de-Dôme), 8 et 9 novembre 2002. Préhistoire du Sud-Ouest, Supplément N°9, Cressensac : 133-151.

De Labriffe, P.-A., Durand, F., Forest, V. et al., 2016. Bram « La Gabache » entre XVe et XIIIe siècles av. n. -è. en Lauragais (Aude). In: Cauliez, J, Sénépart, I, Jallo,t L, et al., (eds.), De la tombe au territoire et actualité de la recherche. Rencontres méridionales de Préhistoire récentes (Montpellier, 2014). Archives d'Ecologie Préhistorique, Toulouse : 603–616.

Delbois, E., 2016. Les offrandes végétales gallo-romaines en contexte funéraire : étude carpologique de la nécropole de La Closeraie (Orange, Vaucluse). Unpublished Master 1 dissertation, university Paul Valéry-Montpellier 3, 1 volume.

Delbois, E., 2017. Etude carpologique du puits gallo-romain de l'Auribelle-Basse (Pézénas, Hérault). Unpublished Master 2 dissertation, university Paul Valéry-Montpellier 3, 1 volume.

Demierre, M (2015). La grotte sanctuaire rutène du Rajal del Gorp. Bilan des fouilles récentes et particularités. In: Olmer, F., Roure, R. (eds.), Les Gaulois au fil de l'eau. Colloque international de l'AFEAF, (Montpellier, 2013). Volume 1. Ausonius Éditions, Bordeaux: 697–718.

Erroux J. (1966). Les orges hallstattiennes du Lycée Technique de Montpellier. O.G.A.M., 18, 5-6 : 455-456.

Erroux J. (1976). Les débuts de l'agriculture en France : les céréales. In : Guilaine J. (ed.), La Préhistoire Française, Vol. 2. CNRS, Paris : 186-191.

Erroux J. (1979a). Détermination de graines carbonisées. In : Roudil J.L., Roudil O., Soulier M. (eds.), La grotte de l'Aigle à Mejannes-le-Clap (Gard) et le Néolithique ancien du Languedoc oriental. Mémoires de la Société Languedocienne de Préhistoire, Montpellier 1 : 75.

Erroux J. (1979b). Etudes de graines de céréales de l'Aven des Corneilles. In : Fages G. (ed.), L'aven des Corneilles, Prades (Lozère). ARALO 7 : 59-61.

Erroux J. (1980). Etude des vestiges paléobotaniques (plantes cultivées et pépins de raisin). In : Dedet B. (dir.), Premières recherches sur l'Oppidum du Plan de la Tour à Gailhan (Gard). Sondages 1975-1977. Caveirac : 117-122.

Erroux J. (1981). Etude des graines des sites préhistoriques des Causses : La Poujade, St-Etienne-de-Gourgas, Pompignan. Paléobiologie Continentale, 12, 1: 273-278.

Erroux J. (1984). Etude de quelques graines de la Liquière. In: Py M. (ed.), La Liquière (Calvisson, Gard), village du Premier âge du Fer en Languedoc oriental. Revue Archéologique de Narbonnaise, Suppl. 11. CNRS, Paris : 349-350.

Erroux J., Poulain T. (1984). Faune et céréales de la grotte 1 de Sargel à Saint-Rome-de-Cernon (Aveyron). Gallia Préhistoire 27, 1 : 211-228.

Erroux J. (1986). Les céréales cultivées du site de Carsac. In: J. Guilaine (ed.), Une agglomération protohistorique en Languedoc. Toulouse, Centre d'Anthropologie des Sociétés Rurales : 215-217.

Erroux J. (1988). Etude des grains, fruits et graines de la grotte de Saint-Marcel (Ardèche). In : Gilles R., Le Néolithique et l'Age du Bronze à la grotte de Saint-Marcel (Ardèche). Ardèche Archéologie, 5 : 42-45.

Erroux J. (1992). Diagnose de quelques débris de végétaux de l'abri du Roc Troué (Sainte-Eulalie-de-Cernon, Aveyron). Bulletin de la Société Préhistorique Française, 89, 7 : 218-219.

Erroux J. (1993). Les céréales carbonisées. Documents d'Archéologie Méridionale 16 : 157-158.

Erroux J., Courtin J. (1974). Aperçu sur l'agriculture préhistorique dans le sud-est de la France. Bulletin de la Société Languedocienne de Géographie, 8, 3-4 : 325-336.

Erroux J., Poulain TL (1984). II. Faune et céréales de la grotte 1 de Sargel à Saint-Rome-de-Cernon (Aveyron). Gallia Préhistoire, 21-1 : 211-228.

Excoffon P., Devillers B., avec la collaboration de Bouby L., Bonnet S. (2006). Nouvelles données sur la position du littoral antique de Fréjus. Le diagnostic archéologique du « théâtre d'agglomération » (Fréjus, Var). ArchéoSciences, 30 : 205-221.

Fabre, L., Figueiral, I., Tardy, C., et al. (2009). Le puits de la villa de Montferrier à Tourbes (Hérault) : des données paléoenvironnementales pour un essai de restitution d'une exploitation antique. Territori i els seu recursos. The territory and its resources. Estudis sobre el mòn rural d'època romana, 4: 73-83.

Fages G. (1979). L'Aven des Corneilles (Prades, Lozère). Cah. ARALO, 7), 106 p.

Fiches, J.-L., Barberan, S., Berdeaux-Le Brazidec, M.-L., Chabal, L., Gafa, R., Gardeisen, A., Garcia, L., Gazenbeek, M., Mathieu, V., Rovira, N., Chevalier, J., Conterio, M., Richard, J.-C., Tosna, D. (2007). Un enclos cultuel sur la berge du Vidourle à Ambrussum (Viletelle, Hérault). Revue Archéologique de Narbonnaise, 40: 47-116.

Figueiral I., Bouby L., Buffat L., Petitot H., Terral J.-F. (2010). Archaeobotany, vine growing and wine producing in Roman Southern France: Le Gasquinoy (Béziers, Hérault). Journal of Archaeological Science, 37, 1 : 139-149.

Figueiral I., Bouby L., Chabal L., Hallavant C., Machado C., Ros J., Ruas M.-P., Schaal C., Zech-Matterne V. (2015). Données archéobotaniques sur les établissements ruraux du second âge du Fer en Languedoc-Roussillon. Documents d'Archéologie Méridionale, 36: 285-294.

Figueiral I., Bouby L., Buffat L., Petitot H., Terral J.-F. (2010). Archaeobotany, vine growing and wine producing in Roman Southern France: Le Gasquinoy (Béziers, Hérault). Journal of Archaeological Science 37, 1: 139-149.

Figueiral I., Pomarèdes H., Court Picon M., Bouby L., Tardy C., Terral J.-F. (2015b). New insights into Mediterranean Gallo-Roman farming: a closer look at archaeological wells in Southern France. Archaeological Anthropological Sciences, 7 : 201-233.

Figueiral I., Séjalon P. (2014). Archaeological wells in southern France: Late Neolithic to Roman plant remains from Mas de Vignoles IX (Gard) and their implications for the study of settlement, economy and environment. Environmental Archaeology, 19, 1: 23-38.

Flottes L. (2015). Étude carpologique des occupations de l'âge du Bronze final à l'Époque romaine sur le site de Corent (Puy-de-Dôme). Mémoire de Master 2, parcours Quaternaire et Préhistoire, Muséum national d'Histoire naturelle, Paris, 94 p.

Garcia D., Orliac D., Marinval P., Pernaud-Orliac J. (1990). Les Courtinals à Mourèze (Hérault). Etude de l'habitat protohistorique et de son territoire. Documents d'Archéologie Méridionale, 13: 15-34.

Guilaine J., Hopf M. (1984) Vestiges d'agriculture et céramiques protohistoriques de la Grotte de Buffens (Caunes-Minervois, Aude). In: Eléments de pré et protohistoire européenne. Hommages à J.-P. Millotte. Annales Littéraires de l'Université de Besançon, Les Belles Lettres, Paris : 629-638.

Guilaine J., Hopf M., Bouby L. (2016). Un ensemble de carporestes protohistoriques découvert à Coumo dal Cat à Ladern (Aude). In: Chazelles C.A. et Schwaller M. (eds.). Vie quotidienne, tombes et symboles des sociétés protohistoriques de Méditerranée nord-occidentale. Mélanges offerts à Bernard Dedet, Vol. 1. Lattes, Association pour le Développement de l'Archéologie en Languedoc-Roussillon, Monographies d'Archéologie Méditerranéenne, Hors série 7 : 103-116.

Hajnalovà M. (2006). Chapitre 6. Études des macro-restes végétaux. In : Deberge Y., Collis J., Dunkley J. (eds), Le Pâtural, Clermont-Ferrand, Puy-de-Dôme. Un établissement agricole gaulois en limagne d'Auvergne. DARA, 30. Alpara, Lyon: 207-226.

Jacquot K. (1994). Paléoécologie et paléoéconomie d'un site de l'âge du Bronze : étude des paléosemences de Chindrieux, Châtillon (Lac du Bourget, Savoie). Unpublished Master thesis, Université de Franche-Comté, Besançon, 2 vol., 120 p.

Jung, C., Bel, V. (2017). Un espace rural antique dans le territoire de la cité de Béziers. Supplément 45. Revue archéologique de Narbonnaise, Montpellier.

Lelouvier, L.-A, Durand, F., Martin, H., et al. (2020). Un établissement rural antique à Massac-Séran? Archéologie tarnaise, 21: 55-65.

Le Roy, L., Berdaux-Le Brazidec, M.-L., Malignas, A., Rovira, N. (2011). De la villa antique à l'établissement alto-médiéval de la Maladrerie à Saillans (Drôme). Permanences et mutations jusqu'au VIIIe siècle. Archéologie du Midi Médiéval, 29: 3-42.

Lundström-Baudais K. (1991). Les macrorestes végétaux de deux puits des IIe-IIIe siècles à Annecy-le-Vieux, Les Ilettes. In: Vivian R. (ed.), Paléoenvironnement Holocène et Archéologie dans les Alpes du Nord et leur piémont. Comité des Travaux Historiques et Scientifiques, Paris : 109-113.

Manen C., Vigne J.-D., Loirat D., Bouby L. (2001). L'Aspre del Paradis à Corneilla-del-Vercol (Pyrénées-Orientales) : contribution à l'étude du Néolithique ancien et final. Bulletin de la Société Préhistorique Française, 98, 3 : 505-528.

Marinval P. (1985). Etude des paléo-semences de deux fosses-silos du Premier Age du fer à L'Arriasse, Vic-le-Fesq (Gard). Documents d'Archéologie Méridionale, 8 : 147-150.

Marinval P. (1986). Analyse paléocarpologique : Baume des Anges, Donzère (Drôme). Age du Bronze final. Bulletin de la Société Préhistorique Française, 83 (1) : 30.

Marinval P. (1988). Cueillette, agriculture et alimentation végétale de l'Epipaléolithique jusqu'au 2ème âge du Fer en France méridionale. Apports palethnographiques de la carpologie. Unpublished PhD thesis, EHESS, Paris, 2 vol., 458 p.

Marinval P. (1993a). Etude carpologique d'offrandes alimentaires végétales dans les sépultures gallo-romaines : réflexions préliminaires. In: Ferdière A. (ed.), Monde des morts, monde des vivants en Gaule rurale. Actes du colloque ARCHEA/AGER (Orléans, Conseil Régional, 7-9 février 1992). 6ème supplément à la Revue Archéologique du Centre de la France, Tours : 45-65.

Marinval P. (1993b). Analyse carpologique du Roc de Dourgne. In: Guilaine J. (dir.). Dourgne. Derniers chasseurs-collecteurs et premiers éleveurs da la haute-vallée de l'Aude. CASR, Toulouse, ARETA, Carcassonne : 415-416.

Marinval P. (1993c). Analyse des paléo-semences. In : Vital J. (ed.). Habitats et sociétés du Bronze final au Premier Age du Fer dans le Jura. Les occupations protohistoriques et néolithiques du Pré de la Cour à Montagnieu (Ain). Monographie du CRA, 11. CNRS, Paris : 50-52.

Marinval P. (2003). L'ensemble carpologique du site bizien de la Salle (Carcassonne, Aude). Bulletin de la Société Préhistorique Française, 100 (2) : 353-355.

Marinval P. (2004). Offrandes alimentaires d'origine végétale en contexte funéraire gallo-romain. Nouveau regard. In: Baray L. (ed.). Archéologie des pratiques funéraires. Approches critiques. Actes de la table ronde des 7 et 9 juin 2001 (Glux-en-Glenne, Bibracte). Centre archéologique européen, Glux-en-Glenne : 197-206.

Marinval P. (2007). Premières données sur l'économie végétale. In: Guilaine J., Manen C., Vigne J.D. (eds.). Pont de Roque Haute. Nouveaux regards sur la néolithisation de la France méditerranéenne. Toulouse, Archives d'Ecologie Préhistorique : 215–217.

Marinval, P. (2008). Analyse carpologique. In: Coularou J., Jallet F., Colomer A., Balbure J. (eds.), Boussargues. Une enceinte chalcolithique des garrigues du Sud de la France. Archives d'Ecologie Préhistorique, Toulouse: 181-198.

Martin L. (2003). Paléosemences et autres restes végétaux : rapport préliminaire. In : Nicod P.-Y., Picavet R., Bernard C. (eds.), Fouille archéologique de la Grande Rivoire à Sassenage (Isère). Rapport de fouille 2000-2003 (opération programmée pluriannuelle 2001-2003). Conservatoire du Patrimoine de l'Isère, Grenoble: 181-188.

Martin L. (2010). Agriculture et alimentation végétale en milieu montagnard au Néolithique : nouvelles données carpologiques dans les Alpes françaises du Nord. Unpublished PhD thesis, Université de Paris I Panthéon-Sorbonne/Basel University, Paris/Basel.

Martin L. (2014). Premiers paysans des Alpes. Alimentation végétale et agriculture au Néolithique. Presses Universitaires de Rennes/Presses Universitaires François Rabelais, Rennes/Tours.

Martin L., Bouby L., Marinval P., Dietsch-Sellami M.-F., Rousselet O., Cabanis M., Durand F., Figueiral I. (2016). L'exploitation des ressources végétales durant le Chasséen : un bilan des données carpologiques en France entre 4400 et 3500 avant notre ère. In : Perrin T., Chambon P., Gibaja J.F., Goude G. (eds.). Le Chasséen, des Chasséens… Retour sur une culture nationale et ses parallèles, Sepulcres de fossa, Cortaillod, Lagozza. Actes du colloque international tenu à Paris (France) du 18 au 20 novembre 2014. Archives d'Ecologie Préhistorique, Toulouse : 259-272.

Marty F., Bouby L., Ivorra S., Terral J.-F. (2016). Conserves d'olives hispaniques en amphores, au Ier s., sur le site de l'Estagnon (Fos-sur-Mer, Bouches-du-Rhône, Fr.). In : Djaoui D. (ed.). Histoires matérielles : terre cuite, bois, métal et autres objets. Des pots et des potes : mélanges offerts à Lucien Rivet. Editions Mergoil, Autun : 481-488.

Mauné S., Carrato C., Rovira N., Le Fur J., Longepierre S., et al. (2013). La boulangerie de Saint-Bézard à Aspiran (Hérault), du Ier s. au IVe s. apr. J.-C. : un exemple d'espace culinaire domanial en Narbonnaise centrale. Gallia, 70 (1) :165-190.

Pelletier D., Cabanis M. (2006). L'occupation du Néolithique moyen II de Champ Lamet III à Pont-du- Château (Puy-de-Dôme, Auvergne) : présentation générale du site et étude du cas particulier d'une aire de traitement de céréales. In: Gasco J. et al. (eds.), Hommes et passé des Causses : hommage à Georges Costantini. Actes du colloque de Millau, 16-18 juin 2005. Archives d'Ecologie Préhistoriques, Toulouse: 205- 222.

Pinaud-Querrac'h R. (2015). Agriculture, alimentation végétale et viniculture dans la Vallée du Rhône aux VIème et Vème siècles av. J.-C: étude carpologique du Mourre de Sève (Sorgues, Vaucluse). Unpublished Master 1 thesis. Université Paul Valery, Montpellier.

Pinaud-Querrac'h R., Rovira N., Beylier A., Gailledrat E. (2020). Les premières données carpologiques de La Monédière (Bessan, Hérault, France), Un comptoir littoral méditerranéen du premier âge du Fer. In : Auxiette G., Mougne C., Peake R., Toulemonde F. (Eds.), Autour de la table : l'alimentation à l'âge du Bronze et au premier âge du Fer. Actes de la journée thématique du 3 mars 2017 à Saint-Germain-en-Laye. Supplément 6 au Bulletin de l'Association pour la Promotion des Recherches sur l'Age du Bronze: 180-193.

Pottrain A., Py M. (1975). Un puits gallo-romain des IIème et IIIème siècles à Nages (Gard). Bulletin de l'Ecole Antique de Nîmes, 10 : 7-19.

Poux M., Argant T., Bouby L., Clément B., Gilles A., Leperlier M., Tillier M., Tripier A. (2013). Une culina de type « pompéien » en territoire lyonnais : l'espace culinaire de la villa de Goiffieux à Saint-Laurent d'Agny (Rhône). Gallia, 70, 1 : 135-164.

Pradat B. (1994). Les offrandes alimentaires végétales dans les tombes antiques : exemples d'incinérations à Lyon et à Marseille. Mémoire de DEA. Université de Paris I, Panthéon-Sorbonne, Paris, 85 p.

Rodriguez G., Marsac P., Erroux J. (1989). Le niveau Saintponien de la grotte-aven du Poteau, Saint-Pons (Hérault). Archéologie en Languedoc, 4 : 57-71.

Rovira, N. (2012a). Les restes carpologiques : intrusions ou offrandes ? In: Dedet B., avec la participation de Cenzon-Salvayre C., Durand A., Gardeisen A., Piquès G., Rovira N., Ambrussum, Hérault : un quartier de la nécropole du second âge du fer. Bibliothèque d'Archéologie Méditerranéenne et Africaine, 11: 269-272.

Rovira, N. (2012b). Les restes carpologiques. In: Fiches J.-L. (ed.), Quatre puits de l'agglomération routière d'Ambrussum (Villetelle, Hérault). Revue Archéologique de Narbonnaise, Supplément 42 : 133-158.

Rovira, N. (2015). Les carporestes du puits de l'espace 21 du Clos de la Lombarde (Narbonne, Aude). In: Sabrié R. (ed.), Le Clos de la Lombarde à Narbonne. Atelier de salaisons, Thermes, Maison IX, Rue D. Ed. Monique Mergoil: 363-376.

Rovira, N., Alonso, N. (2010). Thanatocoenoses of seeds and fruits from Zone 1 at Lattara (Lattes, France) during the 5th-4th centuries BC: the preliminary results. In: Bakels C., Fennema K., Out W. A., Vermeeren C. (eds.), Of Plants and Snails. A collection of papers presented to Wim Kuijper in gratitude for forty years of teaching and identifying. Sidestone Press, Leiden: 217-226.

Rovira N., Alonso N. (2017). Crop production and plant consumption on coastal Languedoc (France) in the Second Iron Age: new data from Pech Maho (Aude), Lattara (Hérault) and Le Cailar (Gard). *Vegetation History and Archaeobotany*, 27 (1): 85-97.

Rovira N., Chabal L. (2008). A foundation offering at the Roman port of Lattara (Lattes, France): the plant remains. Vegetation History and Archaeobotany, 17, Suppl. 1: 191-200.

Roudil J.-L. (1972). L'Age du Bronze du Languedoc oriental. Mém. de la Soc. Préhist. Franc., 10. Paris, Klincksieck, 302 p.

Roudil J.-L., Dedet B., Columeau P., Erroux J., Chabal L. (1993). Les débuts du Bronze final dans les gorges de la Cèze (Gard). I, La grotte du Hasard à Tharaux. Documents d'Archéologie Méridionale, 16 : 157-158.

Roudil J.-L., Roudil O., Soulier M. (1979). La grotte de l'Aigle à Méjannes le Clap (Gard) et le Néolithique Ancien du Languedoc -oriental. Mémoires Soc. languedocienne de Préhist., 1, Montpellier, 85 p.

Ruas M.-P. (1989a). Les paléosemences carbonisées de la fosse 1 (Antiquité tardive). Analyse paléocarpologique. In : Guilaine J., Vaquer J., Coularou J., Treinen-Claustre F. (eds.), Ornaisons-Médor. Archéologie et écologie d'un site de l'Age du Cuivre, de l'Age du Bronze final et de l'Antiquité tardive. Centre d'Anthropologie des Sociétés Rurales/Archéologie en Terre d'Aude, Toulouse/Carcassonne : 247-261.

Ruas M.-P. (1989b). Etude carpologique. In : Fiches J.-L. (ed.), L'Oppidum d'Ambrussum et son territoire. Fouilles au quartier du Sablas (Villetelle, Hérault) : 1979-1985. Monographie du CRA, 2. CNRS, Paris : 169-180.

Ruas M.-P., Bouby L., Campmajo P. (2009). Agriculture en montagne cerdane au Bronze final : les données carpologiques de Llo-Lo Lladre (Pyrénées-Orientales). In : De Méditerranée et d'ailleurs… Mélanges offerts à Jean Guilaine. Archives d'Ecologie Préhistorique, Toulouse : 639-660.

Savard M. (2000). Etude de l'assemblage carpologique de la Baume de Fontbrégoua (Var) du Paléolithique final au Chasséen récent. Unpublished Master thesis, Université Paris I Panthéon-Sorbonne, Université Paris X Nanterre, Paris.

Séjalon P., Py M., Chardenon N., Figueiral I., Forest V., Gafa R., Mourre V., Bousquet J (2014). Occupation du premier âge du Fer sur le site de La Condamine VII à Vauvert (Gard). Documents d'Archéologie Méridionale, 37: 89-142.

Tillier M. (2019). Economie végétale et échanges en Méditerranée romaine (Ier s. av. J.-C. – IIIe s. ap. J.-C.). Etude carpologique de contextes portuaires. Unpublished PhD thesis, Université Paul Valéry-Montpellier 3, Montpellier, 2 vol.

Tillier, M., Rovira, N. (2016). Les restes carpologiques. In: Demangeot, C., Py, M., Dedet, B., Carme, R., Cenzon, C., Roure, R., Rovira, N., Tillier, M., La nécropole du second âge du Fer du Sizen-Vigne à Beaucaire (Gard). Monographies d'Archéologie Méditerranéenne, 37: 223-226.

Toledo I Mur A., avec la collaboration de Bouby L., Bruxelle L., Decanter F., Lagarrigue A., Martin S., Martzluff M., Polloni A., Poirier P. (2018). Les fréquentations à vocation agricole du Néolithique final et de l'âge du Bronze à « El Camp del Viver », à Baho (Pyrénées-Orientales). *In* : Lemercier O., Sénepart I., Besse M., Mordant C. (dir.). *Habitations et habitat du Néolithique à l'âge du Bronze en France et ses marges. Actes des IIe Rencontres Nord/Sud de Préhistoire récente, Dijon, 19-21 novembre 2015*. Archives d'Ecologie Préhistorique, Toulouse : 693-698.

Vaquer J., Ruas, M.-P. (2009). La grotte de l'Abeurador Félines-Minervois (Hérault) : occupations humaines et environnement du Tardiglaciaire à l'Holocène. De Méditerranée et d'ailleurs… Mélanges offerts à Jean Guilaine. Toulouse, Archives d'Ecologie Préhistoriques: 761-792.

Verdin F. (1997). Coudounèu (Lançon-de-Provence, Bouches-du-Rhône), une ferme grenier et son terroir au Ve s. av. J.-C. Documents d'Archéologie Méridionale, 19-20 : 165-198.

Vernet J.-L. (1973). Etude sur l'histoire de la végétation du sud-est de la France au Quaternaire d'après les charbons de bois principalement. Paléobiologie continentale, 4 (1), 90 p.

Vital J., avec la collaboration de Bouby L., Jallet F., Rey P.-J. (2007). Un autre regard sur le gisement du boulevard périphérique nord de Lyon (Rhône) au Néolithique et à l'âge du Bronze. Gallia Préhistoire, 49 : 1-126.

Wiethold J., Treffort J.-M. (2002). Archäobotanische Funde als Hinweis auf Handels- und Kulturkontakte zum Mittelmeergebiet in der Hallstattzeit? Das Beispiel des Fundplatzes von "Roche Noire", Montagnieur (Ain), Frankreich. In: Lang A., Salac V. (eds.), Fernkontakte in der Eisenzeit. Konferenz, Liblice 2000. Archäologisches Institut der Akademie der Wissenschaften der Tschechischen Republik, Praha : 379-394.

Zeist van W., Guilaine J., Gasco J. (1983). L'orge du Bronze moyen de la grotte des Cazals (Sallèles-Cabardès, Aude). Bulletin de la Société Préhistorique Française, 80, 4 : 117-118.

Zwierzinski E., Ruas M.-P. (2004). Etude carpologique. In : Thernot R., Bel V., Mauné S. (eds.), L'établissement rural antique de Soumaltre à Aspiran (Hérault), Ferme, auberge, nécropole en bordure de la voie Cessero-Condatomagus (Ier-IIe s. ap. J.-C.). Archéologie et Histoire Romaine, 13, Mergoil (ed.), Montagnac : 209-215.
