# Supplementary material for: Talkin’ About a Revolution. Changes and Continuities in Fruit Use in Southern France From Neolithic to Roman Times Using Archaeobotanical Data (ca. 5,800 BCE – 500 CE)
Source: Front Plant Sci. 2022 Feb 7;13:719406. doi: 10.3389/fpls.2022.719406 (PMC8859487; doi:10.3389/fpls.2022.719406)
Supplement: Supplementary file 8 [file Table_2.pdf]

Supplementary Table 2. Detailed dataset for all the site-phases included in this study.

Site	Cod Sit-Phas	Pres	type	Period	DATE BP	Quantif	MNI	Citrul	Cumel	Cupre	Cusat	Cusp	Jugla	Lage	Mespi	Moru	Prcer	Prdul	Prpers	Punic	Zizip	Cordi	Phoen	Celti	Coryl	Ficu	Fraga	Maloid	Malu	Olea	Pinpi	Pravce	Pravi	Prdom
10 rue des frères de Turenne	FrdTur-10	U	URBAN	ERo	2000 - 1900	NMI	19	0	0	0	0	0	0	0	0	0	0	0	2	0	0	0	0	0	1	0	0	0	0	1	0	0	3	1
14 rue des Tuileries	LyTuil-8	C	RURAL	IA1/2	2525 - 2425	NMI	487	0	0	0	0	0	0	0	0	0	0	0	0	0	0	0	0	0	8	0	0	0	0	0	0	0	0	0
Acropole (Rue)	RAcro-6	C	RURAL	BA	3250 - 2900	NMI	2193	0	0	0	0	0	0	0	0	0	0	0	0	0	0	0	0	0	0	0	0	0	0	0	0	0	0	0
Acropole (Rue)	RAcro-10	C	RURAL	ERo	2000 - 1750	NMI	13	0	0	0	0	0	3	0	0	0	0	0	0	0	0	0	0	0	0	4	0	0	0	2	0	0	0	0
Acropole (Rue)	RAcro-11	C	RURAL	LRo	1750 - 1700	NMI	24	0	0	0	0	0	0	0	0	0	0	0	0	0	0	0	0	0	0	0	0	0	0	0	0	0	0	0
Aigle (Grotte de l')	GtAig-1	C	CAVE	Neo	7200 - 7000	NMI	5	0	0	0	0	0	0	0	0	0	0	0	0	0	0	0	0	0	0	0	0	0	0	0	0	0	0	0
Alba la Romaine	AlRom-10	C	FUN	ERo	1900 - 1800	P/A	2	0	0	0	0	0	0	0	0	0	0	0	0	0	0	0	0	0	0	0	0	0	0	0	2	0	0	0
Ambrussum	VlAmb-10	C	URBAN	ERo	2025 - 1750	NMI	9721	0	0	0	0	0	1	0	0	0	0	0	0	0	0	0	0	0	1	0	0	0	0	3	0	0	0	0
Ambrussum	VlAmb-10	U	URBAN	ERo	2000 - 1750	NMI	3924	0	10	0	3	0	866	0	0	1	0	2	58	0	0	0	0	125	43	657	2	0	51	180	48	227	0	14
Ambrussum	VlAmb-11	C	URBAN	LRo	1750 - 1600	NMI	17	0	0	0	0	0	1	0	0	0	0	0	0	0	0	0	0	0	0	0	0	0	0	0	0	0	0	0
Ambrussum	VlAmb-11	U	URBAN	LRo	1750 - 1600	NMI	2138	0	0	0	0	0	49	0	0	0	0	0	1	0	0	0	0	6	4	298	0	0	235	7	4	10	0	0
Ambrussum - funerary	VlAmbF-9a	C	FUN	IA2	2300 - 2200	NMI	40	0	0	0	0	0	0	0	0	0	0	0	0	0	0	0	0	0	1	0	0	0	0	0	0	0	0	0
Ambrussum - funerary	VlAmbF-9b	C	FUN	IA2	2100 - 2030	NMI	43	0	0	0	0	0	2	0	0	0	0	0	0	0	0	0	0	0	1	0	0	0	0	3	0	0	0	0
Ambrussum - funerary	VlAmbF-10	C	FUN	ERo	2015 - 1975	NMI	2	0	0	0	0	0	1	0	0	0	0	0	0	0	0	0	0	0	0	0	0	0	0	0	0	0	0	0
Arles-Rhône 3	ArlR3-10	C	URBAN	ERo	2025 - 1750	NMI	7	0	0	0	0	0	0	0	0	0	0	0	0	0	0	0	0	0	0	0	0	0	0	0	1	0	0	0
Arles-Rhône 3	ArlR3-10	U	URBAN	ERo	2000 - 1750	NMI	3397	0	0	5	0	0	78	0	0	3	0	12	1	10	0	0	0	0	13	2055	0	0	7	113	13	7	0	3
Arles-Rhône 5	ArlR5-10	C	URBAN	ERo	1900 - 1850	NMI	3	0	0	0	0	0	1	0	0	0	0	0	0	0	0	0	0	0	0	0	0	0	0	0	0	0	0	0
Arles-Rhône 5	ArlR5-10	U	URBAN	ERo	1900 - 1850	NMI	78	0	0	0	0	0	23	0	0	0	0	0	0	0	0	0	0	0	1	27	0	2	0	0	0	2	0	0
Arriasse	VFArs-7	C	RURAL	IA1	2700 - 2625	NMI	47	0	0	0	0	0	0	0	0	0	0	0	0	0	0	0	0	0	0	0	0	0	0	0	0	0	0	0
Artière Ronzière	ArRz-2	C	RURAL	Neo	6000 - 5500	NMI	59	0	0	0	0	0	0	0	0	0	0	0	0	0	0	0	0	0	0	0	0	0	0	0	0	0	0	0
Aspra del Paradis	AspPar-11	C	RURAL	LRo	1625 - 1550	NMI	40	0	0	0	0	0	0	0	0	0	0	0	0	0	0	0	0	0	0	0	0	0	0	0	0	0	0	0
Aspre del Paradis	AspPar-1	C	RURAL	Neo	7000 - 6500	NMI	3	0	0	0	0	0	0	0	0	0	0	0	0	0	0	0	0	0	1	0	0	0	0	0	0	0	0	0
Assas (Place)	NPAs-10	C	URBAN	ERo	2000 - 1900	NMI	141	0	0	0	0	0	0	0	0	0	0	0	0	0	0	0	0	0	0	0	0	0	0	0	0	0	0	0
Assas (Place)	NPAs-10	U	URBAN	ERo	2000 - 1900	NMI	4342	0	0	0	3	0	414	11	0	12	0	40	50	2	0	0	0	2	186	1147	0	4	0	1333	75	133	0	24
Aubettes (Les)	MuAub-Ro	C	RURAL	Rom	2000 - 1600	NMI	8	0	0	5	0	0	0	0	0	0	0	0	0	0	0	0	0	0	0	1	0	0	0	0	0	0	0	0
Aumignanes	Aumig-10	C	RURAL	ERo	1800 - 1750	NMI	4337	0	0	0	0	0	0	0	0	0	0	0	0	0	0	0	0	0	0	0	0	0	0	0	0	0	0	0
Auriac	Auria-2	C	RURAL	Neo	6000 - 5500	NMI	246	0	0	0	0	0	0	0	0	0	0	0	0	0	0	0	0	0	0	0	0	0	0	0	0	0	0	0
Auribelle-Basse (L')	PzAuri-10	U	URBAN	ERo	1855 - 1740	NMI	6838	0	0	0	0	4	6	0	0	0	0	1	5	0	0	0	0	0	2	208	1	0	0	5	8	0	0	2
Aven des Corneilles	AvCor-3	C	CAVE	Neo	4700 - 4200	NMI	98	0	0	0	0	0	0	0	0	0	0	0	0	0	0	0	0	0	0	0	0	0	0	0	0	0	0	0
Aven du Poteau (Grotte)	GtAPo-3	C	CAVE	Neo	5500 - 5100	NMI	319	0	0	0	0	0	0	0	0	0	0	0	0	0	0	0	0	0	0	0	0	0	0	0	0	0	0	0
Balme Gontran	Bgon-6	C	CAVE	BA	3100 - 2900	NMI	16962	0	0	0	0	0	0	0	0	0	0	0	0	0	0	0	0	0	0	0	0	0	0	0	0	0	0	0
Baou des Noirs	BadNo-6	C	VILLAGE	BA	3400 - 2750	NMI	48	0	0	0	0	0	0	0	0	0	0	0	0	0	0	0	0	0	0	0	0	0	0	0	0	0	0	0
Baou des Noirs	BadNo-7	C	VILLAGE	IA1	2600 - 2400	NMI	1513	0	0	0	0	0	0	0	0	0	0	0	0	0	0	0	0	0	0	0	0	0	0	0	0	0	0	0
Baou Roux	BaRx-4	C	RURAL	BA	4200 - 3700	NMI	5	0	0	0	0	0	0	0	0	0	0	0	0	0	0	0	0	0	0	0	0	0	0	0	0	0	0	0
Baou Roux	BaRx-6	C	VILLAGE	BA	2900 - 2750	NMI	18	0	0	0	0	0	0	0	0	0	0	0	0	0	0	0	0	0	0	0	0	0	0	0	0	0	0	0
Baou Roux	BaRx-9	C	VILLAGE	IA2	2425 - 2375	NMI	1864	0	0	0	0	0	0	0	0	0	0	0	0	0	0	0	0	0	0	0	0	0	0	0	0	0	0	0
Barreau de la Devèze Sud	BrDev-2	C	RURAL	Neo	6000 - 5500	NMI	488	0	0	0	0	0	0	0	0	0	0	0	0	0	0	0	0	0	0	0	0	0	0	0	0	0	0	0
Bauduen (Grotte de)	GtBaud-6	C	CAVE	BA	3350 - 2800	NMI	236	0	0	0	0	0	0	0	0	0	0	0	0	0	0	0	0	0	0	0	0	0	0	0	0	0	0	0
Baume Abeurador	BAbeu-1	C	CAVE	Neo	7400 - 7000	NMI	23	**0**	0	0	0	0	0	**0**	0	0	0	0	0	0	0	**0**	0	**0**	**15**	0	0	0	**0**	0	0	0	**0**	0
Baume Abeurador	BAbeu-2	C	CAVE	Neo	6500 - 6000	NMI	85	0	0	0	0	0	0	0	0	0	0	0	0	0	0	0	0	0	71	0	0	0	0	0	0	0	0	0
Baume Bourbon	BaBou-1	C	CAVE	Neo	7400 - 7000	NMI	32	0	0	0	0	0	0	0	0	0	0	0	0	0	0	0	0	0	0	0	0	0	0	0	0	0	0	0
Baume des Anges	DBAg-6	C	CAVE	BA	2900 - 2750	NMI	4	0	0	0	0	0	0	0	0	0	0	0	0	0	0	0	0	0	0	0	0	0	0	0	0	0	0	0
Baume d'Oullins	BaOul-1	C	CAVE	Neo	7400 - 7000	NMI	219	0	0	0	0	0	0	0	0	0	0	0	0	0	0	0	0	0	0	0	0	0	0	0	0	0	0	0
Baume Fontbrégoua	BFtbg-1	C	CAVE	Neo	7500 - 7000	P/A	15	0	0	0	0	0	0	0	0	0	0	0	0	0	0	0	0	0	1	0	0	0	0	0	0	0	0	0
Baume Fontbrégoua	BFtbg-2a	C	CAVE	Neo	6500 - 6000	NMI	1243	0	0	0	0	0	0	0	0	0	0	0	0	0	0	0	0	0	0	0	0	0	0	0	0	0	0	0
Baume Fontbrégoua	BFtbg-2b	C	CAVE	Neo	6000 - 5500	NMI	52	0	0	0	0	0	0	0	0	0	0	0	0	0	0	0	0	0	0	0	0	0	0	0	0	0	0	0
Baume Layrou	TrBlay-6	C	CAVE	BA	3200 - 3050	NMI	153235	0	0	0	0	0	0	0	0	0	0	0	0	0	0	0	0	0	68	0	0	0	0	0	0	0	0	0
Baume Layrou	TrBlay-6	U	CAVE	BA	3200 - 3050	NMI	182	0	0	0	0	0	0	0	0	0	0	0	0	0	0	0	0	0	0	0	4	0	0	0	0	0	0	0
Bayard (Valence)	Baya-7a	C	RURAL	IA1	2800 - 2700	NMI	213	0	0	0	0	0	0	0	0	0	0	0	0	0	0	0	0	0	3	0	0	0	0	0	0	0	0	0
Bayard (Valence)	Baya-7b	C	RURAL	IA1	2600 - 2500	NMI	1269	0	0	0	0	0	0	0	0	0	0	0	0	0	0	0	0	0	0	0	0	0	0	0	0	0	0	0
BDS	BzBDS-2	C	RURAL	Neo	6500 - 5500	NMI	557	0	0	0	0	0	0	0	0	0	0	0	0	0	0	0	0	0	0	0	0	0	0	0	0	0	0	0
Beaulieu	MtxBea-10	C	RURAL	ERo	2000 - 1900	NMI	2	0	0	0	0	0	0	0	0	0	0	0	0	0	0	0	0	0	0	0	0	0	0	0	0	0	0	0
Bélesta (Grotte de)	GtBel-5	C	CAVE	BA	3650 - 3350	NMI	4	0	0	0	0	0	0	0	0	0	0	0	0	0	0	0	0	0	0	0	0	0	0	0	0	0	0	0
Bélesta (Grotte de)	GtBel-6	C	CAVE	BA	3300 - 2800	NMI	230	0	0	0	0	0	0	0	0	0	0	0	0	0	0	0	0	0	0	0	0	0	0	0	0	0	0	0
Bélesta (Grotte de)	GtBel-7	C	CAVE	IA1	2750 - 2450	NMI	138	0	0	0	0	0	0	0	0	0	0	0	0	0	0	0	0	0	0	0	0	0	0	0	0	0	0	0
Bente Farine	CerBF-11	C	RURAL	LRo	1650 - 1600	NMI	8	0	0	0	0	0	0	0	0	0	0	0	0	0	0	0	0	0	0	0	0	0	0	0	0	0	0	0
Béragne	Bergn-9	C	RURAL	IA2	2125 - 2050	NMI	13158	0	0	0	0	0	0	0	0	0	0	0	0	0	0	0	0	0	0	0	0	0	0	0	0	0	0	0
Berthet 1	LyBer1-8	C	RURAL	IA1/2	2525 - 2450	NMI	50	0	0	0	0	0	0	0	0	0	0	0	0	0	0	0	0	0	0	0	0	0	0	0	0	0	0	0
Berthet 1 - funerary	LyBer1F-10	C	FUN	ERo	1825 - 1775	NMI	25	0	0	0	0	0	4	0	0	0	0	0	0	0	0	0	0	0	0	0	0	0	0	6	0	0	0	0
Berthet 2	LyBer2-6	C	RURAL	BA	2900 - 2700	NMI	58	0	0	0	0	0	0	0	0	0	0	0	0	0	0	0	0	0	1	0	0	0	0	0	0	0	0	0
Berthet 2	LyBer2-8	C	RURAL	IA1/2	2525 - 2450	NMI	199	0	0	0	0	0	0	0	0	0	0	0	0	0	0	0	0	0	4	0	0	0	0	0	0	0	0	0

Site	Cod Sit-Phas	Pres	type	Period	DATE BP	Prsp	Pyru	Viti	Vitipd	Arbu	Corma	Corsa	Crata	Junsp	Myrt	Physa	Pincem	Pinsp	Pista	Prspi	Querc	Rhus	Rosa	Rubca	Rubfr	Rubid	Rubsp	Sambeb	Sambni	Sambsp	Sorb	Vibu	An crops
10 rue des frères de Turenne	FrdTur-10	U	URBAN	ERo	2000 - 1900	0	0	0	0	0	0	0	0	0	0	0	0	0	0	11	0	0	0	0	0	0	0	0	0	0	0	0	0
14 rue des Tuileries	LyTuil-8	C	RURAL	IA1/2	2525 - 2425	0	0	0	0	0	0	0	0	0	0	0	0	0	0	1	0	0	0	0	0	0	0	0	0	0	0	0	478
Acropole (Rue)	RAcro-6	C	RURAL	BA	3250 - 2900	0	0	2	0	0	0	0	0	0	0	0	0	0	0	0	0	0	0	0	0	0	0	0	0	0	0	0	2191
Acropole (Rue)	RAcro-10	C	RURAL	ERo	2000 - 1750	0	0	3	0	0	0	0	0	0	0	0	0	0	0	0	0	0	0	0	0	0	0	0	0	0	0	0	1
Acropole (Rue)	RAcro-11	C	RURAL	LRo	1750 - 1700	0	0	24	0	0	0	0	0	0	0	0	0	0	0	0	0	0	0	0	0	0	0	0	0	0	0	0	0
Aigle (Grotte de l')	GtAig-1	C	CAVE	Neo	7200 - 7000	0	0	0	0	0	0	2	0	0	0	0	0	0	0	0	0	0	0	0	0	0	0	0	0	0	0	0	3
Alba la Romaine	AlRom-10	C	FUN	ERo	1900 - 1800	0	0	0	0	0	0	0	0	0	0	0	0	0	0	0	0	0	0	0	0	0	0	0	0	0	0	0	0
Ambrussum	VlAmb-10	C	URBAN	ERo	2025 - 1750	0	0	333	1	1	0	0	0	0	0	0	0	0	0	0	0	0	0	0	0	0	0	0	0	0	0	0	9381
Ambrussum	VlAmb-10	U	URBAN	ERo	2000 - 1750	0	12	931	26	0	1	7	11	0	0	0	0	0	2	15	8	0	4	0	166	0	0	0	446	0	0	0	8
Ambrussum	VlAmb-11	C	URBAN	LRo	1750 - 1600	0	0	1	0	0	0	0	0	0	0	0	0	0	0	0	0	0	0	0	0	0	0	0	0	0	0	0	15
Ambrussum	VlAmb-11	U	URBAN	LRo	1750 - 1600	0	214	648	1	0	0	21	3	0	0	0	0	0	5	3	0	0	2	0	129	1	0	0	496	0	0	0	1
Ambrussum - funerary	VlAmbF-9a	C	FUN	IA2	2300 - 2200	0	0	21	0	0	1	0	0	0	0	0	0	0	0	0	1	0	0	0	0	0	0	0	0	0	0	0	16
Ambrussum - funerary	VlAmbF-9b	C	FUN	IA2	2100 - 2030	0	0	27	0	0	0	0	0	0	0	0	0	0	0	0	0	0	0	0	0	0	0	0	0	0	0	0	10
Ambrussum - funerary	VlAmbF-10	C	FUN	ERo	2015 - 1975	0	0	1	0	0	0	0	0	0	0	0	0	0	0	0	0	0	0	0	0	0	0	0	0	0	0	0	0
Arles-Rhône 3	ArlR3-10	C	URBAN	ERo	2025 - 1750	0	0	0	0	0	0	0	0	0	0	0	0	0	0	0	0	0	0	0	0	0	0	0	0	0	0	0	6
Arles-Rhône 3	ArlR3-10	U	URBAN	ERo	2000 - 1750	0	5	913	0	0	0	0	0	0	1	0	0	0	0	0	6	0	0	0	23	0	0	0	3	0	0	0	126
Arles-Rhône 5	ArlR5-10	C	URBAN	ERo	1900 - 1850	0	0	1	0	0	0	0	0	0	0	0	0	0	0	0	0	0	0	0	0	0	0	0	0	0	0	0	1
Arles-Rhône 5	ArlR5-10	U	URBAN	ERo	1900 - 1850	0	0	7	0	0	0	0	0	0	0	0	0	0	0	0	0	0	0	0	0	0	1	0	0	2	0	0	13
Arriasse	VFArs-7	C	RURAL	IA1	2700 - 2625	0	0	0	0	0	0	0	0	0	0	0	0	0	0	0	7	0	0	0	0	0	0	0	0	0	0	0	40
Artière Ronzière	ArRz-2	C	RURAL	Neo	6000 - 5500	0	0	2	0	0	0	0	0	0	0	0	0	0	0	0	0	0	0	0	0	0	0	0	0	0	0	0	57
Aspra del Paradis	AspPar-11	C	RURAL	LRo	1625 - 1550	0	0	2	0	0	0	0	0	0	0	0	0	0	0	0	0	0	0	0	1	0	0	0	0	0	0	0	37
Aspre del Paradis	AspPar-1	C	RURAL	Neo	7000 - 6500	0	0	0	0	0	0	0	0	0	0	0	0	0	0	0	0	0	0	0	0	0	0	0	0	0	0	0	2
Assas (Place)	NPAs-10	C	URBAN	ERo	2000 - 1900	0	0	8	0	0	0	0	0	0	0	0	0	0	0	0	0	0	0	0	0	0	0	0	0	0	0	0	133
Assas (Place)	NPAs-10	U	URBAN	ERo	2000 - 1900	0	0	268	11	0	7	0	1	0	0	1	0	0	0	1	4	0	6	0	6	3	0	0	1	0	0	0	597
Aubettes (Les)	MuAub-Ro	C	RURAL	Rom	2000 - 1600	0	0	2	0	0	0	0	0	0	0	0	0	0	0	0	0	0	0	0	0	0	0	0	0	0	0	0	0
Aumignanes	Aumig-10	C	RURAL	ERo	1800 - 1750	0	0	0	0	0	0	0	0	0	0	0	0	0	0	0	0	0	0	0	0	0	0	0	0	0	0	0	4337
Auriac	Auria-2	C	RURAL	Neo	6000 - 5500	0	0	0	0	0	0	0	0	0	0	0	0	0	0	0	0	0	0	0	0	0	0	0	0	0	0	0	246
Auribelle-Basse (L')	PzAuri-10	U	URBAN	ERo	1855 - 1740	0	0	5945	499	0	0	3	0	0	0	0	0	0	0	2	3	0	0	2	15	0	0	61	59	0	0	0	7
Aven des Corneilles	AvCor-3	C	CAVE	Neo	4700 - 4200	0	0	0	0	0	0	0	0	0	0	0	0	0	0	0	0	0	0	0	0	0	0	0	0	0	0	0	98
Aven du Poteau (Grotte)	GtAPo-3	C	CAVE	Neo	5500 - 5100	0	0	0	0	0	0	0	0	0	0	0	0	0	0	0	0	0	0	0	0	0	0	0	0	0	0	0	319
Balme Gontran	Bgon-6	C	CAVE	BA	3100 - 2900	0	0	0	0	0	0	0	0	0	0	0	0	0	0	0	0	0	0	0	0	0	0	0	0	0	0	0	16962
Baou des Noirs	BadNo-6	C	VILLAGE	BA	3400 - 2750	0	0	2	0	0	0	0	0	0	0	0	0	0	0	0	0	0	0	0	0	0	0	0	0	0	0	0	46
Baou des Noirs	BadNo-7	C	VILLAGE	IA1	2600 - 2400	0	0	0	0	4	0	0	0	0	0	0	0	0	0	0	1509	0	0	0	0	0	0	0	0	0	0	0	0
Baou Roux	BaRx-4	C	RURAL	BA	4200 - 3700	0	0	0	0	0	0	0	0	0	0	0	0	0	0	0	0	0	0	0	0	0	0	0	0	0	0	0	5
Baou Roux	BaRx-6	C	VILLAGE	BA	2900 - 2750	0	0	0	0	0	0	0	0	0	0	0	0	0	0	0	0	0	0	0	0	0	0	0	0	0	0	0	18
Baou Roux	BaRx-9	C	VILLAGE	IA2	2425 - 2375	0	0	28	0	0	0	0	0	0	0	0	0	0	0	0	Présent	0	0	0	0	0	0	0	0	0	0	0	1836
Barreau de la Devèze Sud	BrDev-2	C	RURAL	Neo	6000 - 5500	0	0	0	0	0	0	0	0	0	0	0	0	0	0	0	0	0	0	0	0	0	0	0	0	0	0	0	488
Bauduen (Grotte de)	GtBaud-6	C	CAVE	BA	3350 - 2800	0	0	0	0	0	21	0	0	0	0	0	0	0	0	0	0	0	0	0	0	0	0	0	0	0	0	0	215
Baume Abeurador	BAbeu-1	C	CAVE	Neo	7400 - 7000	0	0	**1**	0	**0**	0	**0**	0	4	0	0	0	**0**	**0**	**2**	**0**	**0**	**0**	0	**0**	0	**0**	**0**	**0**	**0**	**0**	**0**	1
Baume Abeurador	BAbeu-2	C	CAVE	Neo	6500 - 6000	0	0	0	0	0	0	0	0	0	0	0	0	0	0	0	0	0	0	0	0	0	0	0	0	0	0	0	14
Baume Bourbon	BaBou-1	C	CAVE	Neo	7400 - 7000	0	0	0	0	0	0	0	0	0	0	0	0	0	0	0	0	0	0	0	0	0	0	0	0	0	0	0	32
Baume des Anges	DBAg-6	C	CAVE	BA	2900 - 2750	0	0	0	0	0	0	0	0	0	0	0	0	0	0	0	0	0	0	0	0	0	0	0	0	0	0	0	4
Baume d'Oullins	BaOul-1	C	CAVE	Neo	7400 - 7000	0	0	0	0	0	0	3	0	0	0	0	0	0	0	0	0	0	0	0	0	0	0	0	0	0	0	0	216
Baume Fontbrégoua	BFtbg-1	C	CAVE	Neo	7500 - 7000	1	0	1	0	0	0	1	0	1	0	0	0	0	1	0	1	0	0	0	0	0	0	0	0	0	0	0	8
Baume Fontbrégoua	BFtbg-2a	C	CAVE	Neo	6500 - 6000	0	0	3	0	0	0	1	0	2	0	0	0	0	0	0	2	0	0	0	0	0	0	0	0	0	0	0	1235
Baume Fontbrégoua	BFtbg-2b	C	CAVE	Neo	6000 - 5500	0	0	1	0	0	0	0	0	0	0	0	0	0	0	0	1	0	0	0	0	0	0	0	0	0	0	0	50
Baume Layrou	TrBlay-6	C	CAVE	BA	3200 - 3050	0	0	0	0	0	0	0	0	0	0	0	0	0	0	0	11	0	0	0	4	0	0	0	0	0	0	0	153152
Baume Layrou	TrBlay-6	U	CAVE	BA	3200 - 3050	0	0	0	0	0	0	0	0	0	0	0	0	0	0	0	3	0	7	0	12	0	0	7	2	0	0	0	147
Bayard (Valence)	Baya-7a	C	RURAL	IA1	2800 - 2700	0	0	0	0	0	1	0	0	0	0	0	0	0	0	0	1	0	0	0	0	0	0	0	0	0	0	0	208
Bayard (Valence)	Baya-7b	C	RURAL	IA1	2600 - 2500	1	0	1	0	0	0	0	0	0	0	0	0	0	0	0	0	0	0	0	0	0	0	0	1	0	0	0	1266
BDS	BzBDS-2	C	RURAL	Neo	6500 - 5500	0	0	0	0	0	0	0	0	0	0	0	0	0	0	0	0	0	0	0	0	0	0	0	0	0	0	0	557
Beaulieu	MtxBea-10	C	RURAL	ERo	2000 - 1900	0	0	1	0	0	0	0	0	0	0	0	0	0	0	0	0	0	0	0	0	0	0	0	0	0	0	0	1
Bélesta (Grotte de)	GtBel-5	C	CAVE	BA	3650 - 3350	0	0	0	0	0	0	0	0	0	0	0	0	0	0	0	0	0	0	0	0	0	0	0	0	0	0	0	4
Bélesta (Grotte de)	GtBel-6	C	CAVE	BA	3300 - 2800	0	0	3	0	0	0	0	0	0	0	0	0	0	0	0	3	0	0	0	0	0	0	0	0	0	0	0	224
Bélesta (Grotte de)	GtBel-7	C	CAVE	IA1	2750 - 2450	0	0	0	0	0	0	0	0	0	0	0	0	0	0	0	5	0	0	0	0	0	0	0	0	0	0	0	133
Bente Farine	CerBF-11	C	RURAL	LRo	1650 - 1600	0	0	2	0	0	0	0	0	0	0	0	0	0	0	0	0	0	0	0	0	0	0	0	0	0	0	0	6
Béragne	Bergn-9	C	RURAL	IA2	2125 - 2050	0	0	0	0	0	0	0	0	0	0	0	0	0	0	0	0	0	0	0	0	0	0	0	0	0	0	0	13158
Berthet 1	LyBer1-8	C	RURAL	IA1/2	2525 - 2450	0	0	1	0	0	0	0	0	0	0	0	0	0	0	0	0	0	0	0	0	0	0	4	0	0	0	0	45
Berthet 1 - funerary	LyBer1F-10	C	FUN	ERo	1825 - 1775	0	0	15	0	0	0	0	0	0	0	0	0	0	0	0	0	0	0	0	0	0	0	0	0	0	0	0	0
Berthet 2	LyBer2-6	C	RURAL	BA	2900 - 2700	0	0	0	0	0	0	0	0	0	0	0	0	0	0	0	0	0	0	0	0	0	0	0	0	0	0	0	57
Berthet 2	LyBer2-8	C	RURAL	IA1/2	2525 - 2450	0	0	1	0	0	0	0	0	0	0	0	0	0	0	0	0	0	0	0	0	0	0	11	0	0	0	0	183

Site	Cod Sit-Phas	Pres	type	Period	DATE BP	Quantif	MNI	Citrul	Cumel	Cupre	Cusat	Cusp	Jugla	Lage	Mespi	Moru	Prcer	Prdul	Prpers	Punic	Zizip	Cordi	Phoen	Celti	Coryl	Ficu	Fraga	Maloid	Malu	Olea	Pinpi	Pravce	Pravi	Prdom
Berthet 2	LyBer2-8	U	RURAL	IA1/2	2525 - 2450	NMI	981	0	0	0	0	0	0	0	0	0	0	0	0	0	0	0	0	0	2	0	0	0	2	0	0	0	0	0
BMVR	BMVR-9	C	OFFSITE	IA2	2300 - 2200	NMI	3	0	0	0	0	0	0	0	0	0	0	0	0	0	0	0	0	0	0	0	0	0	0	0	0	0	0	0
BMVR	BMVR-10	C	OFFSITE	ERo	1900 - 1800	NMI	6	0	0	0	0	0	0	0	0	0	0	0	0	0	0	0	0	0	0	0	0	0	0	0	0	0	0	0
BMVR	BMVR-10	U	OFFSITE	ERo	1900 - 1800	NMI	531	0	0	0	0	0	0	1	0	1	0	0	0	1	0	0	0	0	0	356	0	0	4	6	0	0	0	2
BMVR	BMVR-11	C	OFFSITE	LRo	1600 - 1500	NMI	1	0	0	0	0	0	0	0	0	0	0	0	0	0	0	0	0	0	0	0	0	0	0	0	0	0	0	0
Bouc-Bel-Air	BcBA-3	C	RURAL	Neo	5500 - 4200	NMI	1044	0	0	0	0	0	0	0	0	0	0	0	0	0	0	0	0	0	0	0	0	0	0	0	0	0	0	0
Bouquet (Le)	MtBouq-6	C	RURAL	BA	3400 - 3200	NMI	83	0	0	0	0	0	0	0	0	0	0	0	0	0	0	0	0	0	0	0	0	0	0	0	0	0	0	0
Bouquet (Le)	MtBouq-7	C	RURAL	IA1	2750 - 2600	NMI	43	0	0	0	0	0	0	0	0	0	0	0	0	0	0	0	0	0	1	0	0	0	0	0	0	0	0	0
Bourbou	LpBo-11	C	RURAL	LRo	1750 - 1600	NMI	99	0	0	0	0	0	0	0	0	0	0	0	0	0	0	0	0	0	0	0	0	0	0	7	0	0	0	0
Bourbousson 1	CB1-8	C	RURAL	IA1/2	2500 - 2450	NMI	144	0	0	0	0	0	0	0	0	0	0	0	0	0	0	0	0	0	0	0	0	0	0	0	0	0	0	0
Bourbousson 3	CB3-10	C	RURAL	ERo	1825 - 1750	NMI	164	0	0	0	0	0	0	0	0	0	0	0	0	0	0	0	0	0	0	0	0	0	0	0	0	0	0	0
Bourbousson 3	CB3-11	C	RURAL	LRo	1725 - 1700	NMI	123531	0	0	0	0	0	117	0	0	0	0	0	0	0	0	0	0	0	14	0	0	0	46	0	0	0	0	38
Bousquetas	PaBsq-3	C	RURAL	Neo	5000 - 4700	NMI	2007	0	0	0	0	0	0	0	0	0	0	0	0	0	0	0	0	0	0	0	0	0	0	0	0	0	0	0
Boussargues	ArgBou-3	C	RURAL	Neo	4700 - 4200	NMI	18199	0	0	0	0	0	0	0	0	0	0	0	0	0	0	0	0	0	0	0	0	0	0	0	0	1	0	0
BPNL	BPNL-4	C	RURAL	BA	4200 - 3650	NMI	4068	0	0	0	0	0	0	0	0	0	0	0	0	0	0	0	0	0	9	0	0	1	0	0	0	0	0	0
BPNL	BPNL-6	C	RURAL	BA	3450 - 3300	NMI	7983	0	0	0	0	0	0	0	0	0	0	0	0	0	0	0	0	0	2	0	0	0	0	0	0	0	0	0
BPNL	BPNL-8	C	RURAL	IA1/2	2525 - 2450	NMI	31	0	0	0	0	0	0	0	0	0	0	0	0	0	0	0	0	0	5	0	0	0	0	0	0	0	0	0
BPNL	BPNL-10	C	RURAL	ERo	2000 - 1800	NMI	2	0	0	0	0	0	0	0	0	0	0	0	0	0	0	0	0	0	0	0	0	0	0	0	0	0	0	0
BPNL	BPNL-11	C	RURAL	LRo	1700 - 1600	NMI	14	0	0	0	0	0	4	0	0	0	0	0	0	0	0	0	0	0	0	0	0	0	0	0	0	0	0	0
BPNL - funerary	BPNLfu-10	C	FUN	ERo	2027 - 1950	NMI	40	0	0	0	0	0	8	0	0	0	4	0	0	0	0	0	0	0	11	0	0	0	0	0	0	0	0	0
BPNL - funerary	BPNLfu-10a	C	FUN	ERo	1890 - 1840	NMI	122	0	0	0	0	0	0	0	0	0	0	0	0	0	0	0	0	0	1	0	0	0	0	0	0	0	0	2
BPNL - funerary	BPNLfu-10b	C	FUN	ERo	1800 - 1750	NMI	576	0	0	0	0	0	0	0	0	0	0	0	0	0	0	0	0	0	1	0	0	0	0	0	0	0	0	11
Brassières Nord	MDBN-9a	C	RURAL	IA2	2150 - 2100	NMI	3	0	0	0	0	0	0	0	0	0	0	0	0	0	0	0	0	0	0	0	0	0	0	0	0	0	0	0
Brassières Nord	MDBN-9b	C	RURAL	IA2	2100 - 2050	NMI	16	0	0	0	0	0	0	0	0	0	0	0	0	0	0	0	0	0	0	0	0	0	0	0	0	0	0	0
Brassières Nord	MDBN-9c	C	RURAL	IA2	2050 - 2020	NMI	1	0	0	0	0	0	0	0	0	0	0	0	0	0	0	0	0	0	0	0	0	0	0	0	0	0	0	0
Brassières Nord	MDBN-10	C	RURAL	ERo	2020 - 1975	NMI	20	0	0	0	0	0	0	0	0	0	0	0	0	0	0	0	0	0	0	0	0	0	0	0	0	0	0	0
Brassières Sud	MDBS-7	C	RURAL	IA1	2750 - 2625	NMI	15	0	0	0	0	0	0	0	0	0	0	0	0	0	0	0	0	0	0	0	0	0	0	0	0	0	0	0
Brégoule	Brego-6	C	RURAL	BA	2800 - 2700	NMI	1263	0	0	0	0	0	0	0	0	0	0	0	0	0	0	0	0	0	0	0	0	0	0	0	0	0	0	0
Buffe Arnaud	BufAr-9	C	VILLAGE	IA2	2150 - 2100	P/A	26	0	0	0	0	0	0	0	0	0	0	0	0	0	0	0	0	0	0	0	0	0	0	0	0	0	0	0
Buffens (grotte de)	GtBuf-5	C	CAVE	BA	3650 - 3350	NMI	223	0	0	0	0	0	0	0	0	0	0	0	0	0	0	0	0	0	0	0	0	0	0	0	0	0	0	0
C et G (Grotte)	GtCG-2	C	CAVE	Neo	6000 - 5500	NMI	228	0	0	0	0	0	0	0	0	0	0	0	0	0	0	0	0	0	0	0	0	0	0	0	0	0	0	0
Cadenet	SeCad-10	C	RURAL	ERo	2000 - 1800	NMI	23004	0	0	0	0	0	0	0	0	0	0	0	0	0	0	0	0	0	0	0	0	0	0	0	0	0	0	0
Cadoules	MaCad-3	C	RURAL	Neo	5500 - 4200	NMI	3	0	0	0	0	0	0	0	0	0	0	0	0	0	0	0	0	0	0	0	0	0	0	0	0	0	0	0
Cailar (Le)	Lcail-9a	C	RURAL	IA2	2500 - 2400	NMI	4810	0	0	0	0	0	0	0	0	0	0	1	0	0	0	0	0	0	0	0	0	0	0	1	0	0	0	0
Cailar (Le)	Lcail-9b	C	RURAL	IA2	2300 - 2200	NMI	882	0	0	0	0	0	0	0	0	0	0	0	0	0	0	0	0	0	0	1	0	0	0	0	0	0	0	0
Caisses Saint Jean	CStJea-9a	C	VILLAGE	IA2	2455 - 2400	NMI	50	0	0	0	0	0	0	0	0	0	0	0	0	0	0	0	0	0	0	0	0	0	0	0	0	0	0	0
Caisses Saint Jean	CStJea-9b	C	VILLAGE	IA2	2150 - 2100	NMI	15	0	0	0	0	0	0	0	0	0	0	0	0	0	0	0	0	0	0	0	0	0	0	2	0	0	0	0
Calade	Calad-10	C	FUN	ERo	1960 - 1750	NMI	2	0	0	0	0	0	0	0	0	0	0	0	0	0	0	0	1	0	0	1	0	0	0	0	0	0	0	0
Camelin (Le)	FrCam-10a	C	URBAN	ERo	2040 - 1900	NMI	622	0	0	0	0	0	10	0	0	0	0	0	0	0	0	0	0	0	3	0	0	0	0	16	574	0	0	0
Camelin (Le)	FrCam-10b	C	URBAN	ERo	1900 - 1800	NMI	3	0	0	0	0	0	0	0	0	0	0	0	0	0	0	0	0	0	0	1	0	0	0	0	0	0	0	0
Camelin (Le)	FrCam-10b	U	URBAN	ERo	1900 - 1800	NMI	3287	0	0	0	0	0	0	0	0	0	0	0	0	0	0	0	0	0	0	3033	0	2	0	0	0	0	0	0
Camp de la Torre	LPCTo-9	C	RURAL	IA2	2200 - 2075	NMI	27	0	0	0	0	0	0	0	0	0	0	0	0	0	0	0	0	0	0	0	0	0	0	1	0	0	0	0
Camp del Viver	CaViv-6	C	RURAL	BA	3100 - 2920	NMI	17	0	0	0	0	0	0	0	0	0	0	0	0	0	0	0	0	0	0	0	0	0	0	0	0	0	0	0
Capelles (Lac de)	LaCap-10	C	RURAL	ERo	2030 - 1990	NMI	5	0	0	0	0	0	1	0	0	0	0	0	0	0	0	0	0	0	0	0	0	0	0	0	2	0	0	0
Capelles (Lac de)	LaCap-10	U	RURAL	ERo	2030 - 1990	NMI	4502	0	0	592	0	0	314	0	0	6	0	901	0	0	0	0	1	0	189	317	0	1	0	299	28	229	0	12
Careiron & Pesquier	C&Pesq-11	C	RURAL	LRo	1625 - 1600	NMI	433	0	0	0	0	0	0	0	0	0	0	0	0	0	0	0	0	0	0	0	0	0	0	0	0	0	0	0
Careiron & Pesquier	C&Pesq-11	U	RURAL	LRo	1625 - 1600	NMI	147	0	0	0	0	0	0	0	0	0	0	0	0	0	0	0	0	0	0	3	0	0	0	1	0	0	0	0
Cariaux	CrxFro-10	C	OFFSITE	ERo	2000 - 1770	NMI	10	0	0	0	0	0	0	0	0	0	0	0	0	0	0	0	0	0	0	0	0	0	0	0	0	0	0	0
Cariaux	CrxFro-10	U	OFFSITE	ERo	2000 - 1770	NMI	702	0	0	0	0	0	7	1	0	0	0	0	0	0	0	0	0	0	14	3	0	0	1	0	0	0	2	5
Carrerrassa (La)	Carre-4	C	RURAL	BA	4200 - 3650	NMI	59	0	0	0	0	0	0	0	0	0	0	0	0	0	0	0	0	0	0	0	0	0	0	0	0	0	0	0
Carsac	Carsa-6	C	VILLAGE	BA	3050 - 2750	NMI	5	0	0	0	0	0	0	0	0	0	0	0	0	0	0	0	0	0	0	0	0	0	0	0	0	0	0	0
Carsac	Carsa-7	C	VILLAGE	IA1	2750 - 2450	NMI	275	0	0	0	0	0	0	0	0	0	0	0	0	0	0	0	0	0	0	0	0	0	0	0	0	0	0	0
Castellan	CastIs-9	C	VILLAGE	IA2	2200 - 2100	NMI	779	0	0	0	0	0	0	0	0	0	0	0	0	0	0	0	0	0	0	0	0	0	0	0	0	0	0	0
Castellas	CasRo-9	C	VILLAGE	IA2	2150 - 2050	NMI	2	0	0	0	0	0	1	0	0	0	0	0	0	0	0	0	0	0	0	0	0	0	0	1	0	0	0	0
Castelle et Fromigue - funerary	CstFro-10	C	FUN	ERo	2000 - 1800	NMI	96	0	0	0	0	0	0	0	0	0	0	0	0	0	0	0	0	0	0	0	0	0	0	1	0	0	0	0
Castelle GR	CstGR-10	C	RURAL	ERo	1900 - 1700	NMI	7	0	0	3	0	0	1	0	0	0	0	0	0	0	0	0	0	0	0	0	0	0	0	0	0	0	0	0
Castelle GR	CstGR-10	U	RURAL	ERo	1900 - 1700	NMI	3491	0	0	24	0	0	7	0	0	3	0	0	0	0	0	0	0	0	0	1861	0	0	0	7	5	1	0	0
Castelle-Pahon-Pinède - funerary	CstPP-10	C	FUN	ERo	2000 - 1800	NMI	354	0	0	0	0	0	99	0	0	0	0	0	0	0	0	0	0	0	0	0	0	0	0	1	0	0	0	0
Cazals (Grotte de)	GtCaz-5	C	CAVE	BA	3740 - 3535	NMI	9265	0	0	0	0	0	0	0	0	0	0	0	0	0	0	0	0	0	0	0	0	0	0	0	0	0	0	0
Centre des Impôts	CDImp-3	C	RURAL	Neo	4700 - 4400	NMI	23	0	0	0	0	0	0	0	0	0	0	0	0	0	0	0	0	0	2	0	0	0	0	0	0	0	0	0
Ceron	VeCero-1	C	RURAL	Neo	7000 - 6500	NMI	2	0	0	0	0	0	0	0	0	0	0	0	0	0	0	0	0	0	0	0	0	0	0	0	0	0	0	0
Champ du Perrier (ZAC du)	ChdPe-7	C	RURAL	IA1	2850 - 2600	NMI	42	0	0	0	0	0	0	0	0	0	0	0	0	0	0	0	0	0	0	0	0	0	0	0	0	0	0	0
Champ du Poste (Le)	ChdPo-2	C	RURAL	Neo	6500 - 6000	NMI	136	0	0	0	0	0	0	0	0	0	0	0	0	0	0	0	0	0	0	0	0	0	0	0	0	0	0	0

Site	Cod Sit-Phas	Pres	type	Period	DATE BP	Prsp	Pyru	Viti	Vitipd	Arbu	Corma	Corsa	Crata	Junsp	Myrt	Physa	Pincem	Pinsp	Pista	Prspi	Querc	Rhus	Rosa	Rubca	Rubfr	Rubid	Rubsp	Sambeb	Sambni	Sambsp	Sorb	Vibu	An crops
Berthet 2	LyBer2-8	U	RURAL	IA1/2	2525 - 2450	0	0	45	0	0	0	0	0	0	0	0	0	0	0	0	0	0	0	0	112	0	0	684	135	0	0	0	0
BMVR	BMVR-9	C	OFFSITE	IA2	2300 - 2200	0	0	3	0	0	0	0	0	0	0	0	0	0	0	0	0	0	0	0	0	0	0	0	0	0	0	0	0
BMVR	BMVR-10	C	OFFSITE	ERo	1900 - 1800	0	0	1	1	0	0	0	0	0	0	0	0	0	0	0	0	0	0	0	0	0	0	0	0	0	0	0	4
BMVR	BMVR-10	U	OFFSITE	ERo	1900 - 1800	0	0	59	7	0	0	0	0	0	13	0	0	0	0	0	0	0	1	0	14	0	0	0	0	0	0	0	66
BMVR	BMVR-11	C	OFFSITE	LRo	1600 - 1500	0	0	1	0	0	0	0	0	0	0	0	0	0	0	0	0	0	0	0	0	0	0	0	0	0	0	0	0
Bouc-Bel-Air	BcBA-3	C	RURAL	Neo	5500 - 4200	0	0	0	0	0	0	0	0	0	0	0	0	0	0	0	0	0	0	0	0	0	0	0	0	0	0	0	1044
Bouquet (Le)	MtBouq-6	C	RURAL	BA	3400 - 3200	0	0	0	0	0	0	1	0	0	0	0	0	0	0	0	0	0	0	0	0	0	0	13	1	0	0	0	68
Bouquet (Le)	MtBouq-7	C	RURAL	IA1	2750 - 2600	0	0	5	0	0	0	0	0	0	0	0	0	0	0	0	0	0	0	0	0	0	0	0	0	0	0	0	37
Bourbou	LpBo-11	C	RURAL	LRo	1750 - 1600	0	0	75	13	0	0	0	0	0	0	0	0	0	0	0	0	0	0	0	0	0	0	0	0	0	0	0	4
Bourbousson 1	CB1-8	C	RURAL	IA1/2	2500 - 2450	0	0	0	0	0	0	1	0	0	0	0	0	0	0	0	0	0	0	0	0	0	0	0	0	0	0	0	143
Bourbousson 3	CB3-10	C	RURAL	ERo	1825 - 1750	0	0	3	0	0	0	0	0	0	0	0	0	0	0	0	0	0	0	0	2	0	0	0	4	0	0	0	155
Bourbousson 3	CB3-11	C	RURAL	LRo	1725 - 1700	0	0	50	0	0	0	0	0	0	0	0	0	0	0	0	0	0	0	0	0	0	0	2	0	0	0	0	123264
Bousquetas	PaBsq-3	C	RURAL	Neo	5000 - 4700	0	0	0	0	0	0	0	0	0	0	0	0	0	0	0	0	0	0	0	0	0	0	0	0	0	0	0	2007
Boussargues	ArgBou-3	C	RURAL	Neo	4700 - 4200	0	0	0	0	29	0	0	0	1517	0	0	0	0	3	1	16537	51	0	0	0	0	0	0	0	0	0	0	60
BPNL	BPNL-4	C	RURAL	BA	4200 - 3650	1	0	2	0	0	0	0	5	0	0	0	0	0	0	0	1834	0	0	0	0	0	1	18	0	0	0	0	2197
BPNL	BPNL-6	C	RURAL	BA	3450 - 3300	0	0	0	0	0	0	0	0	0	0	0	0	0	0	0	966	0	0	0	0	0	0	27	3	0	0	0	6985
BPNL	BPNL-8	C	RURAL	IA1/2	2525 - 2450	0	0	1	0	0	0	0	0	0	0	0	0	0	0	0	0	0	0	0	0	0	1	0	0	0	0	0	24
BPNL	BPNL-10	C	RURAL	ERo	2000 - 1800	0	0	2	0	0	0	0	0	0	0	0	0	0	0	0	0	0	0	0	0	0	0	0	0	0	0	0	0
BPNL	BPNL-11	C	RURAL	LRo	1700 - 1600	0	0	1	0	0	0	0	0	0	0	0	0	0	0	0	0	0	0	0	0	0	0	0	0	0	0	0	9
BPNL - funerary	BPNLfu-10	C	FUN	ERo	2027 - 1950	0	0	0	0	0	0	0	0	0	0	0	0	0	0	0	0	0	0	0	0	0	4	0	0	0	0	0	13
BPNL - funerary	BPNLfu-10a	C	FUN	ERo	1890 - 1840	0	0	2	0	0	0	0	0	0	0	0	0	0	0	0	0	0	0	0	0	0	0	2	0	0	0	0	115
BPNL - funerary	BPNLfu-10b	C	FUN	ERo	1800 - 1750	0	0	19	0	0	0	0	0	0	0	0	0	0	0	0	0	0	0	0	0	0	0	0	0	0	0	0	545
Brassières Nord	MDBN-9a	C	RURAL	IA2	2150 - 2100	0	0	0	0	0	0	0	0	0	0	0	0	0	0	0	0	0	0	0	0	0	0	1	0	0	0	0	2
Brassières Nord	MDBN-9b	C	RURAL	IA2	2100 - 2050	0	0	0	0	0	0	0	0	0	0	0	0	0	0	0	0	0	0	0	0	0	0	0	0	0	0	0	16
Brassières Nord	MDBN-9c	C	RURAL	IA2	2050 - 2020	0	0	0	0	0	0	0	0	0	0	0	0	0	0	0	0	0	0	0	0	0	0	0	0	0	0	0	1
Brassières Nord	MDBN-10	C	RURAL	ERo	2020 - 1975	0	0	0	0	0	0	1	0	0	0	0	0	0	0	0	0	0	0	0	0	0	0	0	0	0	0	0	19
Brassières Sud	MDBS-7	C	RURAL	IA1	2750 - 2625	0	0	0	0	0	0	0	0	0	0	0	0	0	0	0	0	0	0	0	0	0	0	0	0	0	0	0	15
Brégoule	Brego-6	C	RURAL	BA	2800 - 2700	0	0	0	0	0	0	0	0	0	0	0	0	0	0	0	0	0	0	0	0	0	0	0	0	0	0	0	1263
Buffe Arnaud	BufAr-9	C	VILLAGE	IA2	2150 - 2100	0	0	1	0	0	0	0	0	0	0	0	0	0	0	0	0	0	0	0	0	0	0	0	0	0	0	0	25
Buffens (grotte de)	GtBuf-5	C	CAVE	BA	3650 - 3350	0	0	0	0	0	0	0	0	0	0	0	0	0	0	0	2	0	0	0	0	0	0	0	0	0	0	0	221
C et G (Grotte)	GtCG-2	C	CAVE	Neo	6000 - 5500	0	0	0	0	0	0	0	0	0	0	0	0	0	0	0	1	0	0	0	0	0	0	0	0	0	0	0	227
Cadenet	SeCad-10	C	RURAL	ERo	2000 - 1800	0	0	0	0	0	0	0	0	0	0	0	0	0	0	0	0	0	0	0	0	0	0	0	0	0	0	0	23004
Cadoules	MaCad-3	C	RURAL	Neo	5500 - 4200	0	0	1	0	0	0	0	0	0	0	0	0	0	0	0	0	0	0	0	0	0	0	0	0	0	0	0	2
Cailar (Le)	Lcail-9a	C	RURAL	IA2	2500 - 2400	0	0	1	0	0	0	0	0	0	0	0	0	0	0	0	0	0	0	0	0	0	0	0	0	0	0	0	4807
Cailar (Le)	Lcail-9b	C	RURAL	IA2	2300 - 2200	0	0	84	0	0	0	0	1	0	0	0	0	0	0	0	0	0	0	0	0	0	0	0	0	0	0	0	796
Caisses Saint Jean	CStJea-9a	C	VILLAGE	IA2	2455 - 2400	0	0	14	2	0	0	0	0	0	0	0	0	0	0	0	0	0	0	0	0	0	0	0	0	0	0	0	34
Caisses Saint Jean	CStJea-9b	C	VILLAGE	IA2	2150 - 2100	0	0	6	0	0	0	0	0	0	0	0	0	0	0	0	0	0	0	0	0	0	0	0	0	0	0	0	7
Calade	Calad-10	C	FUN	ERo	1960 - 1750	0	0	0	0	0	0	0	0	0	0	0	0	0	0	0	0	0	0	0	0	0	0	0	0	0	0	0	0
Camelin (Le)	FrCam-10a	C	URBAN	ERo	2040 - 1900	2	0	3	0	0	0	0	0	0	0	0	0	0	0	0	2	0	0	0	0	0	0	0	0	0	0	0	12
Camelin (Le)	FrCam-10b	C	URBAN	ERo	1900 - 1800	0	0	1	0	0	0	0	0	0	0	0	0	0	0	0	0	0	0	0	0	0	0	0	0	0	0	0	1
Camelin (Le)	FrCam-10b	U	URBAN	ERo	1900 - 1800	0	0	121	60	0	0	0	0	0	0	0	0	0	0	0	0	0	0	0	70	0	0	0	0	0	0	0	1
Camp de la Torre	LPCTo-9	C	RURAL	IA2	2200 - 2075	0	0	1	0	0	0	0	0	0	0	0	0	0	0	0	0	0	0	0	0	0	0	0	0	0	0	0	25
Camp del Viver	CaViv-6	C	RURAL	BA	3100 - 2920	0	0	0	0	0	0	0	0	0	0	0	0	0	0	0	0	0	0	0	0	0	0	3	0	0	0	0	14
Capelles (Lac de)	LaCap-10	C	RURAL	ERo	2030 - 1990	0	0	0	0	0	0	0	0	0	0	0	0	0	0	0	0	0	0	0	0	0	0	0	0	0	0	0	2
Capelles (Lac de)	LaCap-10	U	RURAL	ERo	2030 - 1990	0	0	1515	20	0	0	0	0	0	0	0	0	0	1	1	38	0	2	0	0	0	4	0	0	1	0	0	31
Careiron & Pesquier	C&Pesq-11	C	RURAL	LRo	1625 - 1600	0	0	44	0	0	0	0	0	0	0	0	0	0	0	0	0	0	0	0	0	0	0	0	0	0	0	0	389
Careiron & Pesquier	C&Pesq-11	U	RURAL	LRo	1625 - 1600	0	0	60	6	0	0	0	0	0	0	0	0	0	0	0	0	0	0	0	0	0	4	0	0	0	0	0	73
Cariaux	CrxFro-10	C	OFFSITE	ERo	2000 - 1770	0	0	0	0	0	0	0	0	0	0	0	0	0	0	0	0	0	0	0	0	0	0	0	0	0	0	0	10
Cariaux	CrxFro-10	U	OFFSITE	ERo	2000 - 1770	0	0	134	0	0	0	54	0	0	0	3	0	0	0	20	10	0	6	2	129	0	0	202	108	0	0	1	0
Carrerrassa (La)	Carre-4	C	RURAL	BA	4200 - 3650	0	0	0	0	0	0	0	0	0	0	0	0	0	0	0	0	0	0	0	0	0	0	0	0	0	0	0	59
Carsac	Carsa-6	C	VILLAGE	BA	3050 - 2750	0	0	0	0	0	0	0	0	0	0	0	0	0	0	0	0	0	0	0	0	0	0	0	0	0	0	0	5
Carsac	Carsa-7	C	VILLAGE	IA1	2750 - 2450	0	0	0	0	0	0	0	0	0	0	0	0	0	0	0	0	0	0	0	0	0	0	0	0	0	0	0	275
Castellan	CastIs-9	C	VILLAGE	IA2	2200 - 2100	0	0	776	3	0	0	0	0	0	0	0	0	0	0	0	0	0	0	0	0	0	0	0	0	0	0	0	0
Castellas	CasRo-9	C	VILLAGE	IA2	2150 - 2050	0	0	0	0	0	0	0	0	0	0	0	0	0	0	0	0	0	0	0	0	0	0	0	0	0	0	0	0
Castelle et Fromigue - funerary	CstFro-10	C	FUN	ERo	2000 - 1800	0	0	94	1	0	0	0	0	0	0	0	0	0	0	0	0	0	0	0	0	0	0	0	0	0	0	0	0
Castelle GR	CstGR-10	C	RURAL	ERo	1900 - 1700	0	0	0	0	0	0	0	0	0	0	0	0	0	0	0	0	0	0	0	3	0	0	0	0	0	0	0	0
Castelle GR	CstGR-10	U	RURAL	ERo	1900 - 1700	0	0	1357	177	0	0	0	0	0	0	0	0	0	2	2	0	0	0	0	8	0	0	15	0	0	0	0	22
Castelle-Pahon-Pinède - funerary	CstPP-10	C	FUN	ERo	2000 - 1800	0	0	254	0	0	0	0	0	0	0	0	0	0	0	0	0	0	0	0	0	0	0	0	0	0	0	0	0
Cazals (Grotte de)	GtCaz-5	C	CAVE	BA	3740 - 3535	0	0	0	0	0	0	0	0	0	0	0	0	0	0	0	0	0	0	0	0	0	0	0	0	0	0	0	9265
Centre des Impôts	CDImp-3	C	RURAL	Neo	4700 - 4400	0	0	0	0	0	0	0	0	0	0	0	0	0	0	0	1	0	0	0	0	0	0	0	2	0	0	0	18
Ceron	VeCero-1	C	RURAL	Neo	7000 - 6500	0	0	0	0	0	0	0	0	0	0	0	0	0	0	0	0	0	0	0	0	0	0	0	0	0	0	0	2
Champ du Perrier (ZAC du)	ChdPe-7	C	RURAL	IA1	2850 - 2600	0	0	0	0	0	0	0	0	0	0	0	0	0	0	0	0	0	0	0	0	0	0	0	0	0	0	0	42
Champ du Poste (Le)	ChdPo-2	C	RURAL	Neo	6500 - 6000	0	0	0	0	1	0	0	0	0	0	0	0	0	0	0	1	0	0	0	0	0	0	0	0	0	0	0	134

Site	Cod Sit-Phas	Pres	type	Period	DATE BP	Quantif	MNI	Citrul	Cumel	Cupre	Cusat	Cusp	Jugla	Lage	Mespi	Moru	Prcer	Prdul	Prpers	Punic	Zizip	Cordi	Phoen	Celti	Coryl	Ficu	Fraga	Maloid	Malu	Olea	Pinpi	Pravce	Pravi	Prdom
Champ Lamet III	PdCCL3-2	C	RURAL	Neo	6000 - 5500	NMI	843	0	0	0	0	0	0	0	0	0	0	0	0	0	0	0	0	0	0	0	0	0	0	0	0	0	0	0
Champ Lamet III	PdCCL3-5	C	RURAL	BA	3700 - 3500	NMI	2	0	0	0	0	0	0	0	0	0	0	0	0	0	0	0	0	0	0	0	0	0	0	0	0	0	0	0
Champs (Les)	Champ-7	C	RURAL	IA1	2750 - 2450	NMI	78	0	0	0	0	0	0	0	0	0	0	0	0	0	0	0	0	0	0	0	0	0	0	0	0	0	0	0
Charnage (Rue de)	CLChar-6	C	RURAL	BA	3400 - 2800	NMI	426	0	0	0	0	0	0	0	0	0	0	0	0	0	0	0	0	0	0	0	0	0	0	0	0	0	0	0
Château Royal	CerCR-11	C	RURAL	LRo	1550 - 1450	NMI	3718	0	0	0	0	0	0	0	0	0	0	0	0	0	0	0	0	0	0	0	0	0	0	0	0	0	0	0
Château-l'Arc	ChArc-2	C	RURAL	Neo	6500 - 5500	NMI	4	0	0	0	0	0	0	0	0	0	0	0	0	0	0	0	0	0	0	0	0	0	0	0	0	0	0	0
Châtillon	ChCha-6	C	RURAL	BA	2906 - 2814	P/A	11	0	0	0	0	0	0	0	0	0	0	0	0	0	0	0	0	0	0	0	0	0	1	0	0	0	0	0
Chemin Saint Pierre	LtCSP-9	C	FUN	IA2	2500 - 2300	NMI	11	0	0	0	0	0	0	0	0	0	0	0	0	0	0	0	0	0	7	0	0	0	0	0	0	0	0	0
Chomette (La)	CoCho-5	C	RURAL	BA	3700 - 3500	NMI	9	0	0	0	0	0	0	0	0	0	0	0	0	0	0	0	0	0	0	0	0	0	0	0	0	0	0	0
Christol 3	Chri3-8	C	RURAL	IA1/2	2525 - 2450	P/A	10	0	0	0	0	0	0	0	0	0	0	0	0	0	0	0	0	0	0	0	0	0	0	0	0	0	0	0
Claparouse	Clapa-2	C	RURAL	Neo	6000 - 5500	NMI	10513	0	0	0	0	0	0	0	0	0	0	0	0	0	0	0	0	0	0	0	0	0	0	0	0	0	0	0
Clapiers	VBzCl-10	C	FUN	ERo	2025 - 1750	P/A	2	0	0	0	0	0	0	0	0	0	0	0	0	0	0	0	0	0	0	0	0	0	0	0	0	0	0	0
Cloche (La)	Cloch-9	C	VILLAGE	IA2	2100 - 2050	NMI	49	0	0	0	0	0	0	0	0	0	0	0	0	0	0	0	0	0	0	0	0	0	0	49	0	0	0	0
Clos de la Lombarde	ClLomb-10a	U	URBAN	ERo	2000 - 1900	NMI	17	0	0	0	0	0	3	0	0	0	0	0	0	0	0	0	0	0	3	0	0	0	0	3	6	2	0	0
Clos de la Lombarde	ClLomb-10b	U	URBAN	ERo	1900 - 1800	NMI	40	0	0	0	0	0	8	0	0	0	0	0	11	0	0	0	0	0	2	0	0	0	0	13	4	2	0	0
Clos de la Lombarde	ClLomb-10c	C	URBAN	ERo	1800 - 1700	NMI	282	0	0	0	0	0	0	0	0	0	0	0	0	0	0	0	0	0	0	0	0	0	0	0	0	0	0	0
Clos de la Lombarde	ClLomb-10c	U	URBAN	ERo	1800 - 1700	NMI	1946	0	0	0	9	0	187	0	0	0	0	0	32	0	0	6	0	1	48	15	0	0	2	579	76	81	0	7
Closeraie (La)	OrClo-10	C	FUN	ERo	2010 - 1800	NMI	2492	0	0	0	0	0	27	0	0	0	0	460	0	0	0	0	110	0	363	939	0	0	0	20	229	1	0	3
Colline Saint Jacques	CaStJ-9	C	VILLAGE	IA2	2125 - 2000	NMI	2	0	0	0	0	0	0	0	0	0	0	0	0	0	0	0	0	0	0	1	0	0	0	0	0	0	0	0
Collombières (Les)	Collo-9	C	RURAL	IA2	2370 - 2172	NMI	2	0	0	0	0	0	0	0	0	0	0	0	0	0	0	0	0	0	0	0	0	0	0	0	0	0	0	0
Collombières (Les)	Collo-10	C	RURAL	ERo	2025 - 1750	NMI	1	0	0	0	0	0	0	0	0	0	0	0	0	0	0	0	0	0	0	0	0	0	0	0	0	0	0	0
Colombier (Le)	BClmb-2	C	RURAL	Neo	6000 - 5500	NMI	1487	0	0	0	0	0	0	0	0	0	0	0	0	0	0	0	0	0	11	0	0	0	0	0	0	0	0	0
Colomina d'en Maurell	OrCEM-9	C	RURAL	IA2	2360 - 2200	NMI	13	0	0	0	0	0	0	0	0	0	0	0	0	0	0	0	0	0	0	0	0	0	0	0	0	0	0	0
Combe Grèze	CbGrz-1	C	CAVE	Neo	8000 - 6500	NMI	3	0	0	0	0	0	0	0	0	0	0	0	0	0	0	0	0	0	3	0	0	0	0	0	0	0	0	0
Condamine (La)	Conda-9	C	RURAL	IA2	2500 - 2400	P/A	6	0	0	0	0	0	0	0	0	0	0	0	0	0	0	0	0	0	1	0	0	0	0	0	0	0	0	0
Condamine 7	Cond7-7	C	RURAL	IA1	2525 - 2475	NMI	19	0	0	0	0	0	0	0	0	0	0	0	0	0	0	0	0	0	0	0	0	0	0	0	0	0	0	0
Conesa (Place)	APlCo-8	C	URBAN	IA1/2	2525 - 2400	NMI	429	0	0	0	0	0	0	0	0	0	0	1	0	0	0	0	0	0	0	0	0	0	0	0	0	0	0	0
Conesa (Place)	APlCo-9	C	URBAN	IA2	2400 - 2300	NMI	45	0	0	0	0	0	0	0	0	0	0	0	0	0	0	0	0	0	1	3	0	0	0	0	0	0	0	0
Conque (La)	LConq-6	U	RURAL	BA	3200 - 2900	NMI	12	0	0	0	0	0	0	0	0	0	0	0	0	0	0	0	0	0	1	2	0	0	0	0	0	0	0	0
Contrescarpe (Rue de)	LyCont-10	C	FUN	ERo	1950 - 1900	NMI	10	0	0	0	0	0	3	0	0	0	0	0	0	0	0	0	0	0	0	0	0	0	0	0	2	0	0	0
Corbières (Rue des)	RuCor-9a	C	VILLAGE	IA2	2400 - 2300	NMI	119	0	0	0	0	0	0	0	0	0	0	0	0	0	0	0	0	0	1	7	0	0	0	0	0	0	0	0
Corbières (Rue des)	RuCor-9b	C	VILLAGE	IA2	2200 - 2100	NMI	27	0	0	0	0	0	0	0	0	0	0	0	0	0	0	0	0	0	0	1	0	0	0	0	0	1	0	0
Cordouls (Les)	PuyCor-6	C	VILLAGE	BA	2900 - 2750	NMI	11286	0	0	0	0	0	0	0	0	0	0	0	0	0	0	0	0	0	0	0	0	0	0	0	0	0	0	0
Cordouls (Les)	PuyCor-7	C	VILLAGE	IA1	2700 - 2600	NMI	294	0	0	0	0	0	0	0	0	0	0	0	0	0	0	0	0	0	1	0	0	0	0	0	0	0	0	0
Cordouls (Les)	PuyCor-9	C	VILLAGE	IA2	2500 - 2400	NMI	37	0	0	0	0	0	0	0	0	0	0	0	0	0	0	0	0	0	0	0	0	0	0	0	0	0	0	0
Cordouls (Les)	PuyCor-Ro	C	VILLAGE	Rom	2025 - 1500	NMI	1611	0	0	0	0	0	0	0	0	0	0	0	0	0	0	0	0	0	0	0	0	0	0	0	0	0	0	0
Corent	VMCor-6	C	VILLAGE	BA	2950 - 2800	NMI	271	0	0	0	0	0	0	0	0	0	0	0	0	0	0	0	0	0	0	1	0	0	0	0	0	0	0	0
Corent	VMCor-7	C	RURAL	IA1	2550 - 2510	NMI	98	0	0	0	0	0	0	0	0	0	0	0	0	0	0	0	0	0	0	0	0	0	0	0	0	0	0	0
Corent	VMCor-9	C	VILLAGE	IA2	2140 - 2050	NMI	146	0	0	0	0	0	0	0	0	0	0	0	0	0	0	0	0	0	15	0	0	0	0	0	0	0	0	0
Corent	VMCor-10	C	RURAL	ERo	2050 - 1700	NMI	43	0	0	0	0	0	1	0	0	0	0	0	0	0	0	0	0	0	2	0	0	0	0	0	0	0	0	0
Cormail, Locus 3	CoLoc3-2	C	RURAL	Neo	6000 - 5500	NMI	978	0	0	0	0	0	0	0	0	0	0	0	0	0	0	0	0	0	6	0	0	0	0	0	0	0	0	0
Côtes de Roquefort	CtRoq-3	C	CAVE	Neo	4800 - 4200	NMI	223	0	0	0	0	0	0	0	0	0	0	0	0	0	0	0	0	0	0	0	0	0	0	0	0	0	0	0
Coudoumines 1365B (Les)	CaCoud-1	C	RURAL	Neo	6500 - 6200	NMI	598	0	0	0	0	0	0	0	0	0	0	0	0	0	0	0	0	0	0	0	0	0	0	0	0	0	0	0
Coudoumines 541 (Les)	CaCoud-11	C	RURAL	LRo	1630 - 1500	NMI	3	0	0	0	0	0	0	0	0	0	0	0	0	0	0	0	0	0	0	0	0	0	0	0	0	0	0	0
Coudouneu	Coudo-9	C	VILLAGE	IA2	2425 - 2400	NMI	9476	0	0	0	0	0	0	0	0	0	0	0	0	0	0	0	0	0	0	1	0	0	0	0	0	0	0	0
Cougourlude (La)	LtCog-7	U	RURAL	IA1	2550 - 2475	NMI	3337	0	0	0	0	0	0	0	0	0	0	0	0	0	0	0	0	0	0	2628	0	0	0	0	0	0	0	0
Cougourlude (La)	LtCog-7	C	VILLAGE	IA1	2550 - 2475	NMI	2242	0	0	0	0	0	0	0	0	0	0	0	0	0	0	0	0	0	0	1	0	0	0	0	0	0	0	0
Coumo dal Cat	CoCat-7	C	RURAL	IA1	2600 - 2500	NMI	20217	0	0	0	0	0	0	0	0	0	0	0	0	0	0	0	0	0	0	0	0	0	0	0	0	0	0	0
Courtinals	Courti-8	C	RURAL	IA1/2	2450 - 2450	%	100	0	0	0	0	0	0	0	0	0	0	0	0	0	0	0	0	0	0	0	0	0	0	0	0	0	0	0
Cova de l'Esperit	CoEsp-1	C	CAVE	Neo	7400 - 7000	NMI	19	**0**	0	0	0	0	0	**0**	0	0	0	0	0	0	0	**0**	0	**0**	**0**	0	0	0	**0**	0	0	0	**0**	0
Crès (Le)	BzCrs-2	C	RURAL	Neo	6500 - 6000	NMI	357	0	0	0	0	0	0	0	0	0	0	0	0	0	0	0	0	0	0	0	0	0	0	0	0	0	0	0
Croix Saint Etienne (La)	CxStE-3	C	RURAL	Neo	5500 - 4200	NMI	6	0	0	0	0	0	0	0	0	0	0	0	0	0	0	0	0	0	0	0	0	0	0	0	0	0	0	0
Derrière le Château	DeCha-11	C	RURAL	LRo	1700 - 1500	NMI	46	0	0	0	0	0	0	0	0	0	0	0	0	0	0	0	0	0	0	0	0	0	0	0	0	0	0	0
Doulouzargues	Doulz-9	C	RURAL	IA2	2300 - 2200	NMI	17	0	0	0	0	0	0	0	0	0	0	0	0	0	0	0	0	0	0	0	0	0	0	0	0	0	0	0
Dourgne (Roc de)	Dourg-1	C	CAVE	Neo	7000 - 6500	NMI	5	0	0	0	0	0	0	0	0	0	0	0	0	0	0	0	0	0	5	0	0	0	0	0	0	0	0	0
Encombres	Enc40-2	C	RURAL	Neo	6500 - 6000	NMI	31	0	0	0	0	0	0	0	0	0	0	0	0	0	0	0	0	0	0	0	0	0	0	0	0	0	0	0
Escarpes 2	Escp2-10	C	RURAL	ERo	2000 - 1900	NMI	84	0	0	0	0	0	0	0	0	0	0	0	0	0	0	0	0	0	0	0	0	0	0	0	0	0	0	0
Escarpes 2	Escp2-11	C	RURAL	LRo	1650 - 1500	NMI	19	0	0	0	0	0	0	0	0	0	0	0	0	0	0	0	0	0	0	0	0	0	0	0	0	0	0	0
Estagnon	Estag-10	U	VILLAGE	ERo	1950 - 1920	NMI	900	0	0	0	0	0	1	0	0	1	0	0	0	0	0	0	0	0	0	143	0	0	0	718	0	0	0	2
Fangade (La)	LFang-5	C	RURAL	BA	3450 - 3250	NMI	128	0	0	0	0	0	0	0	0	0	0	0	0	0	0	0	0	0	0	0	0	0	0	0	0	0	0	0
Fangade (La)	LFang-5	U	RURAL	BA	3450 - 3250	NMI	4894	0	0	0	0	0	0	0	0	0	0	0	0	0	0	0	0	0	1	762	0	0	2	0	0	0	0	0
Fangade (La)	LFang-6b	C	RURAL	BA	3200 - 3050	NMI	322	0	0	0	0	0	0	0	0	0	0	0	0	0	0	0	0	0	0	0	0	0	0	0	0	0	0	0
Fangade (La)	LFang-6b	U	RURAL	BA	3200 - 3050	NMI	5486	0	0	0	0	0	0	0	0	0	0	0	0	0	0	0	0	0	1	1400	0	0	0	0	0	0	0	0

Site	Cod Sit-Phas	Pres	type	Period	DATE BP	Prsp	Pyru	Viti	Vitipd	Arbu	Corma	Corsa	Crata	Junsp	Myrt	Physa	Pincem	Pinsp	Pista	Prspi	Querc	Rhus	Rosa	Rubca	Rubfr	Rubid	Rubsp	Sambeb	Sambni	Sambsp	Sorb	Vibu	An crops
Champ Lamet III	PdCCL3-2	C	RURAL	Neo	6000 - 5500	0	0	0	0	0	0	0	0	0	0	0	0	0	0	1	0	0	0	0	0	0	0	0	0	0	0	0	842
Champ Lamet III	PdCCL3-5	C	RURAL	BA	3700 - 3500	0	0	0	0	0	0	0	0	0	0	0	0	0	0	0	0	0	0	0	0	0	0	0	0	0	0	0	2
Champs (Les)	Champ-7	C	RURAL	IA1	2750 - 2450	0	0	1	0	0	0	0	0	0	0	0	0	0	0	0	0	0	0	0	0	0	0	0	0	0	0	0	77
Charnage (Rue de)	CLChar-6	C	RURAL	BA	3400 - 2800	0	0	0	0	0	0	0	0	0	0	0	0	0	0	0	0	0	0	0	0	0	0	0	0	0	0	0	426
Château Royal	CerCR-11	C	RURAL	LRo	1550 - 1450	0	0	1	0	0	0	0	0	0	0	0	0	0	0	0	0	0	0	0	0	0	0	0	0	0	0	0	3717
Château-l'Arc	ChArc-2	C	RURAL	Neo	6500 - 5500	0	0	0	0	0	0	0	0	0	0	0	0	0	0	0	0	0	0	0	0	0	0	0	0	0	0	0	4
Châtillon	ChCha-6	C	RURAL	BA	2906 - 2814	0	0	1	0	0	0	0	0	0	0	0	0	0	0	0	0	0	0	0	0	0	0	0	0	0	0	0	9
Chemin Saint Pierre	LtCSP-9	C	FUN	IA2	2500 - 2300	0	0	4	0	0	0	0	0	0	0	0	0	0	0	0	0	0	0	0	0	0	0	0	0	0	0	0	0
Chomette (La)	CoCho-5	C	RURAL	BA	3700 - 3500	0	0	0	0	0	0	0	0	0	0	0	0	0	0	0	0	0	0	0	0	0	0	1	0	0	0	0	8
Christol 3	Chri3-8	C	RURAL	IA1/2	2525 - 2450	0	0	0	0	0	0	0	0	0	0	0	0	0	0	0	0	0	0	0	0	0	0	0	0	0	0	0	10
Claparouse	Clapa-2	C	RURAL	Neo	6000 - 5500	0	0	0	0	0	0	0	0	0	0	0	0	0	0	0	0	0	0	0	0	0	0	0	0	0	0	0	10513
Clapiers	VBzCl-10	C	FUN	ERo	2025 - 1750	0	0	1	0	0	0	0	0	0	0	0	0	0	0	0	0	0	0	0	0	0	0	0	0	0	0	0	1
Cloche (La)	Cloch-9	C	VILLAGE	IA2	2100 - 2050	0	0	0	0	0	0	0	0	0	0	0	0	0	0	0	0	0	0	0	0	0	0	0	0	0	0	0	0
Clos de la Lombarde	ClLomb-10a	U	URBAN	ERo	2000 - 1900	0	0	0	0	0	0	0	0	0	0	0	0	0	0	0	0	0	0	0	0	0	0	0	0	0	0	0	0
Clos de la Lombarde	ClLomb-10b	U	URBAN	ERo	1900 - 1800	0	0	0	0	0	0	0	0	0	0	0	0	0	0	0	0	0	0	0	0	0	0	0	0	0	0	0	0
Clos de la Lombarde	ClLomb-10c	C	URBAN	ERo	1800 - 1700	0	0	0	0	0	0	0	0	0	0	0	0	0	0	0	0	0	0	0	0	0	0	0	0	0	0	0	282
Clos de la Lombarde	ClLomb-10c	U	URBAN	ERo	1800 - 1700	0	0	895	6	0	0	0	1	0	0	0	0	0	0	0	0	0	0	0	0	0	0	1	0	0	0	0	0
Closeraie (La)	OrClo-10	C	FUN	ERo	2010 - 1800	0	0	74	1	0	0	0	0	0	0	0	0	0	0	0	11	0	0	0	0	0	0	0	0	0	0	0	254
Colline Saint Jacques	CaStJ-9	C	VILLAGE	IA2	2125 - 2000	0	0	1	0	0	0	0	0	0	0	0	0	0	0	0	0	0	0	0	0	0	0	0	0	0	0	0	0
Collombières (Les)	Collo-9	C	RURAL	IA2	2370 - 2172	0	0	0	0	0	0	0	0	0	0	0	0	0	0	0	0	0	0	0	0	0	0	0	0	0	0	0	2
Collombières (Les)	Collo-10	C	RURAL	ERo	2025 - 1750	0	0	0	0	0	0	0	0	0	0	0	0	0	0	0	0	0	0	0	0	0	0	0	0	0	0	0	1
Colombier (Le)	BClmb-2	C	RURAL	Neo	6000 - 5500	0	0	0	0	0	0	0	0	0	0	0	0	0	0	0	0	0	0	0	0	0	0	0	0	0	0	0	1476
Colomina d'en Maurell	OrCEM-9	C	RURAL	IA2	2360 - 2200	0	0	0	0	0	0	0	0	0	0	0	0	0	0	4	0	0	0	0	0	0	0	0	0	0	0	0	9
Combe Grèze	CbGrz-1	C	CAVE	Neo	8000 - 6500	0	0	0	0	0	0	0	0	0	0	0	0	0	0	0	0	0	0	0	0	0	0	0	0	0	0	0	0
Condamine (La)	Conda-9	C	RURAL	IA2	2500 - 2400	0	0	1	0	0	0	0	0	0	0	0	0	0	0	0	1	0	0	0	0	0	0	0	0	0	0	0	3
Condamine 7	Cond7-7	C	RURAL	IA1	2525 - 2475	0	0	0	0	0	0	0	0	0	0	0	0	0	0	0	0	0	0	0	0	0	0	0	0	0	0	0	19
Conesa (Place)	APlCo-8	C	URBAN	IA1/2	2525 - 2400	0	0	17	1	0	0	1	0	0	0	0	0	0	0	0	0	0	0	0	0	0	0	0	0	0	0	0	409
Conesa (Place)	APlCo-9	C	URBAN	IA2	2400 - 2300	0	0	16	1	0	0	0	0	0	0	0	0	0	0	0	0	0	0	0	0	0	0	0	0	0	0	0	24
Conque (La)	LConq-6	U	RURAL	BA	3200 - 2900	0	0	0	0	0	0	0	0	0	0	0	0	0	1	0	0	0	0	0	5	0	0	0	0	1	0	0	2
Contrescarpe (Rue de)	LyCont-10	C	FUN	ERo	1950 - 1900	0	0	0	0	0	0	0	0	0	0	0	0	0	0	0	0	0	0	0	0	0	0	0	0	0	0	0	5
Corbières (Rue des)	RuCor-9a	C	VILLAGE	IA2	2400 - 2300	0	0	4	4	0	0	0	0	0	0	0	0	0	0	0	0	0	0	0	0	0	1	0	0	0	0	0	102
Corbières (Rue des)	RuCor-9b	C	VILLAGE	IA2	2200 - 2100	0	0	8	0	0	0	0	0	0	0	0	0	0	0	0	0	0	0	0	5	0	0	0	0	0	0	0	12
Cordouls (Les)	PuyCor-6	C	VILLAGE	BA	2900 - 2750	0	0	0	0	0	0	0	0	0	0	0	0	0	0	0	5	0	0	0	0	0	0	5	6	0	0	0	11270
Cordouls (Les)	PuyCor-7	C	VILLAGE	IA1	2700 - 2600	0	0	0	0	0	0	0	0	0	0	0	0	0	0	0	272	0	0	0	0	0	0	0	0	0	0	0	21
Cordouls (Les)	PuyCor-9	C	VILLAGE	IA2	2500 - 2400	0	0	0	0	0	0	0	0	0	0	0	0	0	0	0	8	0	0	0	0	0	0	0	0	0	0	0	29
Cordouls (Les)	PuyCor-Ro	C	VILLAGE	Rom	2025 - 1500	0	0	0	0	0	0	0	0	0	0	0	0	0	0	0	0	0	0	0	0	0	0	0	0	0	0	0	1611
Corent	VMCor-6	C	VILLAGE	BA	2950 - 2800	0	0	0	0	0	0	1	0	0	0	0	0	0	0	3	0	0	0	0	0	0	0	0	0	0	0	0	266
Corent	VMCor-7	C	RURAL	IA1	2550 - 2510	0	0	0	0	0	0	0	0	0	0	0	0	0	0	1	0	0	0	0	0	0	0	0	0	1	0	0	96
Corent	VMCor-9	C	VILLAGE	IA2	2140 - 2050	0	0	0	0	0	0	0	1	0	0	0	0	0	0	2	0	0	0	0	0	0	0	0	0	1	0	0	127
Corent	VMCor-10	C	RURAL	ERo	2050 - 1700	0	0	0	0	0	0	0	0	0	0	0	0	0	0	1	0	0	0	0	0	0	0	12	3	0	0	0	24
Cormail, Locus 3	CoLoc3-2	C	RURAL	Neo	6000 - 5500	0	0	0	0	0	0	3	2	0	0	0	0	0	0	0	0	0	0	0	1	0	0	0	0	0	0	0	966
Côtes de Roquefort	CtRoq-3	C	CAVE	Neo	4800 - 4200	0	0	0	0	0	0	0	0	0	0	0	0	0	0	0	12	0	0	0	0	0	0	0	0	0	0	0	211
Coudoumines 1365B (Les)	CaCoud-1	C	RURAL	Neo	6500 - 6200	0	0	0	0	100	0	0	0	1	0	0	0	0	0	0	1	0	0	0	0	0	0	0	0	0	0	0	496
Coudoumines 541 (Les)	CaCoud-11	C	RURAL	LRo	1630 - 1500	0	0	3	0	0	0	0	0	0	0	0	0	0	0	0	0	0	0	0	0	0	0	0	0	0	0	0	0
Coudouneu	Coudo-9	C	VILLAGE	IA2	2425 - 2400	0	0	955	0	0	0	0	0	0	0	0	0	0	0	0	0	0	0	0	0	0	0	0	0	0	0	0	8520
Cougourlude (La)	LtCog-7	U	RURAL	IA1	2550 - 2475	2	0	28	0	0	0	0	0	0	0	0	0	0	0	0	0	0	0	0	40	0	0	20	600	0	0	0	19
Cougourlude (La)	LtCog-7	C	VILLAGE	IA1	2550 - 2475	0	0	6	0	0	0	0	0	0	0	0	0	0	0	0	0	0	0	0	0	0	0	1	0	0	0	0	2234
Coumo dal Cat	CoCat-7	C	RURAL	IA1	2600 - 2500	0	0	3	0	0	0	0	0	0	0	0	0	0	0	0	0	0	0	0	0	0	0	0	0	0	0	0	20214
Courtinals	Courti-8	C	RURAL	IA1/2	2450 - 2450	0	0	0	0	0	0	0	0	0	0	0	0	0	0	0	0	0	0	0	0	0	0	0	0	0	0	0	100
Cova de l'Esperit	CoEsp-1	C	CAVE	Neo	7400 - 7000	0	0	**1**	0	**0**	0	**1**	0	1	0	0	0	**0**	15	0	1	**0**	**0**	0	**0**	0	**0**	**0**	**0**	**0**	**0**	**0**	0
Crès (Le)	BzCrs-2	C	RURAL	Neo	6500 - 6000	0	0	0	0	0	0	0	0	0	0	0	0	0	0	0	0	0	0	0	0	0	0	0	0	0	0	0	357
Croix Saint Etienne (La)	CxStE-3	C	RURAL	Neo	5500 - 4200	0	0	0	0	0	0	0	0	0	0	0	0	0	0	0	0	0	0	0	0	0	0	2	0	0	0	0	4
Derrière le Château	DeCha-11	C	RURAL	LRo	1700 - 1500	0	0	0	0	0	0	0	0	0	0	0	0	0	0	0	0	0	0	0	0	0	0	0	0	0	0	0	46
Doulouzargues	Doulz-9	C	RURAL	IA2	2300 - 2200	0	0	0	0	0	0	0	0	0	0	0	0	0	0	0	0	0	0	0	0	0	0	0	0	0	0	0	17
Dourgne (Roc de)	Dourg-1	C	CAVE	Neo	7000 - 6500	0	0	0	0	0	0	0	0	0	0	0	0	0	0	0	0	0	0	0	0	0	0	0	0	0	0	0	0
Encombres	Enc40-2	C	RURAL	Neo	6500 - 6000	0	0	0	0	0	0	0	0	0	0	0	0	0	0	0	3	0	0	0	0	0	0	0	0	0	0	0	28
Escarpes 2	Escp2-10	C	RURAL	ERo	2000 - 1900	0	0	1	0	0	0	0	0	0	0	0	0	0	0	0	0	0	0	0	0	0	0	0	0	0	0	0	83
Escarpes 2	Escp2-11	C	RURAL	LRo	1650 - 1500	0	0	19	0	0	0	0	0	0	0	0	0	0	0	0	0	0	0	0	0	0	0	0	0	0	0	0	0
Estagnon	Estag-10	U	VILLAGE	ERo	1950 - 1920	0	0	27	2	0	0	0	0	0	0	0	0	0	0	0	0	0	0	0	0	0	2	0	0	2	0	0	2
Fangade (La)	LFang-5	C	RURAL	BA	3450 - 3250	0	0	0	0	0	0	0	0	0	0	0	0	0	2	0	1	0	0	0	0	0	0	0	0	0	0	0	125
Fangade (La)	LFang-5	U	RURAL	BA	3450 - 3250	0	0	93	33	0	0	0	0	0	0	4	0	0	157	3	411	0	40	0	2282	0	0	6	0	0	0	0	1100
Fangade (La)	LFang-6b	C	RURAL	BA	3200 - 3050	0	0	2	0	0	0	0	0	0	0	0	0	0	0	0	2	0	0	0	0	0	0	0	0	0	0	0	318
Fangade (La)	LFang-6b	U	RURAL	BA	3200 - 3050	0	0	26	1	0	0	0	0	4	0	3	0	1	59	2	16	0	2	2	2838	0	0	4	0	0	0	0	1127

Site	Cod Sit-Phas	Pres	type	Period	DATE BP	Quantif	MNI	Citrul	Cumel	Cupre	Cusat	Cusp	Jugla	Lage	Mespi	Moru	Prcer	Prdul	Prpers	Punic	Zizip	Cordi	Phoen	Celti	Coryl	Ficu	Fraga	Maloid	Malu	Olea	Pinpi	Pravce	Pravi	Prdom
Fangade (La)	LFang-6a	C	RURAL	BA	3050 - 2900	NMI	31	0	0	0	0	0	0	0	0	0	0	0	0	0	0	0	0	0	0	0	0	0	0	0	0	0	0	0
Fangade (La)	LFang-6a	U	RURAL	BA	3050 - 2900	NMI	1719	0	0	0	0	0	0	0	0	0	0	0	0	0	0	0	0	0	0	625	0	0	0	0	0	0	0	0
Favorite	LyFav-10	C	FUN	ERo	2000 - 1800	NMI	618	0	0	0	0	0	0	0	0	0	0	0	0	0	0	0	15	0	1	2	0	0	4	3	0	0	0	0
Feuilly (ZAC)	ZAFeuil-1	C	RURAL	Neo	7300 - 6500	NMI	324	0	0	0	0	0	0	0	0	0	0	0	0	0	0	0	0	0	1	0	0	0	319	0	0	0	0	0
Feuilly (ZAC)	ZAFeuil-6	C	RURAL	BA	3200 - 2900	NMI	669	0	0	0	0	0	0	0	0	0	0	0	0	0	0	0	0	0	0	0	0	0	0	0	0	0	0	0
Feuilly (ZAC)	ZAFeuil-8	C	RURAL	IA1/2	2525 - 2400	NMI	564	0	0	0	0	0	0	0	0	0	0	0	0	0	0	0	0	0	9	0	0	0	0	0	0	0	0	0
Feuilly (ZAC)	ZAFeuil-9	C	RURAL	IA2	2425 - 2375	NMI	58	0	0	0	0	0	0	0	0	0	0	0	0	0	0	0	0	0	0	0	0	0	0	0	0	0	0	0
Foisses (Les)	BFoiss-2	C	RURAL	Neo	6000 - 5500	NMI	51	0	0	0	0	0	0	0	0	0	0	0	0	0	0	0	0	0	0	0	0	0	0	0	0	0	0	0
Font aux Pigeons	FtPig-1a	C	CAVE	Neo	7500 - 7000	NMI	26399	0	0	0	0	0	0	0	0	0	0	0	0	0	0	0	0	0	0	0	0	0	0	0	0	0	0	0
Font aux Pigeons	FtPig-1b	C	CAVE	Neo	7000 - 6500	NMI	7514	0	0	0	0	0	0	0	0	0	0	0	0	0	0	0	0	0	0	0	0	0	0	0	0	0	0	0
Font Juvénal	FtJu-1	C	CAVE	Neo	7000 - 6500	NMI	9	0	0	0	0	0	0	0	0	0	0	0	0	0	0	0	0	0	2	0	0	0	0	0	0	0	0	0
Font Juvénal	FtJu-2	C	CAVE	Neo	6500 - 6000	NMI	1678	0	0	0	0	0	0	0	0	0	0	0	0	0	0	0	0	0	1	0	0	0	0	0	0	0	0	0
Fourches (Aven des)	AvFch-6	C	CAVE	BA	3400 - 3250	P/A	10	0	0	0	0	0	0	0	0	0	0	0	0	0	0	0	0	0	0	0	0	0	0	0	0	0	0	0
G (Grotte)	GtCG-5	C	CAVE	BA	3650 - 2800	NMI	187	0	0	0	0	0	0	0	0	0	0	0	0	0	0	0	0	0	0	0	0	0	0	0	0	0	0	0
Gabache XVI	Gab16-5	C	RURAL	BA	3600 - 3350	NMI	20	0	0	0	0	0	0	0	0	0	0	0	0	0	0	0	0	0	0	0	0	0	0	0	0	0	0	0
Gabache XVI	Gab16-5	U	RURAL	BA	3600 - 3350	NMI	12	0	0	0	0	0	0	0	0	0	0	0	0	0	0	0	0	0	0	0	0	0	0	0	0	0	0	0
Gach	Gach-7	C	RURAL	IA1	2750 - 2450	P/A	3	0	0	0	0	0	0	0	0	0	0	0	0	0	0	0	0	0	0	0	0	0	0	0	0	0	0	0
Gandus (Les)	SFTPGa-6	C	VILLAGE	BA	2900 - 2750	P/A	15	0	0	0	0	0	0	0	0	0	0	0	0	0	0	0	0	0	0	0	0	0	1	0	0	0	0	0
Gardi	GoGard-8	C	VILLAGE	IA1/2	2550 - 2475	NMI	1931	0	0	0	0	0	0	0	0	0	0	0	0	0	0	0	0	0	0	0	0	0	0	0	0	0	0	0
Gardon (Grotte du)	GGard-1	C	CAVE	Neo	7300 - 6600	NMI	12	0	0	0	0	0	0	0	0	0	0	0	0	0	0	0	0	0	1	0	0	0	0	0	0	0	0	0
Gardon (Grotte du)	GGard-2	C	CAVE	Neo	6500 - 6250	NMI	5	0	0	0	0	0	0	0	0	0	0	0	0	0	0	0	0	0	0	0	0	0	0	0	0	0	0	0
Gardon (Grotte du)	GGard-5a	C	CAVE	BA	3600 - 3500	NMI	108	0	0	0	0	0	0	0	0	0	0	0	0	0	0	0	0	0	3	0	1	0	0	0	0	0	0	0
Gardon (Grotte du)	GGard-5b	C	CAVE	BA	3500 - 3450	NMI	18	0	0	0	0	0	0	0	0	0	0	0	0	0	0	0	0	0	0	0	0	0	0	0	0	0	0	0
Gardon (Grotte du)	GGard-6a	C	CAVE	BA	3450 - 3300	NMI	166	0	0	0	0	0	0	0	0	0	0	0	0	0	0	0	0	0	0	0	0	0	0	0	0	0	0	0
Gardon (Grotte du)	GGard-6b	C	CAVE	BA	3050 - 3000	NMI	39	0	0	0	0	0	0	0	0	0	0	0	0	0	0	0	0	0	0	0	0	0	0	0	0	0	0	0
Gardon (Grotte du)	GGard-9	C	CAVE	IA2	2300 - 2200	NMI	260	0	0	0	0	0	0	0	0	0	0	0	0	0	0	0	0	0	9	0	2	0	9	0	0	0	0	0
Garenne (La)	PeGar-6a	C	CAVE	BA	3050 - 2900	NMI	17	0	0	0	0	0	0	0	0	0	0	0	0	0	0	0	0	0	3	0	0	0	0	0	0	0	0	0
Garenne (La)	PeGar-6b	C	CAVE	BA	2900 - 2750	NMI	353	0	0	0	0	0	0	0	0	0	0	0	0	0	0	0	0	0	6	0	0	0	0	0	0	0	0	0
Garennes (Les)	ToGar-7	C	RURAL	IA1	2550 - 2550	NMI	169	0	0	0	0	0	0	0	0	0	0	0	0	0	0	0	0	0	0	0	0	0	0	0	0	0	0	0
Gasquinoy	Gasq-10	C	RURAL	ERo	2000 - 1800	NMI	12	0	0	0	0	0	0	0	0	0	0	0	0	0	0	0	0	0	0	0	0	0	0	0	0	0	0	0
Gasquinoy	Gasq-10	U	RURAL	ERo	2000 - 1800	NMI	4524	0	0	0	0	0	94	0	0	0	0	0	14	0	0	0	0	0	7	89	0	2	0	48	7	8	0	10
Gazel (Grotte)	GtGaz-1	C	CAVE	Neo	7400 - 7000	NMI	21	0	0	0	0	0	0	0	0	0	0	0	0	0	0	0	0	0	2	0	0	0	0	0	0	0	0	0
Girardes (Les)	LaGird-10	C	RURAL	ERo	1900 - 1800	NMI	49	0	0	0	0	0	0	0	0	0	0	0	0	0	0	0	0	0	0	0	0	0	0	0	0	0	0	0
Goiffieux	Goiff-10	C	RURAL	ERo	2000 - 1900	NMI	268	0	0	0	0	0	1	0	0	0	0	0	0	0	0	0	0	0	1	0	0	0	0	0	1	0	0	0
Gorge de Loup	LyGdL-8	C	RURAL	IA1/2	2525 - 2450	P/A	5	0	0	0	0	0	0	0	0	0	0	0	0	0	0	0	0	0	0	0	0	0	0	0	0	0	0	0
Goulles (Les)	Goull-8	C	FUN	IA1/2	2440 - 2440	NMI	25	0	0	0	0	0	0	0	0	0	0	0	0	0	0	0	0	0	0	0	0	0	0	0	0	0	0	0
Gramière (La)	LGram-10	C	RURAL	ERo	2000 - 1700	NMI	14	0	0	0	0	0	0	0	0	0	0	0	0	0	0	0	0	0	0	0	0	0	0	2	0	0	0	0
Gramière (La)	LGram-11	C	RURAL	LRo	1750 - 1600	NMI	71	0	0	0	0	0	1	0	0	0	0	0	0	0	0	0	0	0	0	0	0	0	0	1	0	0	0	0
Grand Contras	GdCont-10	U	RURAL	ERo	2000 - 1800	NMI	49	0	0	0	0	0	0	0	0	0	0	0	0	0	0	0	0	0	0	1	0	0	0	0	0	0	0	0
Grande Borne (La)	GdBor-7	C	RURAL	IA1	2800 - 2650	NMI	2	0	0	0	0	0	0	0	0	0	0	0	0	0	0	0	0	0	0	0	0	0	0	0	0	0	0	0
Grande Chaberte (La)	GdChab-10a	C	RURAL	ERo	1975 - 1925	NMI	17	0	0	0	0	0	0	0	0	0	0	0	0	0	0	0	0	0	0	0	0	0	0	0	0	0	0	0
Grande Chaberte (La)	GdChab-10a	U	RURAL	ERo	1975 - 1925	NMI	70052	0	0	19	0	0	16	0	0	0	0	0	0	0	0	0	0	0	4	2452	0	0	0	1002	56	1	0	1
Grande Chaberte (La)	GdChab-10b	C	RURAL	ERo	1900 - 1700	NMI	10126	0	0	0	0	0	0	0	0	0	0	0	0	0	0	0	0	0	0	0	0	0	0	0	0	0	0	0
Grande Chaberte (La)	GdChab-11b	C	RURAL	LRo	1700 - 1500	NMI	1	0	0	0	0	0	0	0	0	0	0	0	0	0	0	0	0	0	0	0	0	0	0	0	0	0	0	0
Grande Chaberte (La) - funerary	GdChab-11a	C	FUN	LRo	1800 - 1650	NMI	1	0	0	0	0	0	0	0	0	0	0	0	0	0	0	0	0	0	0	0	0	0	0	0	0	0	0	0
Grande Rivoire (La)	GdRiv-1	C	CAVE	Neo	7300 - 6500	NMI	25	0	0	0	0	0	0	0	0	0	0	0	0	0	0	0	0	0	14	0	0	0	0	0	0	0	0	0
Grande Rivoire (La)	GdRiv-2a	C	CAVE	Neo	6500 - 6000	NMI	63	0	0	0	0	0	0	0	0	0	0	0	0	0	0	0	0	0	12	0	0	0	1	0	0	0	0	0
Grande Rivoire (La)	GdRiv-2b	C	CAVE	Neo	6000 - 5500	NMI	65	0	0	0	0	0	0	0	0	0	0	0	0	0	0	0	0	0	31	0	0	0	0	0	0	0	0	0
Grande Rivoire (La)	GdRiv-9	C	CAVE	IA2	2100 - 2000	NMI	229	0	0	0	0	0	172	0	0	0	0	0	0	0	0	0	0	0	13	0	0	0	0	0	0	0	0	0
Grande Terre	GdTer-7	C	RURAL	IA1	2500 - 2400	NMI	10	0	0	0	0	0	0	0	0	0	0	0	0	0	0	0	0	0	1	0	0	0	0	0	0	0	0	0
Grange Rouge	QuGRo-6	C	RURAL	BA	3250 - 2900	NMI	192	0	0	0	0	0	0	0	0	0	0	0	0	0	0	0	0	0	0	0	0	0	0	0	0	0	0	0
Grange Rouge	QuGRo-7	C	RURAL	IA1	2700 - 2525	NMI	125	0	0	0	0	0	0	0	0	0	0	0	0	0	0	0	0	0	0	0	0	0	0	0	0	0	0	0
Gravette (La)	LaGrav-6	C	RURAL	BA	2950 - 2950	NMI	3	0	0	0	0	0	0	0	0	0	0	0	0	0	0	0	0	0	0	0	0	0	0	0	0	0	0	0
Gravette (La)	LaGrav-7	C	RURAL	IA1	2525 - 2500	NMI	10	0	0	0	0	0	0	0	0	0	0	0	0	0	0	0	0	0	0	0	0	0	0	0	0	0	0	0
Grésine Est	GRSE-6	C	RURAL	BA	2905 - 2850	NMI	7109	0	0	0	0	0	0	0	0	0	0	0	0	0	0	0	0	0	1	0	0	0	0	0	0	0	0	0
Grésine Est	GRSE-6	U	RURAL	BA	2905 - 2850	NMI	14114	0	0	0	0	0	0	0	0	0	0	0	0	0	0	0	0	0	71	0	2346	0	271	0	0	0	15	0
Grésine Ouest	GRSW-6	C	RURAL	BA	2905 - 2850	NMI	5295	0	0	0	0	0	0	0	0	0	0	0	0	0	0	0	0	0	0	0	0	0	22	0	0	0	0	0
Grésine Ouest	GRSW-6	U	RURAL	BA	2905 - 2850	NMI	11590	0	0	0	0	0	0	0	0	0	0	0	0	0	0	0	0	0	99	0	1436	0	302	0	0	0	9	0
Guérine	Gueri-10	C	FUN	ERo	1925 - 1875	NMI	15	0	0	0	0	0	0	0	0	0	0	0	0	0	0	0	8	0	0	0	0	0	0	0	0	0	0	5
Guillemot-Labouygue	MoGLa-9	C	VILLAGE	IA2	2125 - 2075	NMI	0	0	0	0	0	0	0	0	0	0	0	0	0	0	0	0	0	0	0	0	0	0	0	0	0	0	0	0
Guillemot-Labouygue	MoGLa-10	C	VILLAGE	ERo	2000 - 1900	NMI	2	0	0	0	0	0	0	0	0	0	0	0	0	0	0	0	0	0	0	0	0	0	0	0	0	0	0	0
Hasard (Grotte du)	GtHas-6	C	CAVE	BA	3200 - 3050	NMI	335	0	0	0	0	0	0	0	0	0	0	0	0	0	0	0	0	0	0	0	0	0	0	0	0	0	0	0
Horand 2	LyHor2-10	C	FUN	ERo	2020 - 1750	NMI	3	0	0	0	0	0	0	0	0	0	0	0	0	0	0	0	0	0	2	0	0	0	0	0	0	0	0	1

Site	Cod Sit-Phas	Pres	type	Period	DATE BP	Prsp	Pyru	Viti	Vitipd	Arbu	Corma	Corsa	Crata	Junsp	Myrt	Physa	Pincem	Pinsp	Pista	Prspi	Querc	Rhus	Rosa	Rubca	Rubfr	Rubid	Rubsp	Sambeb	Sambni	Sambsp	Sorb	Vibu	An crops
Fangade (La)	LFang-6a	C	RURAL	BA	3050 - 2900	0	0	0	0	0	0	0	0	0	0	0	0	0	0	0	2	0	0	0	0	0	0	0	0	0	0	0	29
Fangade (La)	LFang-6a	U	RURAL	BA	3050 - 2900	0	0	8	3	0	0	0	0	0	0	0	0	0	52	0	21	0	0	0	739	0	0	0	0	1	0	0	270
Favorite	LyFav-10	C	FUN	ERo	2000 - 1800	0	0	19	0	0	0	0	0	0	0	0	0	0	0	2	0	0	0	0	0	0	0	0	0	0	0	0	572
Feuilly (ZAC)	ZAFeuil-1	C	RURAL	Neo	7300 - 6500	0	0	0	0	0	0	0	0	0	0	0	0	0	0	0	0	0	0	0	0	0	0	0	0	0	0	0	4
Feuilly (ZAC)	ZAFeuil-6	C	RURAL	BA	3200 - 2900	0	0	1	0	0	0	1	0	0	0	0	0	0	0	0	587	0	0	0	0	0	0	2	2	0	0	0	77
Feuilly (ZAC)	ZAFeuil-8	C	RURAL	IA1/2	2525 - 2400	0	0	0	0	0	0	0	0	0	0	0	0	0	0	0	0	0	0	0	0	0	0	0	0	0	0	0	555
Feuilly (ZAC)	ZAFeuil-9	C	RURAL	IA2	2425 - 2375	0	0	0	0	0	0	0	0	0	0	0	0	0	0	0	2	0	0	0	4	0	0	0	0	0	0	0	52
Foisses (Les)	BFoiss-2	C	RURAL	Neo	6000 - 5500	0	0	0	0	0	0	0	0	0	0	0	0	0	0	0	0	0	0	0	0	0	0	0	0	0	0	0	51
Font aux Pigeons	FtPig-1a	C	CAVE	Neo	7500 - 7000	0	0	0	0	0	0	0	0	0	0	0	0	0	49	0	0	0	0	0	0	0	0	0	0	0	0	0	26350
Font aux Pigeons	FtPig-1b	C	CAVE	Neo	7000 - 6500	0	0	0	0	0	0	0	0	0	0	0	0	0	3	0	0	0	0	0	0	0	0	0	0	0	0	0	7511
Font Juvénal	FtJu-1	C	CAVE	Neo	7000 - 6500	0	0	0	0	0	0	1	0	0	0	0	0	0	0	0	1	0	0	0	0	0	0	0	0	0	0	0	5
Font Juvénal	FtJu-2	C	CAVE	Neo	6500 - 6000	0	0	0	0	0	0	0	0	0	0	0	0	0	0	0	19	0	0	0	0	0	0	0	0	0	0	0	1658
Fourches (Aven des)	AvFch-6	C	CAVE	BA	3400 - 3250	0	0	0	0	0	0	0	0	0	0	0	0	0	0	0	0	0	0	0	0	0	0	0	0	0	0	0	10
G (Grotte)	GtCG-5	C	CAVE	BA	3650 - 2800	0	0	0	0	0	0	0	0	0	0	0	0	0	0	0	0	0	0	0	0	0	0	0	0	0	0	0	187
Gabache XVI	Gab16-5	C	RURAL	BA	3600 - 3350	0	0	0	0	0	0	0	0	0	0	0	0	0	0	0	0	0	0	0	0	0	0	0	0	0	0	0	20
Gabache XVI	Gab16-5	U	RURAL	BA	3600 - 3350	0	0	1	0	0	0	0	0	0	0	0	0	0	0	0	0	0	0	0	2	0	0	8	1	0	0	0	0
Gach	Gach-7	C	RURAL	IA1	2750 - 2450	0	0	0	0	0	0	0	0	0	0	0	0	0	0	0	3	0	0	0	0	0	0	0	0	0	0	0	0
Gandus (Les)	SFTPGa-6	C	VILLAGE	BA	2900 - 2750	0	0	0	0	0	1	0	0	0	0	0	0	0	0	0	0	0	0	0	0	0	0	0	0	0	0	0	13
Gardi	GoGard-8	C	VILLAGE	IA1/2	2550 - 2475	0	0	0	0	0	0	0	0	0	0	0	0	0	0	0	0	0	0	0	0	0	0	0	0	0	0	0	1931
Gardon (Grotte du)	GGard-1	C	CAVE	Neo	7300 - 6600	0	0	2	0	0	0	0	0	0	0	0	0	0	0	0	1	0	0	0	0	0	0	0	0	0	0	0	8
Gardon (Grotte du)	GGard-2	C	CAVE	Neo	6500 - 6250	0	0	0	0	0	0	0	0	0	0	0	0	0	0	0	1	0	0	0	0	0	0	0	0	0	0	0	4
Gardon (Grotte du)	GGard-5a	C	CAVE	BA	3600 - 3500	1	0	0	0	0	0	0	0	0	0	0	0	0	0	0	91	0	0	0	0	0	0	4	1	0	0	0	7
Gardon (Grotte du)	GGard-5b	C	CAVE	BA	3500 - 3450	0	0	0	0	0	0	0	0	0	0	0	0	0	0	0	6	0	0	0	0	0	0	2	0	0	0	0	10
Gardon (Grotte du)	GGard-6a	C	CAVE	BA	3450 - 3300	2	0	0	0	0	0	0	0	0	0	0	0	0	0	0	55	0	0	0	0	0	0	1	0	0	0	0	108
Gardon (Grotte du)	GGard-6b	C	CAVE	BA	3050 - 3000	0	0	0	0	0	0	0	0	0	0	0	0	0	0	0	0	0	0	0	0	0	0	0	0	0	0	0	39
Gardon (Grotte du)	GGard-9	C	CAVE	IA2	2300 - 2200	0	0	0	0	0	0	0	0	0	0	0	0	0	0	0	0	0	0	1	0	0	0	4	1	0	0	0	234
Garenne (La)	PeGar-6a	C	CAVE	BA	3050 - 2900	0	0	0	0	0	0	0	0	0	0	0	0	0	0	1	0	0	0	0	0	0	0	0	0	0	0	0	13
Garenne (La)	PeGar-6b	C	CAVE	BA	2900 - 2750	0	0	0	0	0	0	0	0	0	0	0	0	0	0	2	0	0	0	0	0	0	0	0	0	0	0	0	345
Garennes (Les)	ToGar-7	C	RURAL	IA1	2550 - 2550	0	0	0	0	0	0	0	0	0	0	0	0	0	0	0	0	0	0	0	0	0	0	0	0	0	0	0	169
Gasquinoy	Gasq-10	C	RURAL	ERo	2000 - 1800	0	0	11	0	0	0	0	0	0	0	0	0	0	0	0	0	0	0	0	0	0	0	0	0	0	0	0	1
Gasquinoy	Gasq-10	U	RURAL	ERo	2000 - 1800	0	0	3414	473	0	0	0	0	0	2	0	0	0	0	11	268	0	1	0	60	0	0	8	3	0	0	0	5
Gazel (Grotte)	GtGaz-1	C	CAVE	Neo	7400 - 7000	0	0	2	0	0	0	0	0	0	0	0	0	0	0	0	15	0	0	0	0	0	0	0	0	0	0	0	2
Girardes (Les)	LaGird-10	C	RURAL	ERo	1900 - 1800	0	0	2	0	0	0	0	0	0	0	0	0	0	0	0	0	0	0	0	0	0	0	0	0	0	0	0	47
Goiffieux	Goiff-10	C	RURAL	ERo	2000 - 1900	0	0	244	4	0	0	0	0	0	0	0	0	0	0	0	0	0	0	0	0	0	0	0	0	0	0	0	17
Gorge de Loup	LyGdL-8	C	RURAL	IA1/2	2525 - 2450	0	0	0	0	0	0	0	0	0	0	0	0	0	0	0	0	0	0	0	0	0	0	0	0	0	0	0	5
Goulles (Les)	Goull-8	C	FUN	IA1/2	2440 - 2440	0	0	0	0	0	0	0	0	0	0	0	0	0	0	0	22	0	0	0	0	0	0	0	0	0	0	0	3
Gramière (La)	LGram-10	C	RURAL	ERo	2000 - 1700	0	0	4	0	0	0	0	0	0	0	0	0	0	0	0	0	0	0	0	0	0	0	0	0	0	0	0	8
Gramière (La)	LGram-11	C	RURAL	LRo	1750 - 1600	0	0	31	0	0	0	0	0	0	0	0	0	0	0	0	0	0	0	0	0	0	0	0	0	0	0	0	38
Grand Contras	GdCont-10	U	RURAL	ERo	2000 - 1800	0	0	3	0	0	0	0	0	0	0	0	0	0	0	0	0	0	0	0	45	0	0	0	0	0	0	0	0
Grande Borne (La)	GdBor-7	C	RURAL	IA1	2800 - 2650	0	0	0	0	0	0	0	0	0	0	0	0	0	0	0	0	0	0	0	0	0	0	0	0	0	0	0	2
Grande Chaberte (La)	GdChab-10a	C	RURAL	ERo	1975 - 1925	0	0	17	0	0	0	0	0	0	0	0	0	0	0	0	0	0	0	0	0	0	0	0	0	0	0	0	0
Grande Chaberte (La)	GdChab-10a	U	RURAL	ERo	1975 - 1925	0	0	59275	7192	0	0	0	0	0	0	13	0	0	0	0	1	0	0	0	8	0	0	0	0	0	0	0	12
Grande Chaberte (La)	GdChab-10b	C	RURAL	ERo	1900 - 1700	0	0	5353	4773	0	0	0	0	0	0	0	0	0	0	0	0	0	0	0	0	0	0	0	0	0	0	0	0
Grande Chaberte (La)	GdChab-11b	C	RURAL	LRo	1700 - 1500	0	0	1	0	0	0	0	0	0	0	0	0	0	0	0	0	0	0	0	0	0	0	0	0	0	0	0	0
Grande Chaberte (La) - funerary	GdChab-11a	C	FUN	LRo	1800 - 1650	0	0	1	0	0	0	0	0	0	0	0	0	0	0	0	0	0	0	0	0	0	0	0	0	0	0	0	0
Grande Rivoire (La)	GdRiv-1	C	CAVE	Neo	7300 - 6500	0	0	0	0	0	0	1	0	0	0	0	0	0	0	0	0	0	0	0	0	0	1	0	9	0	0	0	0
Grande Rivoire (La)	GdRiv-2a	C	CAVE	Neo	6500 - 6000	0	0	17	0	0	0	0	0	0	0	0	0	0	0	0	0	0	0	0	0	0	0	0	32	0	0	0	1
Grande Rivoire (La)	GdRiv-2b	C	CAVE	Neo	6000 - 5500	0	0	7	0	0	0	0	0	0	0	0	0	0	0	0	1	0	0	0	0	7	0	0	15	0	0	0	4
Grande Rivoire (La)	GdRiv-9	C	CAVE	IA2	2100 - 2000	0	0	19	0	0	0	0	0	0	0	0	0	0	0	0	3	0	0	0	0	1	0	0	12	0	0	0	9
Grande Terre	GdTer-7	C	RURAL	IA1	2500 - 2400	0	0	2	0	0	0	0	0	0	0	0	0	0	0	0	0	0	0	0	0	0	0	0	0	0	0	0	7
Grange Rouge	QuGRo-6	C	RURAL	BA	3250 - 2900	0	0	0	0	0	0	0	0	0	0	0	0	0	0	0	0	0	0	0	0	0	0	0	0	0	0	0	192
Grange Rouge	QuGRo-7	C	RURAL	IA1	2700 - 2525	0	0	0	0	0	0	0	0	0	0	0	0	0	0	0	0	0	0	0	0	0	0	1	0	0	0	0	124
Gravette (La)	LaGrav-6	C	RURAL	BA	2950 - 2950	0	0	0	0	0	0	0	0	0	0	0	0	0	0	0	0	0	0	0	0	0	0	0	0	0	0	0	3
Gravette (La)	LaGrav-7	C	RURAL	IA1	2525 - 2500	0	0	0	0	0	0	0	0	0	0	0	0	0	0	0	0	0	0	0	0	0	0	0	0	0	0	0	10
Grésine Est	GRSE-6	C	RURAL	BA	2905 - 2850	0	0	0	0	0	0	0	0	0	0	0	0	0	0	0	25	0	0	0	0	0	0	0	0	0	0	0	7083
Grésine Est	GRSE-6	U	RURAL	BA	2905 - 2850	0	0	32	15	0	0	1	2	0	0	108	0	0	0	44	107	0	19	136	1087	506	0	0	0	0	0	0	9354
Grésine Ouest	GRSW-6	C	RURAL	BA	2905 - 2850	0	0	0	0	0	0	0	0	0	0	0	0	0	0	0	26	0	0	0	0	0	0	0	0	0	0	0	5247
Grésine Ouest	GRSW-6	U	RURAL	BA	2905 - 2850	0	0	33	18	0	0	0	3	0	0	19	0	0	0	12	53	0	26	77	453	90	0	1	0	0	0	0	8959
Guérine	Gueri-10	C	FUN	ERo	1925 - 1875	0	0	0	0	0	0	0	0	0	0	0	0	0	0	2	0	0	0	0	0	0	0	0	0	0	0	0	0
Guillemot-Labouygue	MoGLa-9	C	VILLAGE	IA2	2125 - 2075	0	0	0	0	0	0	0	0	0	0	0	0	0	0	0	0	0	0	0	0	0	0	0	0	0	0	0	0
Guillemot-Labouygue	MoGLa-10	C	VILLAGE	ERo	2000 - 1900	0	0	0	0	0	0	0	0	0	0	0	0	0	0	0	0	0	0	0	0	0	0	0	0	0	0	0	2
Hasard (Grotte du)	GtHas-6	C	CAVE	BA	3200 - 3050	0	0	0	0	0	0	0	0	0	0	0	0	0	0	0	0	0	0	0	0	0	0	0	0	0	0	0	335
Horand 2	LyHor2-10	C	FUN	ERo	2020 - 1750	0	0	0	0	0	0	0	0	0	0	0	0	0	0	0	0	0	0	0	0	0	0	0	0	0	0	0	0

Site	Cod Sit-Phas	Pres	type	Period	DATE BP	Quantif	MNI	Citrul	Cumel	Cupre	Cusat	Cusp	Jugla	Lage	Mespi	Moru	Prcer	Prdul	Prpers	Punic	Zizip	Cordi	Phoen	Celti	Coryl	Ficu	Fraga	Maloid	Malu	Olea	Pinpi	Pravce	Pravi	Prdom
île Saint Martin	IlStMa-10	C	RURAL	ERo	2000 - 1800	NMI	263	0	0	0	0	0	0	0	0	0	0	0	0	0	0	0	0	0	0	27	0	0	0	2	0	0	0	0
île Saint Martin	IlStMa-11	C	RURAL	LRo	1700 - 1500	NMI	4	0	0	0	0	0	0	0	0	0	0	0	0	0	0	0	0	0	0	0	0	0	0	0	0	0	0	0
Ilet	AnIlet-10	U	URBAN	ERo	1900 - 1700	NMI	1148	0	0	0	18	0	200	0	10	0	0	0	2	0	0	0	0	0	12	5	84	0	60	0	0	0	364	62
Jardin d'hiver	JdHiv-8	C	VILLAGE	IA1/2	2500 - 2400	NMI	1	0	0	0	0	0	0	0	0	0	0	0	0	0	0	0	0	0	0	0	0	0	0	0	0	0	0	0
Jardin d'hiver	JdHiv-9	C	VILLAGE	IA2	2425 - 2400	NMI	3	0	0	0	0	0	0	0	0	0	0	0	0	0	0	0	0	0	0	0	0	0	0	0	0	0	0	0
Jardins de Vert Parc	JVV-2	C	RURAL	Neo	6000 - 5500	NMI	174	0	0	0	0	0	0	0	0	0	0	0	0	0	0	0	0	0	0	0	0	0	0	0	0	0	0	0
Jas del Biau	JDBia-3	C	CAVE	Neo	5400 - 4400	NMI	1782	0	0	0	0	0	0	0	0	0	0	0	0	0	0	0	0	0	1	0	0	0	0	0	0	0	0	0
Jas del Biau 2	JdBia2-3	C	RURAL	Neo	5400 - 4400	NMI	624	0	0	0	0	0	0	0	0	0	0	0	0	0	0	0	0	0	26	0	0	0	0	0	0	0	0	0
Jean Jaurès (Avenue)	NAvJJ-10	C	FUN	ERo	2000 - 1800	NMI	248	0	0	0	0	0	0	0	0	0	0	0	0	0	0	18	2	0	0	10	0	0	0	10	74	0	0	2
Joncasses (Les)	Ljoncs-7	C	FUN	IA1	2700 - 2600	NMI	114	0	0	0	0	0	0	0	0	0	0	0	0	0	0	0	0	0	0	0	0	0	0	0	0	0	0	0
Jouffe	Jouff-8	C	VILLAGE	IA1/2	2525 - 2450	NMI	17682	0	0	0	0	0	0	0	0	0	0	0	0	0	0	0	0	0	0	0	0	0	0	0	0	0	0	0
Jules Verne 10	JV10-10	C	URBAN	ERo	1925 - 1800	NMI	23	0	0	0	0	0	0	0	0	0	0	0	0	0	0	0	0	0	0	9	0	0	0	0	2	0	0	0
Jules Verne 10	JV10-10	U	URBAN	ERo	1925 - 1800	NMI	37122	0	120	0	121	0	130	0	0	172	0	2	2	6	0	1	0	0	8	32804	0	0	18	86	6	0	97	4
Jules Verne 10	JV10-11	C	URBAN	LRo	1800 - 1600	NMI	4	0	0	0	0	0	0	0	0	0	0	0	0	0	0	0	0	0	0	1	0	0	0	0	0	0	0	0
Jules Verne 10	JV10-11	U	URBAN	LRo	1800 - 1600	NMI	9910	0	22	0	11	0	45	0	0	24	0	0	7	0	0	1	0	0	9	8281	0	0	11	54	42	0	7	1
Jules Verne 11	JV11-9	C	URBAN	IA2	2300 - 2100	NMI	12	0	0	0	0	0	0	0	0	0	0	0	0	0	0	0	0	0	1	0	0	0	0	5	0	0	0	0
Jules Verne 11	JV11-9	U	URBAN	IA2	2300 - 2100	NMI	26931	0	40	0	0	0	1	0	0	0	0	5	0	14	0	0	0	0	24	22624	0	0	16	180	1	0	0	0
Jules Verne 14	JV14-7	C	URBAN	IA1	2600 - 2500	NMI	7	0	0	0	0	0	0	0	0	0	0	0	0	0	0	0	0	0	1	0	0	0	0	2	0	0	0	0
Jules Verne 14	JV14-7	U	URBAN	IA1	2600 - 2500	NMI	6067	0	0	0	0	65	0	0	0	0	0	0	0	119	0	0	0	0	41	1706	0	0	64	30	0	0	0	0
Jules Verne 14	JV14-8	C	URBAN	IA1/2	2525 - 2400	NMI	19	0	0	0	0	0	0	0	0	0	0	0	0	0	0	0	0	0	2	0	0	0	0	0	0	0	0	0
Jules Verne 14	JV14-8	U	URBAN	IA1/2	2525 - 2400	NMI	28181	3	81	0	0	0	0	0	0	0	0	3	0	226	0	0	0	0	86	10794	0	0	42	114	9	0	0	0
Jurières Basses	JuBs-11	C	RURAL	LRo	1600 - 1400	NMI	15	0	0	0	0	0	0	0	0	0	0	0	0	0	0	0	0	0	0	0	0	0	0	0	0	0	0	0
Juvignac	MtpJuv-3	C	RURAL	Neo	5500 - 4200	P/A	2	0	0	0	0	0	0	0	0	0	0	0	0	0	0	0	0	0	0	0	0	0	0	0	0	0	0	0
La Garanne	LaGar-10	C	RURAL	ERo	1900 - 1750	NMI	452	0	0	0	0	0	0	0	0	0	0	0	0	0	0	0	0	0	0	0	0	0	1	432	0	0	0	0
La Garanne	LaGar-11	C	RURAL	LRo	1800 - 1650	NMI	26	0	0	0	0	0	0	0	0	0	0	0	0	0	0	0	0	0	0	0	0	0	0	8	0	0	0	0
La Plaine	PuyPla-7	C	RURAL	IA1	2600 - 2500	NMI	42	0	0	0	0	0	0	0	0	0	0	0	0	0	0	0	0	0	1	0	0	0	0	0	0	0	0	0
La Plaine	PuyPla-9	C	RURAL	IA2	2150 - 2100	NMI	1157	0	0	0	0	0	0	0	0	0	0	0	0	0	0	0	0	0	2	0	0	0	0	0	0	0	0	0
La Plaine	PuyPla-9	U	RURAL	IA2	2150 - 2100	NMI	121	0	0	0	0	0	0	0	0	0	0	0	0	0	0	0	0	0	8	0	0	0	0	0	0	0	0	0
La Royale	LaRoy-4	C	RURAL	BA	4200 - 3700	NMI	2862	*0*	*0*	*0*	*0*	*0*	*0*	*0*	*0*	*0*	*0*	*0*	*0*	*0*	0	*0*	*0*	*0*	*0*	*0*	*0*	*0*	*0*	*0*	*0*	*0*	*0*	*0*
Lagarel	SALag-3	C	RURAL	Neo	5000 - 4600	NMI	632	0	0	0	0	0	0	0	0	0	0	0	0	0	0	0	0	0	0	0	0	0	0	0	0	0	0	0
L'Alba	CasAlb-9	C	RURAL	IA2	2175 - 2125	NMI	28	0	0	0	0	0	0	0	0	0	0	0	0	0	0	0	0	0	0	0	0	0	0	0	0	0	0	0
Laouret (Le)	LeLao-6	C	RURAL	BA	3050 - 2900	NMI	77	0	0	0	0	0	0	0	0	0	0	0	0	0	0	0	0	0	0	0	0	0	0	0	0	0	0	0
Lattara	LtLat-8	C	URBAN	IA1/2	2500 - 2400	NMI	125498	0	0	0	0	0	0	0	0	0	0	2	0	0	0	0	0	0	32	312	0	0	0	24	0	3	0	1
Lattara	LtLat-8	U	URBAN	IA1/2	2500 - 2400	NMI	2419	0	0	0	0	12	0	1	0	0	0	0	0	0	0	0	0	0	10	1122	0	0	0	16	0	1	0	0
Lattara	LtLat-9a	C	URBAN	IA2	2400 - 2300	NMI	13287	0	0	0	0	0	1	0	0	0	0	0	0	0	0	0	0	0	4	9	0	0	0	5	0	2	0	0
Lattara	LtLat-9b	C	URBAN	IA2	2300 - 2200	NMI	1756	0	0	0	0	0	0	0	0	0	0	0	0	0	0	0	0	0	1	0	0	0	0	2	0	0	0	0
Lattara	LtLat-9c	C	URBAN	IA2	2200 - 2100	NMI	1111	0	0	0	0	0	0	0	0	0	0	0	0	0	0	0	0	0	0	0	0	0	0	0	0	0	0	0
Lattara	LtLat-9d	C	URBAN	IA2	2100 - 2000	NMI	23705	0	0	0	0	0	0	0	0	0	0	0	0	0	0	0	0	0	0	2	0	0	0	0	0	0	0	0
Lattara	LtLat-9d	U	URBAN	IA2	2050 - 2040	NMI	35498	0	0	0	0	0	0	0	0	0	0	0	0	0	0	0	0	0	0	0	0	0	0	0	0	0	0	0
Lattara	LtLat-10a	U	URBAN	ERo	2000 - 1900	NMI	403	0	0	0	0	0	100	0	0	0	6	0	7	0	0	0	0	114	6	0	0	0	0	10	41	0	0	6
Lattara	LtLat-10a	C	URBAN	ERo	2000 - 1900	NMI	1364	0	0	0	0	0	0	0	0	0	29	0	27	0	0	0	0	0	1	1	0	0	0	34	10	0	0	45
Lattara	LtLat-10b	C	URBAN	ERo	1900 - 1875	NMI	63	0	0	0	0	0	0	0	0	0	0	0	0	0	0	0	0	0	0	0	0	0	0	0	0	0	0	0
Lattara - Cultuel	LtLatC-10	C	FUN	ERo	1975 - 1940	NMI	1431	0	0	0	0	0	0	0	0	0	0	0	0	0	0	0	7	0	0	1200	0	0	0	0	176	0	0	0
Lattara - Port	LtLatP-10a	U	URBAN	ERo	2050 - 2000	NMI	184	0	0	0	0	0	2	0	0	0	0	0	0	0	0	0	0	0	3	64	0	0	0	0	0	0	0	0
Lattara - Port	LtLatP-10b	U	URBAN	ERo	2000 - 2100	NMI	7743	0	0	1	0	0	98	0	0	15	0	0	1	0	0	0	0	0	19	1073	0	0	0	39	0	0	0	1
Lattara - Port	LtLatP-10b	C	URBAN	ERo	2000 - 1900	NMI	3	0	0	0	0	0	0	0	0	0	0	0	0	0	0	0	0	0	0	0	0	0	0	0	0	0	0	0
Lattara - Port	LtLatP-10c	C	URBAN	ERo	1850 - 1800	NMI	17	0	0	0	0	0	1	0	0	0	0	0	0	0	0	0	0	0	0	0	0	0	0	0	0	0	0	0
Lattara - Port	LtLatP-10c	U	URBAN	ERo	1850 - 1800	NMI	11917	0	0	0	0	43	1104	0	0	2	0	0	4	0	4	0	0	0	17	5111	0	0	0	325	43	0	8	7
Lautagne	Lautag-10	C	VILLAGE	ERo	2125 - 2025	NMI	3416	0	0	0	0	0	0	0	0	0	0	0	0	0	0	0	0	0	0	0	0	0	0	2	0	0	0	0
Le Chenet des Pierres	BozCP-2a	C	RURAL	Neo	6500 - 6000	NMI	981	0	0	0	0	0	0	0	0	0	0	0	0	0	0	0	0	0	144	0	0	0	5	0	0	0	0	0
Le Chenet des Pierres	BozCP-2b	C	RURAL	Neo	6000 - 5500	NMI	2192	0	0	0	0	0	0	0	0	0	0	0	0	0	0	0	0	0	278	0	0	0	33	0	0	0	0	0
Les Combariès	Comba-9	C	VILLAGE	IA2	2125 - 2075	NMI	51	0	0	0	0	0	0	0	0	0	0	0	0	0	0	0	0	0	18	0	0	0	0	0	0	0	0	0
Les Croisières	Croisi-1	C	RURAL	Neo	7000 - 6500	NMI	1	0	0	0	0	0	0	0	0	0	0	0	0	0	0	0	0	0	0	0	0	0	0	0	0	0	0	0
Les Croisières	Croisi-2	C	RURAL	Neo	6500 - 5500	NMI	6	0	0	0	0	0	0	0	0	0	0	0	0	0	0	0	0	0	0	0	0	0	0	0	0	0	0	0
Les Croisières	Croisi-7	C	RURAL	IA1	2850 - 2600	NMI	266	0	0	0	0	0	0	0	0	0	0	0	0	0	0	0	0	0	2	0	0	0	0	0	0	0	0	0
Lesse (La)	SaLSS-10a	C	RURAL	ERo	2025 - 1975	NMI	70	0	0	0	0	0	1	0	0	0	0	0	0	0	0	0	0	0	0	0	0	0	0	0	0	0	0	0
Lesse (La)	SaLSS-10a	U	RURAL	ERo	2025 - 1975	NMI	1024	0	0	48	0	0	14	0	0	13	0	0	0	0	0	0	1	0	1	283	0	0	0	55	5	6	0	4
Lesse (La)	SaLSS-10b	U	RURAL	ERo	1950 - 1875	NMI	3136	0	0	0	0	0	4	0	0	2	0	0	6	0	0	0	0	0	2	51	0	0	0	16	282	4	0	7
Lesse (La)	SaLSS-10c	U	RURAL	ERo	1850 - 1825	NMI	537	0	0	0	0	0	1	0	0	2	0	0	0	0	0	0	0	0	0	31	0	0	0	3	2	0	0	0
Lesse (La)	SaLSS-10d	C	RURAL	ERo	1825 - 1725	NMI	2	0	0	0	0	0	0	0	0	0	0	0	0	0	0	0	0	0	0	0	0	0	0	0	0	0	0	0
Lesse (La)	SaLSS-10d	U	RURAL	ERo	1825 - 1725	NMI	1825	0	10	0	0	0	1	0	0	24	0	0	0	0	0	0	0	0	0	83	0	0	0	1	0	0	0	0
Liquière (La)	Lliq-7	C	VILLAGE	IA1	2600 - 2575	NMI	602	0	0	0	0	0	0	0	0	0	0	0	0	0	0	0	0	0	0	0	0	0	0	0	0	0	0	0
Lladre (Lo)	LLLa-6	C	RURAL	BA	3050 - 2800	NMI	12000	0	0	0	0	0	0	0	0	0	0	0	0	0	0	0	0	0	3	0	0	0	0	0	0	0	0	0

Site	Cod Sit-Phas	Pres	type	Period	DATE BP	Prsp	Pyru	Viti	Vitipd	Arbu	Corma	Corsa	Crata	Junsp	Myrt	Physa	Pincem	Pinsp	Pista	Prspi	Querc	Rhus	Rosa	Rubca	Rubfr	Rubid	Rubsp	Sambeb	Sambni	Sambsp	Sorb	Vibu	An crops
île Saint Martin	IlStMa-10	C	RURAL	ERo	2000 - 1800	1	0	51	2	0	0	0	0	0	0	0	0	1	0	0	0	0	0	0	0	0	0	0	0	0	0	0	179
île Saint Martin	IlStMa-11	C	RURAL	LRo	1700 - 1500	0	0	4	0	0	0	0	0	0	0	0	0	0	0	0	0	0	0	0	0	0	0	0	0	0	0	0	0
Ilet	AnIlet-10	U	URBAN	ERo	1900 - 1700	0	0	320	0	0	0	0	0	0	0	0	0	0	0	4	0	0	0	0	0	0	0	0	0	0	0	0	7
Jardin d'hiver	JdHiv-8	C	VILLAGE	IA1/2	2500 - 2400	0	0	1	0	0	0	0	0	0	0	0	0	0	0	0	0	0	0	0	0	0	0	0	0	0	0	0	0
Jardin d'hiver	JdHiv-9	C	VILLAGE	IA2	2425 - 2400	0	0	0	0	0	0	0	0	0	0	0	0	0	0	0	0	0	0	0	0	0	0	0	0	0	0	0	3
Jardins de Vert Parc	JVV-2	C	RURAL	Neo	6000 - 5500	0	0	0	0	0	0	0	0	0	0	0	0	0	0	0	2	0	0	0	0	0	1	29	3	0	0	0	139
Jas del Biau	JDBia-3	C	CAVE	Neo	5400 - 4400	1	0	0	0	0	0	0	0	0	0	0	0	0	0	0	2	0	0	0	0	0	0	0	0	0	0	0	1778
Jas del Biau 2	JdBia2-3	C	RURAL	Neo	5400 - 4400	2	0	1	1	0	0	0	0	0	0	0	0	0	0	0	559	0	0	0	0	0	0	0	0	0	0	0	35
Jean Jaurès (Avenue)	NAvJJ-10	C	FUN	ERo	2000 - 1800	0	0	4	0	0	0	0	0	0	0	0	0	0	0	0	0	0	0	0	0	0	0	0	0	0	0	0	128
Joncasses (Les)	Ljoncs-7	C	FUN	IA1	2700 - 2600	7	0	0	0	0	0	0	0	0	0	0	0	0	0	0	0	0	0	0	0	0	0	0	0	0	0	0	107
Jouffe	Jouff-8	C	VILLAGE	IA1/2	2525 - 2450	0	0	2	0	0	0	0	0	0	0	0	0	0	0	0	109	0	0	0	0	0	0	0	0	0	0	0	17571
Jules Verne 10	JV10-10	C	URBAN	ERo	1925 - 1800	0	0	2	0	0	0	0	0	0	0	0	0	0	0	0	0	0	0	0	0	0	0	0	0	0	0	0	10
Jules Verne 10	JV10-10	U	URBAN	ERo	1925 - 1800	0	0	2991	129	0	0	0	0	1	2	18	0	0	0	0	2	0	0	4	196	0	0	15	15	0	0	0	184
Jules Verne 10	JV10-11	C	URBAN	LRo	1800 - 1600	0	0	0	0	0	0	0	0	0	0	0	0	0	0	0	0	0	0	0	0	0	0	0	0	0	0	0	3
Jules Verne 10	JV10-11	U	URBAN	LRo	1800 - 1600	0	0	830	390	0	0	0	0	4	6	7	0	0	2	0	6	0	0	0	91	0	0	8	0	0	0	0	49
Jules Verne 11	JV11-9	C	URBAN	IA2	2300 - 2100	0	0	2	0	0	0	0	0	0	0	0	0	0	0	0	0	0	0	0	0	0	0	0	0	0	0	0	4
Jules Verne 11	JV11-9	U	URBAN	IA2	2300 - 2100	0	0	2927	270	0	0	0	0	0	10	0	0	0	3	0	6	0	0	0	501	0	0	9	0	0	0	0	300
Jules Verne 14	JV14-7	C	URBAN	IA1	2600 - 2500	0	0	2	0	0	0	0	0	0	0	0	0	0	0	0	0	0	0	0	0	0	0	0	0	0	0	0	2
Jules Verne 14	JV14-7	U	URBAN	IA1	2600 - 2500	0	0	2877	831	0	0	0	0	6	6	4	0	0	12	0	8	0	0	0	169	0	0	17	0	0	0	0	112
Jules Verne 14	JV14-8	C	URBAN	IA1/2	2525 - 2400	0	0	3	0	0	0	0	0	0	0	0	0	0	0	0	0	0	0	0	0	0	0	0	0	0	0	0	14
Jules Verne 14	JV14-8	U	URBAN	IA1/2	2525 - 2400	0	0	12614	2913	0	0	0	0	6	82	2	0	0	16	3	19	0	4	3	554	0	0	0	1	0	0	0	605
Jurières Basses	JuBs-11	C	RURAL	LRo	1600 - 1400	0	0	9	0	0	0	0	0	0	0	0	0	0	0	0	0	0	0	0	0	0	0	0	0	0	0	0	6
Juvignac	MtpJuv-3	C	RURAL	Neo	5500 - 4200	0	0	0	0	0	0	0	0	0	0	0	0	0	0	0	0	0	0	0	0	0	0	0	0	0	0	0	2
La Garanne	LaGar-10	C	RURAL	ERo	1900 - 1750	0	0	8	1	0	0	0	0	0	0	0	0	0	0	0	0	0	0	0	0	0	0	0	0	0	0	0	10
La Garanne	LaGar-11	C	RURAL	LRo	1800 - 1650	0	0	3	0	0	0	0	0	0	0	0	0	0	0	0	0	0	0	0	0	0	0	0	0	0	0	0	15
La Plaine	PuyPla-7	C	RURAL	IA1	2600 - 2500	0	0	1	0	0	0	0	0	0	0	0	0	0	0	0	0	0	0	0	0	0	0	0	0	0	0	0	40
La Plaine	PuyPla-9	C	RURAL	IA2	2150 - 2100	0	0	1	0	0	0	0	0	0	0	0	0	0	0	0	0	0	0	0	0	0	0	0	1	0	0	0	1153
La Plaine	PuyPla-9	U	RURAL	IA2	2150 - 2100	0	0	3	0	0	0	3	0	0	0	0	0	0	0	0	74	0	0	0	17	0	0	10	6	0	0	0	0
La Royale	LaRoy-4	C	RURAL	BA	4200 - 3700	*0*	*0*	*0*	*0*	*0*	0	0	*0*	*0*	*0*	*0*	*0*	0	*0*	*0*	2860	*0*	*0*	*0*	*0*	*0*	*0*	*0*	*0*	*0*	*0*	*0*	2
Lagarel	SALag-3	C	RURAL	Neo	5000 - 4600	0	0	0	0	0	0	0	0	0	0	0	0	0	0	0	0	0	0	0	0	0	0	0	0	0	0	0	632
L'Alba	CasAlb-9	C	RURAL	IA2	2175 - 2125	0	0	1	0	0	0	0	0	0	0	0	0	0	0	0	1	0	0	0	0	0	0	0	2	0	0	0	24
Laouret (Le)	LeLao-6	C	RURAL	BA	3050 - 2900	0	0	0	0	0	0	0	0	0	0	0	0	0	0	0	15	0	0	0	0	0	0	0	0	0	0	0	62
Lattara	LtLat-8	C	URBAN	IA1/2	2500 - 2400	0	2	12136	204	1	1	0	0	0	0	0	0	0	2	5	6	0	1	2	12	0	0	23	1	0	0	0	112728
Lattara	LtLat-8	U	URBAN	IA1/2	2500 - 2400	0	0	825	2	0	2	0	0	0	0	0	0	0	0	0	0	0	0	0	205	1	0	179	39	0	0	0	4
Lattara	LtLat-9a	C	URBAN	IA2	2400 - 2300	0	0	757	22	0	0	0	1	0	0	0	0	0	0	1	5	0	0	0	0	4	0	30	1	0	2	0	12443
Lattara	LtLat-9b	C	URBAN	IA2	2300 - 2200	0	0	1013	6	0	0	0	0	0	0	0	0	0	0	1	1	0	0	0	0	0	0	0	0	0	0	0	732
Lattara	LtLat-9c	C	URBAN	IA2	2200 - 2100	0	0	1027	3	0	0	0	0	0	0	0	0	0	0	0	0	0	0	0	1	0	0	0	0	0	0	0	80
Lattara	LtLat-9d	C	URBAN	IA2	2100 - 2000	0	0	23565	4	0	0	0	0	0	0	0	0	0	0	0	0	0	0	0	0	0	0	1	0	0	0	0	133
Lattara	LtLat-9d	U	URBAN	IA2	2050 - 2040	0	0	35494	4	0	0	0	0	0	0	0	0	0	0	0	0	0	0	0	0	0	0	0	0	0	0	0	0
Lattara	LtLat-10a	U	URBAN	ERo	2000 - 1900	0	0	110	1	0	0	0	0	0	0	0	0	0	0	0	1	0	0	0	0	0	1	0	0	0	0	0	0
Lattara	LtLat-10a	C	URBAN	ERo	2000 - 1900	0	0	95	0	0	0	0	0	0	0	0	0	0	0	26	6	0	9	0	0	0	0	0	0	0	0	0	1081
Lattara	LtLat-10b	C	URBAN	ERo	1900 - 1875	0	0	2	0	0	0	0	0	0	0	0	0	0	0	0	0	0	0	0	0	0	0	0	0	0	0	0	61
Lattara - Cultuel	LtLatC-10	C	FUN	ERo	1975 - 1940	0	0	2	0	0	0	0	0	0	0	0	0	0	0	0	0	0	0	0	0	0	0	0	0	0	0	0	46
Lattara - Port	LtLatP-10a	U	URBAN	ERo	2050 - 2000	0	0	102	6	0	0	0	0	0	0	0	0	0	0	0	0	0	0	0	0	0	1	0	0	5	0	0	1
Lattara - Port	LtLatP-10b	U	URBAN	ERo	2000 - 2100	0	0	6303	60	0	0	0	0	0	0	0	0	1	0	1	17	0	0	0	26	0	0	0	0	0	0	0	88
Lattara - Port	LtLatP-10b	C	URBAN	ERo	2000 - 1900	0	0	0	0	0	0	0	0	0	0	0	0	0	0	0	0	0	0	0	0	0	0	0	0	0	0	0	3
Lattara - Port	LtLatP-10c	C	URBAN	ERo	1850 - 1800	0	0	6	10	0	0	0	0	0	0	0	0	0	0	0	0	0	0	0	0	0	0	0	0	0	0	0	0
Lattara - Port	LtLatP-10c	U	URBAN	ERo	1850 - 1800	0	0	3914	867	0	0	0	0	0	0	1	0	0	0	4	4	0	0	0	106	0	0	224	12	0	0	0	117
Lautagne	Lautag-10	C	VILLAGE	ERo	2125 - 2025	0	0	1	0	0	0	1	0	0	0	0	0	0	0	0	0	0	0	0	0	0	0	0	0	0	0	0	3412
Le Chenet des Pierres	BozCP-2a	C	RURAL	Neo	6500 - 6000	0	0	0	0	0	0	0	0	0	0	0	78	0	0	2	1	0	0	0	0	63	0	0	194	0	0	0	494
Le Chenet des Pierres	BozCP-2b	C	RURAL	Neo	6000 - 5500	0	0	0	0	0	0	0	0	0	0	0	31	0	0	0	17	0	0	0	0	9	0	0	92	0	0	0	1732
Les Combariès	Comba-9	C	VILLAGE	IA2	2125 - 2075	0	0	0	0	0	0	0	0	0	0	0	0	0	0	0	0	0	0	0	0	0	1	0	2	0	0	0	30
Les Croisières	Croisi-1	C	RURAL	Neo	7000 - 6500	0	0	0	0	0	0	0	0	0	0	0	0	0	0	0	1	0	0	0	0	0	0	0	0	0	0	0	0
Les Croisières	Croisi-2	C	RURAL	Neo	6500 - 5500	0	0	0	0	0	0	0	0	0	0	0	0	0	0	0	2	0	0	0	0	0	0	0	0	0	0	0	4
Les Croisières	Croisi-7	C	RURAL	IA1	2850 - 2600	0	0	0	0	0	0	0	0	0	0	0	0	0	0	0	64	0	0	0	0	0	0	0	0	0	0	0	200
Lesse (La)	SaLSS-10a	C	RURAL	ERo	2025 - 1975	0	0	65	0	0	0	0	0	0	0	0	0	0	0	0	0	0	0	0	0	0	0	0	0	0	0	0	4
Lesse (La)	SaLSS-10a	U	RURAL	ERo	2025 - 1975	0	0	549	33	0	0	0	0	0	1	0	0	0	0	0	0	0	0	0	2	0	0	0	0	0	1	0	8
Lesse (La)	SaLSS-10b	U	RURAL	ERo	1950 - 1875	0	0	2677	75	0	0	0	0	0	0	0	0	0	0	0	2	0	0	0	3	0	0	2	0	0	0	0	3
Lesse (La)	SaLSS-10c	U	RURAL	ERo	1850 - 1825	0	0	236	194	0	0	0	0	0	0	0	0	0	0	0	0	0	0	0	29	0	0	0	7	0	0	0	32
Lesse (La)	SaLSS-10d	C	RURAL	ERo	1825 - 1725	0	0	0	0	0	0	0	0	0	0	0	0	0	0	0	0	0	0	0	0	0	0	0	0	0	0	0	2
Lesse (La)	SaLSS-10d	U	RURAL	ERo	1825 - 1725	0	0	121	1108	0	0	0	0	0	4	0	0	0	0	0	0	0	0	0	109	0	0	0	54	0	0	0	310
Liquière (La)	Lliq-7	C	VILLAGE	IA1	2600 - 2575	0	0	0	0	0	0	0	0	0	0	0	0	0	0	0	33	0	0	0	0	0	0	0	0	0	0	0	569
Lladre (Lo)	LLLa-6	C	RURAL	BA	3050 - 2800	0	0	0	0	0	0	0	0	0	0	0	0	0	0	0	0	0	1	0	0	0	0	0	0	0	0	0	11996

Site	Cod Sit-Phas	Pres	type	Period	DATE BP	Quantif	MNI	Citrul	Cumel	Cupre	Cusat	Cusp	Jugla	Lage	Mespi	Moru	Prcer	Prdul	Prpers	Punic	Zizip	Cordi	Phoen	Celti	Coryl	Ficu	Fraga	Maloid	Malu	Olea	Pinpi	Pravce	Pravi	Prdom
Logis de Berre (Le)	LoBer-2	C	RURAL	Neo	6000 - 5500	NMI	246	0	0	0	0	0	0	0	0	0	0	0	0	0	0	0	0	0	0	0	0	0	0	0	0	0	0	0
Lombard (Grotte)	GtLom-1	C	CAVE	Neo	7500 - 7000	NMI	1	0	0	0	0	0	0	0	0	0	0	0	0	0	0	0	0	0	0	0	0	0	0	0	0	0	0	0
Lycée Technique	MtpLT-7	C	RURAL	IA1	2700 - 2600	P/A	2	0	0	0	0	0	0	0	0	0	0	0	0	0	0	0	0	0	0	0	0	0	0	0	0	0	0	0
Machal (Le)	DaMch-4	C	RURAL	BA	4050 - 3600	NMI	169	0	0	0	0	0	0	0	0	0	0	0	0	0	0	0	0	0	6	0	0	0	0	0	0	0	0	0
Madeleine I	PeMa1-10	C	RURAL	ERo	2150 - 2075	NMI	2	0	0	0	0	0	0	0	0	0	0	0	0	0	0	0	0	0	0	0	0	0	0	0	0	0	0	0
Magasins (Les), Buzerens	MaBuz-9a	U	RURAL	IA2	2500 - 2400	NMI	4	0	0	0	0	0	0	0	0	0	0	0	0	0	0	0	0	0	0	0	0	0	0	0	0	0	0	0
Magasins (Les), Buzerens	MaBuz-9b	U	RURAL	IA2	2125 - 2050	NMI	36	0	0	0	0	0	0	0	0	0	0	0	0	0	0	0	0	0	0	0	0	0	0	0	0	0	0	0
Magasins (Les), Buzerens	MaBuz-9b	C	RURAL	IA2	2120 - 2050	NMI	95	0	0	0	0	0	0	0	0	0	0	0	0	0	0	0	0	0	0	0	0	0	0	0	0	0	0	0
Maison Philippon	ElMP-10	C	RURAL	ERo	2000 - 1900	NMI	6	0	0	0	0	0	0	0	0	0	0	0	0	0	0	0	0	0	0	0	0	0	0	0	0	0	0	0
Maladrerie/Lot. du Grand Chêne	MalGdC-11	C	RURAL	LRo	1800 - 1525	NMI	184	0	0	0	0	0	129	0	0	0	0	0	0	0	0	0	0	0	0	0	0	0	0	0	2	0	0	0
Marduel	Mrdu-6	C	VILLAGE	BA	2900 - 2750	NMI	16	0	0	0	0	0	0	0	0	0	0	0	0	0	0	0	0	0	0	0	0	0	0	0	0	0	0	0
Marduel	Mrdu-7	C	VILLAGE	IA1	2550 - 2500	NMI	112	0	0	0	0	0	0	0	0	0	0	0	0	0	0	0	0	0	0	0	0	0	0	0	0	0	0	0
Marduel	Mrdu-8	C	VILLAGE	IA1/2	2500 - 2450	NMI	205	0	0	0	0	0	0	0	0	0	0	0	0	0	0	0	0	0	0	0	0	0	0	0	0	0	0	0
Marduel	Mrdu-9a	C	VILLAGE	IA2	2450 - 2400	NMI	8482	0	0	0	0	0	0	0	0	0	0	0	0	0	0	0	0	0	0	1	0	0	0	0	0	0	0	0
Marduel	Mrdu-9b	C	VILLAGE	IA2	2250 - 2150	NMI	299	0	0	0	0	0	0	0	0	0	0	0	0	0	0	0	0	0	0	0	0	0	0	0	0	0	0	0
Martigues (île)	IlMart-9a	C	VILLAGE	IA2	2400 - 2375	NMI	51525	0	0	0	0	0	0	0	0	0	0	0	0	0	0	0	0	0	0	1	0	0	0	0	0	0	0	0
Martigues (île)	IlMart-9b	U	VILLAGE	IA2	2200 - 2200	P/A	1	0	0	0	0	0	0	0	0	0	0	0	0	0	0	0	0	0	0	0	0	0	0	1	0	0	0	0
Mas (Le)	AnsMa-Ro	C	RURAL	Rom	2025 - 1500	NMI	8	0	0	0	0	0	0	0	0	0	0	0	0	0	0	0	0	0	0	0	0	0	0	0	1	0	0	0
Mas Coste	PeMC-10	C	RURAL	ERo	2120 - 1940	NMI	45	0	0	0	0	0	1	0	0	0	0	0	0	0	0	0	0	0	0	1	0	0	0	5	0	0	0	0
Mas de Causse	LtMdC-7	C	RURAL	IA1	2600 - 2500	NMI	18	0	0	0	0	0	0	0	0	0	0	0	0	0	0	0	0	0	0	2	0	0	0	0	0	0	0	0
Mas de Causse	LtMdC-8	C	RURAL	IA1/2	2525 - 2400	NMI	97	0	0	0	0	0	0	0	0	0	0	0	0	0	0	0	0	0	0	4	0	0	0	0	0	0	0	0
Mas de Causse	LtMdC-9a	C	RURAL	IA2	2500 - 2300	NMI	46	0	0	0	0	0	0	0	0	0	0	0	0	0	0	0	0	0	0	2	0	0	0	0	0	0	0	0
Mas de Causse	LtMdC-9b	C	RURAL	IA2	2100 - 2000	NMI	23	0	0	0	0	0	0	0	0	0	0	0	0	0	0	0	0	0	0	0	0	0	0	0	0	0	0	0
Mas de Causse	LtMdC-10	C	RURAL	ERo	2000 - 1900	NMI	5	0	0	0	0	0	0	0	0	0	0	0	0	0	0	0	0	0	0	0	0	0	0	0	0	0	0	0
Mas de Causse	LtMdC-10	U	RURAL	ERo	2000 - 1900	NMI	658	0	2	0	0	0	3	0	0	0	0	0	0	0	0	0	0	6	0	221	0	0	0	0	1	0	0	0
Mas de Causse	LtMdC-11	C	RURAL	LRo	1800 - 1600	NMI	33	0	0	0	0	0	0	0	0	0	0	1	0	0	0	0	0	0	0	0	0	0	0	0	1	0	0	0
Mas de Causse	LtMdC-11	U	RURAL	LRo	1800 - 1600	NMI	94	0	0	0	0	0	0	0	0	0	0	0	0	0	0	0	0	0	0	2	0	0	0	0	1	0	0	0
Mas de Causse - Cultual	LtMdCC-9	C	FUN	IA2	2500 - 2300	NMI	13	0	0	0	0	0	0	0	0	0	0	0	0	0	0	0	0	0	0	0	0	0	0	0	0	0	0	0
Mas de Vignoles 10	MdV10-1	C	RURAL	Neo	7000 - 6500	NMI	1	0	0	0	0	0	0	0	0	0	0	0	0	0	0	0	0	0	0	0	0	0	0	0	0	0	0	0
Mas de Vignoles 10	MdV10-7	C	RURAL	IA1	2750 - 2625	NMI	42	0	0	0	0	0	0	0	0	0	0	0	0	0	0	0	0	0	0	0	0	0	0	0	0	0	0	0
Mas de Vignoles 9	MdV9-2	C	RURAL	Neo	6500 - 5500	NMI	1	0	0	0	0	0	0	0	0	0	0	0	0	0	0	0	0	0	0	0	0	0	0	0	0	0	0	0
Mas de Vignoles 9	MdV9-2	U	RURAL	Neo	6500 - 5500	NMI	8	0	0	0	0	0	0	0	0	0	0	0	0	0	0	0	0	0	0	3	0	0	0	0	0	0	0	0
Mas de Vignoles 9	MdV9-5	U	RURAL	BA	3650 - 1350	NMI	53	0	0	0	0	0	0	0	0	0	0	0	0	0	0	0	0	0	0	0	0	0	0	1	0	0	0	0
Mas de Vignoles 9	MdV9-7	C	RURAL	IA1	2750 - 2450	NMI	14	0	0	0	0	0	0	0	0	0	0	0	0	0	0	0	0	0	0	0	0	0	0	0	0	0	0	0
Mas de Vignoles 9	MdV9-7	U	RURAL	IA1	2750 - 2450	NMI	331	0	0	0	0	0	0	0	0	0	0	0	0	0	0	0	0	0	0	214	0	0	0	0	0	0	0	0
Mas de Vignoles 9	MdV9-5	C	RURAL	BA	2600 - 1350	NMI	0	0	0	0	0	0	0	0	0	0	0	0	0	0	0	0	0	0	0	0	0	0	0	0	0	0	0	0
Mas de Vignoles 9	MdV9-10	C	RURAL	ERo	2000 - 1900	NMI	4	0	0	0	0	0	0	0	0	0	0	0	0	0	0	0	0	0	0	4	0	0	0	0	0	0	0	0
Mas de Vignoles 9	MdV9-10	U	RURAL	ERo	2000 - 1900	NMI	778	0	0	0	0	0	17	0	0	0	0	0	0	0	0	0	0	0	0	535	0	0	0	0	0	0	0	0
Mas Delfau	PeMDe-10	C	RURAL	ERo	2100 - 1900	NMI	505	0	0	0	0	0	0	0	0	0	0	0	0	0	0	0	0	0	0	0	0	0	0	3	0	0	0	1
Mas des Abeilles	NMdAb-3	C	RURAL	Neo	5500 - 4200	NMI	18490	0	0	0	0	0	0	0	0	0	0	0	0	0	0	0	0	0	0	0	0	0	0	0	0	0	0	0
Mas des Abeilles	NMdAb-7	C	RURAL	IA1	2700 - 2600	NMI	5	0	0	0	0	0	0	0	0	0	0	0	0	0	0	0	0	0	0	0	0	0	0	0	0	0	0	0
Mas Neuf	NMNeu-1	C	RURAL	Neo	7000 - 6500	NMI	47450	0	0	0	0	0	0	0	0	0	0	0	0	0	0	0	0	0	0	0	0	0	0	0	0	0	0	0
Mas Roig	MRoig-10	C	RURAL	ERo	1990 - 1970	NMI	18	0	0	0	0	0	0	0	0	0	0	0	0	0	0	0	0	0	0	0	0	0	0	17	0	0	0	0
Mas Sauvy	VRaMS-10	C	RURAL	ERo	1940 - 1880	NMI	46	0	0	0	0	0	1	0	0	0	0	0	1	0	0	0	0	0	0	0	0	0	0	0	9	0	0	0
Mazeran	BzMaz-10	U	RURAL	ERo	1900 - 1700	NMI	67	0	0	0	0	0	0	0	0	0	0	0	0	0	0	0	0	0	0	0	0	0	0	0	0	0	0	0
Médor	Medo-11	C	RURAL	LRo	1600 - 1400	NMI	280	0	0	0	0	0	0	0	0	0	0	2	1	0	0	0	0	0	1	1	0	0	0	2	2	1	0	8
Menhir (Le)	EyMen-6	C	RURAL	BA	3050 - 2800	NMI	328	0	0	0	0	0	0	0	0	0	0	0	0	0	0	0	0	0	0	0	0	0	0	0	0	0	0	0
Miremand	Mirmd-10	U	RURAL	ERo	2000 - 1900	NMI	788	0	0	0	0	0	3	0	0	0	0	0	1	0	0	0	0	0	0	171	0	0	0	0	0	0	0	0
Monédière (La)	Moned-7	C	URBAN	IA1	2575 - 2525	NMI	5392	0	0	0	0	0	0	0	0	0	0	0	0	0	0	0	0	0	0	0	0	0	0	0	0	0	0	0
Monédière (La)	Moned-8	C	URBAN	IA1/2	2525 - 2450	NMI	37913	0	0	0	0	0	0	0	0	0	0	0	0	0	0	0	0	0	0	0	0	0	1	0	0	0	0	0
Mont Ferrier	MtFer-10	U	RURAL	ERo	2000 - 1900	NMI	1438	0	0	0	0	0	79	0	0	0	0	1	0	0	0	0	0	0	0	158	0	1	0	20	1	0	0	0
Mont Joui	MtJo-8	C	VILLAGE	IA1/2	2525 - 2475	NMI	79	0	0	0	0	0	0	0	0	0	0	0	0	0	0	0	0	0	0	0	0	0	1	0	0	0	0	0
Mont Redon	MtRed-10	U	RURAL	ERo	1900 - 1800	NMI	373	0	0	0	0	0	0	0	0	0	0	0	0	0	0	0	0	0	0	2	0	0	0	0	0	0	0	0
Mont Redon	MtRed-11	U	RURAL	LRo	1700 - 1500	NMI	366	0	0	0	0	0	0	0	0	0	0	0	0	0	0	0	0	0	0	0	0	0	0	0	0	0	0	8
Montfau	MagMt-7	C	VILLAGE	IA1	2550 - 2475	NMI	19	0	0	0	0	0	0	0	0	0	0	0	0	0	0	0	0	0	0	0	0	0	0	0	0	0	0	0
Montjean	Mtjea-8	C	VILLAGE	IA1/2	2525 - 2475	NMI	813	0	0	0	0	0	0	0	0	0	0	0	0	0	0	0	0	0	0	0	0	0	0	0	0	0	0	0
Montou (Grotte de)	GtMto-9	C	CAVE	IA2	2425 - 2300	NMI	6756	0	0	0	0	0	0	0	0	0	0	0	0	0	0	0	0	0	8	0	0	0	0	1	0	0	0	0
Montou (Grotte de)	GtMto-4	C	CAVE	BA	4200 - 3650	NMI	5309	0	0	0	0	0	0	0	0	0	0	0	0	0	0	0	0	0	0	0	0	0	0	1	0	0	0	0
Montou (Grotte de)	GtMto-5a	C	CAVE	BA	4200 - 3350	NMI	1361	0	0	0	0	0	0	0	0	0	0	0	0	0	0	0	0	0	0	0	0	0	0	1	0	0	0	0
Montou (Grotte de)	GtMto-5b	C	CAVE	BA	3650 - 3350	NMI	563	0	0	0	0	0	0	0	0	0	0	0	0	0	0	0	0	0	1	0	0	0	0	3	0	0	0	0
Montou (Grotte de)	GtMto-5c	C	CAVE	BA	3650 - 3250	NMI	35	0	0	0	0	0	0	0	0	0	0	0	0	0	0	0	0	0	0	0	0	0	0	0	0	0	0	0
Montou (Grotte de)	GtMto-6	C	CAVE	BA	3350 - 2800	NMI	369	0	0	0	0	0	0	0	0	0	0	0	0	0	0	0	0	0	21	0	0	0	0	1	0	0	0	0
Mort des Anes	MtAne-3	C	CAVE	Neo	5500 - 5300	NMI	216	0	0	0	0	0	0	0	0	0	0	0	0	0	0	0	0	0	0	0	0	0	0	0	0	0	0	0

Site	Cod Sit-Phas	Pres	type	Period	DATE BP	Prsp	Pyru	Viti	Vitipd	Arbu	Corma	Corsa	Crata	Junsp	Myrt	Physa	Pincem	Pinsp	Pista	Prspi	Querc	Rhus	Rosa	Rubca	Rubfr	Rubid	Rubsp	Sambeb	Sambni	Sambsp	Sorb	Vibu	An crops
Logis de Berre (Le)	LoBer-2	C	RURAL	Neo	6000 - 5500	0	0	0	0	0	0	0	0	0	0	0	0	0	0	0	0	0	0	0	0	0	0	0	0	0	0	0	246
Lombard (Grotte)	GtLom-1	C	CAVE	Neo	7500 - 7000	0	0	0	0	0	0	0	0	0	0	0	0	0	0	0	1	0	0	0	0	0	0	0	0	0	0	0	0
Lycée Technique	MtpLT-7	C	RURAL	IA1	2700 - 2600	0	0	0	0	0	0	0	0	0	0	0	0	0	0	0	0	0	0	0	0	0	0	0	0	0	0	0	2
Machal (Le)	DaMch-4	C	RURAL	BA	4050 - 3600	0	0	1	0	0	0	0	1	0	0	0	0	0	0	0	0	0	0	0	0	0	0	0	0	0	0	0	161
Madeleine I	PeMa1-10	C	RURAL	ERo	2150 - 2075	0	0	1	0	0	0	0	0	0	0	0	0	0	0	0	0	0	0	0	0	0	0	0	0	0	0	0	1
Magasins (Les), Buzerens	MaBuz-9a	U	RURAL	IA2	2500 - 2400	0	0	0	0	0	0	0	0	0	0	0	0	0	0	0	0	0	0	0	2	0	0	2	0	0	0	0	0
Magasins (Les), Buzerens	MaBuz-9b	U	RURAL	IA2	2125 - 2050	0	0	6	0	0	0	0	0	0	0	0	0	0	0	2	0	0	0	0	15	0	0	12	1	0	0	0	0
Magasins (Les), Buzerens	MaBuz-9b	C	RURAL	IA2	2120 - 2050	0	0	0	0	0	0	0	0	0	0	0	0	0	0	0	0	0	0	0	0	0	0	0	0	0	0	0	95
Maison Philippon	ElMP-10	C	RURAL	ERo	2000 - 1900	0	0	0	0	0	0	0	0	0	0	0	0	0	0	0	0	0	0	0	0	0	0	0	0	0	0	0	6
Maladrerie/Lot. du Grand Chêne	MalGdC-11	C	RURAL	LRo	1800 - 1525	0	0	19	0	0	0	0	0	0	0	0	0	0	0	0	0	0	0	0	0	0	0	0	0	0	0	0	34
Marduel	Mrdu-6	C	VILLAGE	BA	2900 - 2750	0	0	0	0	0	0	0	0	0	0	0	0	0	0	0	0	0	0	0	0	0	0	0	0	0	0	0	16
Marduel	Mrdu-7	C	VILLAGE	IA1	2550 - 2500	0	0	0	0	0	0	0	0	0	0	0	0	0	0	0	0	0	0	0	0	0	0	0	0	0	0	0	112
Marduel	Mrdu-8	C	VILLAGE	IA1/2	2500 - 2450	0	0	20	0	0	0	0	0	0	0	0	0	0	0	0	0	0	0	0	0	0	0	0	0	0	0	0	185
Marduel	Mrdu-9a	C	VILLAGE	IA2	2450 - 2400	0	0	110	0	0	0	0	0	0	0	0	0	0	0	0	0	0	0	0	0	0	0	0	0	0	0	0	8371
Marduel	Mrdu-9b	C	VILLAGE	IA2	2250 - 2150	0	0	2	0	0	0	0	0	0	0	0	0	0	0	0	0	0	0	0	0	0	0	0	0	0	0	0	297
Martigues (île)	IlMart-9a	C	VILLAGE	IA2	2400 - 2375	0	0	831	84	0	0	0	0	0	0	0	0	0	0	0	0	0	5	0	0	0	0	0	0	0	0	0	50604
Martigues (île)	IlMart-9b	U	VILLAGE	IA2	2200 - 2200	0	0	0	0	0	0	0	0	0	0	0	0	0	0	0	0	0	0	0	0	0	0	0	0	0	0	0	0
Mas (Le)	AnsMa-Ro	C	RURAL	Rom	2025 - 1500	0	0	7	0	0	0	0	0	0	0	0	0	0	0	0	0	0	0	0	0	0	0	0	0	0	0	0	0
Mas Coste	PeMC-10	C	RURAL	ERo	2120 - 1940	0	0	28	1	0	0	0	0	0	0	0	0	0	0	0	0	0	0	0	0	0	0	0	0	0	0	0	9
Mas de Causse	LtMdC-7	C	RURAL	IA1	2600 - 2500	0	0	2	0	0	0	0	0	0	0	0	0	0	0	0	0	0	0	0	0	0	0	0	1	0	0	0	13
Mas de Causse	LtMdC-8	C	RURAL	IA1/2	2525 - 2400	0	0	25	0	0	0	0	0	0	0	0	0	0	0	0	0	0	0	0	0	0	0	10	26	0	0	0	32
Mas de Causse	LtMdC-9a	C	RURAL	IA2	2500 - 2300	0	0	10	0	0	0	0	0	0	0	0	0	0	0	0	0	0	0	0	0	0	0	9	23	0	0	0	2
Mas de Causse	LtMdC-9b	C	RURAL	IA2	2100 - 2000	0	0	20	0	0	0	0	0	0	0	0	0	0	0	0	0	0	0	0	0	0	0	0	0	0	0	0	3
Mas de Causse	LtMdC-10	C	RURAL	ERo	2000 - 1900	0	0	5	0	0	0	0	0	0	0	0	0	0	0	0	0	0	0	0	0	0	0	0	0	0	0	0	0
Mas de Causse	LtMdC-10	U	RURAL	ERo	2000 - 1900	0	0	338	63	0	0	0	0	0	0	0	0	0	0	0	0	0	0	0	10	0	0	6	3	0	0	0	5
Mas de Causse	LtMdC-11	C	RURAL	LRo	1800 - 1600	0	0	25	2	0	0	0	0	0	0	0	0	0	0	0	1	0	0	0	0	0	0	0	0	0	0	0	3
Mas de Causse	LtMdC-11	U	RURAL	LRo	1800 - 1600	0	0	29	3	0	0	0	0	0	0	0	0	0	0	0	0	0	0	0	2	0	0	51	5	0	0	0	1
Mas de Causse - Cultual	LtMdCC-9	C	FUN	IA2	2500 - 2300	0	0	3	0	0	0	0	0	0	0	0	0	0	0	0	0	0	0	0	0	0	0	0	1	0	0	0	9
Mas de Vignoles 10	MdV10-1	C	RURAL	Neo	7000 - 6500	0	0	0	0	0	0	0	0	0	0	0	0	0	0	0	0	0	0	0	0	0	0	0	0	0	0	0	1
Mas de Vignoles 10	MdV10-7	C	RURAL	IA1	2750 - 2625	0	0	4	0	0	1	0	0	0	0	0	0	0	0	0	0	0	0	0	0	0	0	0	0	0	0	0	37
Mas de Vignoles 9	MdV9-2	C	RURAL	Neo	6500 - 5500	0	0	0	0	0	0	0	0	0	0	0	0	0	0	0	0	0	0	0	0	0	0	0	0	0	0	0	1
Mas de Vignoles 9	MdV9-2	U	RURAL	Neo	6500 - 5500	0	0	0	0	0	0	0	0	0	0	0	0	0	0	0	0	0	0	0	0	0	0	0	0	0	0	0	5
Mas de Vignoles 9	MdV9-5	U	RURAL	BA	3650 - 1350	0	0	4	0	0	0	0	0	0	0	0	0	0	0	0	0	0	0	0	26	0	0	22	0	0	0	0	0
Mas de Vignoles 9	MdV9-7	C	RURAL	IA1	2750 - 2450	0	0	4	0	0	0	0	0	0	0	0	0	0	0	0	0	0	0	0	0	0	0	0	0	0	0	0	10
Mas de Vignoles 9	MdV9-7	U	RURAL	IA1	2750 - 2450	0	0	96	0	0	0	0	0	0	0	0	0	0	0	0	0	0	0	0	13	0	0	5	0	0	0	0	3
Mas de Vignoles 9	MdV9-5	C	RURAL	BA	2600 - 1350	0	0	0	0	0	0	0	0	0	0	0	0	0	0	0	0	0	0	0	0	0	0	0	0	0	0	0	0
Mas de Vignoles 9	MdV9-10	C	RURAL	ERo	2000 - 1900	0	0	0	0	0	0	0	0	0	0	0	0	0	0	0	0	0	0	0	0	0	0	0	0	0	0	0	0
Mas de Vignoles 9	MdV9-10	U	RURAL	ERo	2000 - 1900	0	0	210	0	0	0	0	0	0	0	0	0	0	0	0	0	0	0	0	12	0	0	0	0	0	0	0	4
Mas Delfau	PeMDe-10	C	RURAL	ERo	2100 - 1900	0	0	5	0	0	0	0	0	0	0	0	0	0	0	0	0	0	0	0	2	0	0	0	0	0	0	0	494
Mas des Abeilles	NMdAb-3	C	RURAL	Neo	5500 - 4200	0	0	0	0	0	0	0	0	0	0	0	0	0	0	0	0	0	0	0	0	0	0	0	0	0	0	0	18490
Mas des Abeilles	NMdAb-7	C	RURAL	IA1	2700 - 2600	0	0	0	0	0	0	0	0	0	0	0	0	0	0	0	0	0	0	0	0	0	0	0	0	0	0	0	5
Mas Neuf	NMNeu-1	C	RURAL	Neo	7000 - 6500	0	0	0	0	0	0	0	0	0	0	0	0	0	0	0	0	0	0	0	0	0	0	0	0	0	0	0	47450
Mas Roig	MRoig-10	C	RURAL	ERo	1990 - 1970	0	0	0	0	0	0	0	0	0	0	0	0	0	0	0	0	0	0	0	0	0	0	0	0	0	0	0	1
Mas Sauvy	VRaMS-10	C	RURAL	ERo	1940 - 1880	0	0	0	0	0	0	0	0	0	0	0	0	0	0	0	0	0	0	0	0	0	0	0	0	0	0	0	35
Mazeran	BzMaz-10	U	RURAL	ERo	1900 - 1700	0	0	63	0	0	0	0	0	0	0	0	0	0	0	2	0	0	0	0	0	0	0	0	0	2	0	0	0
Médor	Medo-11	C	RURAL	LRo	1600 - 1400	0	0	86	0	0	0	1	0	0	0	0	0	0	0	0	0	0	0	0	0	0	0	0	0	0	0	0	175
Menhir (Le)	EyMen-6	C	RURAL	BA	3050 - 2800	0	0	0	0	0	0	0	0	0	0	0	0	0	0	0	1	0	0	0	0	0	0	0	0	0	0	0	327
Miremand	Mirmd-10	U	RURAL	ERo	2000 - 1900	0	0	56	10	0	0	0	0	0	0	0	0	0	0	0	0	0	0	0	333	0	0	168	10	0	0	0	36
Monédière (La)	Moned-7	C	URBAN	IA1	2575 - 2525	0	0	1	0	0	0	0	0	0	0	0	0	0	0	0	0	0	0	0	0	0	1	1	0	0	0	0	5389
Monédière (La)	Moned-8	C	URBAN	IA1/2	2525 - 2450	0	0	11	0	0	0	0	0	0	0	0	0	0	0	0	0	0	0	0	0	0	0	0	0	0	0	0	37901
Mont Ferrier	MtFer-10	U	RURAL	ERo	2000 - 1900	0	0	999	137	0	0	0	0	0	0	0	0	0	0	0	7	0	0	0	4	0	0	1	0	0	0	0	30
Mont Joui	MtJo-8	C	VILLAGE	IA1/2	2525 - 2475	0	0	3	0	0	0	0	0	0	0	0	0	0	0	0	0	0	0	0	0	0	0	0	0	0	0	0	75
Mont Redon	MtRed-10	U	RURAL	ERo	1900 - 1800	0	0	4	0	0	0	0	0	0	0	0	0	0	0	0	0	0	0	0	357	0	0	0	7	0	0	0	3
Mont Redon	MtRed-11	U	RURAL	LRo	1700 - 1500	0	0	130	22	0	0	0	0	0	0	0	0	0	0	0	0	0	0	0	22	0	0	121	0	0	0	0	63
Montfau	MagMt-7	C	VILLAGE	IA1	2550 - 2475	0	0	0	0	0	0	0	0	0	0	0	0	0	0	0	0	0	0	0	0	0	0	0	0	0	0	0	19
Montjean	Mtjea-8	C	VILLAGE	IA1/2	2525 - 2475	0	0	0	0	0	0	0	0	0	0	0	0	0	0	0	0	0	0	0	0	0	0	0	0	0	0	0	813
Montou (Grotte de)	GtMto-9	C	CAVE	IA2	2425 - 2300	0	0	6	0	1	0	0	0	0	0	0	0	0	0	0	0	0	0	0	0	0	0	0	0	0	0	0	6740
Montou (Grotte de)	GtMto-4	C	CAVE	BA	4200 - 3650	0	0	0	0	0	0	0	0	0	0	0	0	0	0	0	47	0	0	0	0	0	0	0	0	0	0	0	5261
Montou (Grotte de)	GtMto-5a	C	CAVE	BA	4200 - 3350	0	0	1	0	0	0	0	0	0	0	0	0	0	0	0	317	0	0	0	0	0	0	0	0	0	0	0	1042
Montou (Grotte de)	GtMto-5b	C	CAVE	BA	3650 - 3350	0	0	2	0	0	0	0	0	0	0	0	0	0	0	0	181	0	0	0	0	0	0	0	0	0	0	0	376
Montou (Grotte de)	GtMto-5c	C	CAVE	BA	3650 - 3250	0	0	0	0	0	0	0	0	0	0	0	0	0	0	0	2	0	0	0	0	0	0	0	0	0	0	0	33
Montou (Grotte de)	GtMto-6	C	CAVE	BA	3350 - 2800	0	0	7	0	0	0	0	0	0	0	0	0	0	0	0	78	0	0	0	0	0	0	0	0	0	0	0	262
Mort des Anes	MtAne-3	C	CAVE	Neo	5500 - 5300	0	0	0	0	0	0	0	0	0	0	0	0	0	0	0	0	0	0	0	0	0	0	0	0	0	0	0	216

Site	Cod Sit-Phas	Pres	type	Period	DATE BP	Quantif	MNI	Citrul	Cumel	Cupre	Cusat	Cusp	Jugla	Lage	Mespi	Moru	Prcer	Prdul	Prpers	Punic	Zizip	Cordi	Phoen	Celti	Coryl	Ficu	Fraga	Maloid	Malu	Olea	Pinpi	Pravce	Pravi	Prdom
Motte (La)	AgMot-6	U	RURAL	BA	2900 - 2750	NMI	479	0	0	0	0	0	0	0	0	0	0	0	0	0	0	0	0	0	0	89	25	0	0	0	0	0	0	0
Motte (La)	AgMot-6	C	RURAL	BA	2800 - 2700	NMI	48	0	0	0	0	0	0	0	0	0	0	0	0	0	0	0	0	0	0	0	0	0	0	0	0	0	0	0
Moulin (Le)	Lmoul-2	C	RURAL	Neo	6500 - 6000	NMI	157	0	0	0	0	0	0	0	0	0	0	0	0	0	0	0	0	0	0	0	0	0	1	0	0	0	0	0
Moulin à Vent (Le)	MoVt-10	C	RURAL	ERo	2025 - 1900	NMI	5	0	0	0	0	0	0	0	0	0	0	0	0	0	0	0	0	0	0	0	0	0	0	0	0	0	0	0
Moulin à Vent (Le)	MoVt-10	U	RURAL	ERo	2025 - 1900	NMI	36287	0	0	10	0	0	29	0	0	0	0	1	0	4	0	0	0	0	1	3548	0	3	0	21	3	0	0	1
Moulin d'eau	Moeau-7	C	RURAL	IA1	2850 - 2650	NMI	356	0	0	0	0	0	0	0	0	0	0	0	0	0	0	0	0	0	0	0	0	0	0	0	0	0	0	0
Moulins (Les)	SP3CMo-2	C	RURAL	Neo	6000 - 5500	NMI	1953	0	0	0	0	0	0	0	0	0	0	0	0	0	0	0	0	0	1	0	0	0	0	0	0	0	0	0
Mourre de la Barque	ModBq-5	C	CAVE	BA	3650 - 3250	NMI	42	0	0	0	0	0	0	0	0	0	0	0	0	0	0	0	0	0	1	0	0	0	0	0	0	0	0	0
Mourre de la Barque	ModBq-6	C	CAVE	BA	2900 - 2750	NMI	5	0	0	0	0	0	0	0	0	0	0	0	0	0	0	0	0	0	0	0	0	0	0	0	0	0	0	0
Mourre de Sève	SoMDS-7	C	VILLAGE	IA1	2550 - 2500	NMI	4394	0	0	0	0	0	0	0	0	0	0	0	0	0	0	0	0	0	0	0	0	0	0	4	0	0	0	0
Mourrel-Ferrat	MoFer-9	C	URBAN	IA2	2325 - 2275	NMI	1269	0	0	0	0	0	0	0	0	0	0	0	0	0	0	0	0	0	0	1	0	0	0	0	0	0	0	0
Murée (Grotte)	GtMur-5	C	CAVE	BA	3600 - 3500	NMI	206	0	0	0	0	0	0	0	0	0	0	0	0	0	0	0	0	0	0	0	0	0	0	0	0	0	0	0
Nages	Nage-10	U	RURAL	ERo	2000 - 1999	P/A	15	0	0	0	0	0	3	0	0	0	0	0	2	0	0	0	0	0	2	0	0	0	0	2	1	2	0	1
Nautique (La)	Nautiq-10	U	RURAL	ERo	2030 - 1990	NMI	40212	0	0	1	0	0	51	0	0	21	0	28	0	0	0	0	0	0	13	3349	0	0	0	160	9	0	2	0
Nautique (La)	Nautiq-10	C	RURAL	ERo	2025 - 1750	NMI	6	0	0	0	0	0	0	0	0	0	0	0	0	0	0	0	0	0	1	0	0	0	0	5	0	0	0	0
Nogeiret (Le)	BoNgrt-4	C	RURAL	BA	4200 - 3650	NMI	126	0	0	0	0	0	0	0	0	0	0	0	0	0	0	0	0	0	0	0	0	0	0	0	0	0	0	0
Nogeiret (Le)	BoNgrt-6	C	RURAL	BA	2900 - 2750	NMI	63	0	0	0	0	0	0	0	0	0	0	0	0	0	0	0	0	0	0	0	0	0	0	0	0	0	0	0
Notre Dame de Pitié	NDPit-9	C	VILLAGE	IA2	2220 - 2190	NMI	3	0	0	0	0	0	0	0	0	0	0	0	0	0	0	0	0	0	0	0	0	0	0	3	0	0	0	0
Opme le Cimetière	OpCim-4	C	RURAL	BA	4135 - 3830	NMI	36300	0	0	0	0	0	0	0	0	0	0	0	0	0	0	0	0	0	0	0	0	0	0	0	0	0	0	0
Pampelune (Roc de)	RcPamp-11	C	RURAL	LRo	1700 - 1500	NMI	16733	0	0	0	0	0	0	0	0	0	0	0	0	0	0	0	0	0	0	0	0	0	0	2	0	0	0	0
Parc Saint Georges	LyPSG-9	U	URBAN	IA2	2200 - 2075	NMI	17	0	0	0	0	0	0	0	0	0	0	0	0	0	0	0	0	0	0	2	0	0	0	0	0	0	0	0
Parc Saint Georges	LyPSG-10	C	URBAN	ERo	2000 - 1875	NMI	6	0	0	0	0	0	0	0	0	0	0	0	0	0	0	0	0	0	0	0	0	0	0	0	0	0	0	0
Parc Saint Georges	LyPSG-10	U	URBAN	ERo	2000 - 1875	NMI	3583	0	38	0	0	0	44	1	0	10	0	0	0	0	0	0	0	0	13	2931	20	0	5	2	0	0	13	0
Parc Saint Georges	LyPSG-11a	C	URBAN	LRo	1775 - 1700	NMI	41	0	0	0	0	0	0	0	0	0	0	0	0	0	0	0	0	0	0	0	0	0	0	1	0	0	0	0
Parc Saint Georges	LyPSG-11a	U	URBAN	LRo	1775 - 1700	NMI	5388	0	45	0	5	0	941	2	6	58	0	2	23	0	0	0	0	0	167	2069	144	0	58	52	0	0	126	15
Parc Saint Georges	LyPSG-11b	C	URBAN	LRo	1625 - 1575	NMI	8	0	0	0	0	0	0	0	0	0	0	0	0	0	0	0	0	0	0	0	0	0	0	0	0	0	0	0
Parc Saint Georges	LyPSG-11b	U	URBAN	LRo	1625 - 1575	NMI	28	0	0	0	0	0	0	0	0	0	0	0	0	0	0	0	0	0	0	0	0	0	0	0	0	0	0	0
Parking Jean Jaurès	NPJJ-9	C	URBAN	IA2	2150 - 2050	NMI	18	0	0	0	0	0	0	0	0	0	0	0	0	0	0	0	0	0	0	0	0	0	0	0	0	0	0	0
Parking Jean Jaurès	NPJJ-10	U	URBAN	ERo	2025 - 1800	NMI	9200	0	0	0	0	557	314	0	0	63	0	0	19	0	9	2	0	0	48	6851	0	0	7	37	12	0	15	4
Parking Jean Jaurès	NPJJ-10	C	URBAN	ERo	2025 - 1700	NMI	2097	0	0	0	0	0	1	0	0	0	0	0	2	0	0	0	0	0	0	0	0	0	0	2000	1	0	0	0
Parking Jean Jaurès	NPJJ-11	C	URBAN	LRo	1625 - 1475	NMI	4	0	0	0	0	0	1	0	0	0	0	0	0	0	0	0	0	0	0	0	0	0	0	2	0	0	0	0
Parking Jean Jaurès	NPJJ-11	U	URBAN	LRo	1625 - 1475	NMI	1199	0	0	0	0	0	14	0	0	0	0	1	0	0	0	0	0	0	0	286	0	0	0	5	0	0	7	4
Pâtural 2 (Le)	CFPt2-7	C	RURAL	IA1	2650 - 2520	NMI	38	0	0	0	0	0	0	0	0	0	0	0	0	0	0	0	0	0	0	0	0	0	0	0	0	0	0	0
Pauvadou	FrPauv-10	C	FUN	ERo	1950 - 1800	NMI	3	0	0	0	0	0	0	0	0	0	0	0	0	0	0	0	1	0	0	1	0	0	0	0	0	0	0	0
Pech de Tardieu	PedTa-11	C	RURAL	LRo	1750 - 1300	NMI	176	0	0	0	0	0	0	0	0	0	0	0	0	0	0	0	0	0	0	0	0	0	0	0	0	0	0	0
Pech Maho	PeMah-7	C	VILLAGE	IA1	2550 - 2425	NMI	214	0	0	0	0	0	0	0	0	0	0	0	0	0	0	0	0	0	0	0	0	0	0	0	0	0	0	0
Pech Maho	PeMah-9	C	VILLAGE	IA2	2425 - 2175	NMI	11477	0	0	0	0	0	0	0	0	0	0	0	0	0	0	0	0	0	0	0	0	0	0	4	0	0	0	0
Peigros	Peigr-7	C	VILLAGE	IA1	2600 - 2500	P/A	116	0	0	0	0	0	0	0	0	0	0	0	0	0	0	0	0	0	0	0	0	0	0	0	0	0	0	0
Peiro Signado	PoPS-1	C	RURAL	Neo	7750 - 7600	NMI	283	0	0	0	0	0	0	0	0	0	0	0	0	0	0	0	0	0	0	0	0	0	0	0	0	0	0	0
Pendimoum (Abri)	APend-1a	C	CAVE	Neo	7750 - 7500	NMI	6	0	0	0	0	0	0	0	0	0	0	0	0	0	0	0	0	0	1	0	0	0	0	0	0	0	0	0
Pendimoum (Abri)	APend-1b	C	CAVE	Neo	7500 - 7000	NMI	2	0	0	0	0	0	0	0	0	0	0	0	0	0	0	0	0	0	0	0	0	0	0	0	0	0	0	0
Peras 1	MaPe1-2	C	RURAL	Neo	6500 - 5500	NMI	2	0	0	0	0	0	0	0	0	0	0	0	0	0	0	0	0	0	0	0	0	0	0	0	0	0	0	0
Peras 1	MaPe1-3	C	RURAL	Neo	5500 - 4200	NMI	6	0	0	0	0	0	0	0	0	0	0	0	0	0	0	0	0	0	0	0	0	0	0	0	0	0	0	0
Peras 2	MaPe2-2	C	RURAL	Neo	6500 - 5500	NMI	159	0	0	0	0	0	0	0	0	0	0	0	0	0	0	0	0	0	0	0	0	0	0	0	0	0	0	0
Petit Beaulieu/Puy Long	PtBPL-4	C	RURAL	BA	3900 - 3750	NMI	5772	0	0	0	0	0	0	0	0	0	0	0	0	0	0	0	0	0	2	0	0	0	0	0	0	0	0	0
Petit Clos I	PePC1-10	C	RURAL	ERo	2020 - 1850	NMI	5866	0	0	1	0	0	1	0	0	0	0	1	0	0	0	0	0	0	1	6	0	0	0	1	266	0	0	0
Petit Garlambaud	PtGrl-6	C	RURAL	BA	3350 - 3100	NMI	647	0	0	0	0	0	0	0	0	0	0	0	0	0	0	0	0	0	0	0	0	0	0	0	0	0	0	0
Petites Bâties	PtBat-1	C	RURAL	Neo	7500 - 7000	P/A	4	0	0	0	0	0	0	0	0	0	0	0	0	0	0	0	0	0	0	0	0	0	0	0	0	0	0	0
Pierre Audry (Rue)	LyRPA-10	C	FUN	ERo	1950 - 1800	NMI	11	0	0	0	0	0	0	0	0	0	0	0	1	0	0	0	1	0	1	2	0	0	0	0	2	0	0	0
Pierredon	Pierr-9	C	VILLAGE	IA2	2225 - 2100	NMI	1850	0	0	0	0	0	0	0	0	0	0	0	0	0	0	0	0	0	0	0	0	0	0	0	0	0	0	0
Pirou (Le)	VPiro-4	C	RURAL	BA	4200 - 3600	NMI	486	0	0	0	0	0	0	0	0	0	0	0	0	0	0	0	0	0	0	0	0	0	0	0	0	0	0	0
Pla de l'Aïgo	CaPla-10	C	RURAL	ERo	1950 - 1650	NMI	13	0	0	0	0	0	0	0	0	0	0	0	0	0	0	0	0	0	0	0	0	0	0	0	0	0	0	0
Pla de Molas	PlMol-9	C	RURAL	IA2	2150 - 2100	NMI	58	0	0	0	0	0	0	0	0	0	0	0	0	0	0	0	0	0	0	0	0	0	0	1	0	0	0	0
Pla de Peyre	PdPey-3	C	RURAL	Neo	5500 - 5000	NMI	56	0	0	0	0	0	0	0	0	0	0	0	0	0	0	0	0	0	2	0	0	0	0	0	0	0	0	0
Plafête	Plafet-Bz	C	RURAL	BA	4200 - 2800	NMI	19	0	0	0	0	0	0	0	0	0	0	0	0	0	0	0	0	0	0	0	0	0	0	0	0	0	0	0
Plaines (Les)	CoPla-4	C	RURAL	BA	4100 - 3600	NMI	26	0	0	0	0	0	0	0	0	0	0	0	0	0	0	0	0	0	0	0	0	1	0	0	0	0	0	0
Plaines (Les)	CoPla-6	C	RURAL	BA	3350 - 2800	NMI	2	0	0	0	0	0	0	0	0	0	0	0	0	0	0	0	0	0	0	0	0	0	0	0	0	0	0	0
Plan Tour	GaPdT-8	C	VILLAGE	IA1/2	2500 - 2400	NMI	1756	0	0	0	0	0	0	0	0	0	0	0	0	0	0	0	0	0	0	0	0	0	0	0	0	0	0	0
Plan Tour	GaPdT-9	C	VILLAGE	IA2	2400 - 2300	NMI	10137	0	0	0	0	0	0	0	0	0	0	0	0	0	0	0	0	0	0	0	0	0	0	0	0	0	0	0
Plateau Raverre	PtRav-2	C	RURAL	Neo	6500 - 6000	NMI	66	0	0	0	0	0	0	0	0	0	0	0	0	0	0	0	0	0	1	0	0	0	0	0	0	0	0	0
Plots (Les)	Plots-2	C	RURAL	Neo	6500 - 6000	NMI	75	0	0	0	0	0	0	0	0	0	0	0	0	0	0	0	0	0	1	0	0	0	0	0	0	0	0	0
Pont de Roque Haute	PoPRH-1	C	RURAL	Neo	7750 - 7600	NMI	28	0	0	0	0	0	0	0	0	0	0	0	0	0	0	0	0	0	0	0	0	0	0	0	0	0	0	0
Pontiar (Grotte du)	GtPon-7	C	CAVE	IA1	2550 - 2550	%	104	0	0	0	0	0	0	0	0	0	0	0	0	0	0	0	0	0	0	0	0	0	0	0	0	0	0	0

Site	Cod Sit-Phas	Pres	type	Period	DATE BP	Prsp	Pyru	Viti	Vitipd	Arbu	Corma	Corsa	Crata	Junsp	Myrt	Physa	Pincem	Pinsp	Pista	Prspi	Querc	Rhus	Rosa	Rubca	Rubfr	Rubid	Rubsp	Sambeb	Sambni	Sambsp	Sorb	Vibu	An crops
Motte (La)	AgMot-6	U	RURAL	BA	2900 - 2750	0	0	20	2	0	0	0	2	2	0	0	0	0	2	15	42	0	1	5	37	0	0	32	5	0	0	0	200
Motte (La)	AgMot-6	C	RURAL	BA	2800 - 2700	0	0	0	0	0	0	0	0	0	0	0	0	0	0	0	1	0	0	0	0	0	0	0	0	0	0	0	46
Moulin (Le)	Lmoul-2	C	RURAL	Neo	6500 - 6000	0	0	0	0	0	0	0	0	0	0	0	0	0	0	1	0	0	0	0	0	0	0	0	0	1	0	0	155
Moulin à Vent (Le)	MoVt-10	C	RURAL	ERo	2025 - 1900	0	0	5	0	0	0	0	0	0	0	0	0	0	0	0	0	0	0	0	0	0	0	0	0	0	0	0	0
Moulin à Vent (Le)	MoVt-10	U	RURAL	ERo	2025 - 1900	0	0	26619	4273	0	0	0	0	0	0	5	0	0	0	0	8	0	0	0	1492	0	0	0	21	0	0	0	248
Moulin d'eau	Moeau-7	C	RURAL	IA1	2850 - 2650	0	0	0	0	0	0	0	0	0	0	0	0	0	0	0	0	0	0	0	0	0	0	0	0	0	0	0	356
Moulins (Les)	SP3CMo-2	C	RURAL	Neo	6000 - 5500	0	0	16	0	0	0	0	0	0	0	0	0	0	0	0	0	0	0	0	0	0	0	0	0	0	0	0	1936
Mourre de la Barque	ModBq-5	C	CAVE	BA	3650 - 3250	0	0	1	0	0	4	0	0	0	0	0	0	0	0	0	1	0	0	0	0	0	0	0	0	0	0	0	35
Mourre de la Barque	ModBq-6	C	CAVE	BA	2900 - 2750	0	0	0	0	0	4	0	0	0	0	0	0	0	0	0	0	0	0	0	0	0	0	0	0	0	0	0	1
Mourre de Sève	SoMDS-7	C	VILLAGE	IA1	2550 - 2500	0	0	949	31	0	0	0	0	0	0	0	0	0	0	0	0	0	0	0	0	0	0	0	0	0	0	0	3410
Mourrel-Ferrat	MoFer-9	C	URBAN	IA2	2325 - 2275	0	0	221	0	0	0	0	0	0	0	0	0	0	0	0	0	0	0	0	1	0	0	0	0	0	0	0	1046
Murée (Grotte)	GtMur-5	C	CAVE	BA	3600 - 3500	0	0	0	0	0	5	0	0	0	0	0	0	0	0	0	0	0	0	0	0	0	0	0	0	0	0	0	201
Nages	Nage-10	U	RURAL	ERo	2000 - 1999	0	0	0	0	0	0	0	0	0	0	0	0	0	0	0	1	0	0	0	0	0	0	0	0	0	0	0	0
Nautique (La)	Nautiq-10	U	RURAL	ERo	2030 - 1990	0	0	22118	13865	0	0	0	0	0	0	10	0	0	0	0	1	0	4	0	487	0	0	0	11	0	0	0	82
Nautique (La)	Nautiq-10	C	RURAL	ERo	2025 - 1750	0	0	0	0	0	0	0	0	0	0	0	0	0	0	0	0	0	0	0	0	0	0	0	0	0	0	0	0
Nogeiret (Le)	BoNgrt-4	C	RURAL	BA	4200 - 3650	0	0	0	0	0	0	0	0	0	0	0	0	0	0	0	0	0	0	0	0	0	0	0	0	0	0	0	126
Nogeiret (Le)	BoNgrt-6	C	RURAL	BA	2900 - 2750	0	0	0	0	0	0	0	0	0	0	0	0	0	0	4	0	0	0	0	0	0	0	0	0	0	0	0	59
Notre Dame de Pitié	NDPit-9	C	VILLAGE	IA2	2220 - 2190	0	0	0	0	0	0	0	0	0	0	0	0	0	0	0	0	0	0	0	0	0	0	0	0	0	0	0	0
Opme le Cimetière	OpCim-4	C	RURAL	BA	4135 - 3830	0	0	0	0	0	0	0	0	0	0	0	0	0	0	0	0	0	0	0	0	0	0	0	0	0	0	0	36300
Pampelune (Roc de)	RcPamp-11	C	RURAL	LRo	1700 - 1500	0	0	2	0	0	0	0	0	0	0	0	0	0	0	0	0	0	0	0	0	0	0	0	0	0	0	0	16729
Parc Saint Georges	LyPSG-9	U	URBAN	IA2	2200 - 2075	0	0	1	0	0	0	1	1	0	0	0	0	0	0	0	0	0	0	0	2	3	0	4	3	0	0	0	0
Parc Saint Georges	LyPSG-10	C	URBAN	ERo	2000 - 1875	0	0	2	0	0	0	0	0	0	0	0	0	0	0	0	0	0	0	0	0	0	0	0	0	0	0	0	4
Parc Saint Georges	LyPSG-10	U	URBAN	ERo	2000 - 1875	0	0	59	15	0	0	11	3	0	0	1	0	0	0	4	0	0	5	6	56	0	0	56	9	0	0	0	281
Parc Saint Georges	LyPSG-11a	C	URBAN	LRo	1775 - 1700	0	0	1	0	0	0	0	0	0	0	0	0	0	0	0	0	0	0	0	0	0	0	0	0	0	0	0	39
Parc Saint Georges	LyPSG-11a	U	URBAN	LRo	1775 - 1700	0	0	327	162	0	0	68	44	0	0	8	0	0	0	2	22	0	24	1	89	18	0	392	68	0	0	0	450
Parc Saint Georges	LyPSG-11b	C	URBAN	LRo	1625 - 1575	0	0	0	0	0	0	0	0	0	0	0	0	0	0	0	0	0	0	0	0	0	0	0	0	0	0	0	8
Parc Saint Georges	LyPSG-11b	U	URBAN	LRo	1625 - 1575	0	0	0	0	0	0	0	0	0	0	0	0	0	0	0	0	0	0	0	0	0	0	23	5	0	0	0	0
Parking Jean Jaurès	NPJJ-9	C	URBAN	IA2	2150 - 2050	0	0	14	0	0	0	0	0	0	0	0	0	0	0	0	0	0	0	0	0	0	0	0	0	0	0	0	4
Parking Jean Jaurès	NPJJ-10	U	URBAN	ERo	2025 - 1800	0	5	1072	17	0	0	0	0	4	0	0	0	0	23	0	19	0	12	0	51	0	0	3	0	0	0	0	56
Parking Jean Jaurès	NPJJ-10	C	URBAN	ERo	2025 - 1700	0	0	36	0	0	0	0	0	0	0	0	0	0	0	0	24	0	0	0	0	0	0	0	0	0	0	0	33
Parking Jean Jaurès	NPJJ-11	C	URBAN	LRo	1625 - 1475	0	0	0	0	0	0	0	0	0	0	0	0	0	0	0	0	0	0	0	1	0	0	0	0	0	0	0	0
Parking Jean Jaurès	NPJJ-11	U	URBAN	LRo	1625 - 1475	0	0	650	28	0	0	0	0	0	0	26	0	0	0	2	0	0	6	0	51	0	0	70	6	0	0	0	43
Pâtural 2 (Le)	CFPt2-7	C	RURAL	IA1	2650 - 2520	0	0	0	0	0	0	0	0	0	0	0	0	0	0	0	0	0	0	0	0	0	0	0	0	4	0	0	34
Pauvadou	FrPauv-10	C	FUN	ERo	1950 - 1800	0	0	0	0	0	0	0	0	0	0	0	0	0	0	0	0	0	0	0	0	0	0	0	0	0	0	0	1
Pech de Tardieu	PedTa-11	C	RURAL	LRo	1750 - 1300	0	0	116	2	0	0	0	0	0	0	1	0	0	3	0	0	0	0	0	0	0	0	0	0	0	0	0	54
Pech Maho	PeMah-7	C	VILLAGE	IA1	2550 - 2425	0	0	1	0	0	0	0	0	0	0	0	0	0	0	0	0	0	0	0	0	0	0	0	0	0	0	0	213
Pech Maho	PeMah-9	C	VILLAGE	IA2	2425 - 2175	0	0	519	1	0	0	0	0	0	0	0	0	0	0	0	0	0	0	0	0	0	0	0	0	0	0	0	10953
Peigros	Peigr-7	C	VILLAGE	IA1	2600 - 2500	0	0	0	0	0	0	0	0	0	0	0	0	0	0	0	0	0	0	0	0	0	0	0	0	0	0	0	116
Peiro Signado	PoPS-1	C	RURAL	Neo	7750 - 7600	0	0	1	0	0	0	0	0	0	0	0	0	0	1	0	0	0	0	0	0	0	0	0	0	0	0	0	281
Pendimoum (Abri)	APend-1a	C	CAVE	Neo	7750 - 7500	0	0	1	0	0	0	0	0	0	0	0	0	0	0	0	0	0	0	0	0	0	0	0	0	0	0	0	4
Pendimoum (Abri)	APend-1b	C	CAVE	Neo	7500 - 7000	0	0	0	0	0	0	0	0	0	0	0	0	0	0	0	0	0	0	0	0	0	0	0	0	0	0	0	2
Peras 1	MaPe1-2	C	RURAL	Neo	6500 - 5500	0	0	0	0	0	0	0	0	0	0	0	0	0	0	0	1	0	0	0	0	0	0	0	0	0	0	0	1
Peras 1	MaPe1-3	C	RURAL	Neo	5500 - 4200	0	0	0	0	0	0	0	0	0	0	0	0	0	0	0	0	0	0	0	0	0	0	0	0	0	0	0	6
Peras 2	MaPe2-2	C	RURAL	Neo	6500 - 5500	0	0	0	0	0	0	0	0	0	0	0	0	0	0	0	0	0	0	0	0	0	0	0	0	0	0	0	159
Petit Beaulieu/Puy Long	PtBPL-4	C	RURAL	BA	3900 - 3750	0	0	0	0	0	0	0	0	0	0	0	0	0	0	0	0	0	2	0	0	0	0	2	0	0	0	0	5766
Petit Clos I	PePC1-10	C	RURAL	ERo	2020 - 1850	0	0	1442	358	0	0	0	0	0	0	0	0	0	0	0	0	0	0	0	0	0	0	0	0	0	0	0	3789
Petit Garlambaud	PtGrl-6	C	RURAL	BA	3350 - 3100	0	0	1	1	0	0	0	0	0	0	0	0	0	0	0	223	0	0	0	0	0	0	0	0	0	0	0	422
Petites Bâties	PtBat-1	C	RURAL	Neo	7500 - 7000	0	0	*0*	0	*0*	0	*0*	0	*0*	0	0	0	*0*	*0*	*0*	*0*	*0*	*0*	0	*0*	0	*0*	*0*	*0*	*0*	*0*	*0*	4
Pierre Audry (Rue)	LyRPA-10	C	FUN	ERo	1950 - 1800	0	0	2	0	0	0	0	0	0	0	0	0	0	0	0	0	0	0	0	0	0	0	0	0	0	0	0	2
Pierredon	Pierr-9	C	VILLAGE	IA2	2225 - 2100	0	0	15	3	0	0	0	0	0	0	0	0	0	0	0	0	0	0	0	0	0	0	0	0	0	0	0	1832
Pirou (Le)	VPiro-4	C	RURAL	BA	4200 - 3600	0	0	0	0	0	0	0	0	0	0	0	0	0	0	0	465	0	0	0	0	0	0	0	0	0	0	0	21
Pla de l'Aïgo	CaPla-10	C	RURAL	ERo	1950 - 1650	0	0	8	0	0	0	0	0	0	0	0	0	0	0	0	0	0	0	0	0	0	0	0	0	0	0	0	5
Pla de Molas	PlMol-9	C	RURAL	IA2	2150 - 2100	0	0	3	0	1	0	0	0	0	0	0	0	0	0	0	0	0	0	0	1	0	0	0	0	0	0	0	52
Pla de Peyre	PdPey-3	C	RURAL	Neo	5500 - 5000	0	0	0	0	0	0	0	0	0	0	0	0	0	0	0	0	0	0	0	0	0	0	0	0	0	0	0	54
Plafète	Plafet-Bz	C	RURAL	BA	4200 - 2800	0	0	0	0	0	0	0	0	0	0	0	0	0	0	0	0	0	0	0	0	0	0	0	0	0	0	0	19
Plaines (Les)	CoPla-4	C	RURAL	BA	4100 - 3600	1	0	1	0	0	0	0	0	0	0	0	0	0	0	0	0	0	0	0	0	0	0	0	0	0	0	0	23
Plaines (Les)	CoPla-6	C	RURAL	BA	3350 - 2800	0	0	0	0	0	0	0	0	0	0	0	0	0	0	0	0	0	0	0	0	0	0	0	0	0	0	0	2
Plan Tour	GaPdT-8	C	VILLAGE	IA1/2	2500 - 2400	0	0	43	0	0	1	0	0	0	0	0	0	0	0	0	1	0	0	0	0	0	0	0	0	0	0	0	1711
Plan Tour	GaPdT-9	C	VILLAGE	IA2	2400 - 2300	0	0	7	0	0	0	0	0	0	0	0	0	0	0	0	1	0	0	0	0	0	0	0	0	0	0	0	10129
Plateau Raverre	PtRav-2	C	RURAL	Neo	6500 - 6000	0	0	0	0	0	0	0	0	0	0	0	0	0	0	0	34	0	0	0	0	0	0	0	0	0	0	0	31
Plots (Les)	Plots-2	C	RURAL	Neo	6500 - 6000	0	0	0	0	0	0	0	0	0	0	0	0	0	0	1	0	0	0	0	0	0	0	0	0	0	0	0	73
Pont de Roque Haute	PoPRH-1	C	RURAL	Neo	7750 - 7600	0	0	0	0	0	0	0	0	0	0	0	0	0	0	0	0	0	0	0	0	0	0	0	0	0	0	0	28
Pontiar (Grotte du)	GtPon-7	C	CAVE	IA1	2550 - 2550	0	0	0	0	0	0	0	0	0	0	0	0	0	0	0	0	0	0	0	0	0	0	0	0	0	0	0	104

Site	Cod Sit-Phas	Pres	type	Period	DATE BP	Quantif	MNI	Citrul	Cumel	Cupre	Cusat	Cusp	Jugla	Lage	Mespi	Moru	Prcer	Prdul	Prpers	Punic	Zizip	Cordi	Phoen	Celti	Coryl	Ficu	Fraga	Maloid	Malu	Olea	Pinpi	Pravce	Pravi	Prdom
Port Ariane	LtPA-6a	U	RURAL	BA	3400 - 3200	NMI	9	0	0	0	0	0	0	0	0	0	0	0	0	0	0	0	0	0	0	0	0	0	0	0	0	0	0	0
Port Ariane	LtPA-6a	C	RURAL	BA	3400 - 3200	NMI	1	0	0	0	0	0	0	0	0	0	0	0	0	0	0	0	0	0	0	0	0	0	0	0	0	0	0	0
Port Ariane	LtPA-6b	C	RURAL	BA	3200 - 3000	NMI	39	0	0	0	0	0	0	0	0	0	0	0	0	0	0	0	0	0	0	0	0	0	0	0	0	0	0	0
Port Ariane	LtPA-6b	U	RURAL	BA	3200 - 2900	NMI	38	0	0	0	0	0	0	0	0	0	0	0	0	0	0	0	0	0	0	0	0	0	0	0	0	0	0	0
Port Ariane	LtPA-6c	C	RURAL	BA	2900 - 2800	NMI	27153	0	0	0	0	0	0	0	0	0	0	0	0	0	0	0	0	0	0	0	0	0	0	0	0	0	0	0
Port Ariane	LtPA-7	U	RURAL	IA1	2700 - 2650	NMI	613	0	0	0	0	0	0	0	0	0	0	0	0	0	0	0	0	0	0	0	0	0	0	6	0	0	0	0
Port Ariane	LtPA-7	C	RURAL	IA1	2700 - 2650	NMI	649	0	0	0	0	0	0	0	0	0	0	0	0	0	0	0	0	0	0	0	0	0	0	0	0	0	0	0
Port Ariane	LtPA-9	U	RURAL	IA2	2350 - 2300	NMI	15	0	0	0	0	0	0	0	0	0	0	0	0	0	0	0	0	0	0	0	0	0	0	0	0	0	0	0
Port Ariane	LtPA-9	C	RURAL	IA2	2350 - 2300	NMI	138	0	0	0	0	0	0	0	0	0	0	0	0	0	0	0	0	0	0	0	0	0	0	0	0	0	0	0
Portal Vielh	PVV-6a	C	RURAL	BA	3200 - 2900	NMI	299	0	0	0	0	0	0	0	0	0	0	0	0	0	0	0	0	0	0	0	0	0	0	0	0	0	0	0
Portal Vielh	PVV-6b	C	RURAL	BA	2900 - 2750	NMI	4366	0	0	0	0	0	0	0	0	0	0	0	0	0	0	0	0	0	1	0	0	0	0	0	0	0	0	0
Pré d'Ancy/Charnage	CLPAC-4	C	RURAL	BA	4200 - 3650	NMI	16	0	0	0	0	0	0	0	0	0	0	0	0	0	0	0	0	0	0	0	0	0	0	0	0	0	0	0
Pré d'Ancy/Charnage	CLPAC-5	C	RURAL	BA	3650 - 3250	NMI	49	0	0	0	0	0	0	0	0	0	0	0	0	0	0	0	0	0	0	0	0	0	0	0	0	0	0	0
Pré d'Ancy/Charnage	CLPAC-6	C	RURAL	BA	3300 - 2900	NMI	617	0	0	0	0	0	0	0	0	0	0	0	0	0	0	0	0	0	0	0	0	0	0	0	0	0	0	0
Pré de la Cour	PrdCo-6	C	RURAL	BA	2900 - 2750	NMI	12	0	0	0	0	0	0	0	0	0	0	0	0	0	0	0	0	0	1	0	0	0	0	0	0	0	0	0
Pré de la Cour	PrdCo-7	C	RURAL	IA1	2800 - 2700	NMI	298	0	0	0	0	0	0	0	0	0	0	0	0	0	0	0	0	0	0	0	0	0	0	0	0	0	0	0
Pré-aux-Pêcheurs	PaPe-10	U	URBAN	ERo	2083 - 1930	NMI	34	0	0	0	0	0	0	0	0	0	0	0	0	0	0	0	0	0	0	16	0	0	0	0	0	0	0	0
Pré-aux-Pêcheurs	PaPe-11	C	URBAN	LRo	1850 - 1600	NMI	25	0	0	0	0	0	0	0	0	0	0	0	0	0	0	0	0	0	0	1	0	0	0	2	0	0	0	0
Pré-aux-Pêcheurs	PaPe-11	U	URBAN	LRo	1850 - 1530	NMI	10467	0	0	0	0	64	470	0	0	27	0	2	6	40	6	4	0	0	90	4261	0	2	0	281	75	478	0	3
Près Bas	LpPBa-11	C	RURAL	LRo	1700 - 1670	NMI	1178	0	0	0	0	0	0	0	0	0	0	0	0	0	0	0	0	0	0	0	0	0	0	7	0	0	0	0
Prével Supérieur	PrSup-6	C	CAVE	BA	3300 - 3200	P/A	8	0	0	0	0	0	0	0	0	0	0	0	0	0	0	0	0	0	0	0	0	0	0	0	0	0	0	0
Pt Beaulieu/Puy Lg - funerary	PtBPLfu-4	C	FUN	BA	3900 - 3750	NMI	2	0	0	0	0	0	0	0	0	0	0	0	0	0	0	0	0	0	0	0	0	0	0	0	0	0	0	0
Puech de Mus	PdMus-7	C	VILLAGE	IA1	2700 - 2600	NMI	11	0	0	0	0	0	0	0	0	0	0	0	0	0	0	0	0	0	0	0	0	0	0	0	0	0	0	0
Puech de Mus	PdMus-8	C	VILLAGE	IA1/2	2600 - 2500	NMI	2174	0	0	0	0	0	0	0	0	0	0	0	0	0	0	0	0	0	4	0	0	0	0	0	0	0	0	0
Puech de Mus	PdMus-9	C	VILLAGE	IA2	2400 - 2300	NMI	829	0	0	0	0	0	0	0	0	0	0	0	0	0	0	0	0	0	0	1	0	0	0	0	0	0	0	0
Puech Haut	PaPH-3	C	RURAL	Neo	5300 - 4300	NMI	370	0	0	0	0	0	0	0	0	0	0	0	0	0	0	0	0	0	1	0	0	0	0	0	0	0	0	0
Puig del Baja	PuBaj-10	C	RURAL	ERo	2100 - 1850	NMI	120	0	0	0	0	0	1	0	0	0	0	0	1	0	0	0	0	0	1	0	0	0	0	0	2	0	0	1
Pujals 4	OrPuj-9	C	RURAL	IA2	2200 - 2100	NMI	1	0	0	0	0	0	0	0	0	0	0	0	0	0	0	0	0	0	0	0	0	0	0	0	0	0	0	0
Quai Arloing	LyQAr-10	C	FUN	ERo	2000 - 1875	NMI	1	0	0	0	0	0	0	0	0	0	0	0	0	0	0	0	1	0	0	0	0	0	0	0	0	0	0	0
Quinquiris	Quinq-3	C	RURAL	Neo	4830 - 4480	NMI	2	0	0	0	0	0	0	0	0	0	0	0	0	0	0	0	0	0	0	0	0	0	0	0	0	0	0	0
Quinquiris	Quinq-5	C	FUN	BA	3650 - 3350	NMI	2	0	0	0	0	0	0	0	0	0	0	0	0	0	0	0	0	0	1	0	0	0	0	0	0	0	0	0
Quinquiris	Quinq-7	C	RURAL	IA1	2525 - 2475	NMI	9	0	0	0	0	0	0	0	0	0	0	0	0	0	0	0	0	0	1	0	0	0	0	0	0	0	0	0
Rajal del Gorp	RajdG-9	U	CAVE	IA2	2200 - 2025	NMI	4	0	0	0	0	0	0	0	0	0	0	0	0	0	0	0	0	0	0	0	1	0	0	0	0	0	0	0
Rajal del Gorp	RajdG-9	C	CAVE	IA2	2200 - 2000	NMI	17	0	0	0	0	0	0	0	0	0	0	0	0	0	0	0	0	0	0	0	0	0	0	0	0	0	0	0
Rajal del Gorp	RajdG-10	C	FUN	ERo	2000 - 1900	NMI	18	0	0	0	0	0	0	0	0	0	0	0	0	0	0	0	0	0	0	0	0	0	0	0	0	0	0	0
Rajal del Gorp	RajdG-10	U	FUN	ERo	2000 - 1900	NMI	73	0	0	0	0	0	0	0	0	0	0	0	0	0	0	0	0	0	5	1	24	0	0	0	0	1	0	0
Ravaner 1 (Le)	Rav1-6	C	RURAL	BA	2900 - 2750	NMI	431	0	0	0	0	0	0	0	0	0	0	0	0	0	0	0	0	0	0	0	0	0	0	0	0	0	0	0
RD20e	RD20-10	U	RURAL	ERo	2000 - 1750	NMI	343	0	0	0	0	0	1	0	0	0	0	0	0	0	0	0	0	0	0	314	0	0	0	0	0	0	0	0
Rec de Ligno	VRcLi-10	U	RURAL	ERo	2025 - 1750	P/A	4	0	0	0	0	0	1	0	0	0	0	0	0	0	0	0	0	0	0	0	0	0	0	1	1	0	0	0
Rec de Ligno	VRcLi-10a	C	FUN	ERo	1950 - 1950	NMI	5	0	0	0	0	0	0	0	0	0	0	0	0	0	0	0	0	0	0	0	0	0	0	0	5	0	0	0
Rec de Ligno	VRcLi-10b	C	RURAL	ERo	1925 - 1900	NMI	9	0	0	0	0	0	0	0	0	0	0	0	0	0	0	0	0	0	0	0	0	0	0	5	0	0	0	0
Reille (La)	Reill-10	U	RURAL	ERo	1950 - 1575	NMI	53	0	0	0	0	0	2	0	0	0	0	0	0	0	0	0	0	0	0	1	0	0	0	1	0	1	0	1
Renaudi	MSRen-10	C	RURAL	ERo	2000 - 1900	NMI	296	0	0	0	0	0	1	0	0	0	0	0	0	0	0	0	0	0	3	0	0	0	0	0	0	0	0	0
Renaussas	VRena-10	C	FUN	ERo	2000 - 1700	NMI	11	0	0	0	0	0	6	0	0	0	0	0	0	0	0	0	0	0	0	0	0	0	0	0	0	0	0	0
Resclauza (La)	Resclz-1	C	RURAL	Neo	7000 - 6500	NMI	57	**0**	0	0	0	0	0	**0**	0	0	0	0	0	0	0	**0**	0	**0**	**0**	0	0	0	**0**	0	0	0	**0**	0
Revaute (La)	Revaut-7	C	RURAL	IA1	2650 - 2580	NMI	11	0	0	0	0	0	0	0	0	0	0	0	0	0	0	0	0	0	0	0	0	0	0	0	0	0	0	0
Riaux (Les)	ToRiax-10a	U	URBAN	ERo	1950 - 1900	NMI	276	0	0	0	0	0	4	0	0	0	0	0	0	0	0	0	0	0	0	253	0	0	0	3	0	0	1	0
Riaux (Les)	ToRiax-10b	C	URBAN	ERo	1950 - 1800	NMI	3	0	0	0	0	0	0	0	0	0	0	0	0	0	0	0	0	0	0	1	0	0	0	0	0	0	0	0
Riaux (Les)	ToRiax-10b	U	URBAN	ERo	1900 - 1800	NMI	607	0	0	0	0	5	12	0	0	14	0	0	0	0	0	0	0	0	1	491	0	1	0	2	4	0	0	0
Riaux (Les)	ToRiax-10c	C	URBAN	ERo	1825 - 1700	NMI	1	0	0	0	0	0	0	0	0	0	0	0	0	0	0	0	0	0	0	0	0	0	0	0	0	0	0	0
Riaux (Les)	ToRiax-10c	U	URBAN	ERo	1825 - 1700	NMI	74	0	0	0	0	1	4	0	0	0	0	0	0	0	0	0	0	0	0	35	0	0	0	1	1	0	0	0
Rivaux (Les)	Rivox-2	C	RURAL	Neo	6500 - 5500	NMI	1272	0	0	0	0	0	0	0	0	0	0	0	0	0	0	0	0	0	388	0	0	0	0	0	0	0	0	0
Ro di Bolo	RdBo-3	C	CAVE	Neo	5500 - 4200	NMI	18	0	0	0	0	0	0	0	0	0	0	0	0	0	0	0	0	0	0	0	0	0	0	0	0	0	0	0
Roberte (La)	LaRob-2	C	RURAL	Neo	6000 - 5500	NMI	225	0	0	0	0	0	0	0	0	0	0	0	0	0	0	0	0	0	0	0	0	0	0	0	0	0	0	0
Roc Troué (Abri)	RcTro-1	C	CAVE	Neo	7000 - 6500	NMI	3	0	0	0	0	0	0	0	0	0	0	0	0	0	0	0	0	0	1	0	0	0	0	0	0	0	0	0
Rochas	Rocha-3	C	RURAL	Neo	4800 - 4200	P/A	3	0	0	0	0	0	0	0	0	0	0	0	0	0	0	0	0	0	0	0	0	0	0	0	0	0	0	0
Roche Noire	RoNo-7	C	CAVE	IA1	2600 - 2525	NMI	12248	0	0	0	0	0	0	0	0	0	0	0	0	0	0	0	0	0	2	0	0	0	0	0	0	0	0	0
Roque (La)	LaRoq-9	C	RURAL	IA2	2125 - 2075	NMI	3	0	0	0	0	0	0	0	0	0	0	0	0	0	0	0	0	0	0	0	0	0	0	0	0	0	0	0
Roque (La)	LaRoq-9	U	VILLAGE	IA2	2125 - 2075	NMI	866	0	0	0	0	0	0	0	0	0	0	0	0	0	0	0	0	0	0	589	0	20	0	0	0	0	0	4
Roquemengarde	RQMG-3	C	RURAL	Neo	5450 - 4550	NMI	9789	0	0	0	0	0	0	0	0	0	0	0	0	0	0	0	0	0	0	0	0	0	0	0	0	0	0	0
Roquepertuse	RQP-9a	C	VILLAGE	IA2	2450 - 2400	NMI	1336	0	0	0	0	0	0	0	0	0	0	0	0	0	0	0	0	0	1	0	0	0	0	0	0	0	0	0
Roquepertuse	RQP-9b	C	VILLAGE	IA2	2225 - 2175	NMI	2953	0	0	0	0	0	0	0	0	0	0	0	0	0	0	0	0	0	0	0	0	0	0	0	0	0	0	0
Roquessols	VRoq-3	C	RURAL	Neo	5500 - 4200	NMI	366	0	0	0	0	0	0	0	0	0	0	0	0	0	0	0	0	0	0	0	0	0	0	0	0	0	0	0

Site	Cod Sit-Phas	Pres	type	Period	DATE BP	Prsp	Pyru	Viti	Vitipd	Arbu	Corma	Corsa	Crata	Junsp	Myrt	Physa	Pincem	Pinsp	Pista	Prspi	Querc	Rhus	Rosa	Rubca	Rubfr	Rubid	Rubsp	Sambeb	Sambni	Sambsp	Sorb	Vibu	An crops
Port Ariane	LtPA-6a	U	RURAL	BA	3400 - 3200	0	0	6	0	0	0	0	0	0	0	0	0	0	0	0	0	0	0	0	0	0	0	3	0	0	0	0	0
Port Ariane	LtPA-6a	C	RURAL	BA	3400 - 3200	0	0	0	0	0	0	0	0	0	0	0	0	0	0	0	0	0	0	0	0	0	0	0	0	0	0	0	1
Port Ariane	LtPA-6b	C	RURAL	BA	3200 - 3000	0	0	1	0	0	2	0	0	0	0	0	0	0	0	0	0	0	0	0	0	0	0	0	0	0	0	0	36
Port Ariane	LtPA-6b	U	RURAL	BA	3200 - 2900	0	0	13	0	0	0	0	0	0	0	0	0	0	0	0	0	0	0	0	0	0	0	25	0	0	0	0	0
Port Ariane	LtPA-6c	C	RURAL	BA	2900 - 2800	0	0	1	0	0	0	0	0	0	0	0	0	0	0	0	0	0	0	0	0	0	0	1	0	0	0	0	27151
Port Ariane	LtPA-7	U	RURAL	IA1	2700 - 2650	0	0	109	0	0	0	58	0	0	0	0	0	0	0	32	0	0	0	0	311	0	0	64	33	0	0	0	0
Port Ariane	LtPA-7	C	RURAL	IA1	2700 - 2650	0	0	7	1	0	0	0	0	0	0	0	0	0	0	0	0	0	0	0	0	0	0	3	0	0	0	0	638
Port Ariane	LtPA-9	U	RURAL	IA2	2350 - 2300	0	0	10	1	0	0	0	0	0	0	0	0	0	0	0	0	0	0	0	2	0	0	2	0	0	0	0	0
Port Ariane	LtPA-9	C	RURAL	IA2	2350 - 2300	0	0	11	2	0	0	0	0	0	0	0	0	0	0	0	0	0	0	0	0	0	0	0	0	0	0	0	125
Portal Vielh	PVV-6a	C	RURAL	BA	3200 - 2900	1	0	3	0	0	0	0	0	0	0	0	0	0	0	0	0	0	0	0	0	0	0	2	0	0	0	0	293
Portal Vielh	PVV-6b	C	RURAL	BA	2900 - 2750	0	0	5	0	0	0	0	0	0	0	0	0	0	0	0	832	0	0	0	0	0	0	0	0	0	0	0	3528
Pré d'Ancy/Charnage	CLPAC-4	C	RURAL	BA	4200 - 3650	0	0	0	0	0	0	0	0	0	0	0	0	0	0	0	9	0	0	0	0	0	0	0	0	0	0	0	7
Pré d'Ancy/Charnage	CLPAC-5	C	RURAL	BA	3650 - 3250	0	0	0	0	0	0	0	0	0	0	0	0	0	0	0	0	0	0	0	0	0	0	0	0	0	0	0	49
Pré d'Ancy/Charnage	CLPAC-6	C	RURAL	BA	3300 - 2900	0	0	0	0	0	0	0	0	0	0	0	0	0	0	2	0	0	0	0	1	0	0	1	2	0	0	0	611
Pré de la Cour	PrdCo-6	C	RURAL	BA	2900 - 2750	0	0	0	0	0	0	0	0	0	0	0	0	0	0	0	0	0	0	0	0	0	0	0	0	0	0	0	11
Pré de la Cour	PrdCo-7	C	RURAL	IA1	2800 - 2700	0	0	0	0	0	0	0	0	0	0	0	0	0	0	0	0	0	0	0	0	0	0	0	0	0	0	0	298
Pré-aux-Pêcheurs	PaPe-10	U	URBAN	ERo	2083 - 1930	0	0	0	0	0	0	0	0	1	0	0	0	0	0	0	0	0	0	0	15	0	0	0	2	0	0	0	0
Pré-aux-Pêcheurs	PaPe-11	C	URBAN	LRo	1850 - 1600	2	0	3	0	0	0	0	0	0	0	0	0	0	0	0	0	0	0	0	0	0	0	0	0	0	0	0	17
Pré-aux-Pêcheurs	PaPe-11	U	URBAN	LRo	1850 - 1530	0	0	4230	114	0	2	28	0	18	0	0	0	0	15	6	30	0	0	6	140	0	0	7	37	0	0	0	25
Près Bas	LpPBa-11	C	RURAL	LRo	1700 - 1670	0	0	218	0	0	0	0	0	0	0	0	0	0	0	0	0	0	0	0	0	0	0	0	0	0	0	0	953
Prével Supérieur	PrSup-6	C	CAVE	BA	3300 - 3200	0	0	0	0	1	0	0	0	0	0	0	0	0	0	0	0	0	0	0	0	0	0	0	0	0	0	0	7
Pt Beaulieu/Puy Lg - funerary	PtBPLfu-4	C	FUN	BA	3900 - 3750	0	0	0	0	0	0	0	0	0	0	0	0	0	0	0	0	0	0	0	0	0	0	0	0	0	0	0	2
Puech de Mus	PdMus-7	C	VILLAGE	IA1	2700 - 2600	0	0	0	0	0	0	0	0	0	0	0	0	0	0	0	4	0	0	0	0	0	0	0	1	0	0	0	6
Puech de Mus	PdMus-8	C	VILLAGE	IA1/2	2600 - 2500	0	0	0	0	0	0	0	0	0	0	0	0	0	0	2	23	0	0	0	1	0	0	0	0	0	0	0	2144
Puech de Mus	PdMus-9	C	VILLAGE	IA2	2400 - 2300	0	0	0	0	0	0	0	0	0	0	0	0	0	0	0	18	0	0	0	0	0	0	0	0	0	0	0	810
Puech Haut	PaPH-3	C	RURAL	Neo	5300 - 4300	0	0	2	0	0	0	0	0	0	0	0	0	0	0	0	10	0	0	0	0	0	0	0	0	0	0	0	357
Puig del Baja	PuBaj-10	C	RURAL	ERo	2100 - 1850	0	0	4	0	0	0	0	0	0	0	0	0	0	0	0	1	0	0	0	0	0	0	0	0	0	0	0	109
Pujals 4	OrPuj-9	C	RURAL	IA2	2200 - 2100	0	0	1	0	0	0	0	0	0	0	0	0	0	0	0	0	0	0	0	0	0	0	0	0	0	0	0	0
Quai Arloing	LyQAr-10	C	FUN	ERo	2000 - 1875	0	0	0	0	0	0	0	0	0	0	0	0	0	0	0	0	0	0	0	0	0	0	0	0	0	0	0	0
Quinquiris	Quinq-3	C	RURAL	Neo	4830 - 4480	0	0	0	0	0	0	0	0	0	0	0	0	0	0	0	0	0	0	0	0	0	0	1	0	0	0	0	1
Quinquiris	Quinq-5	C	FUN	BA	3650 - 3350	0	0	0	0	0	0	0	0	0	0	0	0	0	0	0	0	0	0	0	0	0	0	0	0	0	0	0	1
Quinquiris	Quinq-7	C	RURAL	IA1	2525 - 2475	0	0	0	0	0	0	0	0	0	0	0	0	0	0	0	0	0	0	0	0	0	0	0	0	0	0	0	8
Rajal del Gorp	RajdG-9	U	CAVE	IA2	2200 - 2025	0	0	0	0	0	0	0	0	0	0	0	0	0	0	0	0	0	0	0	1	0	0	0	0	1	0	0	1
Rajal del Gorp	RajdG-9	C	CAVE	IA2	2200 - 2000	1	0	0	0	0	0	0	0	0	0	0	0	0	0	0	2	0	0	0	0	0	0	0	0	0	0	0	14
Rajal del Gorp	RajdG-10	C	FUN	ERo	2000 - 1900	0	0	0	0	0	0	0	0	0	0	0	0	0	0	0	0	0	0	0	0	0	0	0	0	0	0	0	18
Rajal del Gorp	RajdG-10	U	FUN	ERo	2000 - 1900	0	0	4	0	0	0	0	0	0	0	0	0	0	0	1	0	0	0	0	26	10	0	0	1	0	0	0	0
Ravaner 1 (Le)	Rav1-6	C	RURAL	BA	2900 - 2750	0	0	0	0	0	0	0	0	0	0	0	0	0	0	0	3	0	0	0	0	0	0	0	0	0	0	0	428
RD20e	RD20-10	U	RURAL	ERo	2000 - 1750	0	0	17	1	0	0	0	0	1	0	0	0	0	0	0	0	0	0	0	9	0	0	0	0	0	0	0	0
Rec de Ligno	VRcLi-10	U	RURAL	ERo	2025 - 1750	0	0	1	0	0	0	0	0	0	0	0	0	0	0	0	0	0	0	0	0	0	0	0	0	0	0	0	0
Rec de Ligno	VRcLi-10a	C	FUN	ERo	1950 - 1950	0	0	0	0	0	0	0	0	0	0	0	0	0	0	0	0	0	0	0	0	0	0	0	0	0	0	0	0
Rec de Ligno	VRcLi-10b	C	RURAL	ERo	1925 - 1900	0	0	3	0	0	0	0	0	0	0	0	0	0	0	0	0	0	0	0	0	0	0	0	0	0	0	0	1
Reille (La)	Reill-10	U	RURAL	ERo	1950 - 1575	0	0	20	8	0	0	0	0	0	0	0	0	0	0	0	0	0	0	0	17	0	0	0	0	0	0	0	2
Renaudi	MSRen-10	C	RURAL	ERo	2000 - 1900	0	0	0	0	0	0	0	0	0	0	0	0	0	0	0	0	0	0	0	0	0	0	0	0	0	0	0	292
Renaussas	VRena-10	C	FUN	ERo	2000 - 1700	0	0	3	0	0	0	0	0	0	0	0	0	0	0	0	0	0	0	0	0	0	0	0	0	0	0	0	2
Resclauza (La)	Resclz-1	C	RURAL	Neo	7000 - 6500	0	0	**0**	0	**0**	0	**0**	0	**0**	0	0	0	**0**	**0**	**0**	**1**	**0**	**0**	0	**0**	0	**0**	**0**	**0**	**0**	**0**	**0**	56
Revaute (La)	Revaut-7	C	RURAL	IA1	2650 - 2580	0	0	0	0	0	0	0	0	0	0	0	0	0	0	0	0	0	0	0	0	0	0	0	0	0	0	0	11
Riaux (Les)	ToRiax-10a	U	URBAN	ERo	1950 - 1900	0	0	11	2	0	0	0	0	0	0	0	0	0	0	0	0	0	0	0	0	0	0	0	0	1	0	0	1
Riaux (Les)	ToRiax-10b	C	URBAN	ERo	1950 - 1800	0	0	0	0	0	0	0	0	0	0	0	0	0	0	0	0	0	0	0	0	0	0	0	0	0	0	0	2
Riaux (Les)	ToRiax-10b	U	URBAN	ERo	1900 - 1800	0	0	60	5	0	0	0	0	0	0	0	0	0	0	0	2	0	0	0	6	0	0	3	0	0	0	0	1
Riaux (Les)	ToRiax-10c	C	URBAN	ERo	1825 - 1700	0	0	0	0	0	0	0	0	0	0	0	0	0	0	0	0	0	0	0	0	0	0	0	0	0	0	0	1
Riaux (Les)	ToRiax-10c	U	URBAN	ERo	1825 - 1700	0	0	26	4	0	0	0	0	0	0	0	0	0	0	0	0	0	0	0	1	0	0	0	0	0	0	0	1
Rivaux (Les)	Rivox-2	C	RURAL	Neo	6500 - 5500	0	0	0	0	0	0	0	0	0	0	0	0	0	0	0	0	0	0	0	0	0	0	0	0	0	0	0	884
Ro di Bolo	RdBo-3	C	CAVE	Neo	5500 - 4200	0	0	0	0	0	0	0	0	0	0	0	0	0	0	0	0	0	0	0	0	0	0	0	0	0	0	0	18
Roberte (La)	LaRob-2	C	RURAL	Neo	6000 - 5500	0	0	0	0	0	0	0	0	0	0	0	0	0	0	0	0	0	0	0	0	0	0	0	0	0	0	0	225
Roc Troué (Abri)	RcTro-1	C	CAVE	Neo	7000 - 6500	0	0	0	0	0	0	0	0	0	0	0	0	0	0	0	0	0	0	0	0	0	0	0	0	0	0	0	2
Rochas	Rocha-3	C	RURAL	Neo	4800 - 4200	0	0	0	0	0	0	0	0	0	0	0	0	0	0	0	3	0	0	0	0	0	0	0	0	0	0	0	0
Roche Noire	RoNo-7	C	CAVE	IA1	2600 - 2525	0	0	0	0	0	0	1	0	0	0	0	0	0	0	1	1	0	0	0	0	0	0	0	97	0	0	0	12146
Roque (La)	LaRoq-9	C	RURAL	IA2	2125 - 2075	0	0	0	0	0	0	0	0	0	0	0	0	0	0	0	0	0	0	0	0	0	0	0	0	0	0	0	3
Roque (La)	LaRoq-9	U	VILLAGE	IA2	2125 - 2075	0	0	104	1	0	0	0	0	1	0	0	0	0	0	0	0	0	0	6	79	0	0	44	0	0	0	0	18
Roquemengarde	RQMG-3	C	RURAL	Neo	5450 - 4550	0	0	5	0	0	0	0	0	0	0	0	0	0	0	1	0	0	0	0	0	0	0	0	0	0	0	0	9783
Roquepertuse	RQP-9a	C	VILLAGE	IA2	2450 - 2400	0	0	15	0	0	0	0	0	0	0	0	0	0	0	0	0	0	0	0	0	0	0	0	0	0	0	0	1320
Roquepertuse	RQP-9b	C	VILLAGE	IA2	2225 - 2175	0	0	12	1	0	0	0	0	0	0	0	0	0	0	0	0	0	0	0	0	0	0	0	0	0	0	0	2940
Roquessols	VRoq-3	C	RURAL	Neo	5500 - 4200	1	0	0	0	0	0	0	0	0	0	0	0	0	0	0	0	0	0	0	0	0	0	0	0	0	0	0	365

Site	Cod Sit-Phas	Pres	type	Period	DATE BP	Quantif	MNI	Citrul	Cumel	Cupre	Cusat	Cusp	Jugla	Lage	Mespi	Moru	Prcer	Prdul	Prpers	Punic	Zizip	Cordi	Phoen	Celti	Coryl	Ficu	Fraga	Maloid	Malu	Olea	Pinpi	Pravce	Pravi	Prdom
Roquette (La)	Roqt-11	C	RURAL	LRo	1625 - 1550	NMI	8	0	0	0	0	0	1	0	0	0	0	0	0	0	0	0	0	0	0	1	0	0	0	0	0	0	0	0
Roquette (La)	Roqt-11	U	RURAL	LRo	1625 - 1550	NMI	2116	0	10	1	0	0	672	0	1	0	0	2	8	0	0	0	0	7	32	78	1	0	14	20	1	0	41	8
Rouget (Le)	MoRou-7	C	RURAL	IA1	2700 - 2600	NMI	878	0	0	0	0	0	0	0	0	0	0	0	0	0	0	0	0	0	0	0	0	0	0	0	0	0	0	0
Roumèges	Roumg-10	U	RURAL	ERo	1970 - 1875	NMI	1243	0	0	0	0	0	5	0	0	0	0	1	0	0	0	0	0	0	0	105	0	0	0	22	0	0	0	13
Rue de la Basse	PrRBa-11	C	RURAL	LRo	1750 - 1500	NMI	3063	0	0	0	0	0	1	0	0	0	0	0	0	0	0	0	0	0	1	0	0	0	0	2	29	0	0	0
Rue de la Corderie	SRRco-Ro	C	URBAN	Rom	2025 - 1500	NMI	26	0	0	0	0	0	5	0	0	0	0	0	0	0	0	0	0	0	0	0	0	0	0	0	0	1	0	0
Rue du Partegal	Prtgl-10	U	RURAL	ERo	1950 - 1900	NMI	607	0	0	0	0	0	1	0	0	0	0	0	0	0	0	0	0	0	0	304	0	0	0	4	0	0	0	0
Sables (Les)	ThSab-7	C	RURAL	IA1	2750 - 2625	NMI	100	0	0	0	0	0	0	0	0	0	0	0	0	0	0	0	0	0	0	0	0	0	0	0	0	0	0	0
Saint Aunès (ZAC)	ZACSA-2	C	RURAL	Neo	6500 - 5500	NMI	546	0	0	0	0	0	0	0	0	0	0	0	0	0	0	0	0	0	0	0	0	0	0	0	0	0	0	0
Saint Bézard	StBez-10	C	RURAL	ERo	1925 - 1875	NMI	36	0	0	0	0	0	0	0	0	0	0	0	0	0	0	0	0	0	0	0	0	0	0	5	0	0	0	0
Saint Etienne (Abri)	AbStE-6	C	CAVE	BA	3200 - 2900	NMI	18	0	0	0	0	0	0	0	0	0	0	0	0	0	0	0	0	0	0	0	0	0	0	0	0	0	0	0
Saint Etienne de Dions	StEDi-8	C	VILLAGE	IA1/2	2525 - 2475	NMI	4	0	0	0	0	0	0	0	0	0	0	0	0	0	0	0	0	0	0	0	0	0	0	0	0	0	0	0
Saint Etienne de Dions	StEDi-9	C	VILLAGE	IA2	2325 - 2275	NMI	342	0	0	0	0	0	0	0	0	0	0	0	0	0	0	0	0	0	0	0	0	0	0	0	0	0	0	0
Saint Lambert	FrSLam-10	C	FUN	ERo	1950 - 1800	NMI	25	0	0	0	0	0	0	0	0	0	0	0	0	0	0	0	6	0	3	7	0	0	1	0	8	0	0	0
Saint Marcel (Grotte)	GtSMa-1	C	CAVE	Neo	7500 - 7000	NMI	5	0	0	0	0	0	0	0	0	0	0	0	0	0	0	0	0	0	0	0	0	0	0	0	0	0	0	0
Saint Marcel (Grotte)	GtSMa-2	C	CAVE	Neo	6000 - 5500	NMI	24	0	0	0	0	0	0	0	0	0	0	0	0	0	0	0	0	0	0	0	0	0	0	0	0	0	0	0
Saint Marcel (Grotte)	GtSMa-3	C	CAVE	Neo	5400 - 4800	NMI	508	0	0	0	0	0	0	0	0	0	0	0	0	0	0	0	0	0	0	0	0	0	0	0	0	0	0	0
Saint Marcel (Grotte)	GtSMa-6	C	CAVE	BA	3300 - 3050	NMI	21	0	0	0	0	0	0	0	0	0	0	0	0	0	0	0	0	0	0	0	0	0	0	0	0	0	0	0
Saint Marcel (Oppidum)	OpStM-8	C	VILLAGE	IA1/2	2500 - 2480	P/A	20	0	0	0	0	0	0	0	0	0	0	0	0	0	0	0	0	0	0	0	0	0	0	0	0	0	0	0
Saint Marcel (Oppidum)	OpStM-9	C	VILLAGE	IA2	2400 - 2375	P/A	21	0	0	0	0	0	0	0	0	0	0	0	0	0	0	0	0	0	0	0	0	0	0	0	0	0	0	0
Saint Martin d'Azirou	SMAz-3	C	FUN	Neo	5500 - 4600	NMI	387	0	0	0	0	0	0	0	0	0	0	0	0	0	0	0	0	0	1	0	0	0	0	0	0	0	0	0
Saint Martin III	SMT3-4	C	RURAL	BA	4200 - 3650	NMI	12	0	0	0	0	0	0	0	0	0	0	0	0	0	0	0	0	0	2	0	0	0	0	0	0	0	0	0
Saint Pierre	StPie-8	C	VILLAGE	IA1/2	2550 - 2475	P/A	2	0	0	0	0	0	0	0	0	0	0	0	0	0	0	0	0	0	0	0	0	0	0	0	0	0	0	0
Saint Pierre	StPie-10	C	VILLAGE	ERo	2020 - 1800	P/A	1	0	0	0	0	0	0	0	0	0	0	0	0	0	0	0	0	0	0	0	0	0	0	1	0	0	0	0
Saint Romain de Jalionas	SRJ-10	C	RURAL	ERo	2000 - 1770	NMI	3	0	0	0	0	0	0	0	0	0	0	0	0	0	0	0	0	0	1	0	0	0	0	0	0	0	0	0
Saint Romain de Jalionas	SRJ-11	C	RURAL	LRo	1800 - 1600	NMI	6419	0	0	0	0	0	1	0	0	0	0	0	0	0	0	0	0	0	0	0	0	0	0	0	0	0	0	0
Saint Romain de Jalionas -T57	SRJ-10	U	OFFSITE	ERo	2000 - 1770	NMI	4446	0	0	0	0	0	13	2	7	0	0	0	2	0	0	0	0	0	5	1190	124	0	0	0	0	0	62	19
Salle (La)	LaSal-2	C	RURAL	Neo	6000 - 5500	NMI	292	0	0	0	0	0	0	0	0	0	0	0	0	0	0	0	0	0	0	0	0	0	0	0	0	0	0	0
Salpétrière (grotte de)	GtSal-3	C	CAVE	Neo	4700 - 4200	NMI	9	0	0	0	0	0	0	0	0	0	0	0	0	0	0	0	0	0	0	0	0	0	0	0	0	0	0	0
Sant Jaume del Crest	CerSJC-10	C	RURAL	ERo	1925 - 1850	NMI	1	0	0	0	0	0	0	0	0	0	0	0	0	0	0	0	0	0	0	0	0	0	0	0	0	0	0	0
Sargel (Grotte)	GtSag-2	C	CAVE	Neo	6500 - 5500	P/A	2	0	0	0	0	0	0	0	0	0	0	0	0	0	0	0	0	0	1	0	0	0	0	0	0	0	0	0
Sargel (Grotte)	GtSag-3	C	CAVE	Neo	4800 - 4200	NMI	303	0	0	0	0	0	0	0	0	0	0	0	0	0	0	0	0	0	1	0	0	0	1	0	0	0	0	0
Sauzet/A7-Section 2	SauzA7-10	C	RURAL	ERo	1925 - 1900	NMI	113	0	0	0	0	0	0	0	0	0	0	0	0	0	0	0	0	0	0	0	0	0	0	0	0	0	0	0
Serre 1 (Le)	RS1-1	C	RURAL	Neo	7000 - 6500	NMI	1145	0	0	0	0	0	0	0	0	0	0	0	0	0	0	0	0	0	0	0	0	0	0	0	0	0	0	0
Serre 1 (Le)	RS1-4	C	RURAL	BA	4200 - 3900	NMI	1895	0	0	0	0	0	0	0	0	0	0	0	0	0	0	0	0	0	0	0	0	0	0	0	0	0	0	0
Serre de Boidon	SedBo-3	C	RURAL	Neo	4800 - 4200	NMI	4428	0	0	0	0	0	0	0	0	0	0	0	0	0	0	0	0	0	2	0	0	0	0	0	0	0	0	0
Sizen-Vigne	SizVig-9	C	FUN	IA2	2400 - 2200	NMI	43	0	0	0	0	0	0	0	0	0	0	0	0	0	0	0	0	0	0	0	0	0	0	0	0	0	0	0
Soumaltre	AsSou-10	C	RURAL	ERo	2000 - 1800	NMI	1156	0	0	0	0	0	0	0	0	0	0	0	0	0	0	0	0	0	0	0	0	0	0	0	0	0	0	0
Soumaltre - funerary	AsSouF-10	C	FUN	ERo	2000 - 1800	NMI	154	0	0	3	0	0	0	0	0	0	0	0	0	0	0	0	0	0	0	25	0	0	0	0	0	0	0	0
Taï (Le)	RTai-1	C	CAVE	Neo	7400 - 7000	NMI	1060	0	0	0	0	0	0	0	0	0	0	0	0	0	0	0	0	0	2	0	0	0	0	0	0	0	0	0
Taï (Le)	RTai-2	C	CAVE	Neo	6500 - 5500	NMI	83	0	0	0	0	0	0	0	0	0	0	0	0	0	0	0	0	0	0	0	0	0	0	0	0	0	0	0
Taï (Le)	RTai-3	C	CAVE	Neo	5500 - 4200	NMI	562	0	0	0	0	0	0	0	0	0	0	0	0	0	0	0	0	0	0	1	0	0	0	0	0	0	0	0
Terrasses Montfo	MagTM-11	U	FUN	LRo	1700 - 1600	NMI	1640	0	0	1	0	0	18	3	0	1	0	1	0	0	0	0	0	0	1	84	0	0	0	2	0	1	0	0
Terrofort 268	CaTer-11	C	RURAL	LRo	1630 - 1550	NMI	1	0	0	0	0	0	0	0	0	0	0	0	0	0	0	0	0	0	0	0	0	0	0	0	0	0	0	0
Teste Nègre	TsNeg-9	C	VILLAGE	IA2	2300 - 2100	NMI	1432	0	0	0	0	0	0	0	0	0	0	0	0	0	0	0	0	0	0	0	0	0	0	0	0	0	0	0
Théâtre d'Agglomération	FrThAg-10	U	OFFSITE	ERo	2030 - 1970	NMI	260	0	0	0	0	0	0	0	0	0	0	0	0	0	0	0	0	0	2	66	0	0	0	1	4	0	0	0
Théâtre de Villeneuve	FrThVi-10a	U	OFFSITE	ERo	2025 - 1950	NMI	7	0	0	0	0	0	0	0	0	0	0	0	0	0	0	0	0	0	0	0	0	0	0	0	0	0	0	7
Théâtre de Villeneuve	FrThVi-10b	C	OFFSITE	ERo	1900 - 1800	NMI	6	0	0	0	0	0	0	0	0	0	0	0	0	0	0	0	0	0	0	0	0	0	0	0	3	0	0	0
Théâtre de Villeneuve	FrThVi-10b	U	OFFSITE	ERo	1900 - 1800	NMI	478	0	0	0	0	0	16	0	0	2	0	0	0	0	0	0	0	0	0	16	0	0	0	0	0	0	0	0
Théâtre de Villeneuve	FrThVi-11a	U	OFFSITE	LRo	1800 - 1700	NMI	241	0	0	31	0	0	0	0	0	0	0	0	0	0	0	0	0	0	0	124	0	0	0	0	1	0	0	53
Théâtre de Villeneuve	FrThVi-11b	C	OFFSITE	LRo	1600 - 1500	NMI	1	0	0	0	0	0	0	0	0	0	0	0	0	0	0	0	0	0	0	0	0	0	0	0	1	0	0	0
Théâtre de Villeneuve	FrThVi-11b	U	OFFSITE	LRo	1600 - 1500	NMI	33	0	0	0	0	0	0	23	0	0	0	0	0	0	0	0	0	0	1	1	0	1	0	0	1	0	0	0
Touar (Le)	Touar-6	C	RURAL	BA	3250 - 2900	NMI	3	0	0	0	0	0	0	0	0	0	0	0	0	0	0	0	0	0	0	0	0	0	0	0	0	0	0	0
Touar (Le)	Touar-7	C	RURAL	IA1	2650 - 2500	NMI	204	0	0	0	0	0	0	0	0	0	0	0	0	0	0	0	0	0	0	0	0	0	0	0	0	0	0	0
Toulons (Les)	RiToul-10	C	RURAL	ERo	1850 - 1800	NMI	184	0	0	0	0	0	5	0	0	0	0	0	0	0	0	0	0	0	0	0	0	0	0	0	0	0	0	0
Toulons (Les)	RiToul-11a	C	RURAL	LRo	1750 - 1700	NMI	23	0	0	0	0	0	0	0	0	0	0	0	0	0	0	0	0	0	0	0	0	0	0	0	0	0	0	0
Toulons (Les)	RiToul-11b	C	RURAL	LRo	1650 - 1600	NMI	38	0	0	0	0	0	0	0	0	0	0	0	0	0	0	0	0	0	0	0	0	0	0	0	0	0	0	0
Tourteix (Le)	OrTxt-4	C	RURAL	BA	3600 - 3200	NMI	63	0	0	0	0	0	0	0	0	0	0	0	0	0	0	0	0	0	0	0	0	0	0	0	0	0	0	0
Tremaïe	Trema-9	C	URBAN	IA2	2125 - 2100	NMI	16	0	0	0	0	0	0	0	0	0	0	0	0	3	0	0	0	0	0	0	0	0	0	0	0	0	0	0
Tumbas (Les)	CerTu-10	C	RURAL	ERo	2100 - 1900	NMI	2	0	0	0	0	0	0	0	0	0	0	0	0	0	0	0	0	0	0	0	0	0	0	0	0	0	0	0
Vaise	LyVais-1	C	RURAL	Neo	7200 - 6500	NMI	396	0	0	0	0	0	0	0	0	0	0	0	0	0	0	0	0	0	0	0	0	0	0	0	0	0	0	0
Vaise	LyVais-2	C	RURAL	Neo	6500 - 5500	NMI	101	0	0	0	0	0	0	0	0	0	0	0	0	0	0	0	0	0	6	0	0	0	0	0	0	0	0	0
Vaise - ZAC Industrie Nord	LyZIN-10	C	FUN	ERo	2025 - 1750	NMI	3565	0	0	4	0	0	0	0	0	0	0	6	0	0	0	0	5	0	31	1	0	0	0	4	0	0	0	0

Site	Cod Sit-Phas	Pres	type	Period	DATE BP	Prsp	Pyru	Viti	Vitipd	Arbu	Corma	Corsa	Crata	Junsp	Myrt	Physa	Pincem	Pinsp	Pista	Prspi	Querc	Rhus	Rosa	Rubca	Rubfr	Rubid	Rubsp	Sambeb	Sambni	Sambsp	Sorb	Vibu	An crops
Roquette (La)	Roqt-11	C	RURAL	LRo	1625 - 1550	0	0	0	0	0	0	0	0	0	0	0	0	0	0	0	3	0	0	0	0	0	0	0	0	0	0	0	3
Roquette (La)	Roqt-11	U	RURAL	LRo	1625 - 1550	0	0	1054	0	0	1	2	2	0	0	6	0	0	0	12	78	0	8	3	9	0	0	2	0	0	0	0	42
Rouget (Le)	MoRou-7	C	RURAL	IA1	2700 - 2600	0	0	0	0	0	0	0	0	0	0	0	0	0	0	0	0	0	0	0	0	0	0	0	0	0	0	0	878
Roumèges	Roumg-10	U	RURAL	ERo	1970 - 1875	0	0	397	45	0	1	0	0	0	0	0	0	0	0	0	1	0	0	0	30	0	0	0	131	0	0	0	492
Rue de la Basse	PrRBa-11	C	RURAL	LRo	1750 - 1500	0	0	3	0	0	0	0	0	0	0	0	0	0	0	1	0	0	0	0	0	0	0	0	0	0	0	0	3026
Rue de la Corderie	SRRco-Ro	C	URBAN	Rom	2025 - 1500	0	0	3	0	0	0	0	0	0	0	0	0	0	0	0	1	0	0	0	0	0	0	0	1	0	0	0	15
Rue du Partegal	Prtgl-10	U	RURAL	ERo	1950 - 1900	0	0	253	21	0	0	0	0	0	0	0	0	0	0	0	0	0	0	0	0	0	0	0	0	0	0	0	24
Sables (Les)	ThSab-7	C	RURAL	IA1	2750 - 2625	0	0	0	0	0	0	0	0	0	0	0	0	0	0	0	0	0	0	0	0	0	0	0	0	0	0	0	100
Saint Aunès (ZAC)	ZACSA-2	C	RURAL	Neo	6500 - 5500	0	0	0	0	0	0	0	0	0	0	0	0	0	0	0	0	0	0	0	0	0	0	0	0	0	0	0	546
Saint Bézard	StBez-10	C	RURAL	ERo	1925 - 1875	0	0	8	0	0	0	0	0	0	0	0	0	0	0	0	2	0	0	0	0	0	0	0	0	0	0	0	21
Saint Etienne (Abri)	AbStE-6	C	CAVE	BA	3200 - 2900	0	0	0	0	0	0	0	0	0	0	0	0	0	0	0	0	0	0	0	0	0	0	0	0	0	0	0	18
Saint Etienne de Dions	StEDi-8	C	VILLAGE	IA1/2	2525 - 2475	0	0	0	0	0	0	0	0	0	0	0	0	0	0	0	0	0	0	0	0	0	0	0	0	0	0	0	4
Saint Etienne de Dions	StEDi-9	C	VILLAGE	IA2	2325 - 2275	0	0	0	0	0	0	0	0	0	0	0	0	0	0	0	0	0	0	0	0	0	0	0	0	0	0	0	342
Saint Lambert	FrSLam-10	C	FUN	ERo	1950 - 1800	0	0	0	0	0	0	0	0	0	0	0	0	0	0	0	0	0	0	0	0	0	0	0	0	0	0	0	0
Saint Marcel (Grotte)	GtSMa-1	C	CAVE	Neo	7500 - 7000	0	0	0	0	0	0	0	0	0	0	0	0	0	0	0	0	0	0	0	0	0	0	0	0	0	0	0	5
Saint Marcel (Grotte)	GtSMa-2	C	CAVE	Neo	6000 - 5500	0	0	0	0	1	0	0	0	0	0	0	0	0	0	0	0	0	0	0	0	0	0	0	0	0	0	0	23
Saint Marcel (Grotte)	GtSMa-3	C	CAVE	Neo	5400 - 4800	0	0	0	0	7	0	0	0	0	0	0	0	0	0	0	1	0	0	0	0	0	0	0	0	0	0	0	500
Saint Marcel (Grotte)	GtSMa-6	C	CAVE	BA	3300 - 3050	0	0	0	0	0	0	0	0	0	0	0	0	0	0	0	0	0	0	0	0	0	0	0	0	0	0	0	21
Saint Marcel (Oppidum)	OpStM-8	C	VILLAGE	IA1/2	2500 - 2480	0	0	0	0	0	0	0	0	0	0	0	0	0	0	0	3	0	0	0	0	0	0	0	0	0	0	0	17
Saint Marcel (Oppidum)	OpStM-9	C	VILLAGE	IA2	2400 - 2375	0	0	0	0	0	0	0	0	0	0	0	0	0	0	0	3	0	0	0	0	0	0	0	0	0	0	0	18
Saint Martin d'Azirou	SMAz-3	C	FUN	Neo	5500 - 4600	0	0	0	0	0	0	0	0	0	0	0	0	0	0	0	5	0	0	0	0	0	0	0	0	0	0	0	381
Saint Martin III	SMT3-4	C	RURAL	BA	4200 - 3650	0	0	0	0	0	0	0	0	0	0	0	0	0	0	0	0	0	0	0	0	0	0	0	4	0	0	0	6
Saint Pierre	StPie-8	C	VILLAGE	IA1/2	2550 - 2475	0	0	1	0	0	0	0	0	0	0	0	0	0	0	0	0	0	0	0	0	0	0	0	0	0	0	0	1
Saint Pierre	StPie-10	C	VILLAGE	ERo	2020 - 1800	0	0	0	0	0	0	0	0	0	0	0	0	0	0	0	0	0	0	0	0	0	0	0	0	0	0	0	0
Saint Romain de Jalionas	SRJ-10	C	RURAL	ERo	2000 - 1770	0	0	0	0	0	0	0	0	0	0	0	0	0	0	0	0	0	0	0	0	0	0	0	0	0	0	0	2
Saint Romain de Jalionas	SRJ-11	C	RURAL	LRo	1800 - 1600	0	0	0	0	0	0	0	0	0	0	0	0	0	0	0	0	0	0	0	0	0	0	0	0	0	0	0	6418
Saint Romain de Jalionas -T57	SRJ-10	U	OFFSITE	ERo	2000 - 1770	0	0	1498	33	0	0	17	0	0	0	9	0	0	0	85	1	0	1	55	106	0	0	770	370	0	0	1	75
Salle (La)	LaSal-2	C	RURAL	Neo	6000 - 5500	0	0	0	0	0	0	0	0	0	0	0	0	0	0	0	0	0	0	0	0	0	0	0	0	0	0	0	292
Salpétrière (grotte de)	GtSal-3	C	CAVE	Neo	4700 - 4200	0	0	1	0	0	0	0	0	0	0	0	0	0	0	0	0	0	0	0	0	0	0	0	0	0	0	0	8
Sant Jaume del Crest	CerSJC-10	C	RURAL	ERo	1925 - 1850	0	0	0	0	0	0	0	0	0	0	0	0	0	0	0	0	0	0	0	0	0	0	0	0	0	0	0	1
Sargel (Grotte)	GtSag-2	C	CAVE	Neo	6500 - 5500	0	0	0	0	0	0	0	0	0	0	0	0	0	0	0	1	0	0	0	0	0	0	0	0	0	0	0	0
Sargel (Grotte)	GtSag-3	C	CAVE	Neo	4800 - 4200	0	0	0	0	0	0	0	0	0	0	0	0	0	0	0	3	0	0	0	0	0	0	0	0	0	0	0	298
Sauzet/A7-Section 2	SauzA7-10	C	RURAL	ERo	1925 - 1900	0	0	0	0	0	0	0	0	0	0	0	0	0	0	0	0	0	0	0	0	0	0	0	0	0	0	0	113
Serre 1 (Le)	RS1-1	C	RURAL	Neo	7000 - 6500	0	0	0	0	0	0	0	0	0	0	0	0	0	0	0	0	0	0	0	0	0	0	0	0	0	0	0	1145
Serre 1 (Le)	RS1-4	C	RURAL	BA	4200 - 3900	0	0	2	0	0	0	0	0	0	0	0	0	0	0	1	47	0	0	0	0	0	0	0	0	0	0	0	1845
Serre de Boidon	SedBo-3	C	RURAL	Neo	4800 - 4200	0	0	0	0	0	0	0	0	0	0	0	0	0	0	0	1	0	0	0	0	0	0	0	0	0	0	0	4425
Sizen-Vigne	SizVig-9	C	FUN	IA2	2400 - 2200	0	0	34	0	0	0	0	0	0	0	0	0	0	0	4	0	0	0	0	0	0	0	0	0	0	0	0	5
Soumaltre	AsSou-10	C	RURAL	ERo	2000 - 1800	0	0	1141	5	0	0	0	0	0	0	0	0	0	0	0	0	0	0	0	0	0	0	0	0	0	0	0	10
Soumaltre - funerary	AsSouF-10	C	FUN	ERo	2000 - 1800	0	0	100	2	0	0	0	0	0	0	0	0	17	0	0	0	0	0	0	0	0	0	0	0	0	0	0	7
Taï (Le)	RTai-1	C	CAVE	Neo	7400 - 7000	0	0	82	0	12	0	30	0	182	0	0	0	0	23	4	4	0	0	0	32	0	0	159	3	0	0	0	527
Taï (Le)	RTai-2	C	CAVE	Neo	6500 - 5500	0	0	9	0	0	0	0	0	0	0	0	0	0	0	0	0	0	0	0	0	0	0	0	0	0	0	0	74
Taï (Le)	RTai-3	C	CAVE	Neo	5500 - 4200	0	0	6	0	0	0	0	0	3	0	0	0	0	0	1	1	0	0	0	0	0	0	10	0	0	0	0	540
Terrasses Montfo	MagTM-11	U	FUN	LRo	1700 - 1600	0	0	1059	292	0	0	3	0	3	0	0	0	0	0	1	8	0	0	0	5	0	0	7	146	0	0	0	4
Terrofort 268	CaTer-11	C	RURAL	LRo	1630 - 1550	0	0	1	0	0	0	0	0	0	0	0	0	0	0	0	0	0	0	0	0	0	0	0	0	0	0	0	0
Teste Nègre	TsNeg-9	C	VILLAGE	IA2	2300 - 2100	0	0	0	0	0	0	0	0	0	0	0	0	0	0	0	0	0	0	0	0	0	0	0	0	0	0	0	1432
Théâtre d'Agglomération	FrThAg-10	U	OFFSITE	ERo	2030 - 1970	0	0	2	0	0	0	0	0	0	0	0	0	0	0	0	0	0	0	0	52	0	0	0	10	0	0	0	123
Théâtre de Villeneuve	FrThVi-10a	U	OFFSITE	ERo	2025 - 1950	0	0	0	0	0	0	0	0	0	0	0	0	0	0	0	0	0	0	0	0	0	0	0	0	0	0	0	0
Théâtre de Villeneuve	FrThVi-10b	C	OFFSITE	ERo	1900 - 1800	0	0	0	0	0	0	0	0	0	0	0	0	0	0	0	0	0	0	0	0	0	0	0	0	0	0	0	3
Théâtre de Villeneuve	FrThVi-10b	U	OFFSITE	ERo	1900 - 1800	0	0	22	0	0	0	0	0	0	0	0	0	0	0	0	0	0	0	0	78	0	0	0	0	0	0	0	344
Théâtre de Villeneuve	FrThVi-11a	U	OFFSITE	LRo	1800 - 1700	0	0	28	0	0	0	0	1	0	0	0	0	0	0	0	1	0	0	0	2	0	0	0	0	0	0	0	0
Théâtre de Villeneuve	FrThVi-11b	C	OFFSITE	LRo	1600 - 1500	0	0	0	0	0	0	0	0	0	0	0	0	0	0	0	0	0	0	0	0	0	0	0	0	0	0	0	0
Théâtre de Villeneuve	FrThVi-11b	U	OFFSITE	LRo	1600 - 1500	0	0	6	0	0	0	0	0	0	0	0	0	0	0	0	0	0	0	0	0	0	0	0	0	0	0	0	0
Touar (Le)	Touar-6	C	RURAL	BA	3250 - 2900	0	0	0	0	0	0	0	0	0	0	0	0	0	0	0	0	0	0	0	0	0	0	0	0	0	0	0	3
Touar (Le)	Touar-7	C	RURAL	IA1	2650 - 2500	0	0	0	0	0	0	0	0	0	0	0	0	0	0	0	0	0	0	0	0	0	0	0	0	0	0	0	204
Toulons (Les)	RiToul-10	C	RURAL	ERo	1850 - 1800	0	0	154	0	0	1	0	0	0	0	0	0	0	0	0	0	0	0	0	0	0	0	0	0	0	0	0	24
Toulons (Les)	RiToul-11a	C	RURAL	LRo	1750 - 1700	0	0	0	0	0	0	0	0	0	0	0	0	0	0	0	0	0	0	0	0	0	0	0	0	0	0	0	23
Toulons (Les)	RiToul-11b	C	RURAL	LRo	1650 - 1600	0	0	33	0	0	0	0	0	0	0	0	0	0	0	0	0	0	0	0	0	0	0	0	0	0	0	0	5
Tourteix (Le)	OrTxt-4	C	RURAL	BA	3600 - 3200	0	0	0	0	0	0	0	0	0	0	0	0	0	0	0	0	0	0	0	0	0	0	1	0	0	0	0	62
Tremaïe	Trema-9	C	URBAN	IA2	2125 - 2100	0	0	13	0	0	0	0	0	0	0	0	0	0	0	0	0	0	0	0	0	0	0	0	0	0	0	0	0
Tumbas (Les)	CerTu-10	C	RURAL	ERo	2100 - 1900	0	0	1	0	0	0	0	0	0	0	0	0	0	0	0	0	0	0	0	0	0	0	0	0	0	0	0	1
Vaise	LyVais-1	C	RURAL	Neo	7200 - 6500	0	0	0	0	0	0	0	0	0	0	0	0	0	0	0	0	0	0	0	0	0	0	0	0	0	0	0	396
Vaise	LyVais-2	C	RURAL	Neo	6500 - 5500	0	0	0	0	0	0	0	0	0	0	0	0	0	0	0	4	0	0	0	0	0	0	0	0	0	0	0	91
Vaise - ZAC Industrie Nord	LyZIN-10	C	FUN	ERo	2025 - 1750	2	0	94	5	0	0	0	0	0	0	0	0	0	0	0	0	0	0	0	0	0	0	5	3	0	0	0	3405

Site	Cod Sit-Phas	Pres	type	Period	DATE BP	Quantif	MNI	Citrul	Cumel	Cupre	Cusat	Cusp	Jugla	Lage	Mespi	Moru	Prcer	Prdul	Prpers	Punic	Zizip	Cordi	Phoen	Celti	Coryl	Ficu	Fraga	Maloid	Malu	Olea	Pinpi	Pravce	Pravi	Prdom
Val du Fou	VaFou-7	C	RURAL	IA1	2650 - 2500	NMI	26	0	0	0	0	0	0	0	0	0	0	0	0	0	0	0	0	0	0	0	0	0	0	0	0	0	0	0
Valladas (Le)	SP3CVal-1	C	RURAL	Neo	7500 - 7000	%	103	0	0	0	0	0	0	0	0	0	0	0	0	0	0	0	0	0	0	0	0	0	0	0	0	0	0	0
Valladas (Le)	SP3CVal-10	C	FUN	ERo	1975 - 1800	NMI	62	0	0	0	0	0	5	0	0	0	0	1	0	0	0	0	20	0	1	14	0	0	0	7	1	0	0	0
Vautes (Les)	Vaute-3	C	RURAL	Neo	4700 - 4200	NMI	1518	0	0	0	0	0	0	0	0	0	0	0	0	0	0	0	0	0	0	0	0	0	0	0	0	0	0	0
Vayssière	Vayss-10	C	FUN	ERo	2000 - 1800	NMI	7602	0	0	0	0	0	1	0	2	0	0	0	0	0	0	0	0	0	2	1	0	0	4	0	0	0	0	6
Véreître	CLVer-6	C	RURAL	BA	3300 - 3050	NMI	83	0	0	0	0	0	0	0	0	0	0	0	0	0	0	0	0	0	2	0	0	0	0	0	0	0	0	0
Vergers (Les)	CaVgrs-10	C	OFFSITE	ERo	1900 - 1900	NMI	87	0	0	0	0	0	0	0	0	0	0	0	0	0	0	0	0	0	0	0	0	0	0	0	0	0	0	0
Vieux-Mounoï (Grotte)	VxMou-1	C	CAVE	Neo	7500 - 6500	NMI	3	0	0	0	0	0	0	0	0	0	0	0	0	0	0	0	0	0	0	0	0	0	0	0	0	0	0	0
Vieux-Mounoï (Grotte)	VxMou-2	C	CAVE	Neo	6500 - 5500	P/A	16	0	0	0	0	0	0	0	0	0	0	0	0	0	0	0	0	0	0	0	0	0	0	0	0	0	0	0
Vieux-Mounoï (Grotte)	VxMou-6	C	CAVE	BA	3350 - 2800	NMI	2	0	0	0	0	0	0	0	0	0	0	0	0	0	0	0	0	0	0	0	0	0	0	0	0	0	0	0
Vignes de l'Espérance	ViEsp-9	C	RURAL	IA2	2500 - 2300	P/A	9	0	0	0	0	0	0	0	0	0	0	0	0	0	0	0	0	0	0	0	0	0	0	0	0	0	0	0
Villa Giribaldi	ViGir-2	C	RURAL	Neo	6500 - 6000	NMI	561	0	0	0	0	0	0	0	0	0	0	0	0	0	0	0	0	0	0	0	0	0	0	0	0	0	0	0
Villelongue	Villg-9	U	RURAL	IA2	2125 - 2025	NMI	31	0	0	0	0	0	0	0	0	0	0	0	0	0	0	0	0	0	0	0	0	0	0	0	0	0	0	0
Villelongue	Villg-10	U	RURAL	ERo	2025 - 1975	NMI	36	0	0	0	0	0	2	0	0	0	0	0	0	0	0	0	0	0	0	0	0	0	0	0	0	0	0	0
Zac de la Burlière	ZACBrl-2	C	RURAL	Neo	6500 - 5500	NMI	12699	0	0	0	0	0	0	0	0	0	0	0	0	0	0	0	0	0	0	0	0	0	0	0	0	0	0	0
ZAC de Sagnon	ZACSag-10	C	FUN	ERo	1970 - 1830	NMI	541	0	0	0	0	0	0	0	0	0	0	0	0	0	0	0	0	0	0	1	0	0	0	38	0	2	0	0

Site	Cod Sit-Phas	Pres	type	Period	DATE BP	Prsp	Pyru	Viti	Vitipd	Arbu	Corma	Corsa	Crata	Junsp	Myrt	Physa	Pincem	Pinsp	Pista	Prspi	Querc	Rhus	Rosa	Rubca	Rubfr	Rubid	Rubsp	Sambeb	Sambni	Sambsp	Sorb	Vibu	An crops
Val du Fou	VaFou-7	C	RURAL	IA1	2650 - 2500	0	0	4	0	0	0	0	0	0	0	0	0	0	0	0	0	0	0	0	0	0	0	0	0	0	0	0	22
Valladas (Le)	SP3CVal-1	C	RURAL	Neo	7500 - 7000	0	0	0	0	0	0	0	0	0	0	0	0	0	0	0	0	0	0	0	0	0	0	0	0	0	0	0	103
Valladas (Le)	SP3CVal-10	C	FUN	ERo	1975 - 1800	0	0	12	0	0	0	0	0	0	0	0	0	0	0	0	0	0	0	0	0	0	0	0	0	0	0	0	1
Vautes (Les)	Vaute-3	C	RURAL	Neo	4700 - 4200	0	0	0	0	0	0	0	0	0	0	0	0	0	0	0	1502	0	0	0	0	0	0	0	0	0	0	0	16
Vayssière	Vayss-10	C	FUN	ERo	2000 - 1800	0	0	5	0	0	0	0	0	0	0	0	0	0	0	1	0	0	1	0	0	0	0	0	0	0	0	0	7579
Véreître	CLVer-6	C	RURAL	BA	3300 - 3050	0	0	0	0	0	0	0	0	0	0	0	0	0	0	0	0	0	0	0	0	0	0	1	0	0	0	0	80
Vergers (Les)	CaVgrs-10	C	OFFSITE	ERo	1900 - 1900	0	0	54	5	0	0	0	0	0	0	0	0	0	0	0	0	0	0	0	0	0	0	0	0	0	0	0	28
Vieux-Mounoï (Grotte)	VxMou-1	C	CAVE	Neo	7500 - 6500	0	0	0	0	0	0	0	0	0	0	0	0	1	0	0	1	0	0	0	0	0	0	0	0	0	0	0	1
Vieux-Mounoï (Grotte)	VxMou-2	C	CAVE	Neo	6500 - 5500	0	0	0	0	0	0	0	0	3	0	0	0	0	0	0	9	0	0	0	0	0	0	0	0	0	0	0	4
Vieux-Mounoï (Grotte)	VxMou-6	C	CAVE	BA	3350 - 2800	0	0	0	0	0	0	0	0	0	0	0	0	0	0	0	2	0	0	0	0	0	0	0	0	0	0	0	0
Vignes de l'Espérance	ViEsp-9	C	RURAL	IA2	2500 - 2300	0	0	0	0	0	0	0	0	0	0	0	0	0	0	1	0	0	0	0	0	0	0	0	0	0	0	0	8
Villa Giribaldi	ViGir-2	C	RURAL	Neo	6500 - 6000	0	0	0	0	0	0	0	0	0	0	0	0	0	0	0	7	0	0	0	0	0	0	0	0	0	0	0	554
Villelongue	Villg-9	U	RURAL	IA2	2125 - 2025	0	0	2	0	0	0	2	0	0	0	0	0	0	0	0	1	0	0	0	11	0	0	15	0	0	0	0	0
Villelongue	Villg-10	U	RURAL	ERo	2025 - 1975	1	0	8	0	0	0	0	0	0	0	0	0	0	0	0	0	0	0	0	18	0	0	0	0	1	0	0	6
Zac de la Burlière	ZACBrl-2	C	RURAL	Neo	6500 - 5500	0	0	0	0	0	0	0	0	9	0	0	0	0	0	0	0	0	0	0	0	0	0	0	0	0	0	0	12690
ZAC de Sagnon	ZACSag-10	C	FUN	ERo	1970 - 1830	0	0	30	1	0	0	0	0	0	0	0	0	0	0	0	0	0	0	0	0	0	0	0	0	0	0	0	469
